# Orchestrating Anion–π^+^ Interactions to Accelerate Metal‐Free Room‐Temperature Phosphorescence

**DOI:** 10.1002/anie.4386683

**Published:** 2026-08-28

**Authors:** Eetu Hakkarainen, Corentin Montagne, Hao‐Cheng Lin, Chen‐Yu Lin, Khai‐Nghi Truong, Po‐Yu Chen, Anton Nechaev, Fabien B. L. Cougnon, Igor O. Koshevoy, Toni Eskelinen, Hao‐Wu Lin, Pi‐Tai Chou, Andrey Belyaev

**Affiliations:** ^1^ Department of Chemistry and Sustainable Technology University of Eastern Finland Joensuu Finland; ^2^ Department of Materials Science and Engineering National Tsing‐Hua University Hsinchu Taiwan; ^3^ Department of Chemistry National Taiwan University Taipei Taiwan; ^4^ Rigaku Europe SE Neu‐Isenburg Germany; ^5^ Advanced Packaging Instrumentation and Metrology Laboratory Industrial Technology Research Institute Hsinchu Taiwan; ^6^ Department of Chemistry Nanoscience Center University of Jyväskylä Jyväskylä Finland; ^7^ Department of Chemistry and Materials Science Aalto University Aalto Finland; ^8^ Institute For Chemical Research Kyoto University Kyoto Japan

**Keywords:** anion–π, charge‐transfer, phosphorescence, pyridinium, radiative rate

## Abstract

Overcoming the long‐standing trade‐off between efficiency and radiative rate in metal‐free phosphorescence remains a central challenge in molecular photophysics and is pivotal for the next‐generation optoelectronic technologies. Here, we demonstrate that tuning the strength of anion–π^+^ interactions in pyridinium‐based contact ion pairs (CIPs) enables exceptionally fast room‐temperature phosphorescence (RTP) spanning 540 to 605 nm and, with radiative rates reaching 5.2×10^5^ s^−1^, places this crystalline material among the fastest known organic phosphors. By systematically tuning electron deficiency, steric confinement, and CIP organization through mono‐ and dicarboxyester‐functionalized pyridinium iodides, we achieved unusually short and highly polarized I^−^–π^+^ tunable contacts that approach 3.50 Å. Structural analysis reveals not only tightly bound, sandwich‐like, and columnar‐organized ion pairs but also unexpected dynamic behavior, including reversible, thermally activated anion drift in the solid state. Collectively, these solid state features engender strongly coupled iodide → π^+^ charge‐transfer states with enhanced spin–orbit coupling, thereby promoting ultrafast intersystem crossing. Spectroscopic and theoretical studies identify an intensity‐borrowing mechanism within the charge‐transfer manifold that underlies the accelerated phosphorescence and/or thermally stimulated delayed phosphorescence. The resulting materials show promising X‐ray scintillation performance. Our work establishes anion–π^+^ engineering as a general and previously underexploited strategy for achieving high‐rate, metal‐free phosphorescence.

## Graphical abstract

Tuning anion–π^+^ interactions in pyridinium iodide contact ion pairs enables ultrafast metal‐free room‐temperature phosphorescence (540–605 nm) with radiative rates up to 5.2×10^5^ s^−1^. Strong iodide→π^+^ charge‐transfer coupling, enhanced spin–orbit interactions, and dynamic ion‐pair organization accelerate intersystem crossing and emission, establishing anion–π^+^ engineering as a powerful strategy for high‐performance organic phosphors.

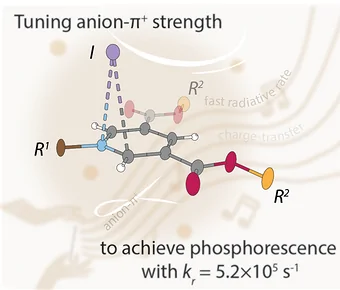

## 1 Introduction

Questioning the existence of soft anion–π interactions back in the early 2000s marked a decisive moment in supramolecular chemistry and established a novel dimension for the exploration and manipulation of noncovalent interactions [1, 2]. Initially met with skepticism due to the apparent electrostatic repulsion between negatively charged species and the filled orbitals of π‐systems, subsequent theoretical and experimental studies demonstrated that electron‐deficient aromatic rings could indeed favorably interact with anions through polarization and quadrupolar interactions at energies of ∼2–12 kcal/mol [3]. This breakthrough not only validated anion–π interactions as a powerful noncovalent tool but also expanded the conceptual framework of supramolecular design [4]. Since then, anion–π interactions have been harnessed in a variety of applications, including ion transporting [5, 6, 7], crystal engineering, regulation of phase purity [8], molecular recognition [9, 10], anion–π templating catalysis [11, 12], and the construction of self‐assembled architectures [13, 14]. In addition to fundamental importance, these examples highlight the significance of anion–π contacts for the development of versatility of functional materials [10, 15].

In contrast to more scrutinized three‐component adducts composed of neutral organic π‐acidic fragments (e.g., naphthalenediimides, tetra‐ and triazines, and polyfluoro‐ or cyanobenzenes) and alkylammonium salts [16], exploration of anion–π^+^ interactions within intrinsically ionic bi‐component systems (i.e., anion–cation) has only recently gained momentum, and offered a compelling strategy to advance optoelectronic and bioimaging applications [15, 17, 18, 19, 20]. Intuitively, energetic favorability of the attraction between anions and electron‐deficient π‐systems is anticipated to be enhanced electrostatically when an aromatic molecular scaffold carries a positive charge. For instance, in phosphonium, oxazolium, or quinolinium salts, the presence of a strong Coulombic component gives rise to a highly polarized anion(X^−^)–π^+^ contact ion‐pairing: a feature observed as a short anion–π(centroid) distance being smaller than (∑vdW radii + 0.2 Å) in structurally analyzed compounds. Importantly, these stabilizing interactions facilitate orbital mixing and the formation of ionic charge‐transfer (CT) complexes. In these systems, the nature of the ions and the energies of both absorption and photoemission follow a Mulliken‐type correlation [21], which reveals a delicate way of tuning the photophysical properties (Figure 1). This effect is particularly pronounced for crystalline materials, where anion–π^+^ interactions promote room‐temperature phosphorescence (RTP) via the heavy atom effect, thereby enabling applications in anti‐counterfeiting, photochromic materials, data encryption, and scintillation technologies [22, 23, 24]. In a rigid crystalline environment, the synergy between the properties of a counterion and the polarization of a π^+^‐system increases spin–orbit coupling (SOC), which accelerates intersystem crossing (ISC) and promotes efficient organic phosphorescence originating from an anion→π^+^ CT excited state. In particular, the approach demonstrated by Partanen et al. for diphosphonium diiodide contact ion pairs (CIPs) illustrates a facile means of control over frontier molecular orbitals (LUMO control, Figure 1A) that enables wide alteration of emission energies (*λ*_em_ ranges from 550 to 880 nm), complemented by fast radiative rates up to 2.8×10^5^ s^−1^ [25]. Apart from the HOMO control (Figure 1B) [26], a common feature resulting from halide variations in isostructural salts is the ability to regulate the radiative rate that was ultimately described by the groups of Tang [27, 28], Kuang [29], and Zhang [30]. Consequently, the tunability of the emission mechanism varying from prompt fluorescence (typically for PF_6_^−^/F^−^/Cl^−^ anions), long‐lived phosphorescence/dual emission (for Cl^−^, Br^−^) or phosphorescence (for I^−^), was exemplified by a 1,2,3,4‐tetraphenyloxazolium salt and reflects the balance in the population of the S_n_ and T_n_ states influenced by a heavy anion (*k*_ISC_ ∝ *Z* ^8^/*r* ^6^, where *Z* and *r* denote the atomic number and the distance to the emissive motif). Equally critical is the proximity of the halide to the emissive motif, as we have recently demonstrated, underscoring the necessity of maintaining minimal spatial separation to maximize ISC efficiency and affect the excited‐state dynamics [31].

**FIGURE 1:**
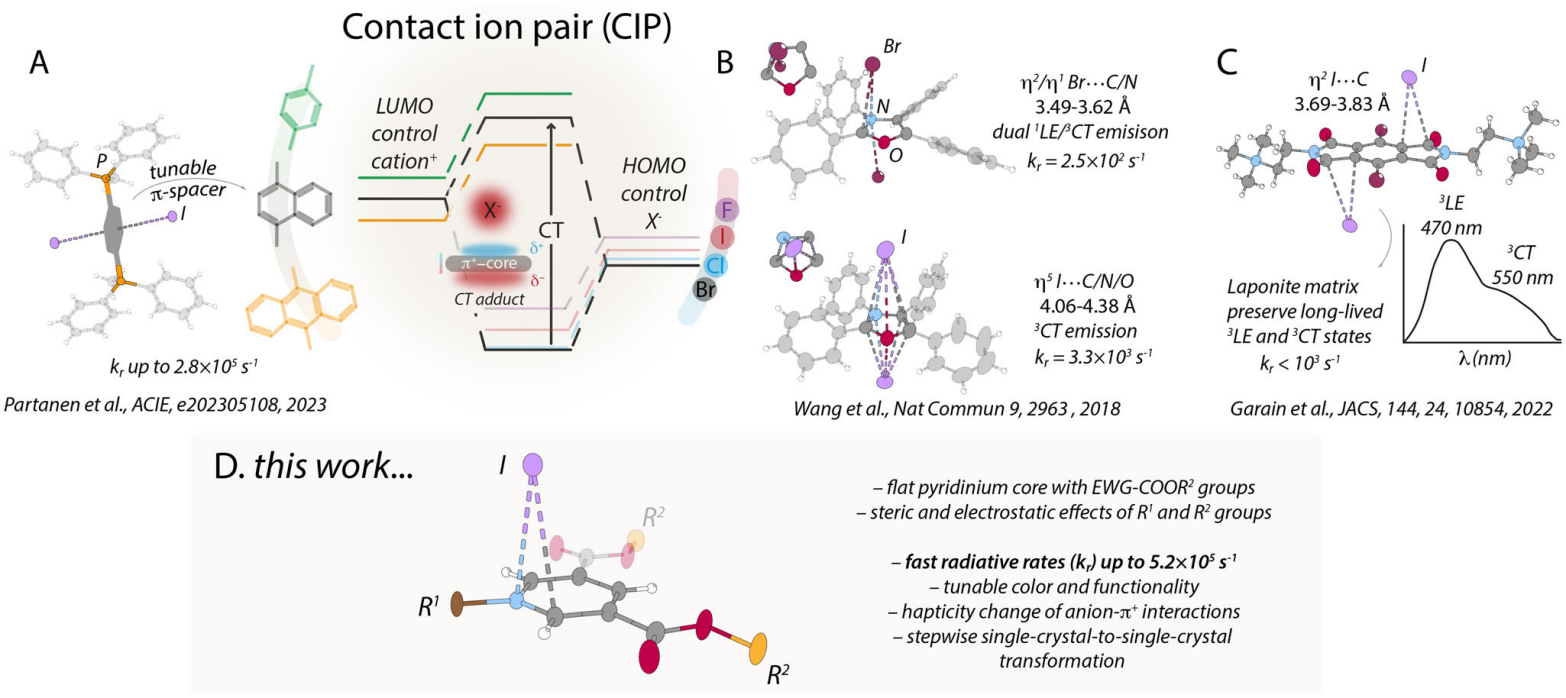
Diagram of the charge‐transfer adduct and representative strategies for modulating photophysical behavior in contact ion pairs (CIPs). (A) Variation of the LUMO energy levels in diphosphonium diiodide CIPs by means of the conjugated core. (B) Control of the excited‐state dynamics in CIPs via counterion selection. (C) Restriction of ion mobility by integrating CIPs into a Laponite matrix to preserve long‐lived emission. (D) Design principles underlying the current study.

Manipulating the desired order of ion pairs in the solid state, and especially translating such organization into solution or thin films, remains a critical challenge that restricts broad practical utility of CIPs. The inherent high mobility of both cations and anions typically leads to a complex equilibrium involving relaxation of fully solvated, solvent‐shared, and separated ion‐pair states [32, 33], or to the formation of non‐uniform amorphous or polymorphic crystalline phases. Moreover, frontier orbitals are perturbed by polar solvents, which effectively reduce the LUMO and lead to a possible irreversible anion‐induced thermal or photoelectron transfer, generating persistent reactive radicals, *σ*‐complexes or Meisenheimer intermediates, hence eroding chemical and photostability [34, 35]. Under such conditions, anion–π^+^ interactions are disrupted, undermining supramolecular assembly crucial for functional behavior. This disruption often permits the domination of non‐radiative decays, which causes a complete loss of intrinsic photoluminescence properties. To address this challenge, George and coworkers embedded the dicationic pyromellitic diimide CIP into a rigid Laponite nanoplate matrix that partially suppressed ion mobility and preserved the spatial arrangement required for anion–π^+^ engagement (Figure 1C) [36, 37]. Although this approach allowed for some retention of millisecond‐long‐lived emission, that is, only slow radiative rates, associated with the anion–π^+^ CT in soft materials, the resulting material shows multiple CIP associates.

In this work, we aim to orchestrate the cooperativity of electronic, steric, and lipophilic effects within the pyridinium core to develop selective, robust and efficient CIPs. This approach enables the corresponding phosphorescence among the fastest known organic, metal‐free phosphors, with *k*_r_ up to 5.2×10^5^ s^−1^, thereby broadening the functional relevance of anion–π^+^ interactions.

## 2 Results and Discussion

### 2.1 Design Considerations

Several key principles guide the design of charge‐assisted CIPs in this work. We employ a planar pyridinium scaffold that provides accessible and tunable sites for anion–π^+^ interactions (Figure 1D). Pyridinium derivatives are well‐established electron acceptors and, when incorporated into covalent donor–acceptor (D–A) architectures, frequently exhibit efficient fluorescence originating from intramolecular charge‐transfer states [38, 39]. In contrast, simple pyridinium salts are generally weakly or non‐emissive because non‐radiative relaxation dominates and triplet formation is inefficient. Although pyridinium salts are well known for ion pairing and charge‐transfer (CT) absorption in solution [40, 41], their use as phosphorescent CIPs remains largely unexplored, likely due to elongated anion–π^+^ contacts that limit ISC and favor non‐radiative decay. To address existing limitations, we aim to enhance Coulombic attraction by introducing electron‐withdrawing ester groups, thereby strengthening ion pairing and promoting preorganization in both solution and the solid state. Systematic variation of substitution pattern and steric bulk enables control over anion proximity, solubility, and self‐solvation, while increased lipophilicity facilitates processing in non‐polar media [42, 43].

### 2.2 Synthesis and Characterization

Pyridinium salts decorated with one methoxycarbonyl substituent *ortho*‐(*o‐*), *meta*‐(*m‐*), *para(p)*‐**pyI** (group I, analogs of Kosower's salts) [41] were accessed via the alkylation of mono‐carboxy ester pyridines (**MP**) with methyl iodide in nearly quantitative yields (Scheme 1, see Supporting Information for more details). Similarly, treatment of dimethyl pyridine‐3,5‐dicarboxylate (**DP**, group II) in oxygen‐free conditions with R^1^I (R^1^ = Me, Et, Pr, and *^i^* Pr) delivers **1–4** with gradually descending yields (94% → 89% → 54% → 21%), reflecting the decreased reactivity of the carbocations. We further modified **DP** by introducing ancillary chiral menthol ester groups (–**^/+^M**: (–)‐ or (+)‐menthol) via reacting –**^/+^M** with 3,5‐pyridinedicarbonyl dichloride (see Supporting Information for more details). Alternatively, menthol in **6** ^Br^ was introduced using (–)‐menthyl‐2‐bromoacetate (–**ACMBr**) as an alkylating agent, followed by conversion of **6** ^Br^ to **6** via metathesis with excess KI in methanol/dichloromethane.

**SCHEME 1:**
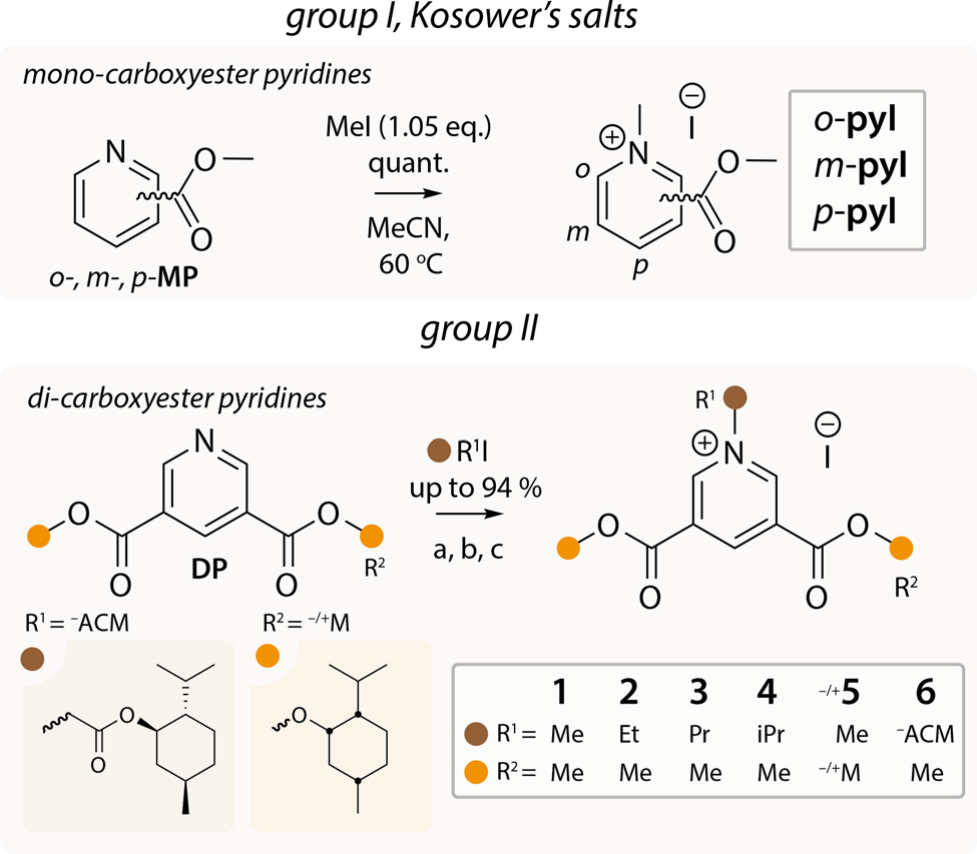
Synthesis of studied pyridinium salts *o*‐, *m*‐, *p*‐**pyI**, and **1–6**: ^a^R^1^I, 1.5–2.5 eq., (50°C–60°C, MeCN); ^b^two‐step approach using dimethyl/diethyl carbonate (140°C, MeOH) and KI [44]; ^c^ **6** was synthesized using (–)‐menthyl‐2‐bromoacetate (–**ACMBr**) with subsequent conversion of **6** ^Br^ to **6** with KI in methanol/dichloromethane; ^*^ **1** ^OTf^, **1** ^Br^, and **1** ^BARF^ were synthesized using MeOTf with subsequent metathesis with KBr/NaBARF, (BARF = tetrakis[3,5‐bis(trifluoromethyl)phenyl]borate), respectively (see Supporting Information); • chiral carbon atom.

After recrystallization, the titled salts were isolated as uniform white (**1** ^Br^, **6** ^Br^, **1** ^BARF^, and **1** ^OTf^), pale (*m*‐ and *o*‐**pyI**), yellow (**1–3**, **–^/+^5**, and **6**) or orange (*p*‐**pyI**, **4**) crystalline solids. Thermal analysis (TGA, DSC) revealed that the samples remained thermally stable after melting at temperatures up to 480 K (Figures S1 and S2). The composition and purity of the synthesized compounds were confirmed using a combination of nuclear magnetic resonance spectroscopy and high‐performance liquid chromatography (Figures S3 and S4; for more details, see Supporting Information).

### 2.3 Solution studies

In dichloromethane (DCM), **1–6** exhibit similar pronounced low‐energy broad CT absorption bands (*λ*_abs_ = 425 nm), whereas the CT bands of *o,m,p*‐**pyI** vary from 400 to 440 nm (Figure 2; Figures S5 and S6). These bands originate from iodide→π* transitions, highlighting the persistence of strong ion pairing even in moderately polar solvents (see Figures S5 and S7; Table S1). Further support for the CT nature of these states is provided by solvatochromic studies of –**5**, which exhibits a pronounced solvent dependence of its absorption spectra (Figures 2 (inset) and S6). The most significant bathochromic shifts are observed in toluene, benzene, and diethyl ether, where broad low‐energy absorption tails extend beyond 600 nm, indicating enhanced stabilization of the CT state through ion‐pair and solvent interactions. In contrast, acetonitrile and hexane exhibit hypsochromically shifted absorption spectra with no discernible low‐energy tail, highlighting the strong sensitivity of the CT transition to the surrounding medium. Figure 2B shows the evolution of the UV–vis absorption spectra of **1** ^BARF^ (10^−3^ M, DCM) upon titration with ^n^Bu_4_I (0.1 M, DCM); the inset highlights the growth in intensity at 426 nm with increasing iodide concentration and the corresponding fit to the binding isotherm. The calculated ion‐pairing constant of **1** ^BARF^ (K_eff_ = 153 M^−1^ for **1** ^BARF^ + I^−^ ⇆ **1** + BARF–) is higher than that of the reference Kosover salt *p*‐**py** ^BARF^ (K_eff_ = 9 M^−1^) [40], indicating substantially stronger cation–anion interactions resulting from the introduction of a secondary electron‐withdrawing group.

**FIGURE 2:**
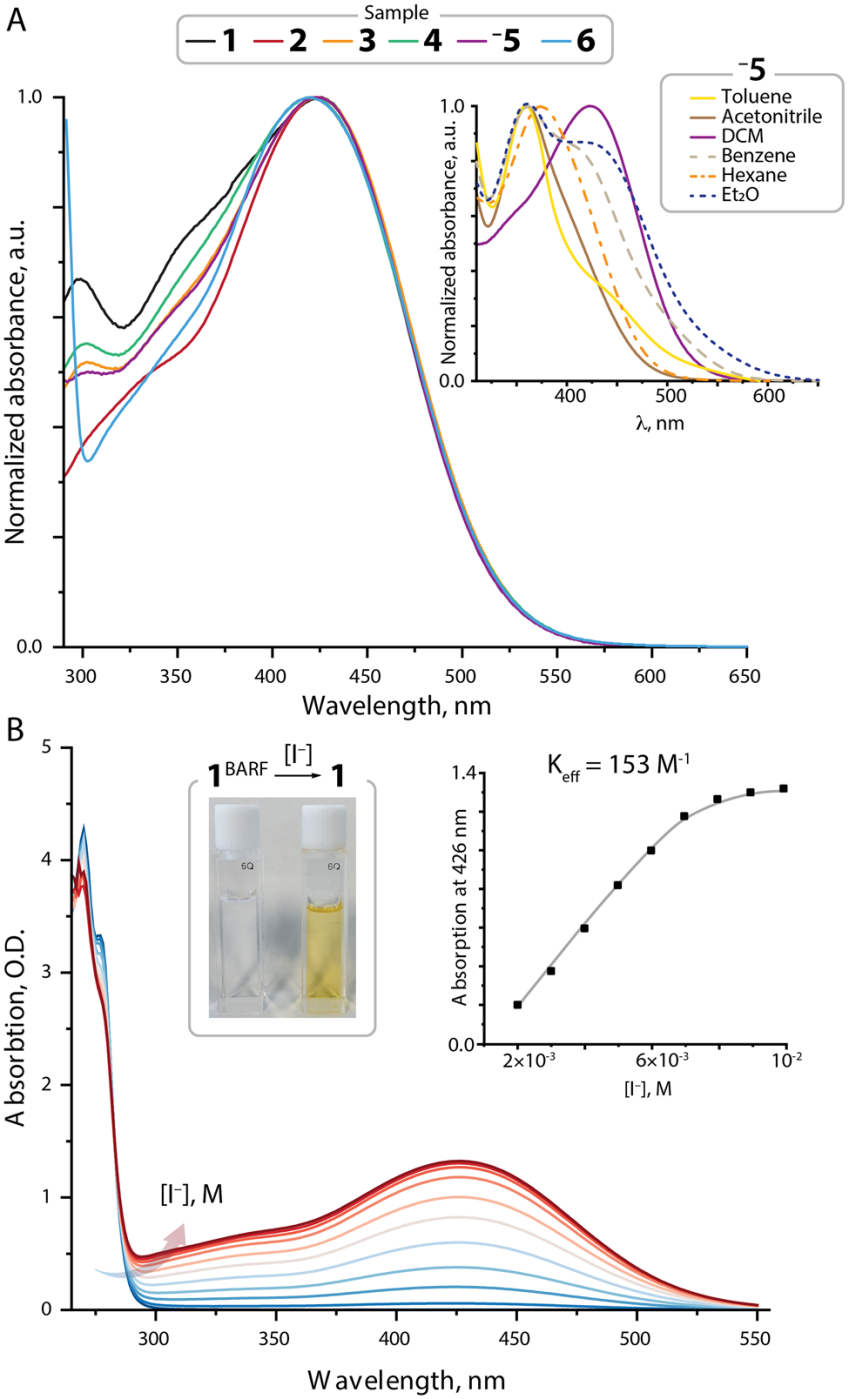
(A) Normalized absorption profiles of **1–6** (10^−4^ M, DCM). (B) Evolution of the UV–vis absorption spectra of **1** ^BARF^ (10^−3^ M, DCM, blue trace) upon titration with *^n^* Bu_4_I (0.1 M, DCM). The inset shows in (A) normalized absorption profiles of **^−^5** and in (B) the increase in intensity at 426 nm upon addition of iodide and the fit to the binding isotherm.

Despite evidence for tight ion pairing in **1–6**, they remain non‐emissive in solution unless frozen to 77 K (Figures S8–S10 and Table S2), indicating that efficient non‐radiative decay pathways dominate under these conditions. Steady‐state and time‐resolved measurements of frozen matrices confirm that all contact ion pairs (**1–6**) exhibit broad anion→π^+^ CT phosphorescence (*λ*_em_ = 540–597 nm) with microsecond lifetimes (*τ*_av_ = 0.87–3.49 µs), while the solvent dependence observed for –**5** demonstrates that the CT excited state is affected by ion‐pairing and solvation even in frozen matrices (Table S2). Solutions of –**5** in a variety of solvents are stable at 297 K during absorption measurements but undergo decomposition upon relatively intense photoexcitation, precluding reliable evaluation of its emission behavior (Figure S11).

### 2.4 Crystallographic Studies

Pyridinium salts *o‐* **pyI**, **1–4**, **1** *^X^* (*X* = Br, OTf, and BARF) and **^−^5** were characterized by single‐crystal X‐ray diffraction (SCXRD, XtaLAB Synergy‐R, Rigaku), while micropowders of *m‐* **pyI** and *p‐* **pyI** were analyzed using continuous rotation three‐dimensional electron diffraction (3DED, XtaLAB Synergy‐ED, Rigaku) (see Figures 3 and 4; Figures S12–S18; Tables S3–S8) [45].

**FIGURE 3:**
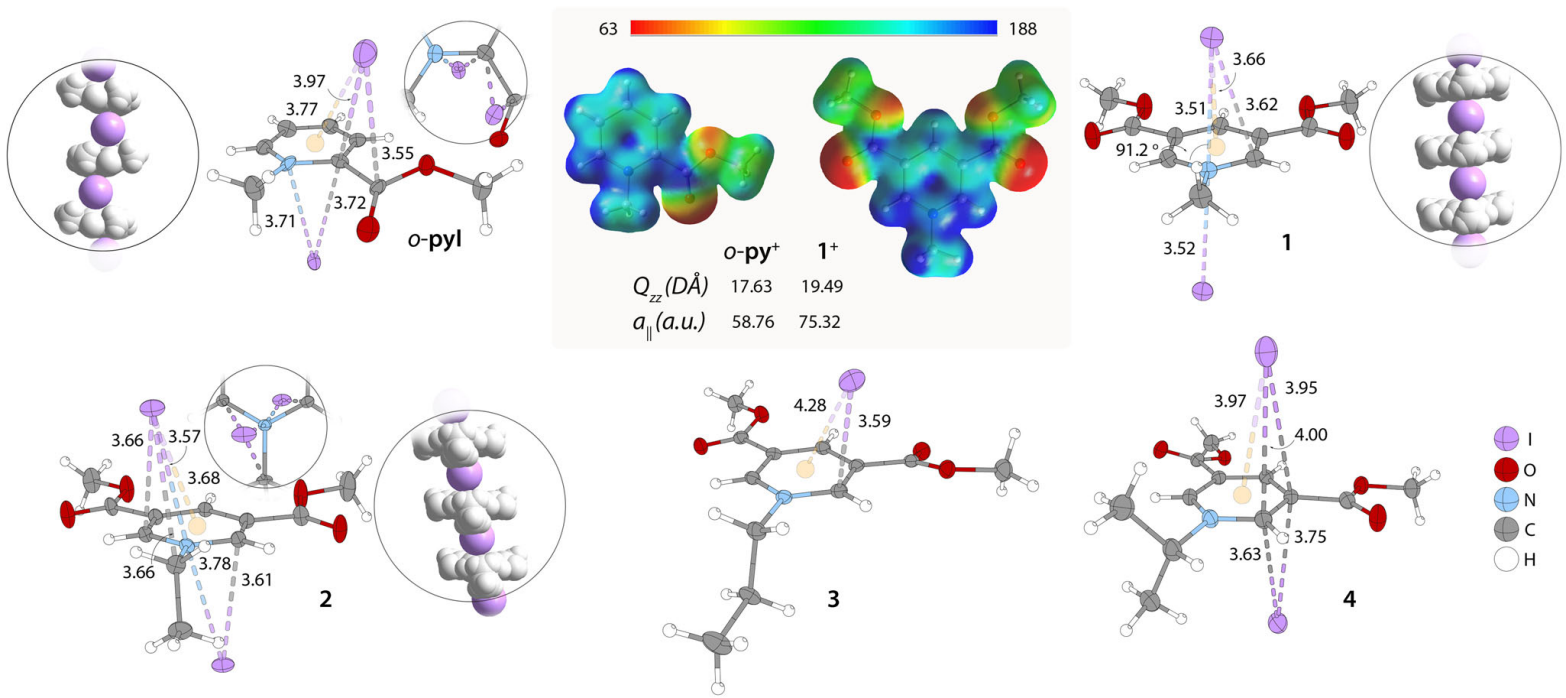
Molecular structures of ion pairs *o‐* **pyI** and **1–4** at 150 K with depicted shortest I⋯C/I⋯N/I⋯centroid distances (SCXRD, displacement ellipsoids are shown at the 50% probability level, the second anion in sandwich of I^−^‐(pyridinium^+^)–I^−^ fragment was extracted from packing). Insets show electrostatic potential (ESP, in kcal/mol) maps of *o‐* **py** ^+^ and **1** ^+^ cations, projections of I⋯C and I⋯N interactions over the pyridine plane, and fragments of packing. Bond lengths are given in Ångström (Å).

**FIGURE 4:**
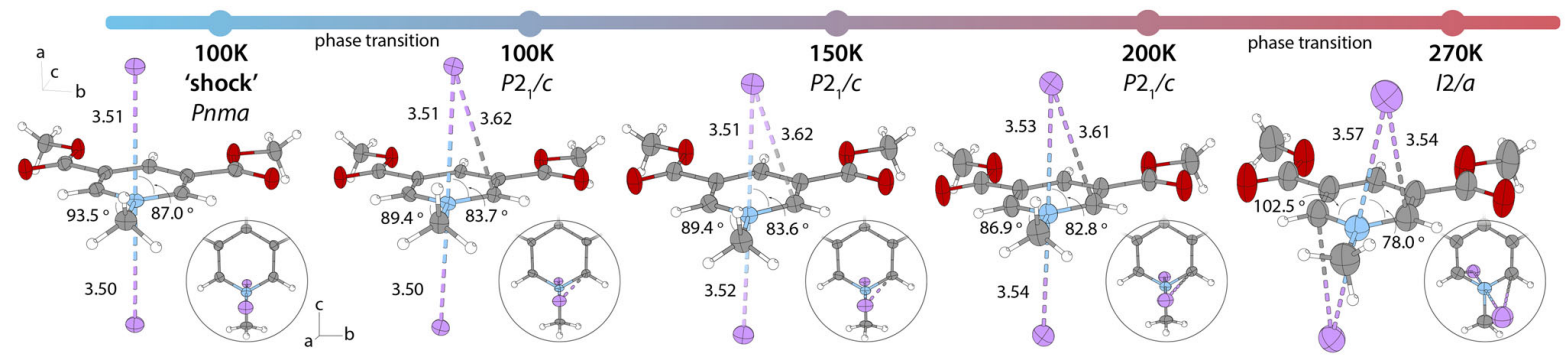
Thermally induced drift of iodide in the solid state upon cooling–heating cycling: molecular sandwich I^−^‐**1^+^**‐I^−^ at variable temperatures (100–270 K) featuring the shortest I⋯C and I⋯N distances (displacement ellipsoids are shown at the 50% probability level). Bond lengths are given in Ångström (Å).

All pyridinium iodides exhibit distinct anion(I^−^)–π(pyridinium^+^) interactions, with contact (*η*–anion–π interaction mode) and I^…^centroid distances ranging from 3.44 to 3.78 Å and 3.66 to 4.28 Å, respectively (Figures 3 and S13–S18). The packing of *o*‐**pyI** adopts a columnar alternating arrangement of pyridinium and iodide units, characterized by two distinct anion–π^+^ communications. One involves *η* ^2^ contacts (3.710(2)/3.726(2) Å), while the other features an additional short contact between the iodide and the carbon atom of the carboxy‐fragment, with an I···C separation of just 3.553(2) Å, that is, 0.13 Å shorter than the sum of the corresponding van der Waals radii (96.5% of *r*_vdW_[I,C] = 3.68 Å). This unusual orientation is consistent with the calculated electrostatic potential (ESP, Figures 3 and S19) surface of *o*‐**pyI**, which reveals a pronounced positive potential at the C‐carboxylate atom, on the side of the pyridinium core. By contrast, in the monosubstituted derivatives, *m*‐**pyI** and *p*‐**pyI** (Figure S16), the crystal structures feature a single set of slightly out‐of‐plane interactions: an *η* ^1^‐type contact of 3.706(2) Å in *m*‐**pyI**, and an *η* ^2^‐type contact of 3.707(1)/3.711(1) Å in *p*‐**pyI** [46].

In **1**, bearing two carboxylate ester substituents, the anion(I^−^)–π(pyridinium^+^) interactions are significantly stronger, giving rise to two short contacts: 3.521(3) Å (*η* ^1^‐type, I⋯N) and 3.514(1)/3.616(4) Å (*η* ^2^‐type, for I⋯N/I⋯C, respectively), which together define a sandwich‐like arrangement around the pyridine motif. The remarkably short distances (99.4% of *r*_vdW_[I,N] and 98.4% of *r*_vdW_[I,C]) are unprecedented for known neutral and charged anion(X–)‐π^+^ adducts, and, perhaps, stem from enhanced Coulomb attraction introduced by two electron‐withdrawing groups. Indeed, the energy decomposition analysis between the cation and anion(s) indicates that the electrostatic contribution in **1** is more favorable by 4.5–12.9 kcal/mol compared to pyridinium derivatives bearing a mono‐carboxyester substituent (see Table S9). Similarly, the total interaction energy of the sandwich species I^−^–**X** ^+^–I^−^ (*X* = **1** vs. *p*‐**pyI**) differs significantly, with the dicarboxyester adducts stabilized by ≈18 kcal/mol (≈28%). The computed *Q*_zz_ components of the quadrupole tensor for cations were found to be in a similar range (**1^+^** 19.49 vs. *o*‐**py^+^** 17.63/*p*‐**py^+^** 19.41 DÅ). In contrast, the predicted polarizability for **1^+^** (*a*_||_ = 75.32 a.u.) is significantly larger than those for *o*‐**py^+^**/*p*‐**py^+^** (58.76/59.74 a.u.), hence aligning well with the possibility for stronger anion–π^+^ communication in **1** [47]. The above interpretation is independently supported by Bader's QTAIM analysis based on the DFT‐calculated electron density (Table S10 and Figure S20), which identifies bond critical points (BCP) between the iodide anion and the pyridinium framework in **1** and x‐**pyI**. Compared with x‐**pyI**, the dicarboxyester contact ion pair **1** (and the I^−^–**1** ^+^–I^−^ sandwich configuration) exhibits higher electron densities at BCP (*ρ* = 0.061–0.064 vs. 0.048–0.055 e·Å^−3^) together with larger positive Laplacians (∇^2^ *ρ* = 0.67–0.69 vs. 0.38–0.49 e·Å^−5^). In all cases, the |V|/G ratios remain below unity (0.79–0.88), and the total energy density is positive, confirming that these exceptionally short contacts correspond to strong, closed‐shell, electrostatically dominated anion–π^+^ interactions. These descriptors independently corroborate the crystallographic observation that the dicarboxyester framework strengthens the anion–π^+^ interactions compared with the monosubstituted x‐**pyI** analogues.

Steric hindrance alteration of alkyl tails in **2–4** allows for fine tuning of the ion pairing: (i) iodide ions gradually shift from the close to N^+^‐vertical position toward more peripheral localizations, and (ii) the shortest anion–cation contacts systematically lengthen, that is, from unusual *η* ^3^‐type involving alkyl sp^3–^carbon atom (3.571(3)/3.661(3)/3.657(2) Å) in **2**, to 3.588(3) Å for *η* ^1^(C_1_) in **3**, and short 3.633(3)/3.749(2) Å and long 3.951(3)/4.002(3) Å sets for *η* ^2^(C_1_C_2_) in **4**. The crystal structures of **3** and **4** no longer display the alternating columnar arrangement observed in **1** and **2** (see insets, Figure 3). Compared with *o*‐**pyI**, the alternating (I^−^‐**1^+^/2^+^**)−(I^−^‐**1^+^/2^+^**) units intercalate in a tail‐to‐tail fashion, forming either slightly angled (**1**) or parallel (**2**) columns along the *a–*axis, connected through inter‐pairing of the carboxy ester fragments. The anion–π^+^ interactions are complemented by extensive hydrogen bonds, which predominantly involve the acidic N‐alkyl and methyl‐ester H‐donors interacting with either iodide anions or ester oxygen atoms. This hydrogen‐bonded supramolecular network establishes tight ion‐pair assemblies and guides the columnar or intercalated packing modes observed across the series.

Unexpectedly, placing a single crystal of **1** under a cryostream at 150 K resulted in its immediate disintegration, prompting a detailed variable‐temperature crystallographic study (Figure 4 and Table S4). When the crystal is cooled slowly from 270 to 90 K (ca. 60 K per hour), it preserves its shape and reveals two distinct reversible single‐crystal‐to‐single‐crystal (SCSC) transformations confirmed by cyclic SC‐ and PXRD studies (Figures 4 and S21). The first transition from the monoclinic space group *I*2/*a* to *P*2_1_/*c* occurs at 215–220 K, as revealed by DSC analysis (Figure S22). The second transformation from the monoclinic (*P*2_1_/*c*) to a higher symmetry orthorhombic (*Pnma*) phase, which is accompanied by an overall 4% volume contraction and significant change of the *b* (12.66 to 12.28 Å) and *β* (97.3 to 90.0°) parameters, takes place upon further cooling to 90 K. When the sample is subjected to a rapid freezing from 298 to below 180 K, the phase transition *I*2/*a*→ *Pnma* occurs instantly. Although the sandwich‐like (I^−^‐**1^+^**)–(I^−^‐**1^+^**) columnar topology is retained, the unit cell transformations involve a subtle but collective molecular rearrangement, which includes a drift of the iodide counterion of total ca. 0.2 Å along the pyridinium plane and *b*‐axis and a hapticity change from *η* ^2^(C_1_N_1_) to *η* ^1^(N_1_). This results in nearly perpendicular I—‐N^+^(pyridinium) connectivity (see insets of Figure 4), and an appreciable contraction of the anion–π^+^ distances (Figure 4). Such thermally activated space‐group interconversions based on counterion migration and hapticity switching are, to the best of our knowledge, unprecedented in organic ion‐pair crystals. While SCSC transitions are known in metal–organic frameworks, spin‐crossover complexes, and flexible coordination polymers, they rarely occur in rigid, densely packed ionic solids [48]. The reversible anion relocation observed in **1**, therefore, represents a unique form of thermally responsive elasticity and structural adaptability, establishing this material as a rare benchmark for SCSC behavior in purely organic anion–cation assemblies. VT measurements were also performed for samples **2–4** and revealed a 1%–3% cell contraction and anion–π^+^ distance shortening by 0.007–0.066 Å upon cooling without alteration of the space groups (Figures S13–S15 and Tables S5–S7).

### 2.5 Solid State Luminescence

Consistent with the solution studies, photophysical behavior is governed by ion‐pair charge‐transfer (CT). The crystalline salts **1–4** exhibit intense yellow‐orange coloration (Figures 5 and S23–S26; Tables 1 and S11), associated with the strong CT character (iodide→π^*^) of a low‐energy broad excitation band (*λ*_exc_ = 470–485 nm), which is considerably red‐shifted relative to the monosubstituted *o,m,p*‐**pyI** systems (*λ*_exc_ = 380–430 nm). The frontier molecular orbitals, together with the TDDFT‐calculated excitation electron‐density difference plots (Figures 5 and Tables S12–S18), consistently support the assignment of the lowest excited state to an iodide→π charge‐transfer configuration. In particular, the excitation density difference plots directly show electron‐density depletion on the iodide anion and accumulation on the pyridinium π‐system upon absorption/excitation.

**FIGURE 5:**
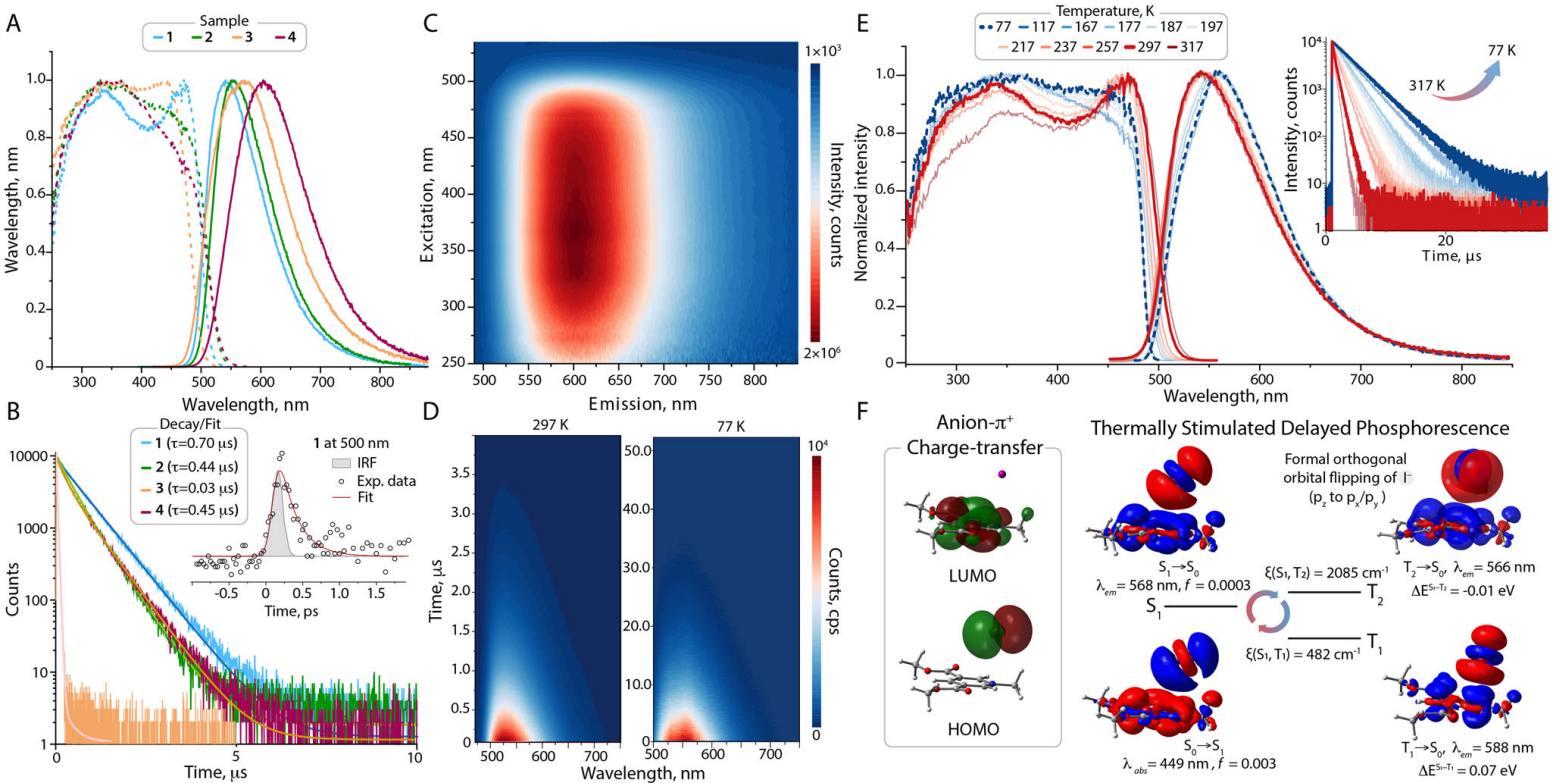
(A) Normalized solid state emission and excitation spectra of **1–4** at 297 K. (B) Time‐resolved emission decays of **1–4** and their fits (inset shows early relaxation dynamics of **1** at 500 nm obtained by femtosecond emission up‐conversion). (C) Excitation–emission map for **1** at 297 K. (D) Time‐dependent emission spectra of **1** measured at 297 and 77 K. (E) Variable temperature excitation, emission and time‐resolved emission decays for **1**. (F) HOMO/LUMO plots for **1**; excitation (S_0_→S_1_), singlet (S_1_→S_0_) and triplet emission (T_2_/T_1_→S_0_) electron density difference plots for **1** (loss electron density: blue, gain: red).

The crystalline materials *m*‐ and *p*‐**pyI** are non‐emissive at room temperature, given the relatively long anion–π^+^ separations (≥3.70 Å), which limit effective electronic coupling and decrease the rate of heavy‐atom‐induced ISC. At 77 K, the suppression of non‐radiative decay channels enables the photoemission in *m*‐**pyI**, observed as a broad CT band peaking at 520 nm with an average lifetime of 0.31 µs, whereas *p*‐**pyI** remains non‐emissive (Figures S25 and S26; Tables 1 and S11). Salt *o*‐**pyI** exhibits a distinct behavior as a more favorable ion‐pair arrangement leads to detectable, although weak, room‐temperature luminescence. This emission, with a maximum at 593 nm and a short excited‐state lifetime of 25 ns (Q.Y. < 0.01) indicates that non‐radiative decay processes still dominate under ambient conditions. Upon cooling to 77 K, the lifetime increases substantially to 0.50 µs without a significant shift in emission energy, which is consistent with the relaxation of the triplet excited state, that is, phosphorescence [25, 27, 28, 31, 37].

**TABLE 1:** Photophysical data of the solids *m*‐ and *o*‐**pyI** and **1–6** at 297 and 77 K.

	*λ*_exc_ (nm)	*λ*_em_ (nm)		*τ*_av_ (µs)^a^		Q.Y.	*k*_r_ (10^5^ s^−1^)^b^	*k*_nr_ (10^6^ s^−1^)^b^
	297 K	297 K	77 K	297 K	77 K	297 K	297 K	297 K
*m*‐**pyI**	380	n.e.	520	n.d.	0.31	n.d.	—	—
*o*‐**pyI**	430	593	590	0.007^*^	0.50	<0.01	—	—
**1**	475	540	560	0.70	3.56	0.22	3.1	1.1
**2**	480	555	565	0.44	2.70	0.23	5.2	1.8
**3**	470	575	540	0.025^*^	6.23	0.01	4.0	39.6
**4**	485	605	612	0.45	2.69	0.11	2.4	2.0
**^–/+^5**	420	565	577	0.027^*^	4.14	0.01	3.3	36.7
**6**	465	570	572	n.d.	2.89	<0.01	—	—

^a^ The average amplitude‐weighted emission lifetime for multiexponential decays (*τ*_av_ = Σ*A*_i_ *τ*_i_, where *A*_i_—the weight of the exponent) measured at *λ*_em_ maxima in MCS or ^*^TCSPC (*o*‐**pyI**, **3** and –**^/+^5** at 297 K) modes.

^b^ *k*_r_ = Q.Y./*τ*_av_, *k*_nr_ = (1‐Q.Y.)/*τ*_av_. n.e.—non‐emissive; n.d.—not determined due to low emission intensity.

Tighter ion pairing in crystalline **1–4** is manifested by their dramatically enhanced photoluminescence at 297 K (Figures 5A and S27–34; Tables 1 and S11). All species display broad CT emission bands spanning from green–yellow to orange and maximized at 540 nm (**1**), 555 nm (**2**), 575 nm (**3**), and 605 nm (**4**). This systematic bathochromic shift reflects the gradual narrowing of the CT bandgap across the series and follows the progressive drift of the iodide anion along the pyridinium motif. The accompanying increase in the anion–π^+^ distance emulates a donor–acceptor (D–A) architecture with spatially separated D and A groups that leads to the stabilization of the CT excited state [42, 43].

Salts **1** and **2**, which feature the shortest anion–cation contacts, exhibit the highest quantum yields (Q.Y. = 0.22 and 0.23, respectively), whereas **3** and **4** show reduced efficiencies (Q.Y. = 0.01 and 0.11), indicating an increased contribution from non‐radiative decays. Time‐resolved emission measurements reveal sub‐microsecond lifetimes (*τ*_av_ = 0.70, 0.44, and 0.45 µs for **1**, **2**, and **4**, respectively), corresponding to exceptionally high radiative rate constants (*k*_r_ = 2.4–5.2×10^5^ s^−1^), along with moderate non‐radiative rates (*k*_nr_ = 1.1–2.0×10^6^ s^−1^). In contrast, **3** shows a nearly two‐order‐of‐magnitude increase of *k*_nr_ reaching 0.4×10^8^ s^−1^, which accounts for its low emission efficiency. This pronounced quenching is likely associated with a more flexible cation‐anion organization facilitating enhanced vibrational relaxation and energy dissipation, which possibly arises from conformationally mobile alkyl chains and the absence of the rigid columnar packing found in its intensely emissive analogues. In addition, ^+/–^ **5** and **6** exhibit poor CT luminescence at 297 K (Figures S24 and S34; Table S11), presumably as a result of the presence of excessive hydrogen bonding and high non‐radiative rates introduced by menthol groups. No detectable chiral emission of enantiopure ^+^ **5** and –**5** was observed (Figure S35).

For **1–4**, the *k*_r_s are determined to lie in the range of 10^5–^10^6^ s^−1^, unequivocally indicating a phosphorescent origin. Notably, the corresponding microsecond‐scale radiative lifetimes rank among the shortest reported for metal‐free and/or heavy‐halogen‐substituted organic phosphors. These findings imply pronounced spin–orbit coupling (SOC) between the singlet and triplet manifolds in **1–4**, rendering the T_1_→S_0_ transition partially allowed (vide infra). The strong SOC is further expected to promote rapid ISC, consistent with the absence of detectable steady‐state fluorescence for **1–4**. Additional support is provided by femtosecond emission up‐conversion measurements of the solid films (Figure S24). Monitoring the emission at ∼500 nm, tentatively assigned to the fluorescence region, reveals an ultrafast decay component of 240 fs for **1** (inset, Figure 5B), indicative of highly efficient ISC. This observation is in good agreement with the computed ISC rate for **1** (*k*_ISC_ > 10^11^ s^−1^; see Supporting Information), implying that ISC overwhelmingly outcompetes the S_1_ → S_0_ radiative decay (typically on the nanoseconds timescale), thereby leading to near‐unity population of the triplet manifold.

Excitation–emission mapping (EEM) of **1–4** (Figure 5C; Figures S29, S31, and S33) reveals a single, broad emissive domain spanning a wide excitation range, consistent with emission originating from a ^3^CT excited state. This assignment is further corroborated by time‐resolved emission spectra of **1** at 297 and 77 K (Figure 5D), which show no discernible temporal spectral evolution, indicating the absence of significant conformational relaxation or excited‐state interconversion on the emission timescale. Theoretical analysis suggests that ISC in **1** and **2** proceeds preferentially from the S_1_ (CT) state to the second triplet state (T_2_) (Tables S12–S19, vide infra). This pathway is facilitated by the near‐degeneracy of S_1_ and T_2_ (Δ*E*(S_1–_T_2_) = −0.01 eV) and by an orthogonal reorientation (“flipping”) of p orbital of the iodide relative to the π‐system, which preserves angular momentum. Consequently, a large SOC matrix element, ξ(S_1_, T_2_), of up to 2085 cm^−^^1^ is predicted (vide infra). Subsequent rapid internal conversion from T_2_ to T_1_ efficiently populates the lowest triplet state, from which phosphorescence occurs. This cascade S_1_(CT) → T_2_ → T_1_ thus constitutes an efficient pathway to the lowest triplet state and underpins high phosphorescence rates observed in these ion‐paired systems.

Further insight into the emission behavior is provided by variable‐temperature steady‐state and time‐resolved measurements (Figure 5E; Tables S11 and S27–S34). Lifetimes monitored at different wavelengths are identical and increase linearly upon cooling, reaching 3.56 µs for **1** and 2.70 µs for **2** at 77 K. This trend reflects suppression of non‐radiative decay pathways and is consistent with the emission originating from the triplet state. Upon cooling, the emission maxima of **1** and **2** undergo modest bathochromic shifts (ca. 320–650 cm^−1^) while retaining nearly unchanged full widths at half maximum (FWHM), indicating minimal structural reorganization between the emissive states at 297 and 77 K. Indeed, the calculated energy separations between the two lowest CT triplet states (T_1_ and T_2_) for **1** and **2** are only 0.08–0.11 eV, comparable to thermal energy at ambient conditions. As a result, thermally activated repopulation of T_2_ from T_1_ is feasible, and both states, in equilibrium, contribute to room‐temperature emission. Within this framework, the high‐energy shoulders observed at 77 K can be reasonably attributed to a reduced population of the higher‐lying T_2_ state, leading to a diminished contribution from T_2_ → S_0_ emission. Consistently, the decreased T_2_ population also accounts for the small bathochromic shifts observed experimentally at 77 K. The possibility of dual phosphorescence arising from two energetically distinct excited states associated with an asymmetric I^−^–**1** ^+^/**2** ^+^–I^−^ anion–π^+^ arrangement can also be considered. However, distinguishing between these pathways is experimentally infeasible due to their very similar relaxation timescales and energetic profiles, as indicated by TDDFT calculations (see Table S12 versus Table S13).

In contrast, **3** and **4** display purely rigidochromic behavior under cryogenic conditions (Table S11) [31], indicating that their emission arises exclusively from the lowest triplet state (T_1_). The relatively large energy gaps to higher‐lying triplet states (Δ*E*(T_1–_T_2_) = 0.20–0.22 eV) preclude thermally activated repopulation of T_2_, such that the luminescence is governed solely by structural relaxation on a single T_1_ excited‐state surface. The thermally induced blue shift observed for **3** (575/540 nm at 297/77 K), together with its ca. 20‐fold higher non‐radiative rate relative to **4**, points to a lower degree of rigidity in the crystals of **3**. This, in turn, implies a more localized and less stabilized CT state, consistent with reduced iodide–pyridinium electronic coupling.

The origin of the exceptionally fast phosphorescence observed in **1–4** is of fundamental importance. Theoretical calculations reveal that the lowest triplet state (T_1_) is not electronically isolated, but instead strongly coupled to higher‐lying singlet excited states (S_1–_S_3_) through substantial spin–orbit coupling (Tables S20–S23). Specifically, SOC matrix elements between T_1_/T_2_ and higher singlet states (notably S_2_ and S_3_) are calculated to lie in the range of 113–2133 cm^−^^1^, while these singlet states retain appreciable oscillator strengths for radiative decay to the ground state (for S_1–_S_3_ → S_0_ *f* is up to 0.1574). Under these conditions, the formally spin‐forbidden T_1_/T_2_ → S_0_ transitions can effectively ‘borrow’ intensity from allowed singlet transitions, leading to a pronounced enhancement of the phosphorescence rate [49]. This mechanism is further reinforced by the CT character of the excited states and the strong perturbation imposed by the heavy iodide counterion. Furthermore, reorientation of the p orbital of the iodide involved in the n–π* transition, specifically from p_z_ in S_1_ to p_x_/p_y_ in S_1_ and T_2_, also facilitates spin flipping, as the required angular momentum change is compensated by this n–π* orbital reconfiguration. This effect contributes to the ultrafast ISC, exemplified by the 240 fs time constant observed for **1** (inset, Figure 5B, vide supra).

Phosphorescence radiative rate constants on the order of 10^5–^10^6^ s^−1^ are exceedingly rare among purely organic systems. In particular, the *k*_r_ value of 5.2 × 10^5^ s^−1^ for **2** ranks among the fastest reported for metal‐free triplet emitters. It outperforms the benchmark organic phosphors such as hexakis(arylthio)benzenes [50], which exhibit *k*_r_ values on the order of 3.3×10^5^ s^−1^ in the solid state. Importantly, fast phosphorescence is a critical yet rarely optimized parameter in metal‐free organic emitters, since efficient photoemission from the triplet state has conventionally relied on phosphorescent complexes of heavy transition metals such as iridium and platinum. Developing molecular design strategies that enable exceptionally rapid triplet radiative decay using earth‐abundant elements is therefore highly desirable, as it minimizes triplet accumulation and competing non‐radiative losses while providing a sustainable platform for next‐generation optoelectronic devices, X‐ray scintillators, and other technologies requiring efficient and rapid photon conversion [51]. In this context, the pyridinium iodide systems **1–4** showcase successful intensity borrowing in anion→π CT states, providing a compelling design principle for achieving exceptionally fast phosphorescence without relying on covalently bound heavy metal centers but rather on the main‐group counterion. The role of the iodide in the population of the triplet state and its relaxation is highlighted by **1** ^OTf^/**1** ^BARF^ and **1** ^Br^, where the tight ion‐paired geometry is retained but luminescence is absent (**1** ^OTf^/**1** ^BARF^), or very weak CT phosphorescence is observed (**1** ^Br^; *λ*_em_ = 530 nm, Φ < 0.01, Figure S26 and Table S11).

### 2.6 X‐Ray Scintillation

Compounds **1**, **2**, and **4** exhibited detectable radioluminescence under X‐ray irradiation, consistent with their efficient charge‐transfer phosphorescence (Figure S36). Sample **1** was selected for proof‐of‐concept evaluation of its X‐ray scintillation performance (Figure 6). The mass attenuation coefficient and attenuation efficiency of **1** are comparable to those of the CsI:Tl reference in the low‐energy X‐ray region (Figure 6A,B). The estimated light yield of **1** reaches 8900 photons/MeV, compared to 54000 photons/MeV for CsI:Tl. Importantly, the RL intensity shows a linear dependence on X‐ray dose rate over the investigated range (*R* ^2^ = 0.997; Figure 6C), indicating a reliable dose‐dependent response. X‐ray imaging using a scintillation screen based on crystalline **1** demonstrated the limit of detection of 2.1 nGy/s and a spatial resolution of 9.9 lp/mm at MTF = 0.2, as determined by the slanted‐edge method (Figure 6D). The material's practical imaging capability was further demonstrated by the clear visualization of metallic and plastic components in an integrated circuit chip (Figure 6D, inset). These results highlight the potential of organized anion–π^+^ phosphors as metal‐free scintillation materials.

**FIGURE 6:**
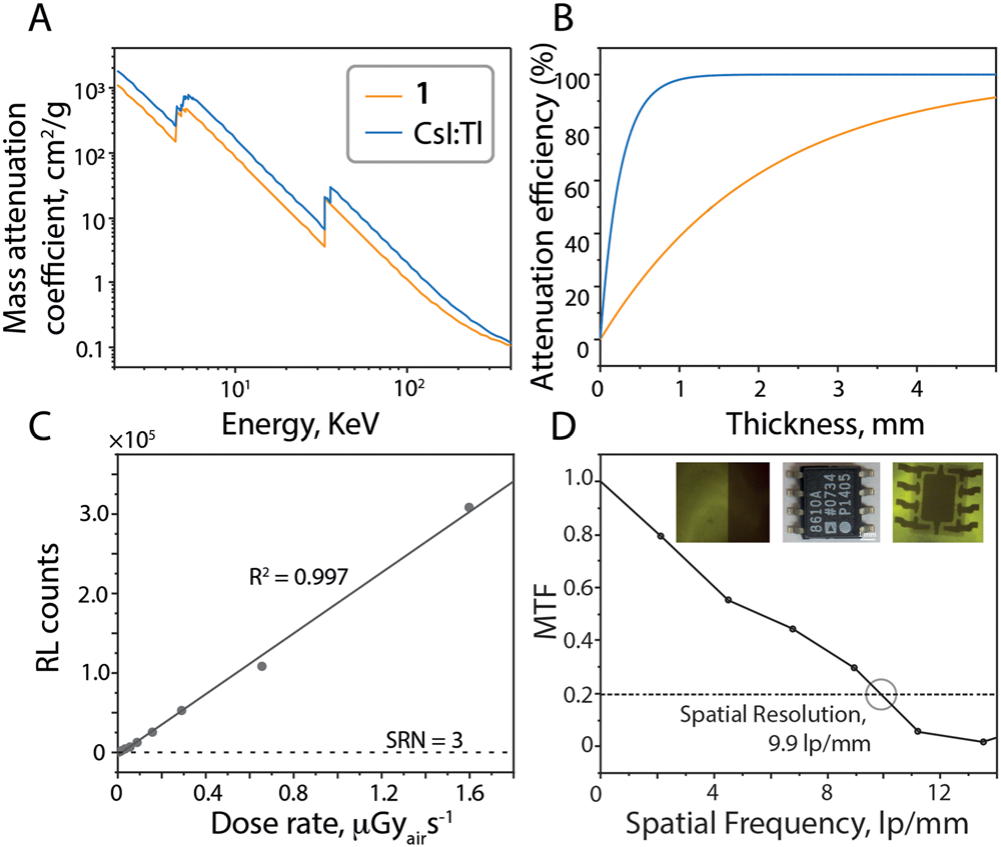
X‐ray scintillation performance. (A) The plot of absorption coefficients as functions of photon energy for solid **1** and CsI:Tl standard. (B) Thickness‐dependent attenuation efficiency of solid **1** and CsI:Tl at 30 keV. (C) The linear relationship between RL intensity and the dose rate. (D) Spatial resolution obtained by slanted‐edge method (insets: the photograph of the film under ambient light and X‐rays; application of the thin film **1** in a safety check: X‐ray image of the chip).

## 3 Conclusion

In this work, we have demonstrated that deliberate orchestration of anion–π^+^ interactions in pyridinium‐based contact ion pairs enables unprecedented acceleration of room‐temperature phosphorescence in metal‐free organic systems. While one carboxylate group enables observation of nearly ‘dark’ non‐emissive CT states, the combination of two electron‐withdrawing substituents with further steric control, enforces short‐distance and tunable anion–π^+^ contacts that stabilize charge‐transfer excited states and enhance spin–orbit coupling. This results in efficient intersystem crossing and exceptionally fast phosphorescence with radiative rates, up to 5.2×10^5^ s^−1^, owing to (i) the heavy atom effect of surrounding iodides and (ii) the change of orientation of the p orbital of the iodide involved in the n–π^*^ electronic transition. Structural insights demonstrate, for the first time, the mobility of anion–π^+^ interactions. The dynamics is associated with the iodide drift and hapticity change from *η* ^2^(C_1_N_1_) at 297 K to *η* ^1^(N_1_) at 100 K, realized via a two‐stepwise single‐crystal‐to‐single‐crystal reversible transformation. Variable‐temperature photophysical measurements, complemented by theoretical analysis, reveal that intensity borrowing within the charge‐transfer (CT) manifold underpins the interplay between phosphorescence and thermally activated delayed phosphorescence. Beyond providing fundamental insight, the demonstrated high limit of detection of 2.1 nGy/s and a spatial resolution of 9.9 lp/mm of scintillation performance underscores the practical promise of these materials. Collectively, these findings establish a unified design paradigm for high‐performance organic phosphors based on ion‐pair engineering and delineate new opportunities for sustainable optoelectronic and radiation‐detection technologies.

## Author Contributions

**Eetu Hakkarainen**: investigation, data curation, supervision, formal analysis, writing – review and editing, and visualization. **Corentin Montagne**: investigation, data curation, and formal analysis. **Hao‐Cheng Lin**: investigation, writing – review and editing, data curation, and methodology. **Chen‐Yu Lin**: writing – review and editing, investigation, data curation, and methodology. **Khai‐Nghi Truong**: investigation, resources, and writing – review and editing. **Po‐Yu Chen**: resources. **Anton Nechaev**: investigation. **Fabien B. L. Cougnon**: funding acquisition, writing – review and editing, and supervision. **Igor O. Koshevoy**: funding acquisition, writing – review and editing, and supervision. **Toni Eskelinen**: methodology, investigation, writing – review and editing, and software. **Hao‐Wu Lin**: funding acquisition, resources, supervision, and writing – review and editing. **Pi‐Tai Chou**: funding acquisition, writing – review and editing, resources, supervision, and visualization. **Andrey Belyaev**: conceptualization, investigation, writing – original draft, writing – review and editing, visualization, methodology, data curation, supervision, project administration, and validation.

## Conflicts of Interest

The authors declare no conflicts of interest.

## Supporting information



The authors have cited additional references within the Supporting Information [40, 41, 52, 53, 54, 55, 56, 57, 58, 59, 60, 61, 62, 63, 64, 65, 66, 67].**Supporting File**: anie74485‐sup‐0001‐SuppMat.pdf.

## Data Availability

The data that supports the findings of this study are available in the Supporting Information of this article.
