# Supplementary material for: Orchestrating Anion–π+ Interactions to Accelerate Metal‐Free Room‐Temperature Phosphorescence
Source: Angew Chem Int Ed Engl. 2026 Aug 28;65(43):e4386683. doi: 10.1002/anie.4386683 (PMC13676399; doi:10.1002/anie.4386683)
Supplement: Supplementary file 1 [file ANIE-65-e4386683-s001.pdf]

Supporting information for

Orchestrating Anion-π^+ Interactions to Accelerate Metal-Free Room-Temperature Phosphorescence

Eetu Hakkarainen,[a] Corentin Montagne,[a] Hao-Cheng Lin,[b] Chen-Yu Lin,[c] Khai-Nghi Truong,[d] Po-Yu Chen,[e] Anton Nechaev,[f] Fabien Cougnon,[f] Igor O. Koshevoy,[a] Toni Eskelinen,[g,h] Hao-Wu Lin,[b] Pi-Tai Chou,[c]* Andrey Belyaev [a]*

[a] Department of Chemistry, University of Eastern Finland, Yliopistokatu 7, Joensuu, 80101, Finland

[b] Department of Materials Science and Engineering, National Tsing-Hua University, 101, Sec. 2, Kuang-Fu Road, Hsinchu 30013, Taiwan, Republic of China.

[c] Department of Chemistry, National Taiwan University, Taipei, 10617 Taiwan, Republic of China

[d] Rigaku Europe SE, Hugenottenallee 167, 63263 Neu-Isenburg, Germany

[e] Advanced Packaging Instrumentation and Metrology Laboratory, Industrial Technology Research Institute, Hsinchu 30013, Taiwan, Republic of China

[f] Department of Chemistry, University of Jyväskylä, Survontie 9 B, 40500 Jyväskylä, Finland

[g] Department of Chemistry and Materials Science, Aalto University, FI-00076 Aalto, Finland

[h] Institute for Chemical Research, Kyoto University, Uji, Kyoto 611-0011, Japan

E-mail: andrei.beliaev@uef.fi; chop@ntu.edu.tw

Table of Contents

Contents

**Experimental Procedures / Characterization methods.**	**4**
**Figures S1–2**. DSC and TGA data of the studied salts **1**–**6**, $\mathbf{1}^{Br}$, $\mathbf{1}^{OTf}$, *o,m,p*-**pyl**.	**13**
**Figures S3–4**. HPLC chromatograms of **1**–**6** and $\mathbf{1}^{Br}$, $\mathbf{1}^{OTf}$, $\mathbf{6}^{Br}$, *m*-**pyl** and *p*-**pyl**.	**15**
**Figure S5.** Normalized absorption profiles of the *o,m,p*-**pyl** and **1**, $\mathbf{1}^{Br}$, $\mathbf{6}^{Br}$ in DCM.	**17**
**Figure S6**. Absorption profiles of the samples **1**-**6** at 10^{-4} M measured in DCM and in a variety of solvents for $^{-}$**5**.	**17**
**Figure S7**. (A) Calculated (DFT) and (B) experimental (in DCM, 10^{-4} M) absorption spectra of **1**, $\mathbf{1}^{X}$ (X = BARF, OTf) and cation $\mathbf{1}^{+}$.	**18**
**Table S1**. Calculated singlet excited states ($S_0 \rightarrow S_n$, n = 1–9) of **1** supplemented with energies, NTO weights, transition character, and representative hole/electron NTO.	**19**
**Figure S8.** Steady-state excitation and emission spectra of frozen DCM solutions of **1**–**4** and **6** measured at 77 K.	**20**
**Figure S9**. Time-resolved and fitted decay curves of frozen DCM solutions of **1**–**4** and **6** measured at 77 K.	**21**
**Figure S10**. Steady-state emission and excitation spectra of frozen solutions of $^{-}$**5** measured at 77 K.	**22**
**Table S2***.* Summary of the photophysical properties of frozen solutions of **1**–**6**.	**23**
**Figure S11**. Steady-state absorption, emission and excitation spectra of $^{-}$**5** measured at 297 K in aerobic conditions in toluene.	**24**
**Figure S12**. Experimental and calculated PXRD data of the **1**–**4**, $^{-}$**5, $\mathbf{1}^{OTf}$**.	**25**
**Tables S3–8**. Crystal data and structure refinement.	**26**
**Figures S13–15**. Molecular views of the **2**–**4** at variable temperatures.	**32**
**Figures S16–18**. Molecular views of the *m*-**pyl**, *p*-**pyl**, $^{-}$**5** and $\mathbf{1}^{Br}$, $\mathbf{1}^{OTf}$, $\mathbf{1}^{BARF}$ and $\mathbf{4}^{A}$ **and 4-I/I3.**	**33**
**Figure S19**. Calculated ESP plots for *o*-, *m*-, *p*-**pyl, 1** and **2.**	**35**
**Table S9**. Energy decomposition analysis (kcal/mol).	**36**
**Table S10**. Summary of Bader's Quantum Theory of Atoms in Molecules (QTAIM) descriptors.	**37**
**Figure S20**. QTAIM analysis of the anion–π^{+} interactions in selected **1**, I^{-}–$\mathbf{1}^{+}$–I^{-}, *o*-**pyl**, and I^{-}– *o*-$\mathbf{py}^{+}$–I^{-}.	**38**
**Figure S21**. Cyclic VT–PXRD measurements (cooling–heating) of the salt **1**.	**39**
**Figure S22**. Cyclic DSC measurements of the crystalline **1**.	**40**
**Figure S23**. Solid-state reflectance spectra of *p*-**pyl**, *o*-**pyl** and *m*-**pyl** measured at 297 K.	**41**
**Figure S24.** Solid-state absorption (synchronous scans), steady-state excitation, and emission spectra of crystalline samples **1–3**, $^{-}$**5** and **6** measured at 297 K; early relaxation decays and fits measured by fluorescence up-conversion technique.	**42**

Table S11. Summary of the photophysical properties of pyridinium salts (**1-4, 1^{Br}, $^{-}$5, 6^{Br}, 6**) in the solid state measured at VT. **43**

Figure S25. Steady state excitation, emission, and time-resolved emission spectra of crystalline samples *o***-pyI and** *m***-pyI** measured at 297 and/or 77 K. **44**

Figure S26. Steady state excitation, emission, and time-resolved emission spectra of crystalline samples **6^{Br}** measured at 77 K, and **6** and **1^{Br}** measured at 297 and 77 K. **45**

Tables S12–18. HOMO/LUMO levels; excitation, fluorescence and triplet emission; electron density difference plots for **1–4**. **46**

Table S19. Calculated oscillator strengths, transition energies, radiative rate constants of phosphorescence, energy differences between the first excited states, spin-orbit coupling matrix elements between singlet and triplet states and intersystem crossing rates (k_{ISC}) for **1**–**4** in Pyr^{+} --- I^{-} and I^{-} --- Pyr^{+} --- I^{-} configurations. **53**

Figures S27–34. VT steady-state (excitation and emission) and time-resolved emission studies of crystalline **1–4, $^{-}$5**. **54**

Figure S35. Solid-state CPL measurements of $^{-}$**5** and $^{+}$**5**. **59**

Table S20–23. Computed spin-orbit coupling matrix elements, energy differences between the lowest excited triplet state and 10 lowest lying singlet excited states, and oscillator strengths for the spin-allowed singlet-singlet transitions for **1–4**. **59**

Figure S36. Radioluminescence spectra of crystalline samples **1**, **2**, and **4**. **62**

Figures S37–**64**. Copies of ^{1}H and ^{13}C NMR spectra. **63**

References **77**

Experimental Procedures

General Comments

Isomers (1*R*,2*S*,5*R*)-2-Isopropyl-5-methylcyclo-hexyl 2-bromoacetate and (1*S*,2*R*,5*S*)-2-isopropyl-5-methylcyclo-hexyl 2-bromoacetate were synthesized according to a published method.[1] Other reagents were used without purification as received from suppliers. All reactions were carried out under an inert nitrogen atmosphere in Sealed tubes or Schlenk flasks. The solution ^{1}H and $^{13}C\{^{1}H\}$ NMR spectra were recorded on Bruker 500 MHz Avance. Microanalyses were carried out in the analytical laboratory of the University of Eastern Finland.

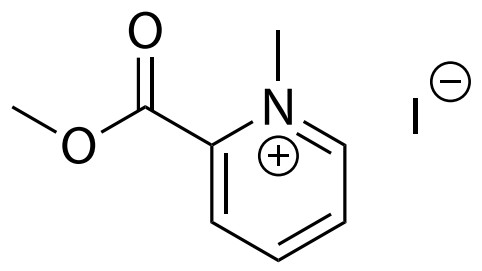

2-(methoxycarbonyl)-1-methylpyridin-1-ium iodide (*o*-**pyI**). Methyl picolinate (1.00 g, 7.30 mmol, 1.05 equiv.) was placed in a Schlenk tube and degassed. Acetonitrile (10 mL) and iodomethane (0.47 mL, 7.66 mmol, 1.05 equiv.) were then added under a nitrogen atmosphere, and the resulting mixture was heated at 60 °C to afford a homogeneous, transparent yellow solution. After 3 h, the solvent was removed *in vacuo*, and the resulting solid was washed with diethyl ether (6 × 20 mL). Recrystallization by gas-phase diffusion of diethyl ether into an acetonitrile solution of *o*-**pyI** afforded pale crystalline material, suitable for SCXRD analysis, in nearly quantitative yield.
^{1}H NMR (500 MHz, $CDCl_3$) δ 9.81 (d, *J* = 5.4 Hz, 1H), 8.85 (t, *J* = 7.8 Hz, 1H), 8.61 (d, *J* = 7.8 Hz, 1H), 8.46 (d, *J* = 5.4 Hz, 1H), 4.77 (s, 3H), 4.10 (s, 3H). $^{13}C\{^{1}H\}$ NMR (126 MHz, $CDCl_3$) δ 159.59, 150.47, 147.16, 141.99, 131.58, 130.56, 55.16, 50.29. Anal. Calcd for $C_8H_{10}INO_2$: C, 34.43; H, 3.61; N, 5.02. Found: C, 34.28; H, 3.59; N, 4.91.

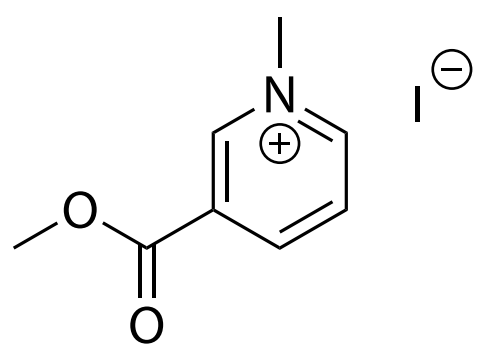

3-(methoxycarbonyl)-1-methylpyridin-1-ium iodide (*m*-**pyI**). Methyl nicotinate (1.00 g, 7.30 mmol, 1.05 equiv.) was placed in a Schlenk tube and degassed. Acetonitrile (10 mL) and iodomethane (0.47 mL, 7.66 mmol, 1.05 equiv.) were then added under a nitrogen atmosphere, and the resulting mixture was heated at 60 °C to afford a homogeneous, transparent yellow solution. After 3 h, the solvent was removed *in vacuo*, and the resulting solid was washed with diethyl ether (6 × 20 mL). Recrystallization by gas-phase diffusion of diethyl ether into a methanol solution of *m*-**pyI** afforded pale yellow polycrystalline material in nearly quantitative yield.
^{1}H NMR (500 MHz, $CDCl_3$) δ 9.78 (t, *J* = 6.1 Hz, 1H), 9.33 (s, 1H), 8.93 (dd, *J* = 7.1, 6.1 Hz, 1H), 8.29 (t, *J* = 7.1 Hz, 1H), 4.78 (s, 3H), 4.07 (s, 3H). $^{13}C\{^{1}H\}$ NMR (126 MHz, $CDCl_3$) δ 161.56, 149.25, 146.38, 145.28, 130.32, 128.86, 54.11, 50.60, 17.57. Anal. Calcd for $C_8H_{10}INO_2$: C, 34.43; H, 3.61; N, 5.02. Found: C, 34.33; H, 3.48; N, 4.99.

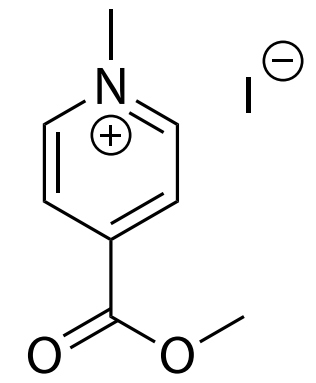

4-(methoxycarbonyl)-1-methylpyridin-1-ium iodide (*p*-**pyI**). Methyl isonicotinate (1.00 g, 7.30 mmol, 1.05 equiv.) was placed in a Schlenk tube and degassed. Acetonitrile (10 mL) and iodomethane (0.47 mL, 7.66 mmol, 1.05 equiv.) were then added under a nitrogen atmosphere, and the resulting mixture was heated at 60 °C to afford a homogeneous, transparent yellow solution. After 3 h, the reaction mixture was cooled to a –18 °C, and the orange solid was filtered and washed with diethyl ether (6 × 20 mL).

Recrystallization from acetonitrile solution (from hot to –18 °C) afforded *p*-**pyI** as an orange crystalline material in nearly quantitative yield.
^{1}H NMR (500 MHz, DMSO-d_6) δ 9.17 (d, *J* = 5.5 Hz, 2H), 8.50 (d, *J* = 5.5 Hz, 2H), 4.43 (s, 3H), 3.98 (s, 3H). ^{13}C{^{1}H} NMR (126 MHz, DMSO-d_6) δ 162.63, 147.05, 143.33, 126.77, 53.83, 48.48. Anal. Calcd for $C_8H_{10}INO_2$: C, 34.43; H, 3.61; N, 5.02. Found: C, 34.23; H, 3.45; N, 5.11.

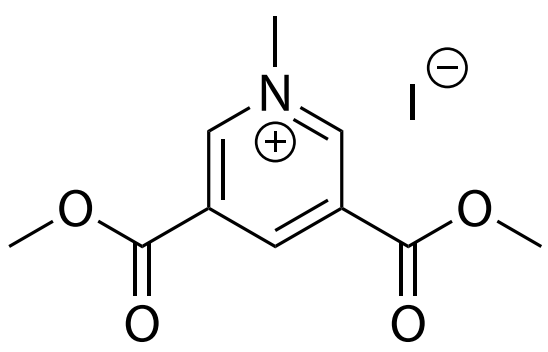

3,5-Bis(methoxycarbonyl)-1-methylpyridin-1-ium iodide (**1**). Dimethyl pyridine-3,5-dicarboxylate (1.47 g, 7.53 mmol, 1.00 equiv.) was dissolved in acetonitrile (10 mL) in a sealed tube and degassed. Iodomethane (0.70 mL, 11.30 mmol, 1.50 equiv.) was then added in a single portion. The mixture was sealed and heated at 50 °C to afford a homogeneous, transparent yellow solution. After stirring for 4 h, the reaction mixture was cooled down and poured into diethyl ether (100 mL), and the resulting precipitate was collected by filtration, washed with diethyl ether (6 × 20 mL), and dried under high vacuum to give a yellow solid (2.39 g, 94% yield). Recrystallization by gas-phase diffusion of diethyl ether into an acetonitrile or methanol solution of **1** at room temperature afforded a yellow crystalline material suitable for scXRD analysis.
^{1}H NMR (500 MHz, DMSO-d_6) δ 9.78 (s, 2H), 9.10 (s, 1H), 4.50 (s, 3H), 4.02 (s, 6H). ^{13}C{^{1}H} NMR (126 MHz, DMSO-d_6) δ 161.61, 149.87, 143.73, 129.37, 53.80, 48.66. Anal. Calcd for $C_{10}H_{12}INO_4$: C, 35.63; H, 3.59; N, 4.15. Found: C, 35.46; H, 3.59; N, 4.08.
NB! We additionally attempted a more environmentally benign synthesis of the salts **1** and **2** via a two-step alkylation/metathesis route employing dimethyl/diethyl carbonate and KI, which resulted in either a modest overall yield of 13% for **1**, or as little as 1–2% conversion for **2**. Such low efficiency is likely due to the strongly deactivated nature of the dicarboxyester–pyridine core, especially when compared to the high yields (up to 97%) reported for more nucleophilic systems like dimethylaminopyridine. While this green approach demonstrates conceptual potential, its practical utility remains limited for deactivated pyridines under the required harsh conditions (MeOH, 140 °C, 60h) and poor conversion.

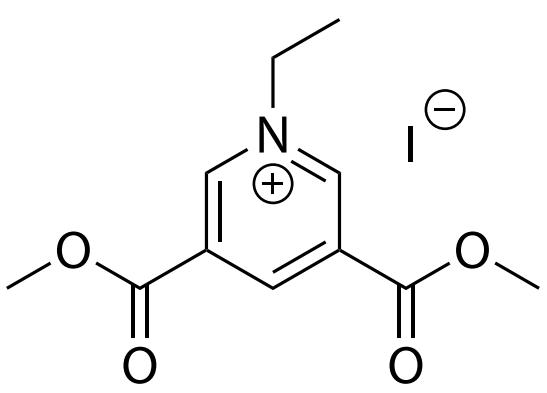

3,5-Bis(methoxycarbonyl)-1-ethylpyridin-1-ium iodide (**2**). Obtained similarly to **1** from dimethyl pyridine-3,5-dicarboxylate (1.00 g, 5.13 mmol, 1.00 equiv.) and iodoethane (0.62 mL, 7.69 mmol, 1.50 equiv.). Recrystallization by a gas phase diffusion of diethyl ether into a methanol solution of **2** gave orange crystals suitable for scXRD analysis (1.60 g, 89%).
^{1}H NMR (500 MHz, DMSO-D_6) δ 9.82 (s, 2H), 9.08 (s, 1H), 4.79 (q, *J* = 7.3 Hz, 2H), 3.98 (s, 6H), 1.53 (t, *J* = 7.3 Hz, 3H).^{13}C{^{1}H} NMR (126 MHz, DMSO-D_6) δ 162.18, 149.45, 144.39, 130.64, 58.03, 54.31, 17.03. Anal. Calcd for $C_{11}H_{14}INO_4$: C, 37.63; H, 4.02; N, 3.99. Found: C, 37.59; H, 4.03; N, 3.95.

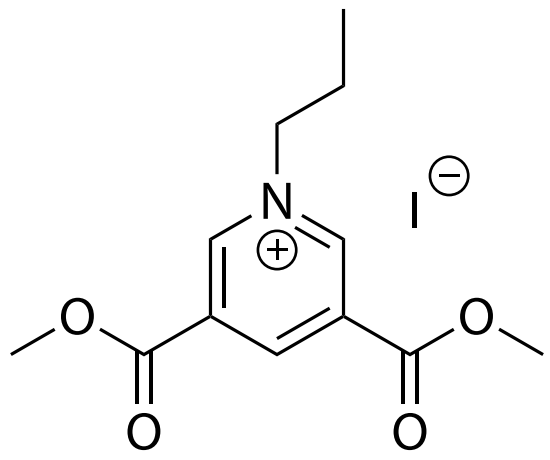

3,5-Bis(methoxycarbonyl)-1-propylpyridin-1-ium iodide (3). Obtained similarly to **1** from dimethyl pyridine-3,5-dicarboxylate (1.00 g, 5.13 mmol, 1.00 equiv.) and iodopropane (1.00 mL, 10.26 mmol, 2.00 equiv.) at 60 °C and 6 h of heating. Recrystallization by a gas phase diffusion of diethyl ether into a methanol solution of **3** gave yellow crystals suitable for scXRD analysis (1.60 g, 54%).
^{1}H NMR (500 MHz, $CDCl_3$) δ 9.88 (s, 2H), 9.34 (s, 1H), 5.16 (t, *J* = 7.5 Hz, 2H), 4.07 (s, 6H), 2.17 (tq, *J* = 7.5, 7.0 Hz, 2H), 1.08 (t, *J* = 7.0 Hz, 3H). $^{13}C\{^1H\}$ NMR (126 MHz, $CDCl_3$) δ 161.03, 148.67, 145.33, 130.85, 64.84, 54.27, 24.89, 10.63. Anal. Calcd for $C_{12}H_{16}INO_4$: C, 39.47; H, 4.42; N, 3.84. Found: C, 39.74; H, 4.35; N, 3.74.

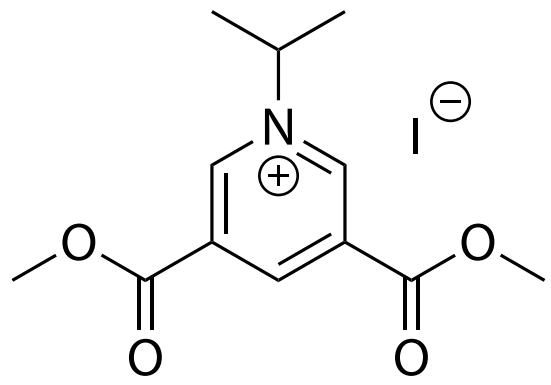

3,5-Bis(methoxycarbonyl)-1-isopropylpyridin-1-ium iodide (4). Obtained similarly to **1** from dimethyl pyridine-3,5-dicarboxylate (1.00 g, 5.13 mmol, 1.00 equiv.) and iodopropane (1.00 mL, 12.82 mmol, 2.50 equiv.) at 60 °C and 6 h of heating. Recrystallization by a gas phase diffusion of diethyl ether into a methanol solution of **4** gave pale orange crystals, suitable for scXRD analysis (1.60 g, 21%). Crystallization from acetonitrile allowed to obtain of an acetonitrile solvate $\mathbf{4^A}$ non-emissive polymorph with an admixture of the non-solvated form **4**. NB: Solution of **4** that left under aerobic conditions within 2 weeks delivers brown crystals composed of $\mathbf{4^+}$ with 1:1 mixed I^- and I_3^- counterions (see scXRD details in Table S7, entry **4(I3)**, Figure S16).
^{1}H NMR (500 MHz, $CDCl_3$) δ 9.86 (s, 2H), 9.39 (s, 1H), 6.01 – 5.85 (oc, J= 5.5 Hz, 1H), 4.11 (s, 6H), 1.87 (d, *J* = 5.5 Hz, 6H). $^{13}C\{^1H\}$ NMR (126 MHz, $CDCl_3$) δ 161.03, 146.79, 145.42, 131.38, 66.97, 54.31, 23.34. Anal. Calcd for $C_{12}H_{16}INO_4$: C, 39.47; H, 4.42; N, 3.84. Found: C, 39.55; H, 4.29; N, 3.96.
NB! Interestingly, the syntheses of **3** and **4** at elevated temperatures (from 60 to 70 °C) do not enhance the yields of desired compounds but rather lead to the appearance of mixtures of propyl re-esterification products, which are practically impossible to separate.

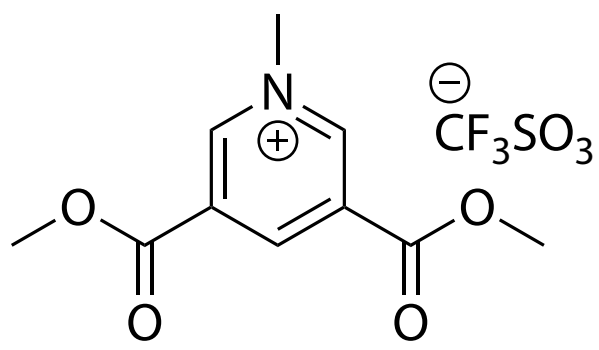

3,5-Bis(methoxycarbonyl)-1-methylpyridin-1-ium trifluoromethanesulfonate ($\mathbf{1^{OTf}}$). Dimethyl pyridine-3,5-dicarboxylate (1.00 g, 5.12 mmol, 1.00 equiv.) was dissolved in dichloromethane (15 mL), and methyl triflate (0.56 mL, 5.12 mmol, 1.00 equiv.) was added dropwise at –80 °C (acetone/liquid nitrogen bath). The mixture was stirred for 45 min and then allowed to warm to room temperature. The resulting precipitate was collected by centrifugation, washed with diethyl ether (3 × 15 mL), and dried under high vacuum to give a white crystalline solid (1.64 g, 89% yield). Crystals suitable for SCXRD analysis were obtained by gas-phase diffusion of diethyl ether into a methanol solution of $\mathbf{1^{OTf}}$.
^{1}H NMR (500 MHz, DMSO-D_6) δ 9.73 (s, 2H), 9.08 (s, 1H), 4.46 (s, 3H), 3.98 (s, 6H). $^{13}C\{^1H\}$ NMR (126 MHz, DMSO-D_6) δ 162.14, 150.41, 144.26, 129.94, 54.31, 49.14. Anal. Calcd for $C_{11}H_{12}F_3NO_7S$: C, 36.77; H, 3.37; N, 3.90; S, 8.92. Found: C, 36.70; H, 3.47; N, 3.86; S, 8.92.

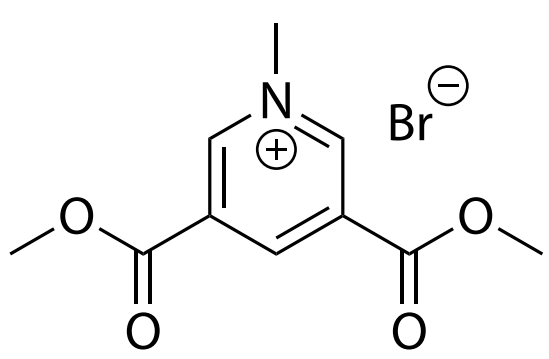

3,5-Bis(methoxycarbonyl)-1-methylpyridin-1-ium bromide (1^{Br}). **1**^{OTf} (0.15 g, 0.41 mmol, 1.00 equiv.) was added to a suspension of KBr in MeOH (1g in 5 ml). The mixture was stirred for 30 min, evaporated, and the precipitate was extracted with dichloromethane (15 ml). Solvent was evaporated on a rotovap, and the procedure was repeated for 3 more times with a new KBr/MeOH suspension. The solvent was removed under vacuum to obtain the white product (0.12 g, quantitative yield). Crystals suitable for SCXRD analysis were obtained by gas-phase diffusion of diethyl ether into a dichloromethane solution of **1**^{Br}. ^{1}H NMR (500 MHz, $CDCl_3$) δ 9.81 (s, 2H), 9.33 (s, 1H), 4.90 (s, 3H), 4.06 (s, 6H). ^{13}C{^{1}H} NMR (126 MHz, $CDCl_3$) δ 161.08, 149.83, 144.91, 130.55, 54.16, 50.53. Anal. Calcd for $C_{10}H_{12}NO_4Br$: C, 41.40; H, 4.17; N, 4.83. Found: C, 41.38; H, 4.30; N, 4.87.

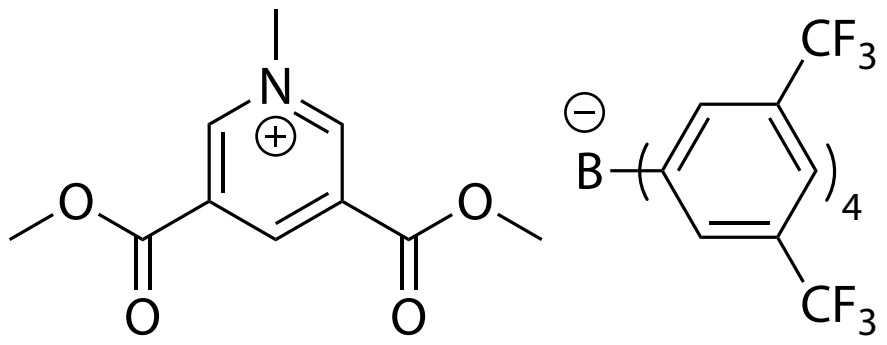

3,5-Bis(methoxycarbonyl)-1-methylpyridin-1-ium tetrakis(3,5-bis(trifluoromethyl)phenyl) borate (1^{BARF}). Sodium tetrakis(3,5-bis(trifluoromethyl)phenyl) borate (0.54 g, 0.61 mmol, 1.03 eq.) in 15 ml of DCM was added to a solution of **1** (0.20 g, 0.59 mmol, 1 eq.) in DCM (20 ml) to afford a clear yellow solution. The solution was stirred for 0.5 h until the color disappeared. The resulting clear colorless solution was filtered and evaporated on a rotovap to afford **1**^{BARF} as an off-white powder. Recrystallization by a slow evaporation of a 2:1 DCM : n-hexane solution of **1**^{BARF} gave clear, colorless crystals suitable for SCXRD analysis (0.50 g, 80 % yield). ^{1}H NMR (500 MHz, CD_2Cl_2) δ 9.55 (s, 1H), 9.25 (s, 2H), 7.71 (s, 8H), 7.55 (s, 4H), 4.52 (s, 3H), 4.08 (s, 6H). ^{13}C{^{1}H} NMR (126 MHz, CD_2Cl_2) δ 162.2 (q, *J* = 50.1 Hz), 160.2, 148.3, 146.9, 135.2, 133.1, 129.3 (qq, *J* = 31.6 Hz, 2.8 Hz), 125.0 (q, *J* = 272.4 Hz), 117.9 (m), 55.1, 50.5.

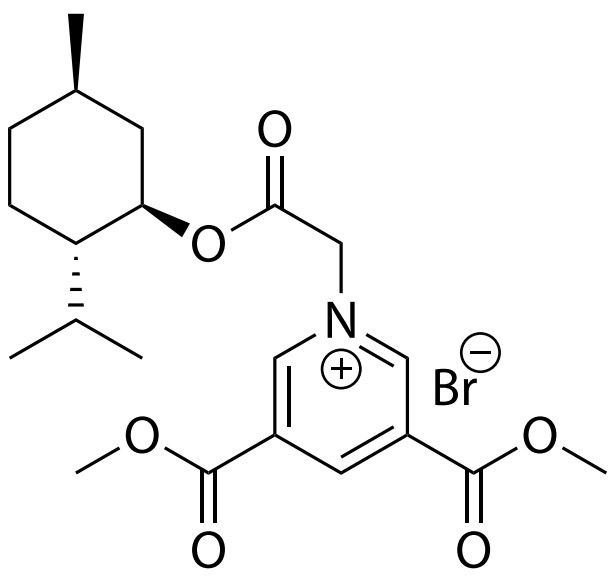

6^{Br}**.** Dimethyl pyridine-3,5-dicarboxylate (0.52 g, 2.67 mmol, 1.00 equiv.), (1*R*,2*S*,5*R*)-2-isopropyl-5-methylcyclohexyl 2-bromoacetate (1.48 g, 5.33 mmol, 2.00 equiv.), and acetonitrile (5 ml) were placed in a sealed tube (35 ml) and degassed. The reaction mixture was heated at 75 °C, affording a homogeneous, transparent yellow solution within 30 min. After 4 h, the reaction mixture was cooled, and diethyl ether (50 ml) was added. The resulting precipitate was collected by filtration, washed with diethyl ether (6 × 20 ml), and dried under vacuum to yield a white solid. Recrystallization by gas-phase diffusion of diethyl ether into a dichloromethane solution of **6**^{Br} afforded pale crystalline material (0.84 g, 67% yield).
^{1}H NMR (500 MHz, $CDCl_3$) δ 9.82 (s, 2H), 9.47 (s, 1H), 6.41 (d, *J* = 17.5 Hz, 1H), 6.22 (d, *J* = 17.5 Hz, 1H), 4.82 (td, *J* = 11.0, 7.8 Hz, 1H), 4.10 (d, *J* = 2.0 Hz, 6H), 2.04 (d, *J* = 12.0 Hz, 1H), 1.89 – 1.83 (m, 1H), 1.71 (t, *J* = 13.1 Hz, 2H), 1.50 – 1.42 (m, 2H), 1.20 (dd, *J* = 7.1, 2.0 Hz, 1H), 1.07 (dq, *J* = 38.2, 11.9 Hz, 2H), 0.92 (dd, *J* = 6.8, 2.1 Hz, 6H), 0.75 (dd, *J* = 7.0, 2.0 Hz, 3H). ^{13}C{^{1}H} NMR (126 MHz, $CDCl_3$) δ 165.30, 160.96, 150.71, 146.12, 130.01, 78.84, 65.93, 62.25, 54.21, 46.79, 40.66, 33.95, 31.57, 26.07, 23.13, 21.96, 20.93, 16.15, 15.36. Anal. Calcd for $C_{21}H_{30}BrNO_6$: C, 53.40; H, 6.41; N, 2.97. Found: C, 53.37; H, 6.30; N, 2.93.

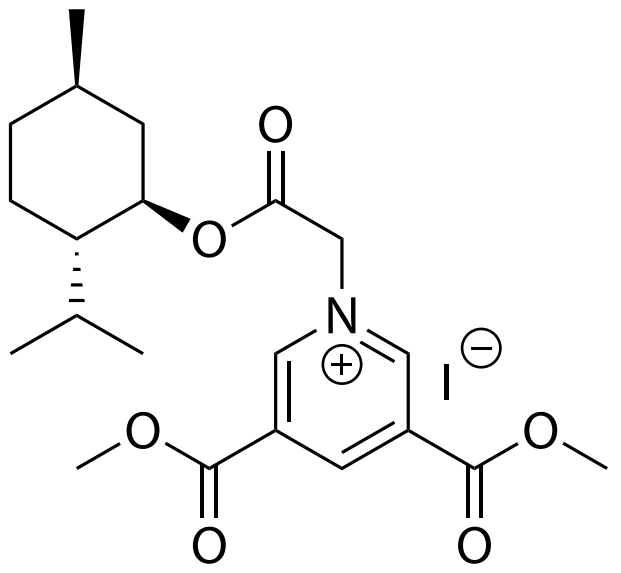

6. **6**^{Br} (0.10 g, 0.21 mmol, 1.00 equiv.) was dissolved in a suspension of KI in methanol (5 g in 30 mL). The resulting yellow suspension was filtered, and the solvent was removed under vacuum to afford an orange-yellow solid. The residue was extracted with dichloromethane (30 mL), filtered, and concentrated under vacuum to give an orange product. This metathesis procedure was repeated three additional times. The final precipitate was collected by filtration, washed with diethyl ether (3 × 20 mL), and dried under vacuum to afford the product as a yellow solid (0.08 g, 73% yield).

^{1}H NMR (500 MHz, $CDCl_3$) δ 9.91 (s, 2H), 9.40 (s, 1H), 6.26 (m, 1.6 Hz, 2H), 4.79 (td, *J* = 12.0, 5.5 Hz, 1H), 4.06 (s, 6H), 2.04 (d, *J* = 12.1 Hz, 1H), 1.93 – 1.84 (m, 1H), 1.71 (m, 2H), 1.47 (m, 2H), 0.91–0.76 (m, 11H). ^{13}C{^{1}H} NMR (126 MHz, $CDCl_3$) δ 164.59, 160.87, 150.26, 146.44, 130.18, 79.10, 65.94, 62.62, 54.27, 46.82, 40.72, 33.95, 31.59, 26.12, 23.16, 21.97, 20.95, 16.21, 15.36. Anal. Calcd for $C_{21}H_{30}INO_6$: C, 48.56; H, 5.82; N, 2.70. Found: C, 46.97; H, 5.78; N, 2.38.

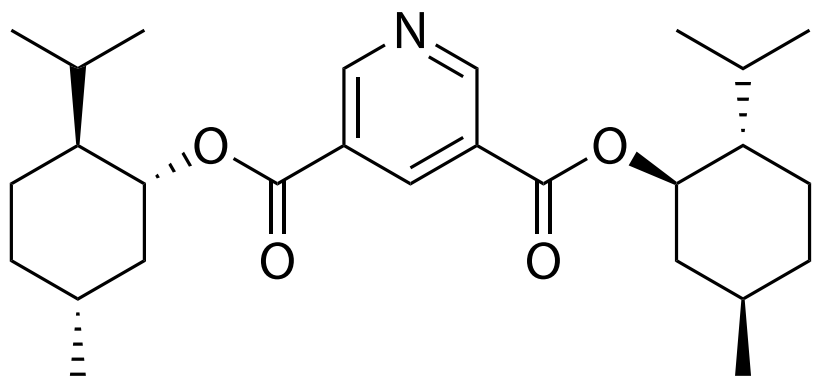

(–)-a5. Dichloroanhydride was prepared according published procedure.[2] Dinicotinic acid (3.00 g, 17.94 mmol, 1.00 equiv.) was suspended in thionyl chloride (30 mL), and two drops of DMF were added. The mixture was refluxed for 2 h, and the solvent was then removed under high vacuum. The resulting dichloroanhydride was dissolved in dichloromethane (10 mL) and added dropwise at 0 °C (ice–NaCl bath) to a solution of (–)-menthol (6.17 g, 39.47 mmol, 2.20 equiv.) and triethylamine (3.99 g, 39.47 mmol, 5.50 mL, 2.20 equiv.) in dichloromethane (30 mL). The reaction mixture was stirred for 30 min while warming to room temperature. The organic phase was separated, washed with saturated NaCl solution (3 × 60 mL), and concentrated under reduced pressure to yield a yellowish oil. The crude residue was purified by column chromatography (Silica gel 60, EtOAc/hexane, 1:10), and the solvent was removed under vacuum to afford the product as a white solid (6.52 g, 82% yield).

^{1}H NMR (500 MHz, $CDCl_3$) δ 9.33 (s, 2H), 8.83 (s, 1H), 4.98 (td, *J* = 10.9, 4.7 Hz, 2H), 2.11 (d, *J* = 10.9 Hz, 2H), 1.92 (m, 2H), 1.74 (m, 4H), 1.61 – 1.54 (m, 4H), 1.13 (m, 4H), 0.92 (m, 14H), 0.79 (d, *J* = 7.0 Hz, 6H). ^{13}C{^{1}H} NMR (126 MHz, $CDCl_3$) δ 164.19, 154.12, 138.07, 126.67, 76.08, 47.19, 40.93, 34.25, 31.55, 26.60, 23.62, 22.08, 20.82, 16.53. Anal. Calcd for $C_{27}H_{41}NO_4$: C, 73.10; H, 9.32; N, 3.16. Found: C, 72.98; H, 9.26; N, 3.11.

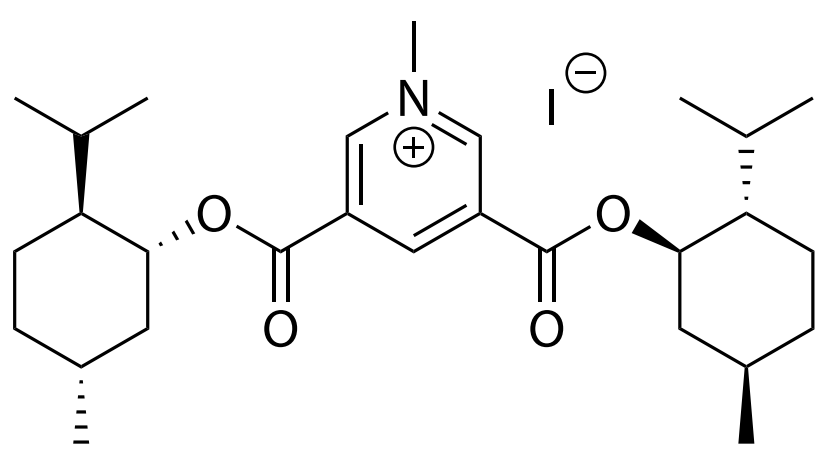

($^{-}$5). Chiral pyridine (**–**)-**a5** (0.80 g, 1.80 mmol, 1.00 equiv.) was dissolved in acetonitrile (15 mL), and iodomethane (0.22 mL, 3.60 mmol, 2.00 equiv.) was added in a sealed tube. The mixture was heated at 55 °C to afford a homogeneous, transparent yellow solution. After 12 h, the solvent was removed under vacuum to yield an orange solid, and cold diethyl ether (20 mL) was added. The resulting suspension was sonicated for 15 min, during which a pale yellow precipitate formed. The solid was collected, washed with cold diethyl ether (3 × 5 mL), and dried under vacuum to give a fibrous, wool-like yellow material

(0.77 g, 73% yield). Slow evaporation of a toluene solution of $^{-}$**5** under reduced pressure afforded $^{-}$**5** as a yellow needle–like crystalline material suitable for scXRD analysis.
^{1}H NMR (500 MHz, $CDCl_3$) δ 9.67 (s, 2H), 9.32 (s, 1H), 5.10 (td, *J* = 10.8, 4.6 Hz, 2H), 4.87 (s, 3H), 2.10 (m, 2H), 1.92 (m, 2H), 1.79 – 1.67 (m, 6H), 1.54 (m, 4H, submerged with residual signal of H_2O), 1.29 (dd, *J* = 11.7, 10.8 Hz, 2H), 1.12 (dd, *J* = 11.7, 10.8 Hz, 2H), 0.95 (m, 14H), 0.78 (d, *J* = 8.6 Hz, 6H). ^{13}C{^{1}H} NMR (126 MHz, $CDCl_3$) δ 160.11, 149.05, 145.19, 133.78, 131.02, 78.72, 58.45, 51.45, 46.75, 40.67, 33.9817, 31.65, 26.31, 23.25, 22.01, 20.94, 18.52, 16.21. Anal. Calcd for $C_{25}H_{44}INO_4$: C, 57.43; H, 7.57; N, 2.39. Found: C, 57.41; H, 7.35; N, 2.28.

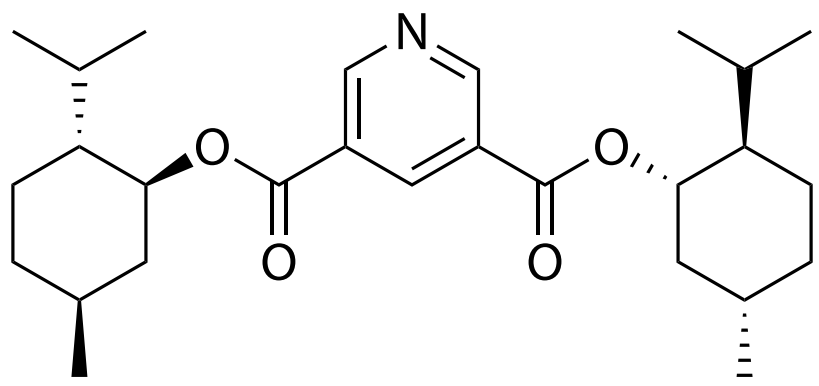

(**+**)-**a5**. Synthesized similarly to (**–**)-**a5** from dinicotinic acid (2.34 g, 14.01 mmol, 1.00 equiv.), thionyl chloride (25 ml), (+)-menthol (4.81 g, 30.83 mmol, 2.20 equiv.) and triethylamine (3.11 g, 30.83 mmol, 4.29 mL, 2.20 equiv.) to afford (**+**)-**a5** as white crystalline material (4.91 g, 79%). NMR spectroscopy data is identical to (**–**)-**a5**.

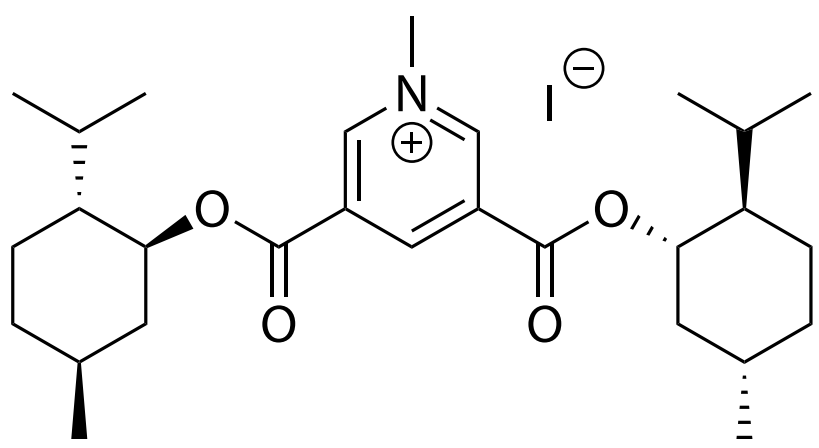

($^{+}$5). Synthesized similarly to $^{-}$**5** from (**+**)-**a5** (1.21 g, 2.73 mmol, 1.00 equiv.) and iodomethane (0.34 mL, 5.45 mmol, 2.00 equiv.) to $^{+}$**5** afford a pale yellow precipitate (1.34 g, 84%). NMR spectroscopy data is identical to $^{-}$**5**.

NB! Modified $^{-/+}$**M DPs** were methylated, giving $^{-/+}$**5** (64%) as yellow solids, being soluble in toluene and diethyl ether at concentrations 10^{-3} M at room temperature.

Characterization methods.

TGA measurements were carried out using a Mettler Toledo TGA 2 instrument in the temperature range of 30–600 °C with a heating rate of 10 °C min^{-1}, under a nitrogen atmosphere (flow rate 50 ml min^{-1}). 70 μl aluminum oxide pans were used for the experiments. Before the experiments, the pans were tempered at 1000 °C for 10 minutes and kept in an exicator to remove and protect from humidity. **DSC** measurements were carried out using a Mettler Toledo DSC 823^{e} instrument in the temperature range from –120 to 400 °C with a heating rate of 10 °C min^{-1}, under a nitrogen atmosphere (flow rate 50 ml min^{-1}). 40 μl aluminum pans were used for the experiments, with an empty pan serving as a reference. **HPLC** chromatograms were obtained from an Agilent 1260 Infinity II liquid chromatograph equipped with a diode array detector (G71115A, monitored at 260 nm), binary pump, heated column compartment at 25 °C, and autosampler controller. Phenomenex Gemini 3μm C18 column (3.5-micrometer diameter particle size, 4.6×150 mm) was used for all analytical experiments. 2 μl aliquots were injected and eluted with H_2O/MeCN solution (TFA 0.1%) in a gradient mode (5-30% of MeCN) within 13 minutes. **Elemental analysis (EA)** was carried out in the analytical laboratory of the University of Eastern Finland. **PXRD diffractograms** were collected from bulk recrystallized material (three

averaged replicas from ground crystalline material) using a Rigaku XtaLAB Synergy-R with Cu K_α radiation (λ = 1.54184 Å, Microfocus beam, Divergence 2.3 mRad, 600 sec exposure time) with Gandolfi strategy for data collection. Calculated data was generated using Mercury 2024.3.1 (Build 428092) software.

Photophysical measurements were performed under an inert atmosphere of argon. Solution samples were prepared in aerated conditions at approx 10^{-4}–10^{-5} M concentration in spectroscopy-grade solvents that were additionally dried over molecular sieves. Before measurements, solid samples were dried under a vacuum (10^{-3} mbar) for at least 30 minutes in order to avoid the influence of residual solvent and water. Both excitation and emission spectra of the samples were recorded on an Edinburgh Instrument FLS1000 spectrometer, equipped with a 450 W ozone-free Xenon arc lamp, double monochromators for the excitation and emission pathways, and a red-sensitive photomultiplier (PMT-980) as detector. The excitation and emission spectra were corrected using the standard corrections supplied by the manufacturer for the spectral power of the excitation source and the sensitivity of the detector. Variable temperature and low-temperature measurements were performed using an Oxford Optistat DN 77K cryostat. The solid samples' quantum yields were measured using an integrating sphere QYPro in a direct mode (Edinburgh). Solid-state absorption spectra were acquired with an integrating sphere (Edinburgh Instruments) via synchronous scanning relative to a blank substrate. The luminescence lifetimes were measured using an EP-LED pulsed laser diode (320, 365 nm), Xenon flashlamp, or VP-LED laser diode (375 nm) with the multichannel-scaling (MCS) or time-correlated single photon counting (TCSPC) methods for short lifetimes. The emission was collected at right angles to the excitation source, with the emission wavelength selected using a double-grated monochromator and detected by the respective PMT. Solid-state reflectance spectra were measured using a PerkinElmer Lambda 900 UV/Vis/NIR spectrophotometer equipped with an integrating sphere. Fluoracle and FAST spectrometer operating software and Origin Pro 2019 9.6.0 were used for data analysis and processing.

UV-Vis titration experiments of $\mathbf{1}^{BARF}$, fitting and obtaining apparent binding constants from fitting isotherms were performed using the technique described previously.[3,4]

Fluorescence up-conversion. Ultrafast fluorescence up-conversion measurements (FOG100-DX, CDP Corp.) were carried out using a stable 120-femtosecond laser oscillator (Tsunami, Spectra-Physics). The pump beam (400 nm) was generated from the fundamental output of the oscillator (800 nm) through second harmonic generation (SHG) using a β-barium borate (BBO) crystal. A neutral density (ND) filter was employed to control the energy of the pump beam following SHG. A lens system was subsequently used to focus the pump beam onto the sample, collect the resulting fluorescence, and direct the fluorescence onto a BBO crystal (cut at 38°) for sum-frequency generation.

The crystalline samples were uniformly dispersed onto the sample cell, and measurements were performed in transmission mode using a rotating sample stage to ensure sample uniformity. The gate beam (800 nm) was routed through a mechanical delay line and intersected the fluorescence beam collinearly at the sum-frequency BBO crystal. The polarization angle between the pump and gate beams was set to the magic angle (54.7°) to suppress polarization-dependent artifacts arising from molecular rotation. The generated sum-frequency signal was passed through a monochromator and detected by a photomultiplier tube (PMT). The full width at half

maximum (FWHM) of the instrument response function (IRF) was approximately 220 femtoseconds.

Radioluminescence spectra were collected at 298 K from pressed crystalline materials **1**, **2** and **4** using an Ocean Optics SR-6VN500-10 (400–1040 nm) compact spectrometer with optical fiber and collimating lens. Samples were irradiated continuously with CuKα (λ = 1.54184 Å) source (INCOATEC microfocus sealed tube, 50 kV, 1 mA). Spectra were obtained with an integration time of 5 seconds and further corrected for baseline dark counts.

Single crystal X-ray diffraction (SCXRD) and **three-dimensional electron diffraction (3DED)**: The single crystals were immersed in a film of NVH mounted on a polyimide microloop (MiTeGen), transferred to a stream of cold nitrogen (Cryostream 700 cooling system by Oxford Cryosystems), and measured at a temperature of 100–270 K. The X-ray diffraction data were collected on a Rigaku XtaLAB Synergy-R diffractometer equipped with a HyPix-Arc100° detector using mirror-monochromated Cu Kα (λ = 1.54184 Å) radiation (INCOATEC microfocus sealed tube). Electron diffraction data were collected using the Rigaku XtaLAB Synergy-ED diffractometer (for *p*–**pyI** at 175 K and *m*-**pyI** at 293 K) equipped with a Rigaku HyPix-ED detector optimized for operation in the continuous rotation 3D-ED experimental setup.[5,6] Data acquisition was performed under high vacuum with an electron wavelength of 0.0251 Å (200 kV). (HyPix-ED). The samples *p*–**pyI** and *m*-**pyI** were ground up between microscopic slides before being loaded on a lacey-carbon 200 mesh Cu grid. The sample consisted of plate-like crystallites of a few 100 nanometers in thickness. Two datasets for *p*–**pyI** and *m*-**pyI** were used for merging. By merging multiple datasets, more complete, significant, and redundant data was obtained, resulting in a better quality structure refinement. The frames were integrated with the CrysAlis^{Pro} software package using a narrow-frame algorithm (Rigaku Oxford Diffraction, CrysAlis^{Pro} software system, Rigaku Corporation, Wrocław, Poland, 2024 (version 1.171.44.115a)). The CrysAlis^{Pro} for ED program package was used for unit cell refinements, merging, and data reductions. The structure was solved using the intrinsic phasing method, and refined and visualized with the OLEX2-1.5 program.[7] A semiempirical absorption correction (SCALE3 ABSPACK) was applied to all data. All non-hydrogen atoms were refined anisotropically. Hydrogen atoms were included in the structure factor calculations. All hydrogen atoms were assigned to idealized geometric positions. The crystallographic details are summarized in Tables S2–7. CCDC **2551206**–**2551231** contains supplementary crystallographic data for this paper.

Theoretical calculations were performed using the Orca (version 6.0.1) software package.[8] To model the solid-state materials, QM/MM (quantum mechanics/molecular mechanics) models were utilized. The experimentally obtained crystal structures were expanded in three dimensions to form a supercell. From the centre of the supercell, one cation-anion pair (with the shortest anion-π distance) was assigned as the QM layer and described at the density functional theory (DFT) level of theory. Surrounding ions in the supercell were assigned as the MM layer and their coordinates were kept fixed during optimization. The MM layer was described with a simple forcefield generated with Orca's built-in makeff utility program. Electronic embedding was used to account for solid-state solvation effects. Ground state geometry optimizations were performed with DFT, while time-dependent DFT (TD-DFT) was utilized for excited state geometry optimizations using the PBE0 hybrid density functional[9] together with a def2-TZVP basis set and the corresponding

effective core potential (ECP) for iodine atoms.[10] Frequency calculations were carried out for all optimized structures. Single-point TD-DFT calculations were performed for each optimized structure using the scalar-relativistic X2C Hamiltonian together with perturbative inclusion of spin-orbit coupling effects.[11,12] The relativistic calculations utilized the X2C-TZVPAll all-electron basis set for all atoms.[13] Radiative rates for phosphorescence and non-radiative rates for intersystem crossing were calculated using a Fermi Golden Rule approach implemented in Orca's excited state dynamics (ESD) module.[14] ESD calculations were performed in the Franck-Condon limit, neglecting contributions from Herzberg-Teller coupling. All calculations utilized the resolution-of-the-identity and chain-of-spheres for exchange (RIJCOSX) algorithms to reduce the computational burden.[15–18]

To gain insight into bonding and iodide-π^+ interactions, the topology of the electron density was analyzed using the Quantum Theory of Atoms in Molecules (QTAIM) approach. Critical point search and electron density analysis were conducted with the AIMAll program, using wavefunctions obtained from the DFT calculations. To simulate the absorption spectra, 50 lowest-lying singlet-singlet and singlet-triplet excitations were calculated, and the simulated spectra were plotted using a Gaussian broadening function (FWHM = 0.3 eV). Furthermore, to characterize the nature of electronic transitions, Natural Transition Orbitals (NTOs) were obtained for the 20 lowest-lying singlet-singlet excitations for compound **1**. Molecular graphs illustrating the bond paths and critical points were generated using AIMAll.

Scintillation film preparation: Crystalline **1** was finely ground for 15 min in a mortar and suspended in DCM (100 mg/mL). After stirring, the solution was drop-casted on a glass substrate at 45 ^{o}C.

X-ray scintillator measurements: The X-ray attenuation efficiency was calculated from the following equation:

$$\text{X-ray attenuation efficiency (\%)} = (1-e^{-\mu t})\times 100\%$$

Where μ is the product of the attenuation coefficient and the density of the sample. The attenuation coefficient was calculated from the National Institute of Standards and Technology (NIST) database. The linearity of the RL response and the limit of detection (LoD) were measured with a photon-counting system combining a PMT (PMC-100-20, Becker & Hickl) and a single photon counter (SR400, Stanford Research Systems). The light yield was calculated considering the PMT responsivity across different wavelengths and the X-ray attenuation of the samples. All samples were measured under the same illuminated area defined by a 1-cm aperture and the same dose rate.

$$\frac{LY_{sample}}{LY_{CsI:Tl}} = \frac{R_{sample}/A_{sample}}{R_{CsI:Tl}/A_{CsI:Tl}} = \frac{\int I_{sample}(\lambda)S(\lambda)d\lambda / \int I_{sample}(\lambda)d\lambda}{\int I_{CsI:Tl}(\lambda)S(\lambda)d\lambda / \int I_{CsI:Tl}(\lambda)d\lambda} \div \frac{A_{sample}}{A_{CsI:Tl}}$$

Where R is the RL counts, I(λ) is the RL intensity, S(λ) is the responsivity of the PMT and A is the equivalent X-ray attenuation efficiency.

The X-ray images were acquired by a commercial camera (ZV-E10, Sony). The modulation transfer function (MTF) was calculated using the slanted-edge method from the following equation:

$$MTF\ (v) = F\left(LSF\ (x)\right) = F\ (\frac{dESF(x)}{dx})$$

The edge spread function (ESF) was obtained from an edge image of 0.03 mm lead sheet. The line spread function (LSF) was derived by differentiating the ESF. The MTF was then obtained by performing a Fourier transform on the LSF.

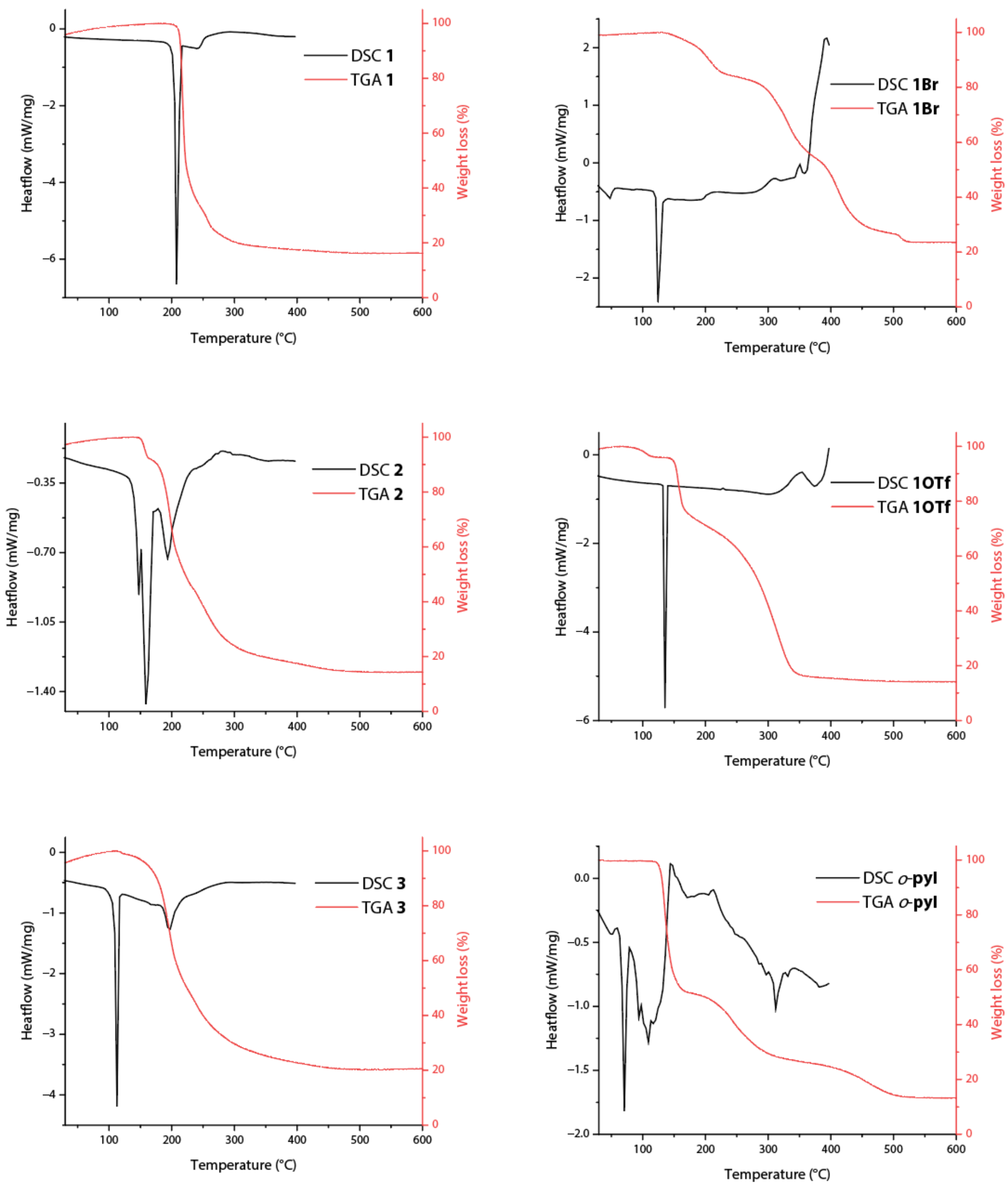

Figure S1. DSC (black trace) and TGA (red trace) data of the studied salts **1–3**, $\mathbf{1}^{Br}$, $\mathbf{1}^{OTf}$, *o*-**pyl**.

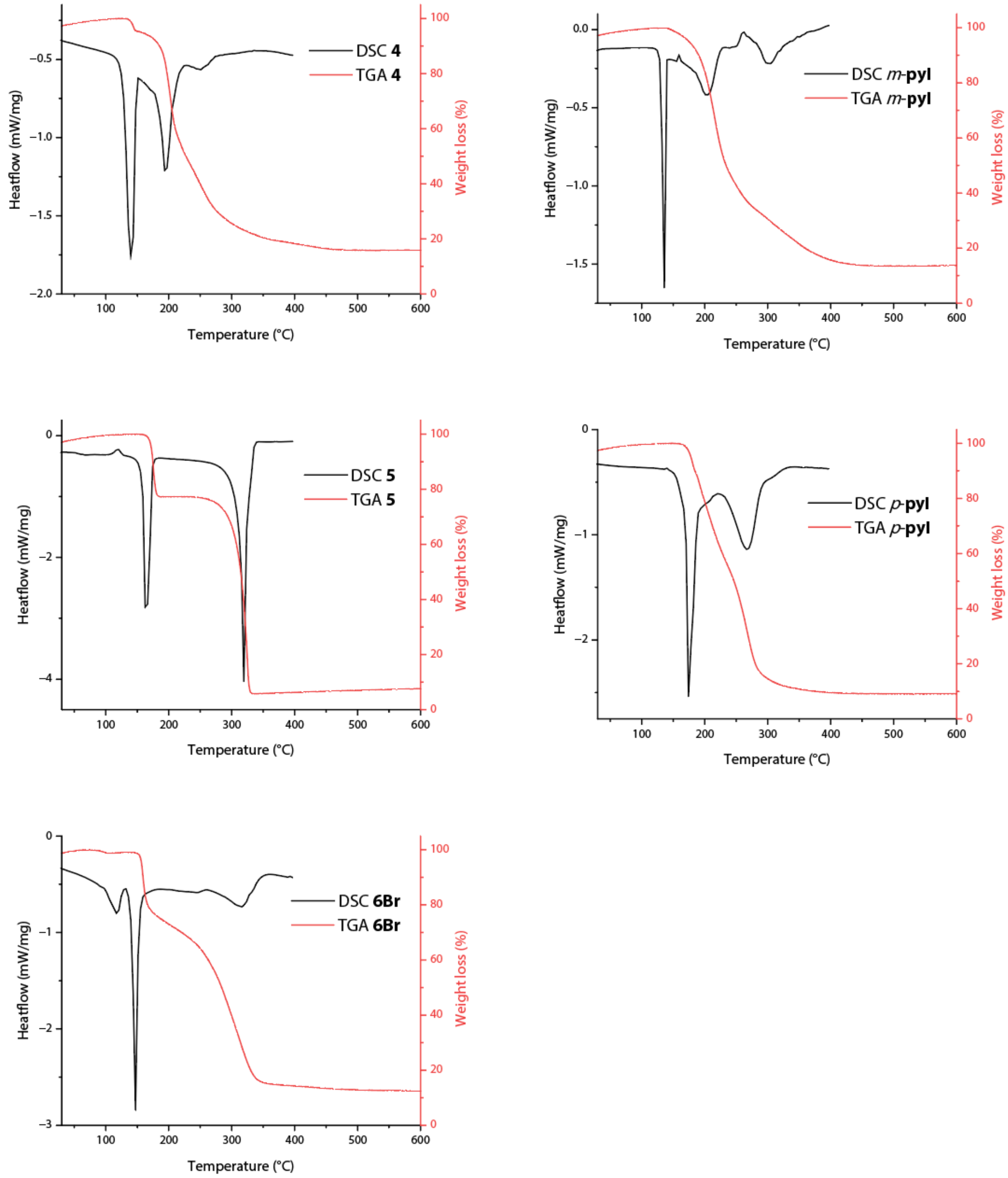

Figure S2. DSC (black trace) and TGA (red trace) data of the studied salts **4–6**, *m*-**pyl**, *p*-**pyl**.

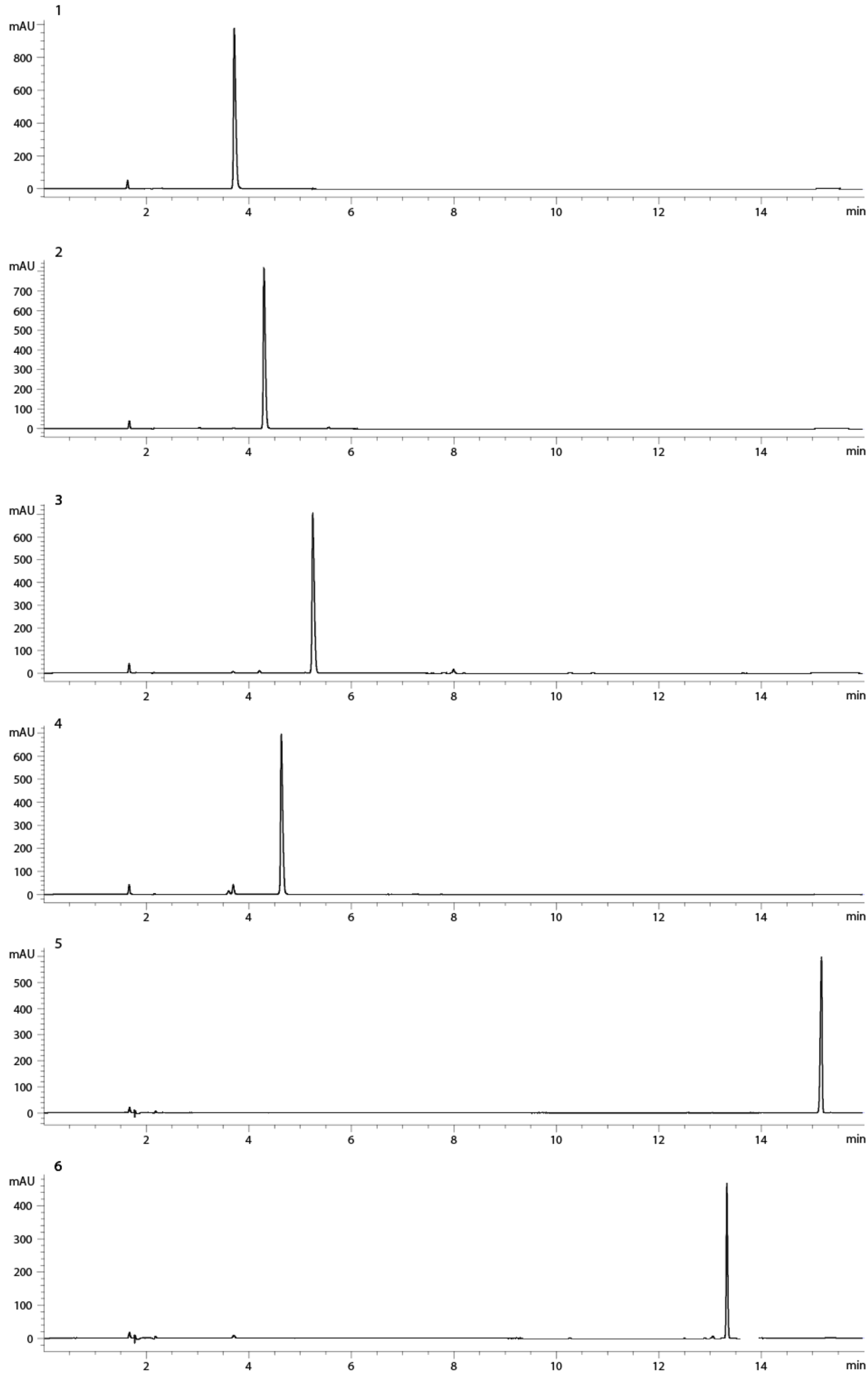

Figure S3. HPLC chromatograms of pyridinium salts **1–6** (monitored at 260 nm).

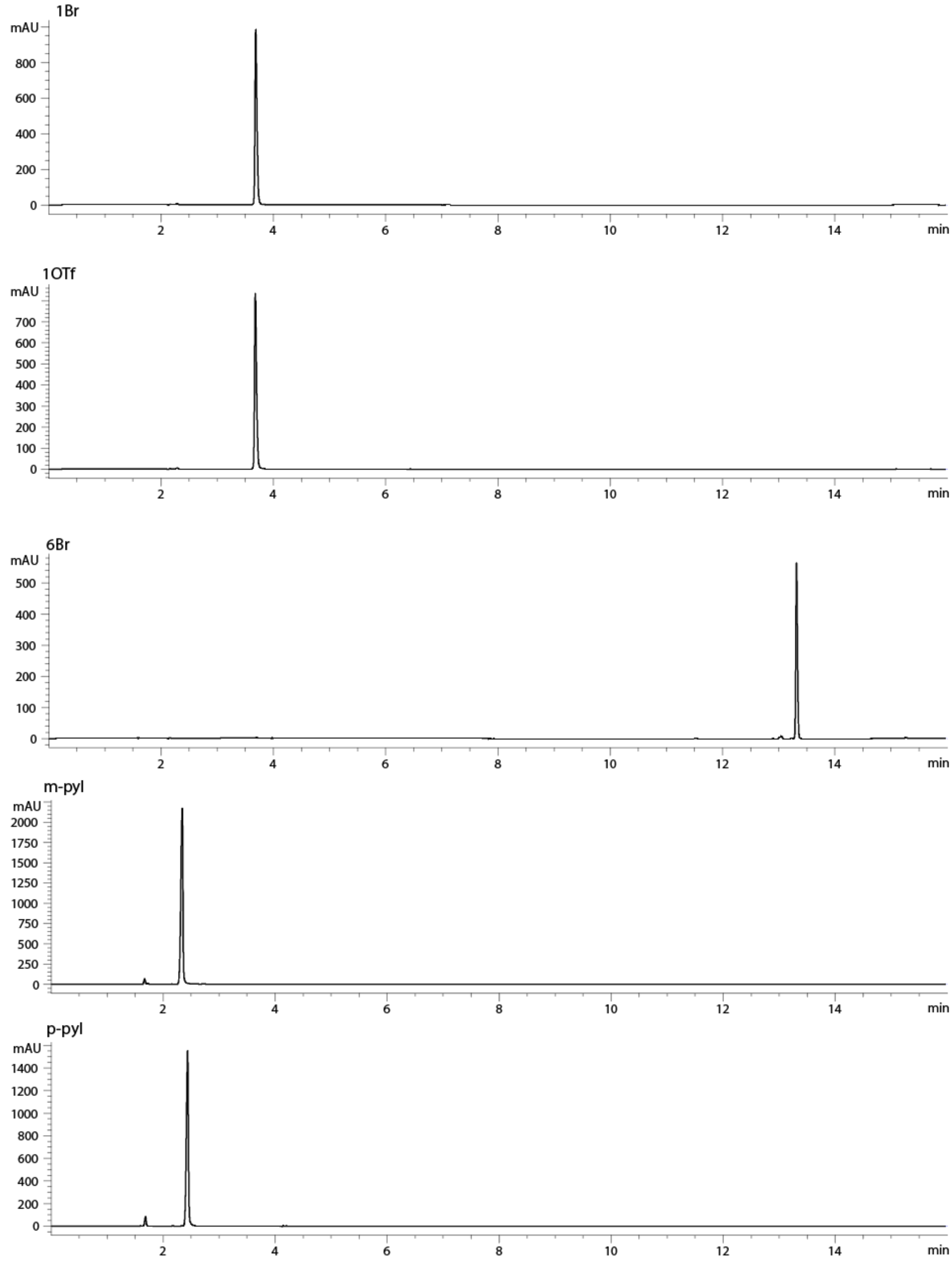

Figure S4. HPLC chromatograms of pyridinium salts **1**^{Br}**,** **1**^{OTf}**,** **6**^{Br}**,** *m*-**pyl** and *p*-**pyl** (monitored at 260 nm).

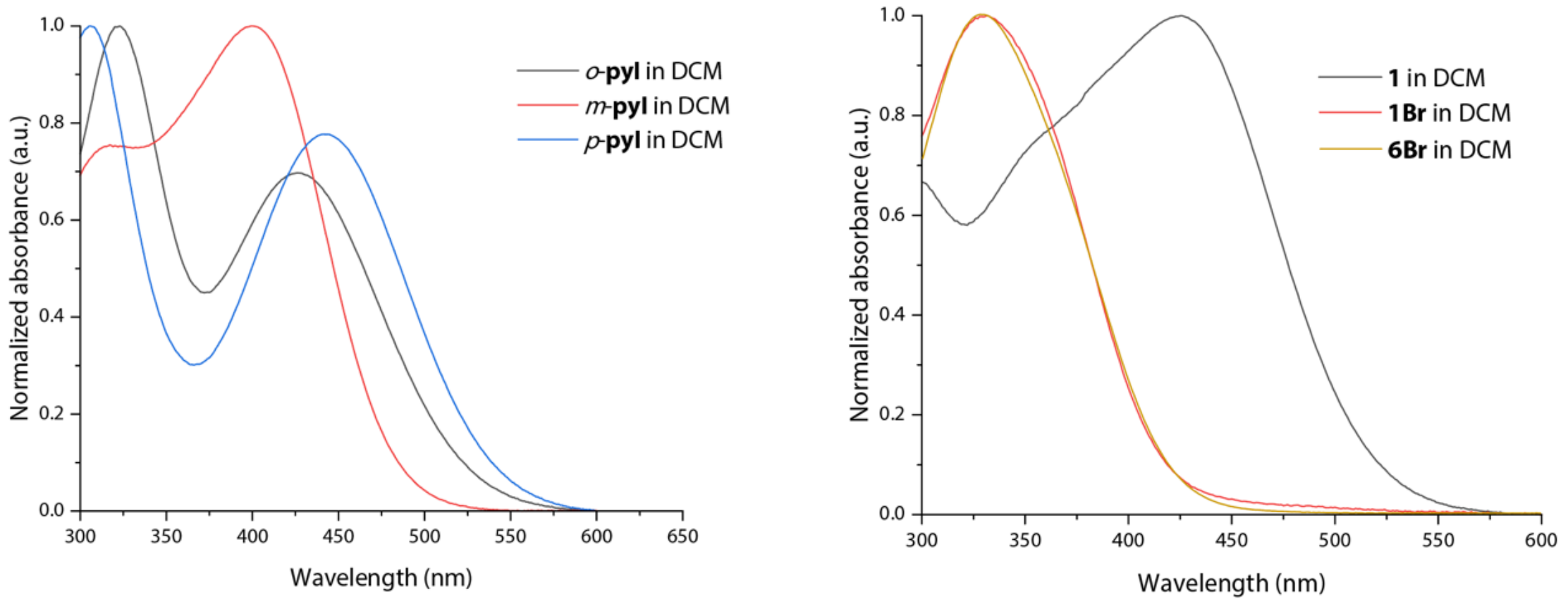

Figure S5. Normalized absorption profiles of the *x*-**pyl** and **1**, **1**^{Br}, **6**^{Br} measured in DCM (approx. 10^{-4} M).

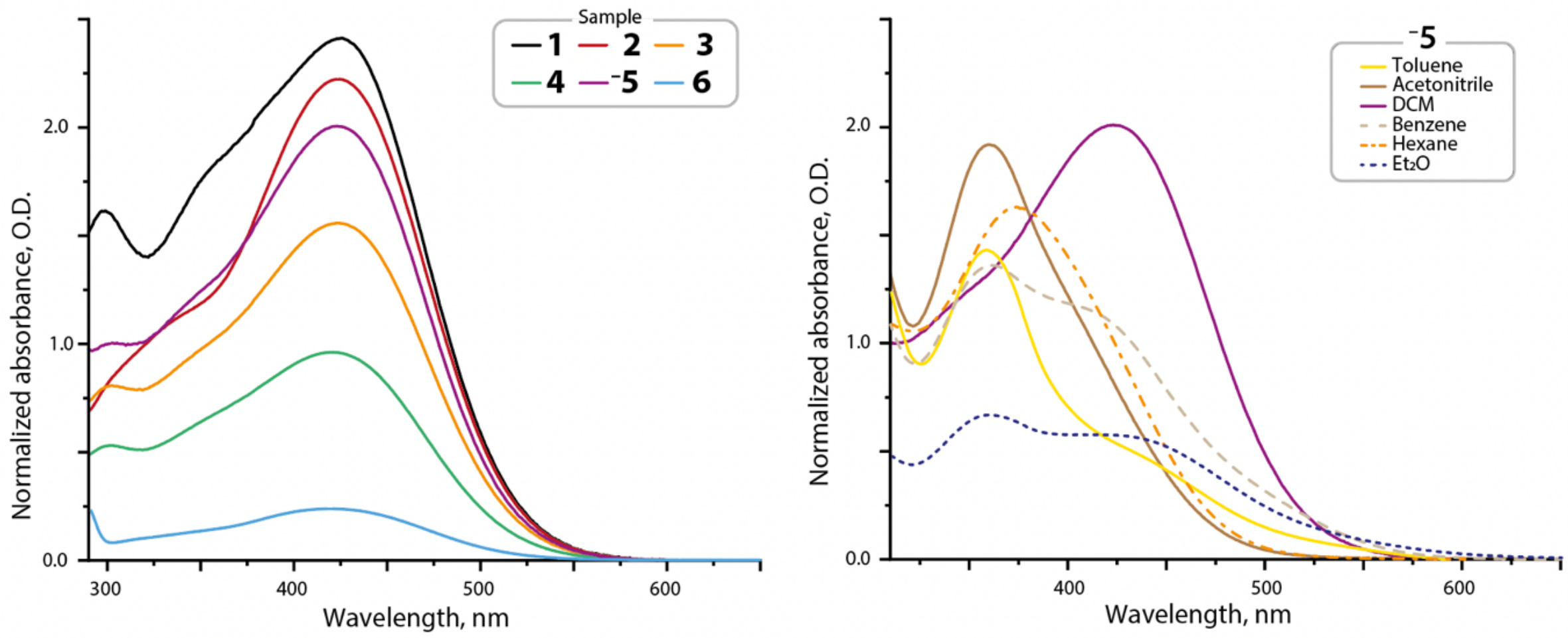

Figure S6. Absorption profiles of the samples **1-6** at 10^{-4} M measured in DCM (left) and in a variety of solvents for $^{-}$**5**.

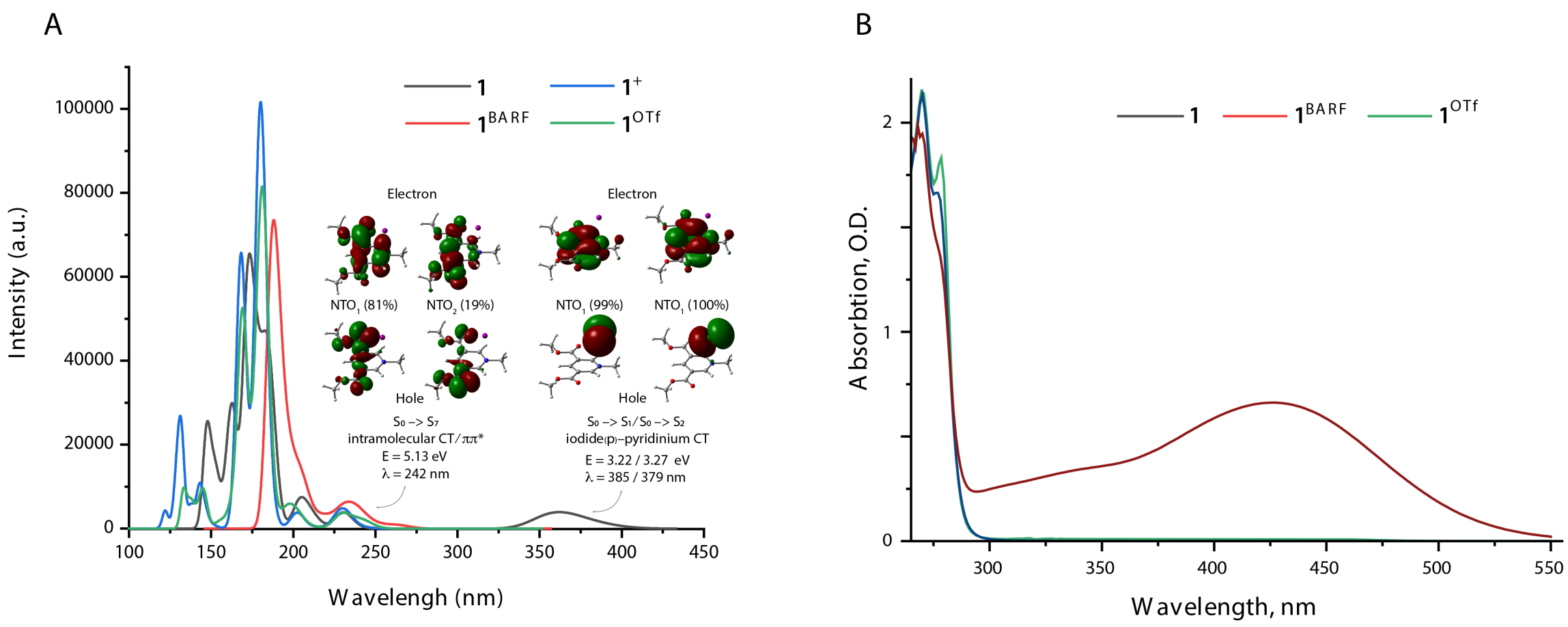

Figure S7. (A) Calculated (DFT) and (B) experimental (in DCM, 10^{-4} M) absorption spectra of **1**, $\mathbf{1}^{X}$ (X = BARF, OTf) and cation $\mathbf{1}^{+}$. Inset shows selected natural transition orbitals (NTO) and their energies.

Table S1. Calculated singlet excited states ($S_0 \rightarrow S_n$, n = 1–9) of **1** supplemented with energies, NTO weights, transition character, and representative hole/electron NTO.

$S_0 \longrightarrow S_n$	E [eV]	λ [nm]	NTO1 w%	NTO2 w%	Character	NTO1 (Hole)	NTO1 (Electron)
1	3.22	385	0.99		I->π* CT	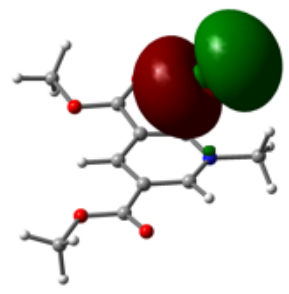	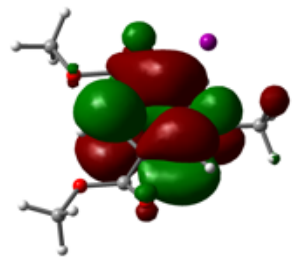
2	3.27	379	1.00		I->π* CT	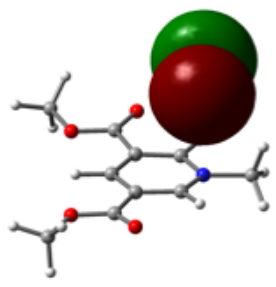	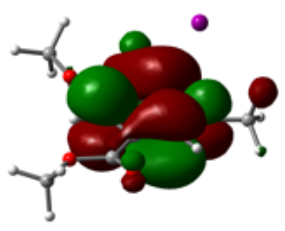
3	3.34	371	0.98		I->π* CT	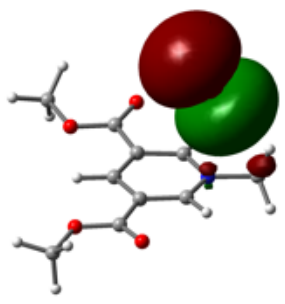	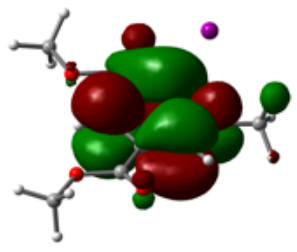
4	3.36	369	1.00		I->π* CT	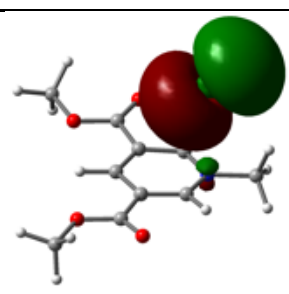	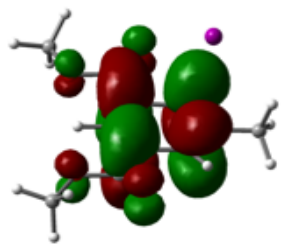
5	3.46	359	0.98		I->π* CT	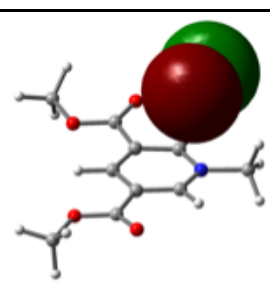	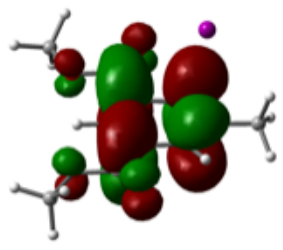
6	3.48	357	1.00		I->π* CT	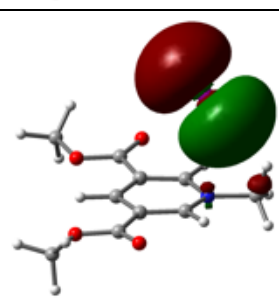	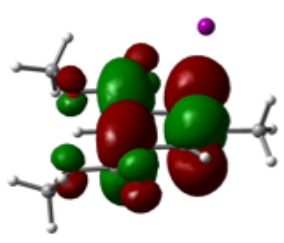
7	5.13	242	0.81	0.19	intra mol. CT/π-π*	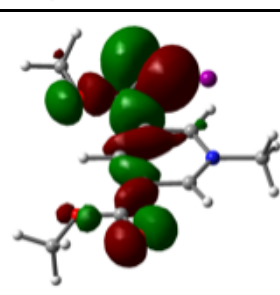	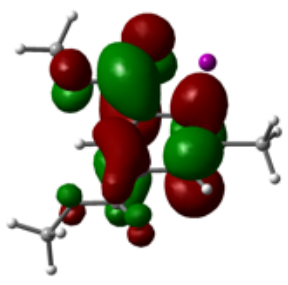
8	5.22	237	0.72	0.28	intra mol. CT/π-π*	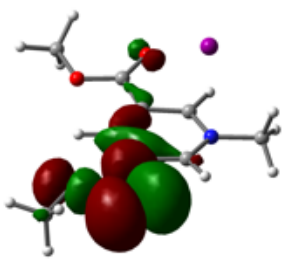	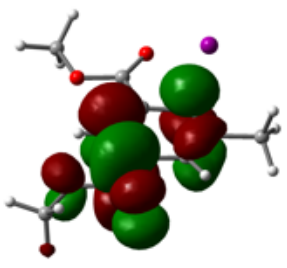
9	5.38	231	0.66	0.33	π-π*	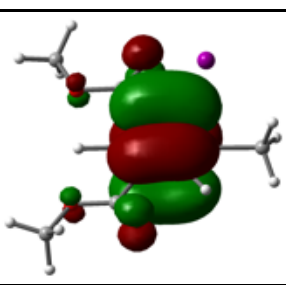	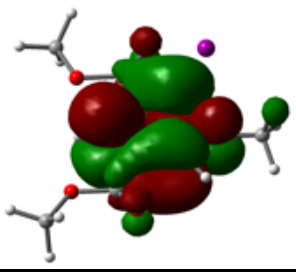

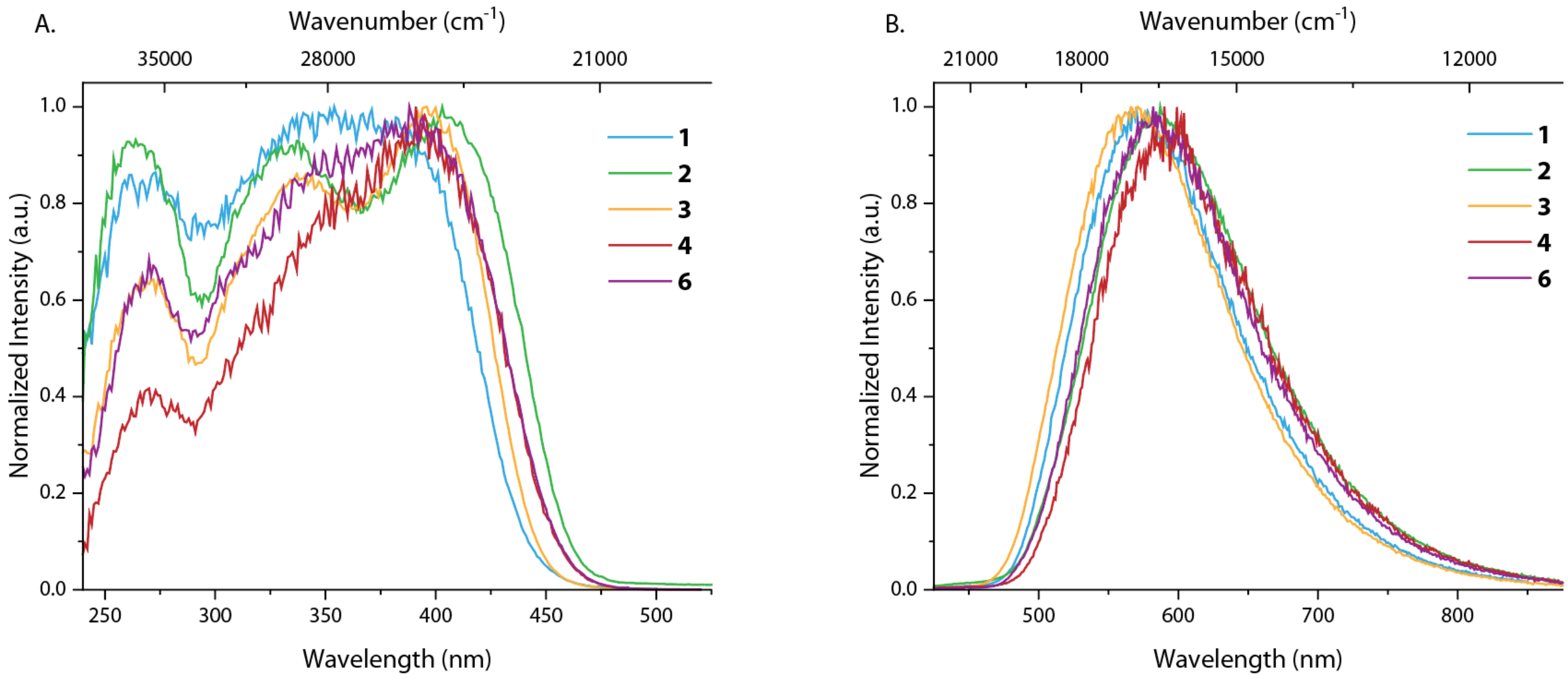

Figure S8. Steady-state excitation (A.) and emission (B.) spectra of frozen DCM solutions of **1–4** and **6** measured at 77 K.

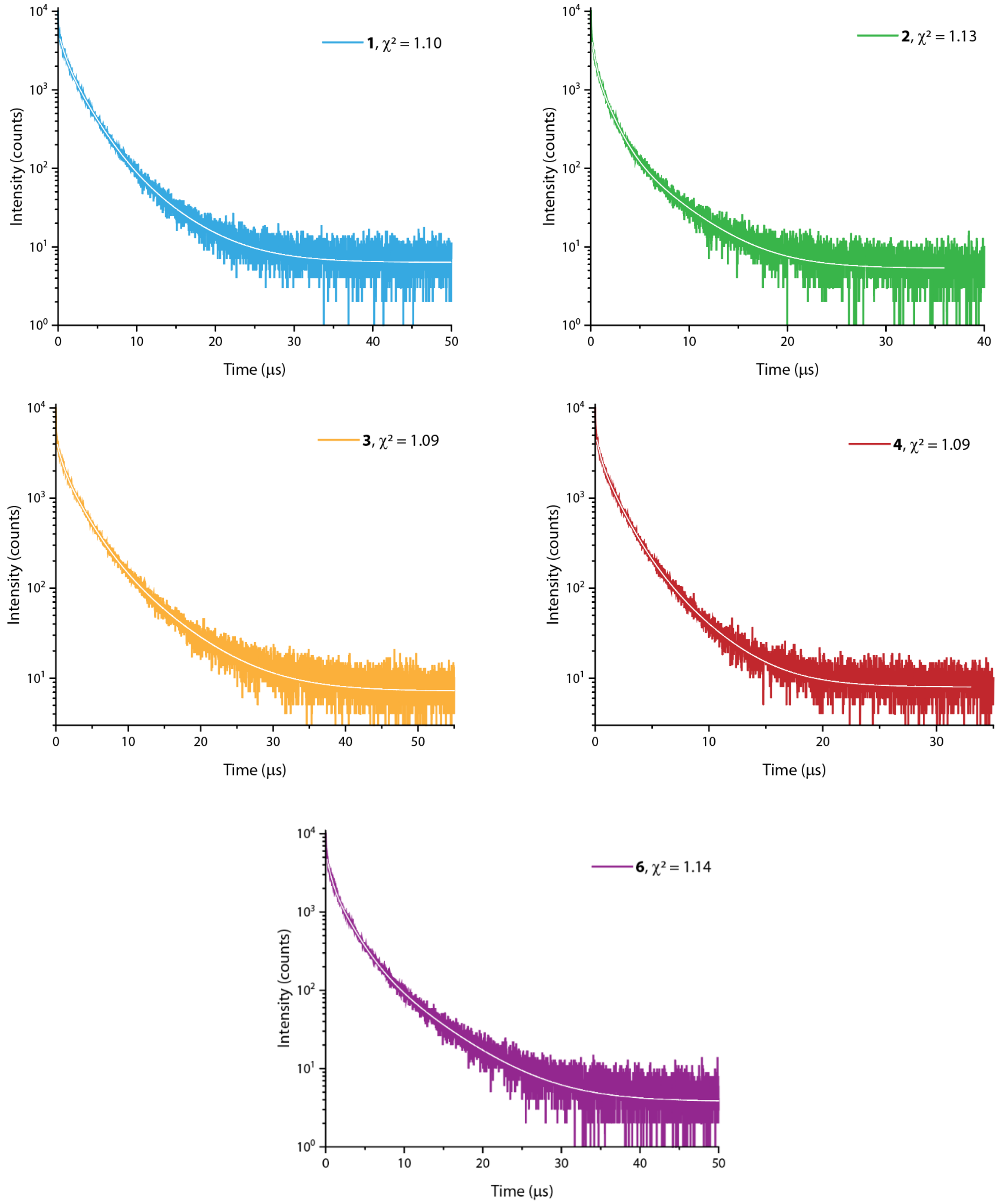

Figure S9. Time-resolved and fitted decay curves of frozen DCM solutions of **1**–**4** and **6** measured at 77 K.

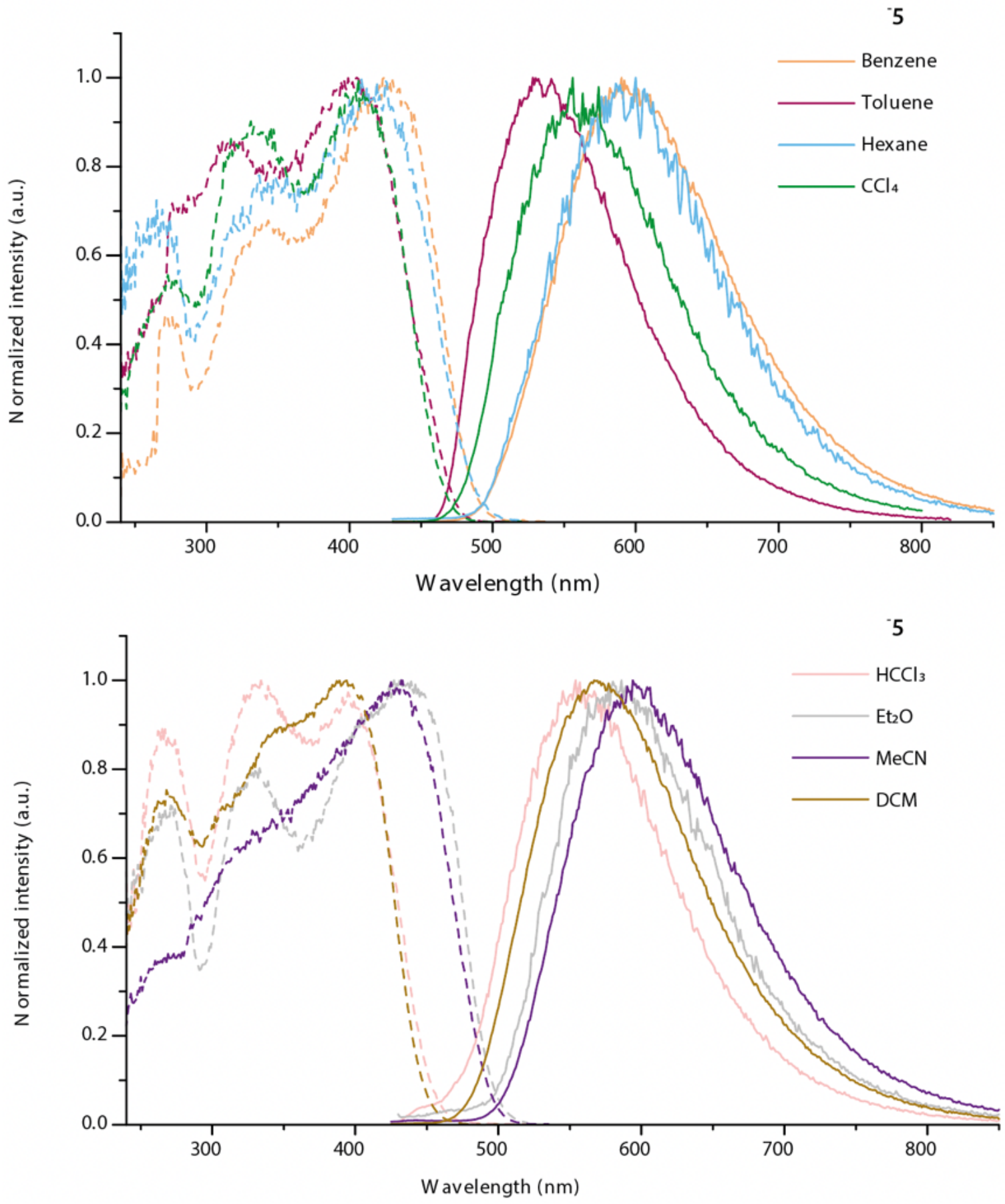

Figure S10. Steady-state emission and excitation spectra of frozen solutions of $^{-}$**5** measured at 77 K.

Table S2. Summary of the photophysical properties of frozen solutions of **1**–**6** (all solutions at approx. 10^{-4} M).

	λ_{exc} [nm]	λ_{em} [nm]	τ (A_i) [μs]	τ_{av} [μs][a]
**1** in DCM	267, 359	574	0.67 (37), 2.13 (55), 5.26 (8)	1.84
**2** in DCM	264, 334, 405	589	0.30 (48), 1.19 (45), 4.14 (7)	0.96
**3** in DCM	270, 339, 396	570	0.64 (34), 2.40 (54), 6.29 (12)	2.29
**4** in DCM	271, 392	593	0.33 (33), 1.37 (55), 3.43 (12)	1.23
$^{-}$**5** in DCM	269, 390	572	0.52 (32), 2.18 (54), 5.67 (14)	2.15
$^{-}$**5** in MeCN	428	597	0.54 (37), 1.92 (53), 4.67 (10)	1.69
$^{-}$**5** in $CHCl_3$	333, 395	555	1.90 (55), 5.55 (45)	3.49
$^{-}$**5** in Et_2O	402, 435	585	0.12 (5), 1.80 (69), 4.57 (26)	2.42
$^{-}$**5** in CCl_4	405	560	0.64 (41), 2.60 (51), 7.62 (18)	2.20
$^{-}$**5** in Hexane	415	590	0.11 (2), 1.12 (24), 3.49 (74)	1.03
$^{-}$**5** in Toluene	400	540	1.00 (9), 2.61 (24), 7.51 (67)	2.29
$^{-}$**5** in Benzene	428	592	0.13 (42), 1.03 (45), 2.88 (13)	0.87
**6** in DCM	270, 384	585	0.48 (44), 1.94 (48), 5.83 (8)	1.63

[a] Amplitude-weighted average lifetime, calculated from $\tau_{av}=\Sigma A_i\tau_i$, A_i=weight of the i-th exponent.

NB! Steady-state and time-resolved photoluminescence measurements of frozen solutions demonstrate that all investigated contact ion pairs (**1**–**6**) exhibit broad charge-transfer (CT) emission with maxima spanning 540–597 nm and microsecond excited-state lifetimes of multi-exponential origin (τ_{av} = 0.87–3.49 μs, Table S1), consistent with phosphorescence originating from the multiple anion→π^+ CT excited state. The absence of nanosecond fluorescence under cryogenic conditions indicates efficient population of the triplet manifold following photoexcitation. The excited-state properties remain sensitive to the frozen surrounding solvent environment, as exemplified by compound $^{-}$**5**, which was investigated in a series of solvents of different polarities and coordinating abilities. Depending on the solvent, both the excitation (λ_{exc} = 333–435 nm) and emission (λ_{em} = 540–597 nm) maxima vary considerably, while the phosphorescence lifetime ranges from 0.87 to 3.49 µs. These observations indicate that the energy of the CT state is perturbed by solvent-dependent ion-pairing and specific solvation effects, reflecting the dynamic nature of through-space anion→π^+ charge-transfer interactions even in frozen matrices.

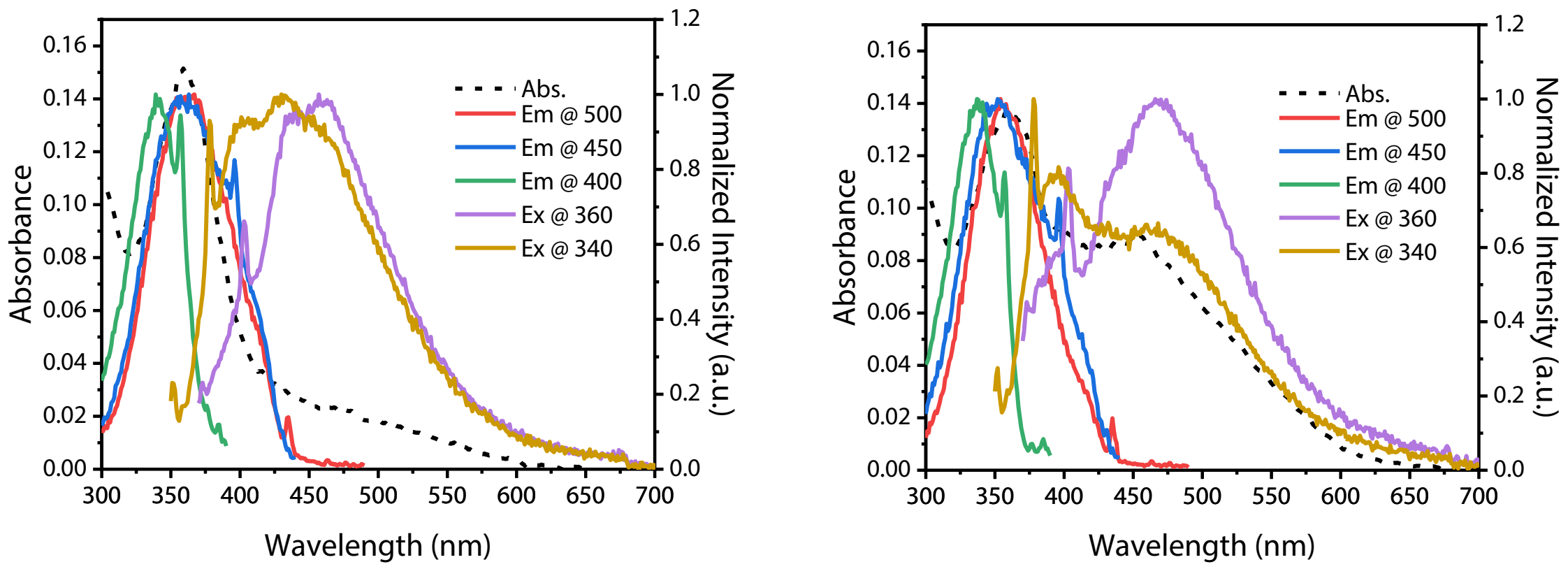

Figure S11. Steady-state absorption, emission, and excitation spectra of $^{-}$**5** measured at 297 K in aerobic conditions in toluene. Results demonstrate multiple species present in solution upon excitation and their evolution over time.

NB! During the course of the photophysical investigation, compound $^{-}$**5** was found to exhibit limited stability in solutions, with the extent of degradation strongly depending on the solvent and storage conditions. In particular, prolonged exposure of toluene solutions to ambient conditions resulted in gradual changes in the absorption and emission spectra, indicating partial decomposition of the sample.

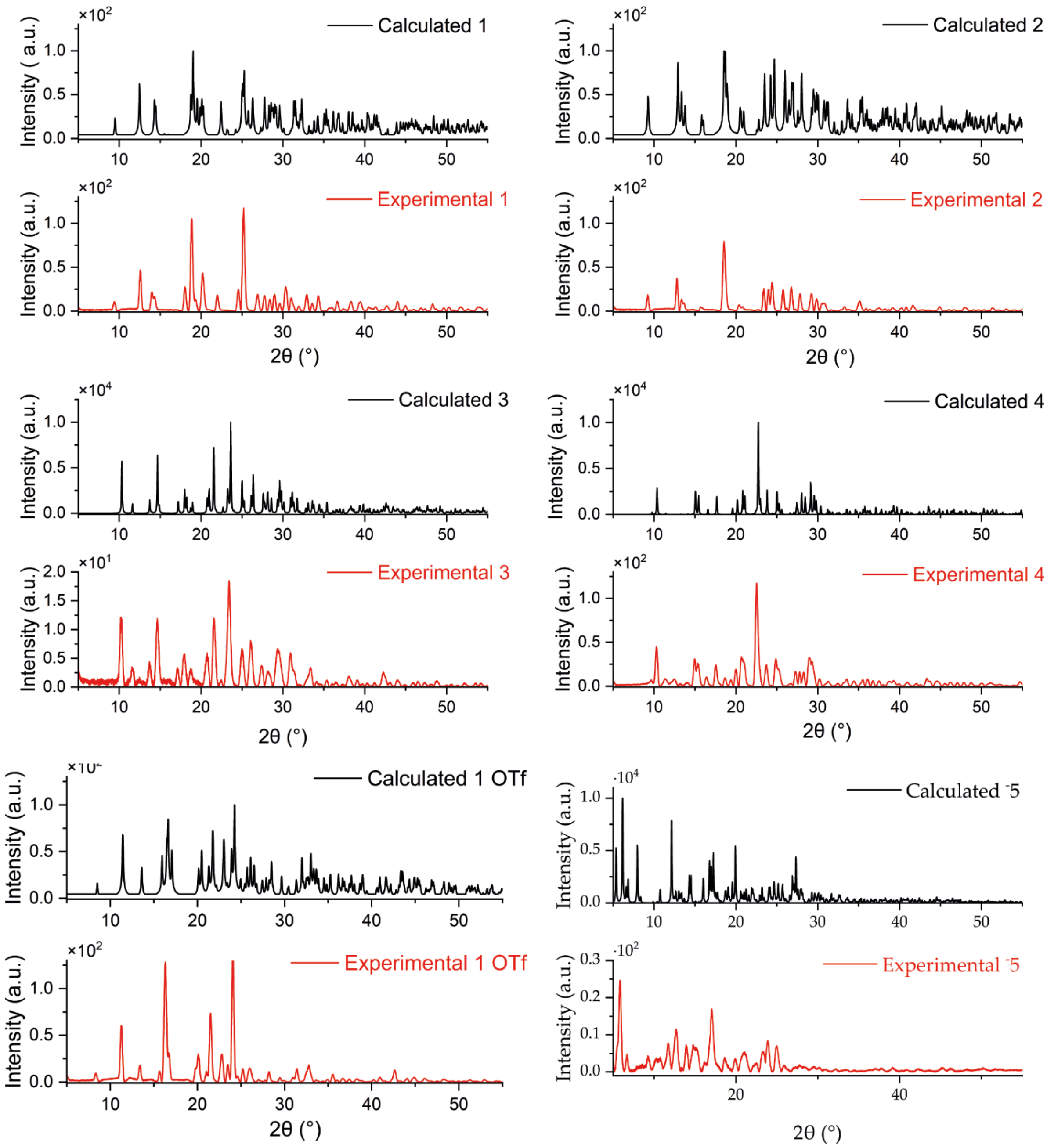

Figure S12. Experimental and calculated pXRD data of the **1–4**, **⁻5, 1**^{OTf}.

Table S3. Crystal data and structure refinement for *o*-, *m*-, *p*-**pyl**, **1**^{Br} and **1**^{OTf}.

Identification code	*o*-**pyl**	*m*-**pyl** *	*p*-**pyl** *	**1**^{Br}	**1**^{OTf}
CCDC number	2551206	2551207	2551208	2551209	2551210
Empirical formula	$C_8H_{10}INO_2$	$C_8H_{10}INO_2$	$C_8H_{10}INO_2$	$C_{10}H_{12}BrNO_4$	$C_{11}H_{12}F_3NO_7S$
Formula weight	279.07	279.07	279.07	290.12	359.28
Temperature [K]	180.0	293(2)	175(2)	150.0	150.0
Crystal system	monoclinic	monoclinic	monoclinic	orthorhombic	monoclinic
Space group	*Cc*	*P*2$_1$/*c*	*P*2$_1$/*n*	*Pbca*	*P*2$_1$/*n*
a [Å]	8.2381(3)	9.0642(18)	8.0285(9)	12.8016(2)	8.38227(8)
b [Å]	9.5261(3)	14.727(3)	6.7948(14)	6.90606(13)	20.77412(18)
c [Å]	12.4261(4)	7.907(3)	18.1335(19)	26.6508(4)	8.40069(6)
α [°]	90	90	90	90	90
β [°]	92.633(3)	92.62(3)	90.415(9)	90	97.0823(8)
γ [°]	90	90	90	90	90
Volume [Å^3]	974.13(6)	1054.3(5)	989.2(3)	2356.16(7)	1451.69(2)
Z	4	4	4	8	4
ρ_{calc} [g/cm^3]	1.903	1.758	1.874	1.636	1.644
μ [mm^{-1}]	25.543	0.000	0.000	4.786	2.690
F(000)	536.0	170.0	170.0	1168.0	736.0
Crystal size [mm^3]	0.31 × 0.24 × 0.21	– *	– *	0.102 × 0.099 × 0.067	0.159 × 0.134 × 0.102
Radiation type [Å]	Cu Kα (λ = 1.54184)	electron (λ = 0.0251)	electron (λ = 0.0251)	Cu Kα (λ = 1.54184)	Cu Kα (λ = 1.54184)
2Θ range for data collection [°]	14.222 to 129.858	0.158 to 1.798	0.196 to 1.798	6.634 to 158.48	8.512 to 160.206
Index ranges	-9 ≤ h ≤ 9, -11 ≤ k ≤ 11, -14 ≤ l ≤ 14	-11 ≤ h ≤ 11, -18 ≤ k ≤ 18, -9 ≤ l ≤ 9	-10 ≤ h ≤ 10, -8 ≤ k ≤ 8, -22 ≤ l ≤ 22	-16 ≤ h ≤ 12, -7 ≤ k ≤ 8, -30 ≤ l ≤ 33	-10 ≤ h ≤ 10, -26 ≤ k ≤ 26, -7 ≤ l ≤ 10
Reflections collected	6187	12983	12114	9419	24084
Independent reflections	1537 [R_{int} = 0.0771, R_{sigma} = 0.0507]	2151 [R_{int} = 0.2894, R_{sigma} = 0.2112]	2023 [R_{int} = 0.2056, R_{sigma} = 0.1260]	2499 [R_{int} = 0.0312, R_{sigma} = 0.0301]	3118 [R_{int} = 0.0350, R_{sigma} = 0.0195]
Data/restraints/parameters	1537/2/111	2151/87/112	2023/0/112	2499/0/148	3118/0/212
Goodness-of-fit on F^2 (a)	1.054	1.290	1.127	1.047	1.077
Final R indexes [I>=2σ (I)] (b)	R_1 = 0.0703, wR_2 = 0.1707	R_1 = 0.2006, wR_2 = 0.4913	R_1 = 0.1692, wR_2 = 0.4059	R_1 = 0.0358, wR_2 = 0.0987	R_1 = 0.0311, wR_2 = 0.0838
Final R indexes [all data] (b)	R_1 = 0.0706, wR_2 = 0.1712	R_1 = 0.2835, wR_2 = 0.5500	R_1 = 0.2030, wR_2 = 0.4263	R_1 = 0.0388, wR_2 = 0.1017	R_1 = 0.0325, wR_2 = 0.0850
Largest diff. peak/hole [e/Å^{-3}]	2.77/-2.22	0.19/-0.20	0.27/-0.19	1.44/-0.61	0.38/-0.33
Flack parameter	-0.01(3)	–	–	–	–

(a) GooF = S = $[[\sum w(F_o^2-F_c^2)^2]/(m-n)]^{1/2}$, where m = number of reflexes and n = number of parameters; (b) $R_1 = \sum||F_o|-|F_c||/\sum|F_o|$; $wR_2 = [\sum[w(F_o^2-F_c^2)^2/\sum[(wF_o^2)^2]]^{1/2}$; $w = 1/[\sigma^2(F_o^2)+(aP)^2+bP]$, where $P = (F_o^2+2F_c^2)$; * measured using ED (Electrons feature very strong interactions with the electrostatic potential of atoms. Subsequently, electron diffraction allows for performing experiments with crystallites in the nanometer range. However, it needs to be considered that the absorption of the samples is much stronger and the data are affected by dynamical diffraction as well as ionic scattering factors compared to X-ray diffraction. This can lead to seemingly bad R-values for the refinement in the simplistic kinematic approximation.)

Table S4. Crystal data and structure refinement for **1** at 100, 150, 200 and 270 K.

Identification code	**1 (100)**	**1 (150)**	**1 (200)**	**1 (270)**	**1 (rt – 90)***
CCDC number	2551211	2551212	2551213	2551214	–
Empirical formula	$C_{10}H_{12}INO_4$	$C_{10}H_{12}INO_4$	$C_{10}H_{12}INO_4$	$C_{10}H_{12}INO_4$	$C_{10}H_{12}INO_4$
Formula weight	337.11	337.11	337.11	337.11	337.11
Temperature [K]	100.0	150.0	200.0	270.0	90.0
Crystal system	monoclinic	monoclinic	monoclinic	monoclinic	orthorhombic
Space group	$P2_1/c$	$P2_1/c$	$P2_1/c$	$I2/a$	$Pnma$
a [Å]<br>b [Å]<br>c [Å]<br>α [°]<br>β [°]<br>γ [°]	14.1606(4)<br>12.2964(3)<br>7.0159(2)<br>90<br>92.755(3)<br>90	14.1845(4)<br>12.3382(3)<br>7.0346(2)<br>90<br>92.944(2)<br>90	14.21080(19)<br>12.42281(16)<br>7.06614(12)<br>90<br>93.6919(13)<br>90	14.1937(2)<br>12.6616(2)<br>7.13370(10)<br>90<br>97.2530(10)<br>90	14.186(2)<br>12.2833(15)<br>6.9920(11)<br>90<br>90<br>90
Volume [$Å^3$]	1220.23(6)	1229.51(6)	1244.85(3)	1271.77(3)	1218.4(3)
Z	4	4	4	4	4
ρ_{calc} [g/cm^3]	1.835	1.821	1.799	1.761	1.838
μ [mm^{-1}]	20.651	20.495	20.242	19.814	20.681
F(000)	656.0	656.0	656.0	656.0	656.0
Crystal size [mm^3]	0.06 × 0.03 × 0.03	0.06 × 0.03 × 0.03	0.06 × 0.03 × 0.03	0.06 × 0.03 × 0.03	0.03 × 0.023 × 0.12
Radiation type [Å]	Cu Kα (λ = 1.54184)	Cu Kα (λ = 1.54184)	Cu Kα (λ = 1.54184)	Cu Kα (λ = 1.54184)	Cu Kα (λ = 1.54184)
2Θ range for data collection [°]	6.248 to 134.992	9.506 to 134.96	6.232 to 159.512	9.394 to 160.02	9.524 to 175.268
Index ranges	-16 ≤ h ≤ 16, -14 ≤ k ≤ 14, -7 ≤ l ≤ 8	-16 ≤ h ≤ 16, -14 ≤ k ≤ 14, -8 ≤ l ≤ 6	-17 ≤ h ≤ 17, -15 ≤ k ≤ 15, -8 ≤ l ≤ 8	-7 ≤ h ≤ 8, -16 ≤ k ≤ 15, -18 ≤ l ≤ 18	-7 ≤ h ≤ 8, -17 ≤ k ≤ 17, -14 ≤ l ≤ 15
Reflections collected	14400	14383	45990	9278	4559
Independent reflections	2203 [R_{int} = 0.0755, R_{sigma} = 0.0377]	2201 [R_{int} = 0.0751, R_{sigma} = 0.0405]	2639 [R_{int} = 0.0997, R_{sigma} = 0.0290]	1366 [R_{int} = 0.1026, R_{sigma} = 0.0542]	1337 [R_{int} = 0.1753, R_{sigma} = 0.0965]
Data/restraints/parameters	2203/0/148	2201/0/148	2639/0/149	1366/0/78	1337/0/39
Goodness-of-fit on F^2 [(a)]	1.050	1.031	1.113	1.115	–
Final R indexes [I>=2σ (I)] [(b)]	R_1 = 0.0409, wR_2 = 0.1078	R_1 = 0.0382, wR_2 = 0.1011	R_1 = 0.0318, wR_2 = 0.0886	R_1 = 0.0458, wR_2 = 0.1042	–
Final R indexes [all data] [(b)]	R_1 = 0.0429, wR_2 = 0.1099	R_1 = 0.0402, wR_2 = 0.1033	R_1 = 0.0332, wR_2 = 0.0897	R_1 = 0.0462, wR_2 = 0.1049	–
Largest diff. peak/hole [$e/Å^{-3}$]	2.30/-1.88	2.17/-1.77	0.85/-1.22	1.23/-0.93	–

[(a)] GooF = S = $[[\sum w(F_o^2-F_c^2)^2]/(m-n)]^{1/2}$, where m = number of reflexes and n = number of parameters; [(b)] $R_1 = \sum||F_o|-|F_c||/\sum|F_o|$; $wR_2 = [\sum[w(F_o^2-F_c^2)^2/\sum[(wF_o^2)^2]]^{1/2}$; $w = 1/[\sigma^2(F_o^2)+(aP)^2+bP]$, where $P = (F_o^2+2F_c^2)$. * – while VT measurements were conducted starting from RT by slow cooling to 100 K(approx. 60 K/h rate), this sample was subjected to shock freezing from room temperature to 100 K, the collected data matches CSD Entry (CONQIH, 983186).

Table S5. Crystal data and structure refinement for **2** at 100, 150, 200 and 250 K.

Identification code	**2 (100)**	**2 (150)**	**2 (200)**	**2 (250)**
CCDC number	2551215	2551216	2551217	2551218
Empirical formula	$C_{11}H_{14}INO_4$	$C_{11}H_{14}INO_4$	$C_{11}H_{14}INO_4$	$C_{11}H_{14}INO_4$
Formula weight	351.13	351.13	351.13	351.13
Temperature [K]	100.0	150.0	200.0	250.0
Crystal system	monoclinic	monoclinic	monoclinic	monoclinic
Space group	*C*2/*c*	*C*2/*c*	*C*2/*c*	*C*2/*c*
a [Å]<br>b [Å]<br>c [Å]<br>α [°]<br>β [°]<br>γ [°]	19.1060(3)<br>7.30700(10)<br>18.9617(3)<br>90<br>91.851(2)<br>90	19.1269(3)<br>7.33010(10)<br>18.9929(3)<br>90<br>91.9630(10)<br>90	19.1574(3)<br>7.35660(10)<br>19.0281(3)<br>90<br>92.0850(10)<br>90	19.1920(3)<br>7.38470(10)<br>19.0647(3)<br>90<br>92.2000(10)<br>90
Volume [Å^3]	2645.81(7)	2661.28(7)	2679.92(7)	2699.99(7)
Z	8	8	8	8
ρ_{calc} [g/cm^3]	1.763	1.753	1.741	1.728
μ [mm^{-1}]	19.075	18.964	18.833	18.693
F(000)	1376.0	1376.0	1376.0	1376.0
Crystal size [mm^3]	0.181 × 0.154 × 0.118	0.181 × 0.154 × 0.118	0.181 × 0.154 × 0.118	0.181 × 0.154 × 0.118
Radiation type [Å]	Cu Kα (λ = 1.54184)	Cu Kα (λ = 1.54184)	Cu Kα (λ = 1.54184)	Cu Kα (λ = 1.54184)
2Θ range for data collection [°]	9.262 to 129.986	9.252 to 160.166	9.238 to 159.53	9.222 to 160.094
Index ranges	-22 ≤ h ≤ 22, -8 ≤ k ≤ 8, -22 ≤ l ≤ 22	-24 ≤ h ≤ 24, -8 ≤ k ≤ 9, -24 ≤ l ≤ 23	-24 ≤ h ≤ 24, -8 ≤ k ≤ 9, -24 ≤ l ≤ 23	-24 ≤ h ≤ 20, -8 ≤ k ≤ 9, -24 ≤ l ≤ 24
Reflections collected	9557	11451	11130	11080
Independent reflections	2243 [R_{int} = 0.0509, R_{sigma} = 0.0385]	2837 [R_{int} = 0.0523, R_{sigma} = 0.0405]	2855 [R_{int} = 0.0440, R_{sigma} = 0.0354]	2853 [R_{int} = 0.0470, R_{sigma} = 0.0374]
Data/restraints/parameters	2243/0/159	2837/0/157	2855/0/159	2853/0/157
Goodness-of-fit on F^2 [(a)]	1.050	1.037	1.041	1.052
Final R indexes [I>=2σ (I)] [(b)]	R_1 = 0.0314, wR_2 = 0.0803	R_1 = 0.0381, wR_2 = 0.0998	R_1 = 0.0363, wR_2 = 0.0956	R_1 = 0.0369, wR_2 = 0.0957
Final R indexes [all data] [(b)]	R_1 = 0.0330, wR_2 = 0.0819	R_1 = 0.0398, wR_2 = 0.1016	R_1 = 0.0384, wR_2 = 0.0974	R_1 = 0.0406, wR_2 = 0.0985
Largest diff. peak/hole [e/Å^{-3}]	0.21/-0.11	1.99/-1.88	0.08/-0.04	1.68/-1.05

[(a)] GooF = S = $[[\sum w(F_o^2-F_c^2)^2]/(m-n)]^{1/2}$, where m = number of reflexes and n = number of parameters; [(b)] $R_1 = \sum||F_o|-|F_c||/\sum|F_o|$; $wR_2 = [\sum[w(F_o^2-F_c^2)^2/\sum[(wF_o^2)^2]]^{1/2}$; $w = 1/[\sigma^2(F_o^2)+(aP)^2+bP]$, where $P = (F_o^2+2F_c^2)$

Table S6. Crystal data and structure refinement for **3** at 100, 150, 200 and 250 K.

Identification code	**3 (100)**	**3 (150)**	**3 (200)**	**3 (250)**
CCDC number	2551219	2551220	2551221	2551222
Empirical formula	$C_{12}H_{16}INO_4$	$C_{12}H_{16}INO_4$	$C_{12}H_{16}INO_4$	$C_{12}H_{16}INO_4$
Formula weight	365.16	365.16	365.16	365.16
Temperature [K]	100.0	150.0	200.0	250.0
Crystal system	monoclinic	monoclinic	monoclinic	monoclinic
Space group	$P2_1/n$	$P2_1/n$	$P2_1/n$	$P2_1/n$
a [Å]<br>b [Å]<br>c [Å]<br>α [°]<br>β [°]<br>γ [°]	8.49590(10)<br>17.0089(2)<br>9.61940(10)<br>90<br>90.9530(10)<br>90	8.49200(10)<br>17.0591(2)<br>9.66690(10)<br>90<br>90.9420(10)<br>90	8.49060(10)<br>17.1250(2)<br>9.72340(10)<br>90<br>90.9090(10)<br>90	8.48570(10)<br>17.1982(2)<br>9.78320(10)<br>90<br>90.8700(10)<br>90
Volume [Å^3]	1389.87(3)	1400.21(3)	1413.62(3)	1427.58(3)
Z	4	4	4	4
ρ_{calc} [g/cm^3]	1.745	1.732	1.716	1.699
μ [mm^{-1}]	18.183	18.048	17.877	17.702
F(000)	720.0	720.0	720.0	720.0
Crystal size [mm^3]	0.05 × 0.032 × 0.011	0.05 × 0.032 × 0.011	0.05 × 0.032 × 0.011	0.05 × 0.032 × 0.011
Radiation type [Å]	Cu Kα (λ = 1.54184)	Cu Kα (λ = 1.54184)	Cu Kα (λ = 1.54184)	Cu Kα (λ = 1.54184)
2Θ range for data collection [°]	10.402 to 159.416	10.372 to 159.104	10.332 to 159.368	10.288 to 160.008
Index ranges	-10 ≤ h ≤ 8, -21 ≤ k ≤ 21, -12 ≤ l ≤ 12	-10 ≤ h ≤ 10, -21 ≤ k ≤ 21, -9 ≤ l ≤ 12	-10 ≤ h ≤ 9, -21 ≤ k ≤ 21, -11 ≤ l ≤ 12	-10 ≤ h ≤ 10, -21 ≤ k ≤ 21, -10 ≤ l ≤ 12
Reflections collected	10181	10958	11134	10967
Independent reflections	2880 [R_{int} = 0.0382, R_{sigma} = 0.0275]	2919 [R_{int} = 0.0391, R_{sigma} = 0.0280]	3001 [R_{int} = 0.0423, R_{sigma} = 0.0333]	3023 [R_{int} = 0.0469, R_{sigma} = 0.0374]
Data/restraints/parameters	2880/0/166	2919/0/166	3001/0/166	3023/0/166
Goodness-of-fit on F^2 (a)	1.092	1.086	1.044	1.025
Final R indexes [I>=2σ (I)] (b)	R_1 = 0.0315, wR_2 = 0.0861	R_1 = 0.0284, wR_2 = 0.0773	R_1 = 0.0338, wR_2 = 0.0927	R_1 = 0.0454, wR_2 = 0.1311
Final R indexes [all data] (b)	R_1 = 0.0321, wR_2 = 0.0866	R_1 = 0.0291, wR_2 = 0.0780	R_1 = 0.0349, wR_2 = 0.0938	R_1 = 0.0471, wR_2 = 0.1334
Largest diff. peak/hole [e/Å^{-3}]	1.11/-1.20	1.12/-0.79	1.78/-1.42	2.03/-1.73

(a) GooF = S = $[[\sum w(F_o^2-F_c^2)^2]/(m-n)]^{1/2}$, where m = number of reflexes and n = number of parameters; (b) $R_1 = \sum||F_o|-|F_c||/\sum|F_o|$; $wR_2 = [\sum[w(F_o^2-F_c^2)^2/\sum[(wF_o^2)^2]]^{1/2}$; $w = 1/[\sigma^2(F_o^2)+(aP)^2+bP]$, where $P = (F_o^2+2F_c^2)$

Table S7. Crystal data and structure refinement for **4** at 100, 150, 200, 250 and 290 K.

Identification code	**4 (100)**	**4 (150)**	**4 (200)**	**4 (250)**	**4 (290)**
CCDC number	2551223	2551224	2551225	2551226	2551227
Empirical formula	$C_{12}H_{16}INO_4$	$C_{12}H_{16}INO_4$	$C_{12}H_{16}INO_4$	$C_{12}H_{16}INO_4$	$C_{12}H_{16}INO_4$
Formula weight	365.16	365.16	365.16	365.16	365.16
Temperature [K]	100.0	150.0	200.0	250.0	290.0
Crystal system	monoclinic	monoclinic	monoclinic	monoclinic	monoclinic
Space group	$P2_1/c$	$P2_1/c$	$P2_1/c$	$P2_1/c$	$P2_1/c$
a [Å]<br>b [Å]<br>c [Å]<br>α [°]<br>β [°]<br>γ [°]	7.75090(10)<br>10.65210(10)<br>17.0506(2)<br>90<br>90.0880(10)<br>90	7.75900(10)<br>10.68830(10)<br>17.0912(2)<br>90<br>90.1340(10)<br>90	7.77090(10)<br>10.73470(10)<br>17.1339(3)<br>90<br>90.210(2)<br>90	7.78630(10)<br>10.7745(2)<br>17.1736(3)<br>90<br>90.312(2)<br>90	7.8048(2)<br>10.8186(2)<br>17.2076(4)<br>90<br>90.478(2)<br>90
Volume [Å^3]	1407.75(3)	1417.38(3)	1429.27(3)	1440.73(4)	1452.91(6)
Z	4	4	4	4	4
ρ_{calc} [g/cm^3]	1.723	1.711	1.697	1.683	1.669
μ [mm^{-1}]	17.952	17.830	17.681	17.541	17.394
F(000)	720.0	720.0	720.0	720.0	720.0
Crystal size [mm^3]	0.13 × 0.091 × 0.087	0.13 × 0.091 × 0.087	0.13 × 0.091 × 0.087	0.13 × 0.091 × 0.087	0.13 × 0.091 × 0.087
Radiation type [Å]	Cu Kα (λ = 1.54184)	Cu Kα (λ = 1.54184)	Cu Kα (λ = 1.54184)	Cu Kα (λ = 1.54184)	Cu Kα (λ = 1.54184)
2Θ range for data collection [°]	9.79 to 159.62	9.76 to 159.918	9.722 to 160.116	9.69 to 160.502	9.656 to 160.146
Index ranges	-9 ≤ h ≤ 9, -13 ≤ k ≤ 11, -21 ≤ l ≤ 21	-9 ≤ h ≤ 9, -13 ≤ k ≤ 9, -21 ≤ l ≤ 21	-9 ≤ h ≤ 9, -13 ≤ k ≤ 8, -21 ≤ l ≤ 21	-9 ≤ h ≤ 9, -13 ≤ k ≤ 9, -21 ≤ l ≤ 21	-9 ≤ h ≤ 9, -13 ≤ k ≤ 9, -20 ≤ l ≤ 21
Reflections collected	11061	15317	11065	11428	11352
Independent reflections	2986 [R_{int} = 0.0429, R_{sigma} = 0.0330]	3011 [R_{int} = 0.0453, R_{sigma} = 0.0277]	2925 [R_{int} = 0.0448, R_{sigma} = 0.0337]	3051 [R_{int} = 0.0405, R_{sigma} = 0.0325]	3059 [R_{int} = 0.0395, R_{sigma} = 0.0378]
Data/restraints/parameters	2986/0/167	3011/0/167	2925/0/167	3051/0/167	3059/0/167
Goodness-of-fit on F^2 [(a)]	1.080	1.080	1.095	1.089	1.083
Final R indexes [I>=2σ (I)] [(b)]	R_1 = 0.0339, wR_2 = 0.0866	R_1 = 0.0326, wR_2 = 0.0862	R_1 = 0.0351, wR_2 = 0.0955	R_1 = 0.0359, wR_2 = 0.0932	R_1 = 0.0377, wR_2 = 0.1000
Final R indexes [all data] [(b)]	R_1 = 0.0347, wR_2 = 0.0873	R_1 = 0.0333, wR_2 = 0.0868	R_1 = 0.0370, wR_2 = 0.0970	R_1 = 0.0382, wR_2 = 0.0950	R_1 = 0.0409, wR_2 = 0.1022
Largest diff. peak/hole [e/Å^{-3}]	0.88/-1.32	1.12/-0.97	0.88/-1.07	0.83/-1.03	1.04/-0.79

[(a)] GooF = S = $[[\sum w(F_o^2-F_c^2)^2]/(m-n)]^{1/2}$, where m = number of reflexes and n = number of parameters; [(b)] $R_1 = \sum||F_o|-|F_c||/\sum|F_o|$; $wR_2 = [\sum[w(F_o^2-F_c^2)^2/\sum[(wF_o^2)^2]]^{1/2}$; $w = 1/[\sigma^2(F_o^2)+(aP)^2+bP]$, where $P = (F_o^2+2F_c^2)$

Table S8. Crystal data and structure refinement for **4^A**, **4(I3)** and **⁻5**.

Identification code	**4^A**	**4 (I3)**	**⁻5**	**1^BARF**
CCDC number	2551228	2551229	2551230	2551231
Empirical formula	$C_{26}H_{35}I_2N_3O_8$	$C_{12}H_{16}I_2NO_4$	$C_{63}H_{96}I_2N_2O_8$	$C_{42}H_{24}BF_{24}NO_4$
Formula weight	771.37	492.06	1261.20	1073.43
Temperature [K]	150.0	150.0	150.0	150 .0
Crystal system	triclinic	monoclinic	monoclinic	triclinic
Space group	*P*-1	*I*2/*a*	*P*2$_1$	*P*-1
a [Å]<br>b [Å]<br>c [Å]<br>α [°]<br>β [°]<br>γ [°]	11.2448(2)<br>12.0785(2)<br>12.7698(2)<br>86.1510(10)<br>70.0990(10)<br>74.720(2)	9.38260(10)<br>18.2576(2)<br>19.9098(2)<br>90<br>98.8520(10)<br>90	14.3672(7)<br>7.4633(3)<br>32.9661(19)<br>90<br>92.794(5)<br>90	13.6966(2)<br>13.7552(2)<br>15.8743(2)<br>66.6650(10)<br>82.7850(10)<br>65.277(2)
Volume [Å^3]	1572.69(5)	3370.00(6)	3530.6(3)	2491.50(7)
Z	2	8	2	2
ρ_{calc} [g/cm^3]	1.629	1.940	1.186	1.431
μ [mm^{-1}]	16.114	29.407	7.359	1.379
F(000)	764.0	1864.0	1312.0	1072.0
Crystal size [mm^3]	0.232 × 0.153 × 0.121	0.21 × 0.079 × 0.056	0.102 × 0.054 × 0.027	0.326 × 0.232 × 0.187
Radiation type [Å]	Cu Kα (λ = 1.54184)	Cu Kα (λ = 1.54184)	Cu Kα (λ = 1.54184)	Cu Kα (λ = 1.54184)
2Θ range for data collection [°]	7.364 to 129.986	6.604 to 159.218	5.368 to 159.412	6.07 to 144.984
Index ranges	-13 ≤ h ≤ 12, -14 ≤ k ≤ 12, -14 ≤ l ≤ 14	-11 ≤ h ≤ 8, -22 ≤ k ≤ 22, -25 ≤ l ≤ 25	-18 ≤ h ≤ 13, -3 ≤ k ≤ 9, -41 ≤ l ≤ 40	-16 ≤ h ≤ 16, -17 ≤ k ≤ 17, -19 ≤ l ≤ 19
Reflections collected	19717	13558	20684	39001
Independent reflections	5355 [R_{int} = 0.0622, R_{sigma} = 0.0586]	3574 [R_{int} = 0.0822, R_{sigma} = 0.0591]	9894 [R_{int} = 0.1057, R_{sigma} = 0.1096]	9828 [Rint = 0.0317, Rsigma = 0.0320]
Data/restraints/parameters	5355/0/361	3574/0/171	9894/13/691	9828/183/706
Goodness-of-fit on F^2 (a)	1.030	1.042	1.012	1.066
Final R indexes [I>=2σ (I)] (b)	R_1 = 0.0464, wR_2 = 0.1175	R_1 = 0.0510, wR_2 = 0.1360	R_1 = 0.0919, wR_2 = 0.2408	R1 = 0.0846, wR2 = 0.2419
Final R indexes [all data] (b)	R_1 = 0.0481, wR_2 = 0.1195	R_1 = 0.0524, wR_2 = 0.1376	R_1 = 0.1409, wR_2 = 0.2947	R1 = 0.0911, wR2 = 0.2486
Largest diff. peak/hole [e/Å^{-3}]	1.45/-2.00	1.87/-2.46	1.88/-2.40	0.74/-0.65
Flack parameter	–	–	0.006(15)	–

(a) GooF = S = $[[\sum w(F_o^2-F_c^2)^2]/(m-n)]^{1/2}$, where m = number of reflexes and n = number of parameters; (b) $R_1 = \sum||F_o|-|F_c||/\sum|F_o|$; $wR_2 = [\sum[w(F_o^2-F_c^2)^2/\sum[(wF_o^2)^2]]^{1/2}$; $w = 1/[\sigma^2(F_o^2)+(aP)^2+bP]$, where $P = (F_o^2+2F_c^2)$

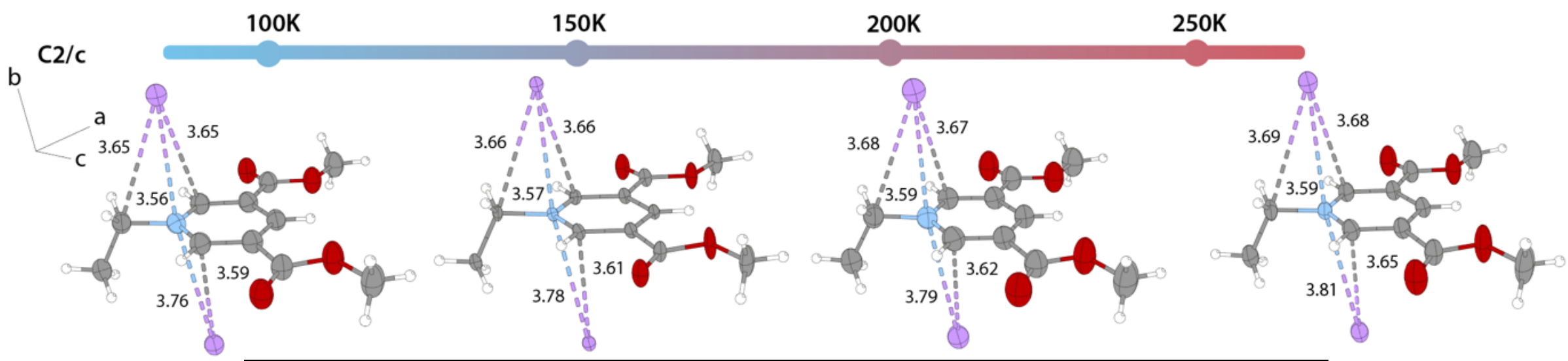

T, K	N1···I1, Å	C1···I1, Å	C5/C4···I1, Å
100	3.559 / 3.767	3.644	3.580 / 3.765
150	3.570 / 3.779	3.661	3.607 / 3.781
200	3.586 / 3.799	3.674	3.618 / 3.801
250	3.594 / 3.810	3.684	3.646 / 3.827

Figure S13. Molecular view of the **2** with selected shortest I···E (E = C or N) anion-π distances (displacement ellipsoids are shown at the 50% probability level). Table with the shortest anion-π distances I···E(E = C or N) in the crystal packing of **2** at variable temperatures.

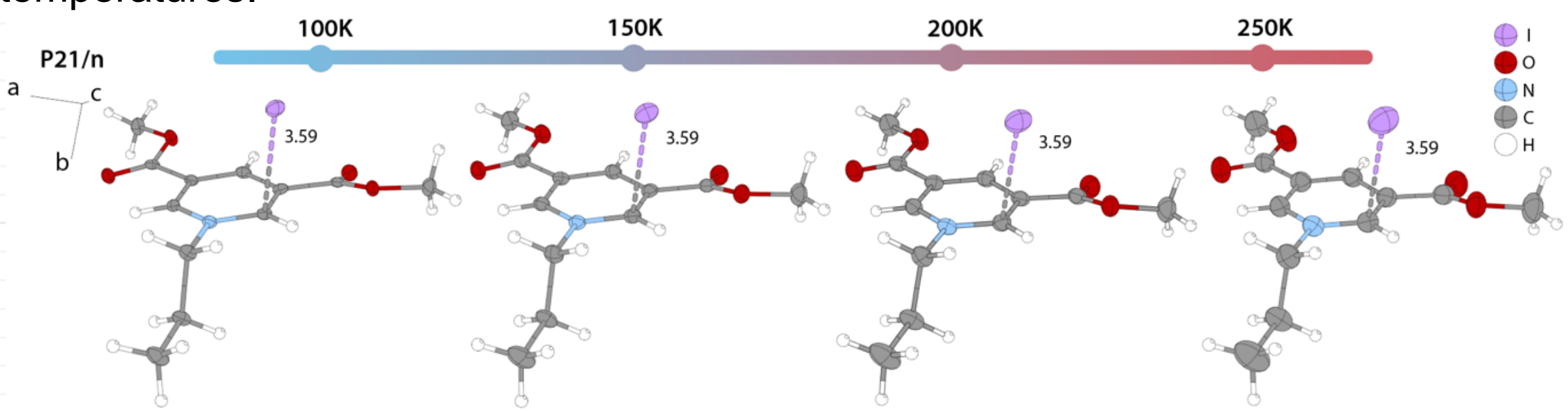

T, K	C1···I1, Å
100	3.586
150	3.588
200	3.588
250	3.593

Figure S14. Molecular view of the **3** with selected shortest I···C anion-π distances (displacement ellipsoids are shown at the 50% probability level). Table with the shortest anion-π distances I···E(E = C or N) in the crystal packing of **3** at variable temperatures.

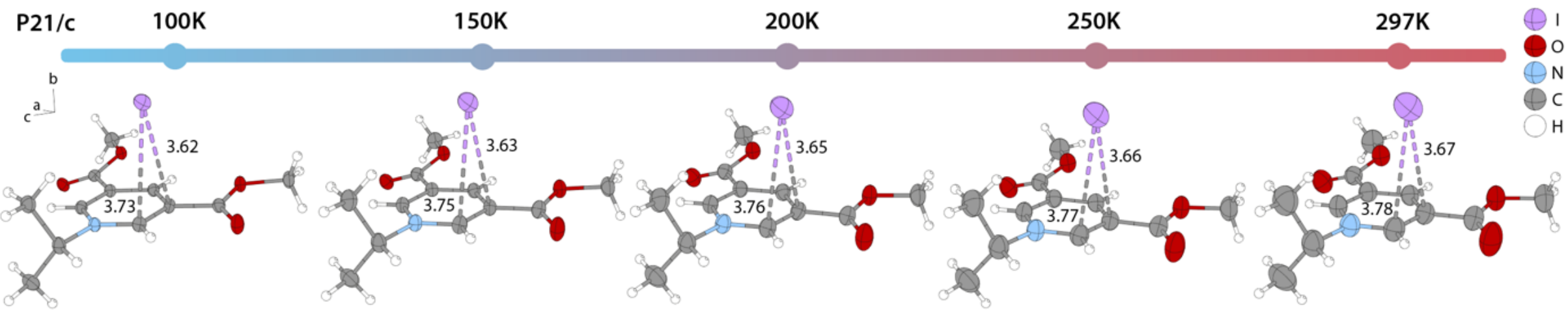

T, K	C1···I1, Å	C2···I1, Å
100	3.622	3.734
150	3.633	3.749
200	3.647	3.759
250	3.656	3.772
297	3.670	3.779

Figure S15. Molecular view of the **4** with selected shortest I···C anion-π distances (displacement ellipsoids are shown at the 50% probability level). Table with the shortest anion-π distances I···E(E = C or N) in the crystal packing of **4** at variable temperatures.

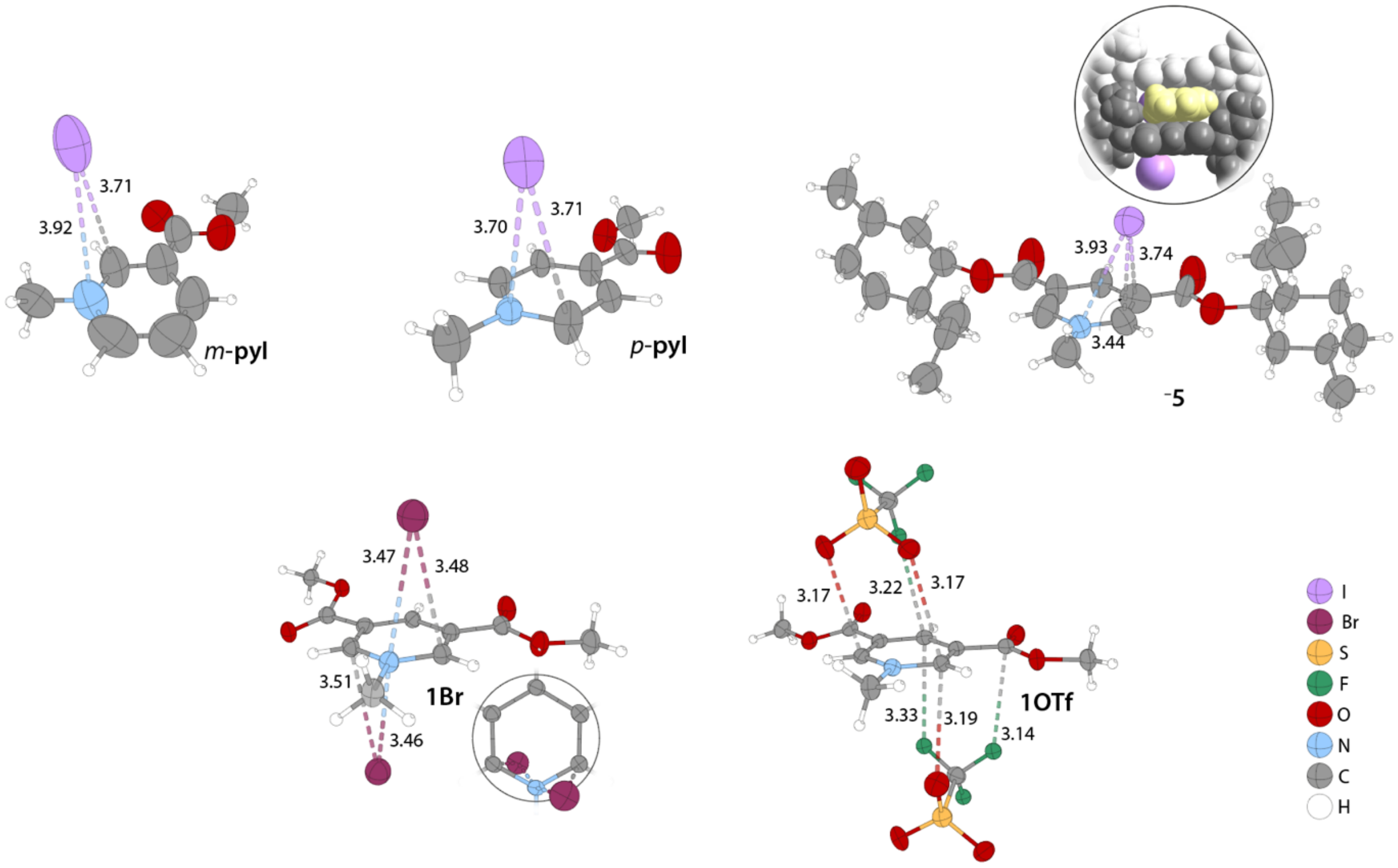

Figure S16. Molecular views of the *m*-**pyl**, *p*-**pyl**, $^{-}$**5** and **1**^{Br}, **1**^{OTf} with selected shortest I···C/N anion-π and I···H distances (displacement ellipsoids are shown at the 50% probability level). A columnar staircase arrangement involving alternating π-stacked toluene-solvated molecules is observed in ion-pair $^{-}$**5**, however, the quality of the data does not allow to confirm depicted shortest anion-π^{+} contacts (3.44/3.74 Å η^2 type (C_1C_2; 95.0% of r_{vdW}[I,C])).

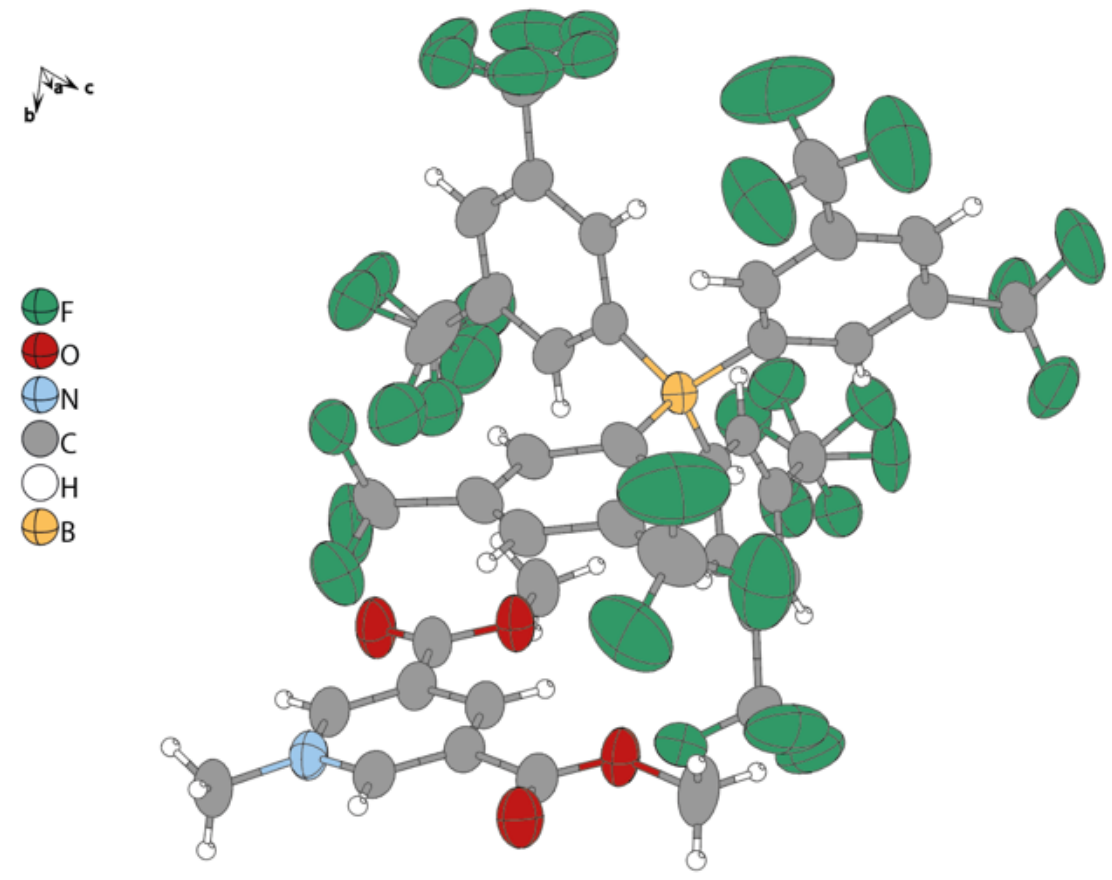

Figure S17. Molecular view of the **1**^{BARF} (displacement ellipsoids are shown at the 50% probability level).

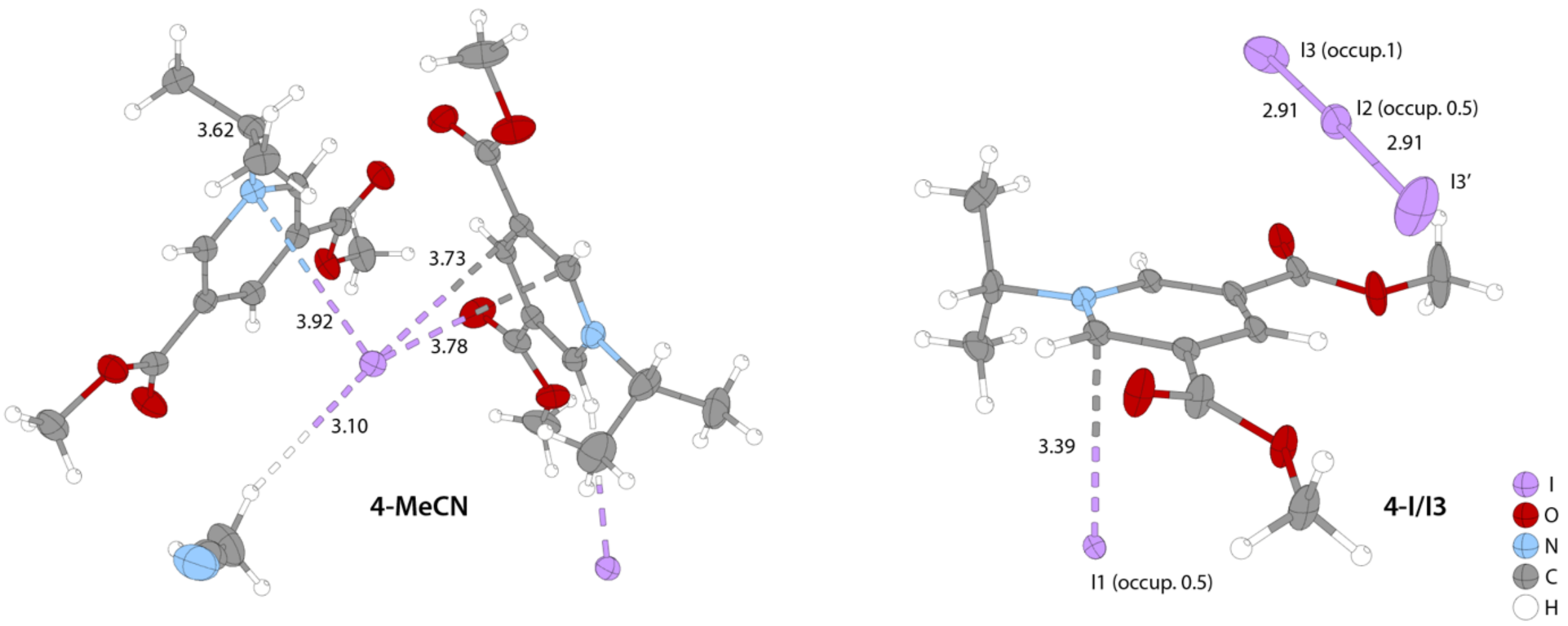

Figure S18. Molecular views of the **4**^{A} **and 4-I/I3** with selected shortest I···C/N anion-π and I···H distances (displacement ellipsoids are shown at the 50% probability level).

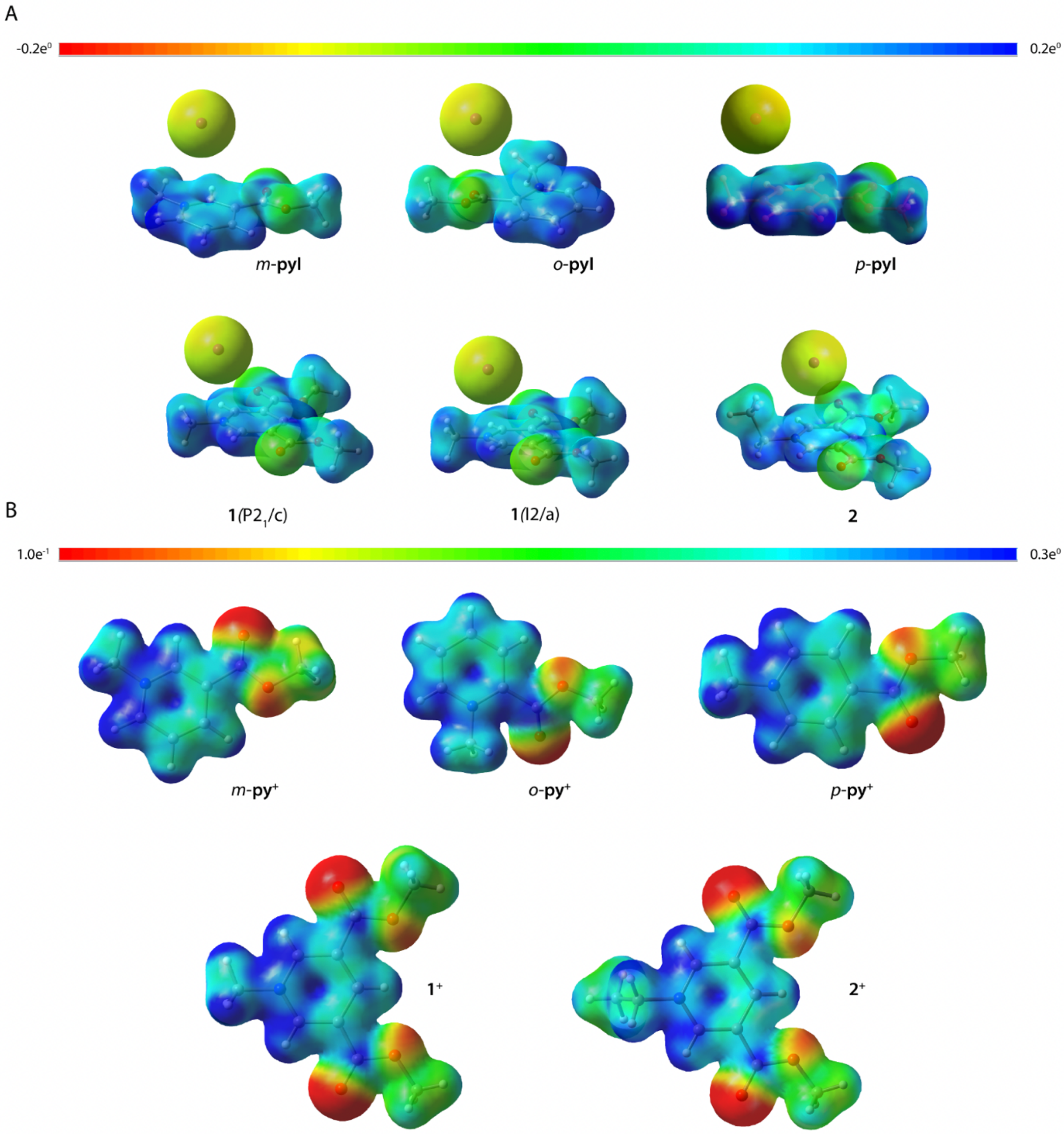

Figure S19. Calculated ESP plots (in a.u.; isovalue = 0.02 a.u.) for ionic pairs (A) *o-*, *m-*, *p*-**pyl, 1** and **2** and their cations (B).

Table S9. Energy decomposition analysis (kcal/mol).

	**1**[a]	**1**[b]	**2**	**1**[a,c]	**1**[b,c]	**2**[c]	o-**pyl**	m-**pyl**	p-**pyl**	p-**pyl**[c]
Orbital	-3.5	-4.8	-4.1	-6.8	-7.7	-6.8	-4.1	-3.0	-3.8	-5.8
Electrostatic	-88.9	-90.8	-89.2	-175.4	-174.3	-171.7	-84.3	-79.9	-86.3	-162.5
Pauli	19.2	21.6	23.4	37.0	37.4	39.0	20.1	12.0	17.1	46.1
CPCM	41.0	40.8	39.2	81.5	80.9	78.9	37.7	39.7	40.8	76.4
**Sum**	**-32.2**	**-33.2**	**-30.7**	**-63.8**	**-63.8**	**-60.6**	**-30.5**	**-31.1**	**-32.1**	**-45.8**

Energy decomposition analysis between cation and anion fragments. Orbital = Energy from orbital mixing, i.e., a measure of covalency. Electrostatic = Coulombic interaction between the two fragments. Pauli = Repulsion due to the Pauli exclusion principle. CPCM = Solvent contribution. [a] Crystallographically determined geometry at 100 K. [b] Crystallographically determined geometry at 270 K. [c] Sandwiched I^- --- Pyr^+ --- I^- model.

Table S10. Summary of Bader's Quantum Theory of Atoms in Molecules (QTAIM) descriptors obtained from computed DFT for the anion–π⁺ interactions in **1**, I^-**–1⁺–**I^-, and *x*-**pyl**, including electron density (ρ), Laplacian of the electron density (∇²ρ), |V|/G ratio, and total energy density (H) at the bond critical points (BCPs).

	**1**			
BCP	ρ [e/Å³]	∇²ρ [e/Å⁵]	|V|/G	H [a.u.]
I - N	6.21E-02	6.66E-01	0.83	1.00E-03
	I^-–**1**–I^-			
BCP	ρ [e/Å³]	∇²ρ [e/Å⁵]	|V|/G	H [a.u.]
I - N	6.35E-02	6.88E-01	0.83	1.02E-03
	6.14E-02	6.67E-01	0.82	1.04E-03
I - H	4.67E-02	4.67E-01	0.81	7.56E-04
	o-**pyl**			
BCP	ρ [e/Å³]	∇²ρ [e/Å⁵]	|V|/G	H [a.u.]
I - C	5.29E-02	4.89E-01	0.88	5.32E-04
I - H	3.62E-02	3.69E-01	0.79	6.59E-04
	4.38E-02	3.82E-01	0.81	6.23E-04
	I^-– *o*-**pyl** –I^-			
BCP	ρ [e/Å³]	∇²ρ [e/Å⁵]	|V|/G	H [a.u.]
I - N	4.82E-02	4.53E-01	0.81	7.40E-04
I - C	5.55E-02	4.93E-01	0.87	5.69E-04
I - H	4.31E-02	3.82E-01	0.81	6.25E-04
	3.59E-02	3.67E-01	0.79	6.54E-04
	m-**pyl**			
BCP	ρ [e/Å³]	∇²ρ [e/Å⁵]	|V|/G	H [a.u.]
I - C	4.89E-02	3.84E-01	0.86	4,83E-04
	p-**pyl**			
BCP	ρ [e/Å³]	∇²ρ [e/Å⁵]	|V|/G	H [a.u.]
I - C	5.02E-02	4.37E-01	0.83	6.54E-04
I - H	5.97E-02	5.13E-01	0.84	7.15E-04

The QTAIM analysis was performed on the optimized geometries described in the Computational Methods section using the same level of theory.

1

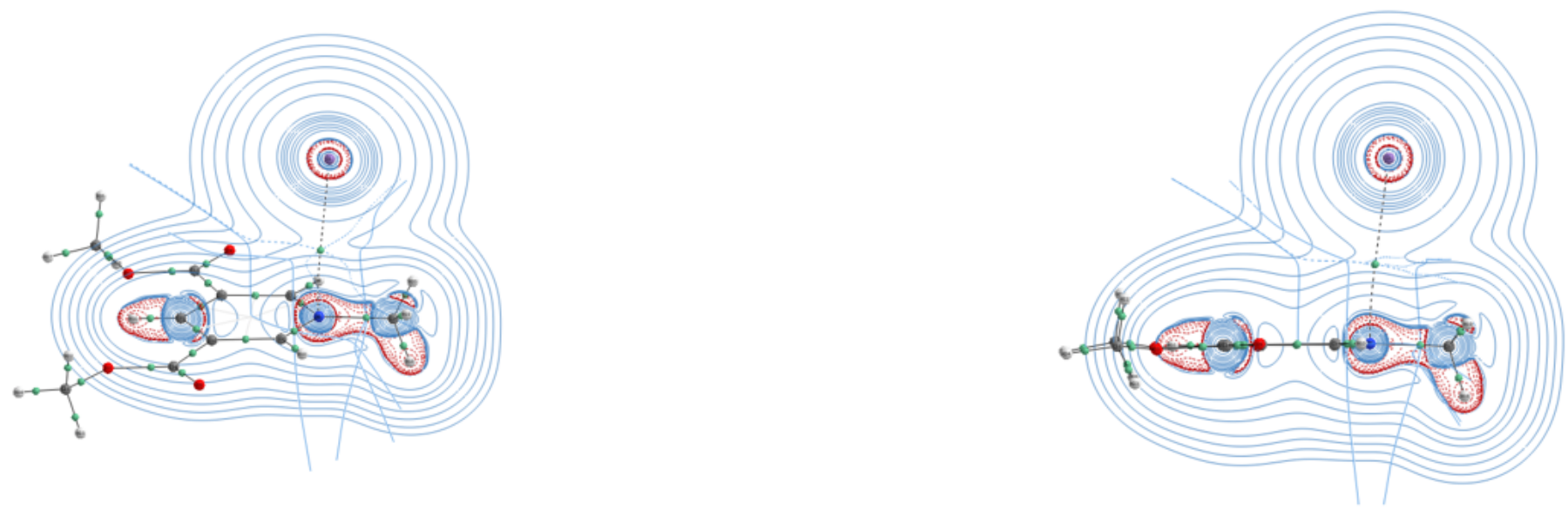

I^{-}–**1**$^{+}$–I^{-}

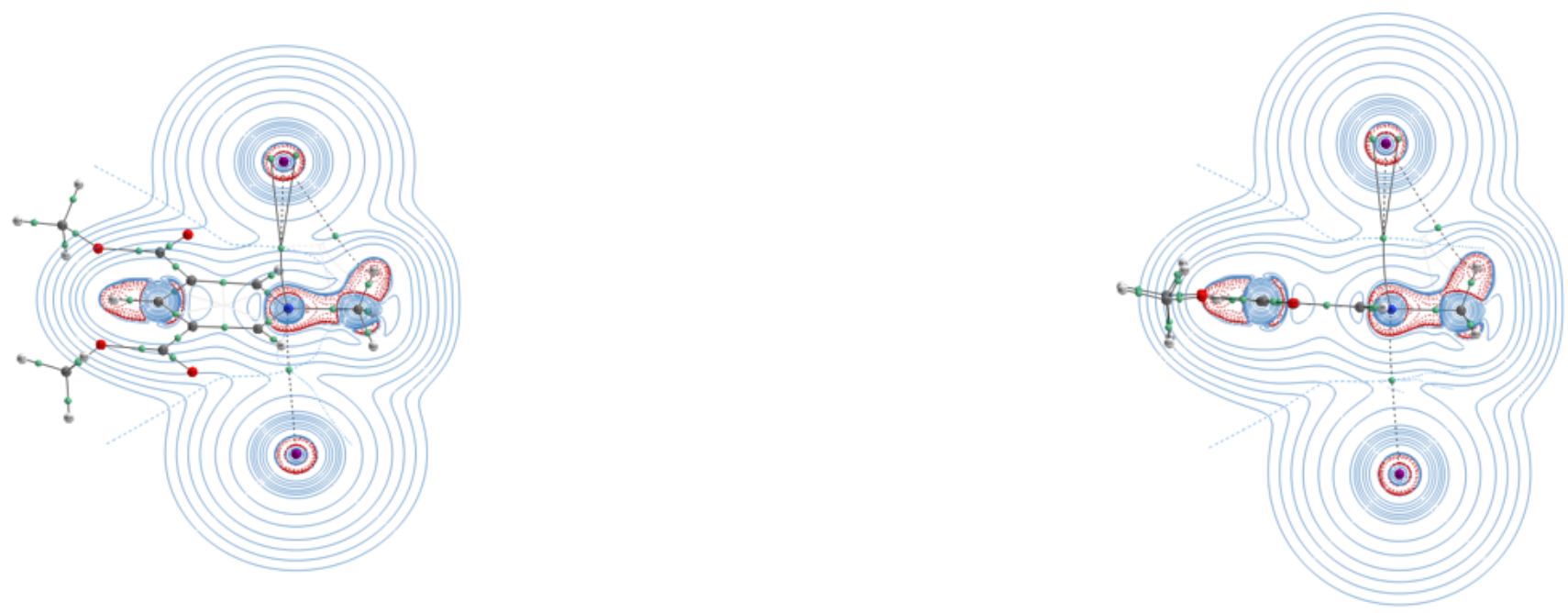

o-**pyI**

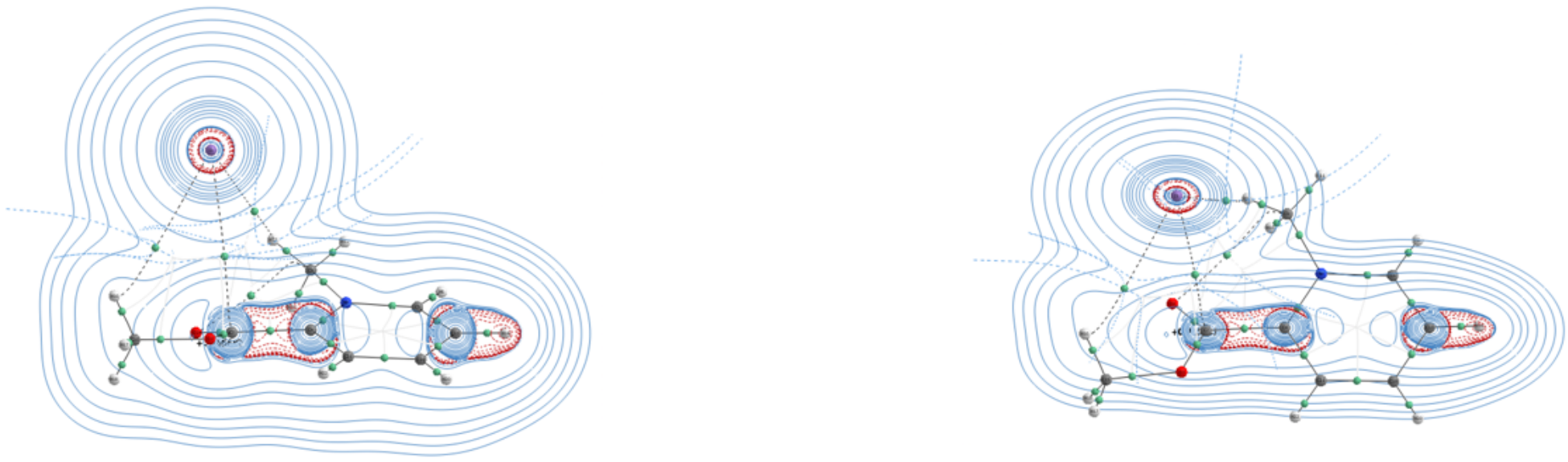

I^{-}– *o*-**py**$^{+}$–I^{-}

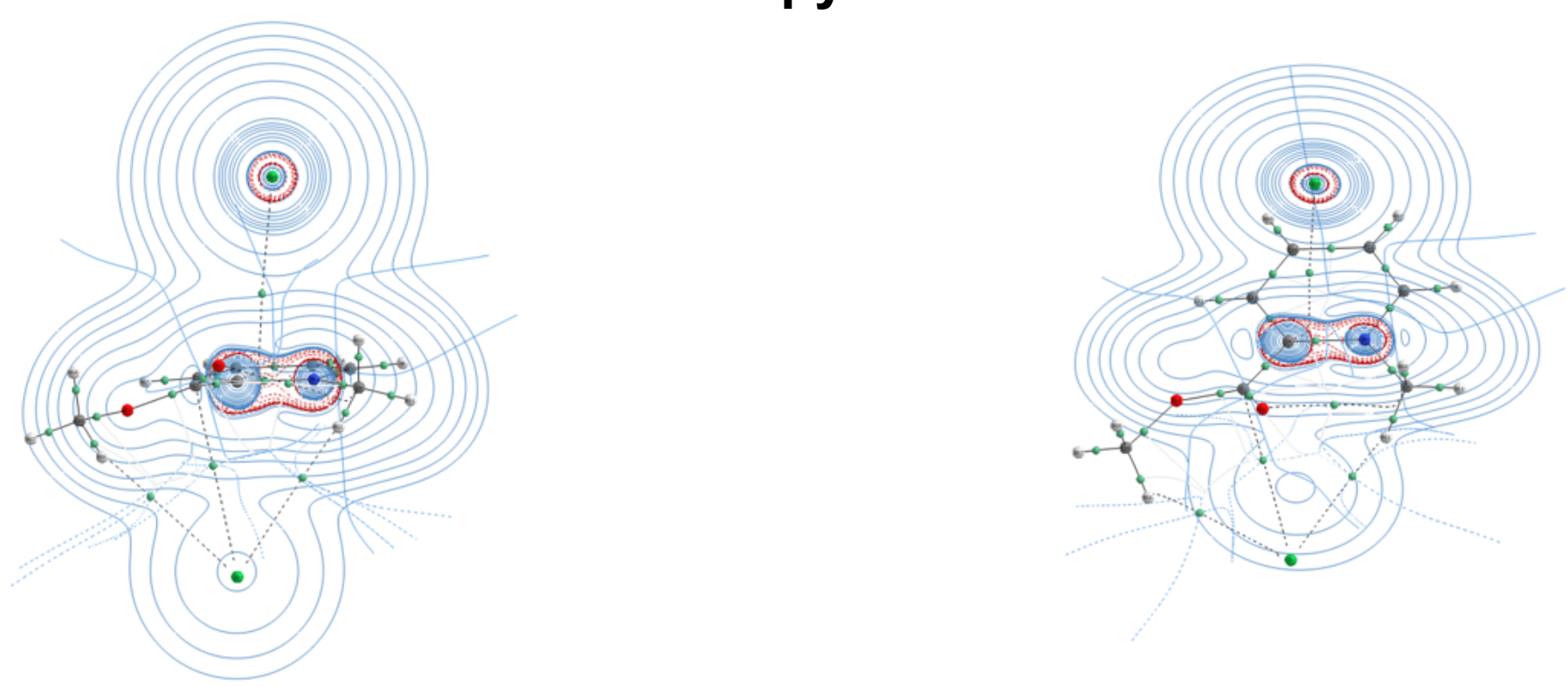

Figure S20. QTAIM analysis of the anion–π^{+} interactions in selected **1**, I^{-}–**1**$^{+}$–I^{-}, *o*-**pyI**, and I^{-}– *o*-**py**$^{+}$–I^{-} Bond paths (black) and bond critical points (green) are overlaid on the sliced calculated electron-density distribution (in plane of anion-π $^{+}$ interactions).

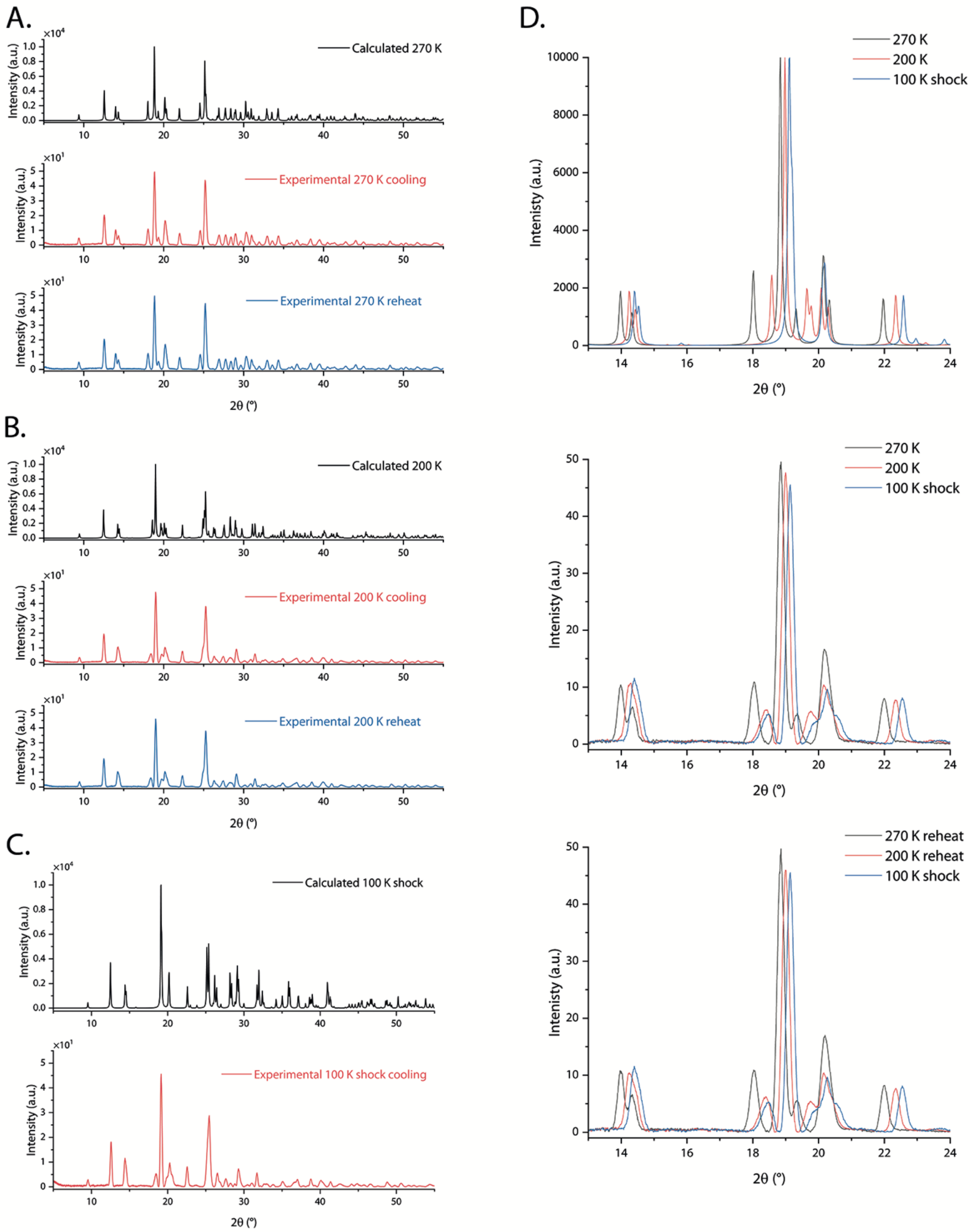

Figure S21. A–C: Cyclic VT–PXRD measurements (cooling–heating) of the salt **1**; D: zoomed data of the calculated (upper) pattern obtained from scXRD data at 270, 200 and 100 K (shocked), zoomed data for the cooling process 270 → 200 → 100 K (bottom) and zoom data for heating 100 → 200 → 270 K (down).

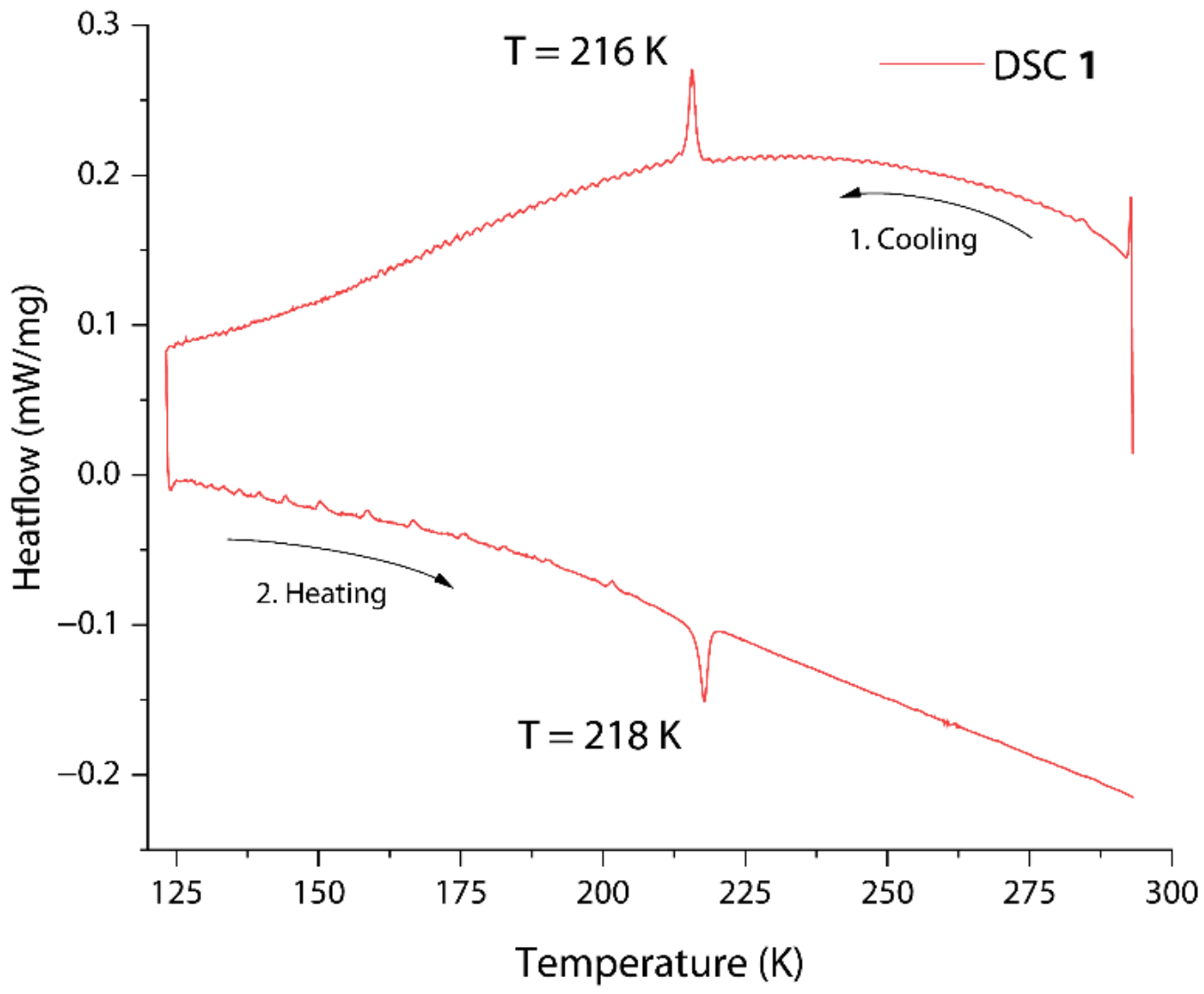

Figure S22. Cyclic DSC measurements (cooling to 150 K, then heating to 297 K) of the crystalline **1**.

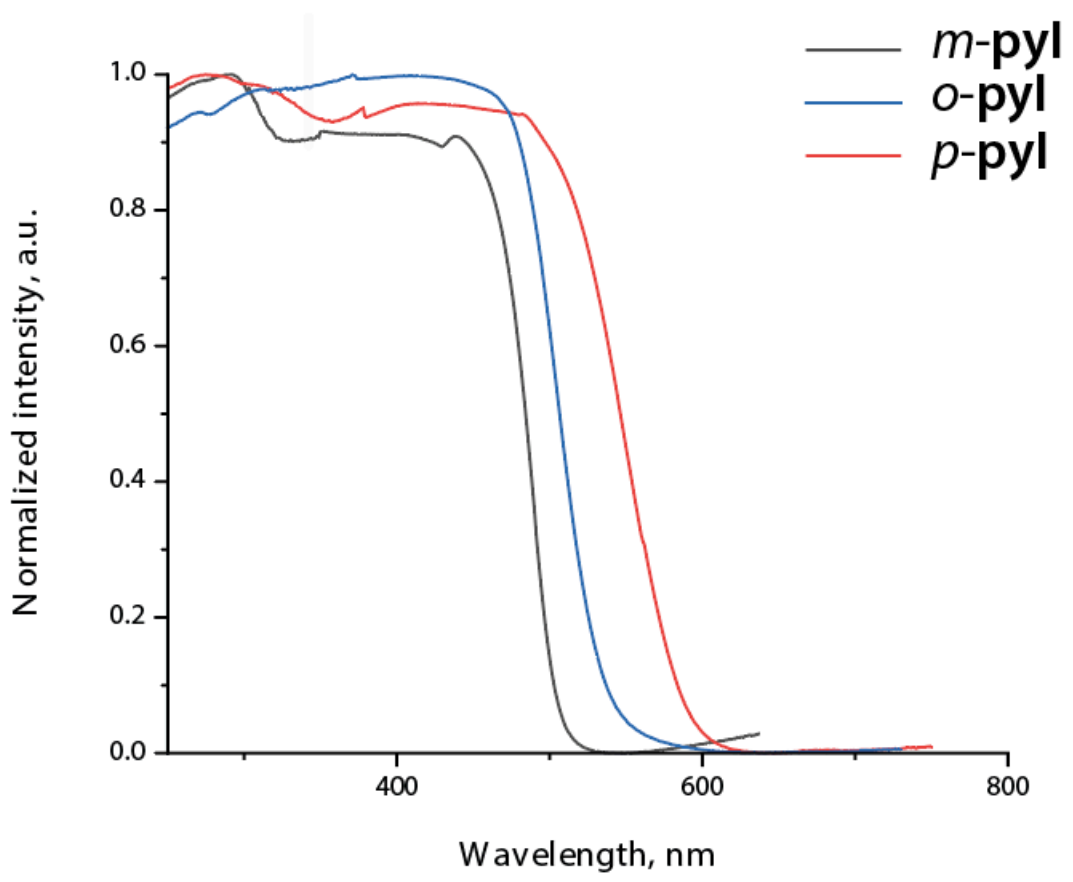

Figure S23. Solid-state reflectance spectra of *p*-**pyl**, *o*-**pyl** and *m*-**pyl** measured at 297 K.

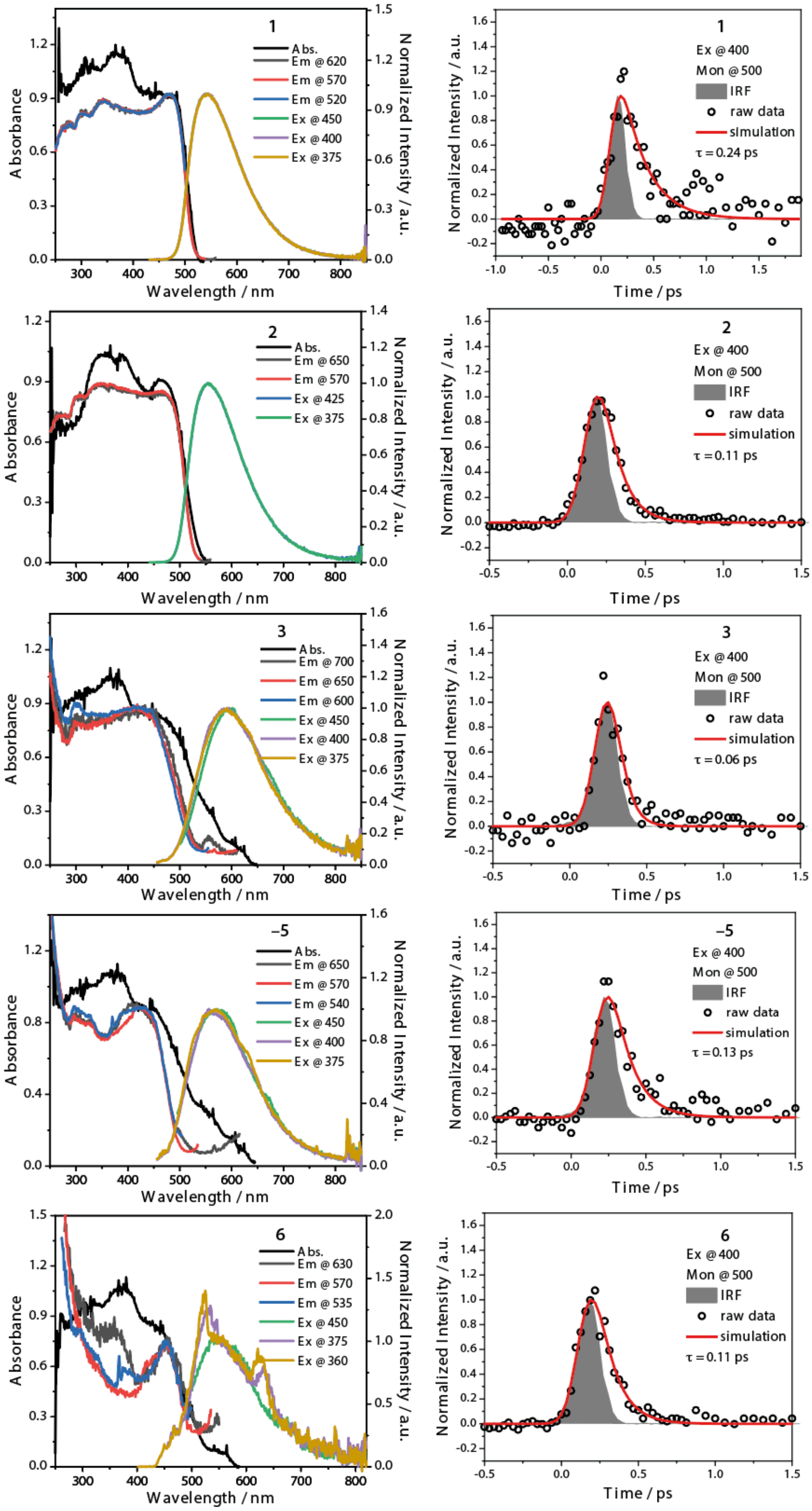

Figure S24. Left column – solid-state absorption (synchronous scans), steady-state excitation, and emission spectra of crystalline samples **1–3**, $^{-}$**5** and **6** measured at 297 K; right column – early relaxation decays and fits measured by fluorescence up-conversion technique.

Table S11. Summary of the photophysical properties of pyridinium salts (**1-4, 1**^{Br}, **⁻5, 6**^{Br}**, 6**) in the solid state measured at VT.

	T [K]	λ_{exc} [nm]	λ_{em} [nm]	$\tau^i(A^i)$ [μs]	τ_{av} [μs]^a	Q.Y.	k_r^b [x10^5 s^{-1}]	k_{nr}^b [x$10^6$$s^{-1}$]
**1**	317	472	543	0.448	-			
	297	471	541	0.699	-	0.22	3.1	1.1
	257	471	543	1.151	-			
	237	465	546	1.325	-			
	217	344	548	1.487	-			
	197	363	554	1.917	-			
	187	362	557	2.043	-			
	177	364	558	2.175	-			
	167	339	556	2.366	-			
	117	352	559	3.106	-			
	77	359	560	3.563	-			
**2**	297	331	553	0.440	-	0.23	5.2	1.6
	257	341	559	1.004	-			
	217	460	563	1.374	-			
	167	443	563	1.806	-			
	117	455	567	2.265	-			
	77	336	565	2.702	-			
**3**	297	444	572	0.013 (40), 0.032 (60)	0.025	0.01	4.0	39.6
	257	289	554	0.14 (96), 0.54 (4)	0.155			
	217	332	551	0.30 (64), 0.63 (36)	0.419			
	167	293	538	0.97 (57), 1.70 (43)	1.280			
	117	338	541	2.97 (67), 4.42 (33)	3.444			
	77	423	540	5.50 (67), 7.71 (33)	6.229			
**4**	297	335	604	0.23(45), 0.64 (55)	0.452	0.11	2.4	2.0
	257	335	603	0.445(29), 0.99 (71)	0.829			
	217	329	606	1.01 (58), 1.49 (42)	1.211			
	167	328	608	1.74 (98), 3.41 (2)	1.770			
	117	332	611	2.27 (99), 7.70 (1)	2.330			
	77	328	612	2.61 (99), 10.13 (1)	2.688			
**⁻5**	297	419	567	0.022 (81), 0.043 (19)	0.027	0.01	3.3	36.7
	257	427	577	0.07 (10), 0.21 (82), 0.52 (8)	0.219			
	217	427	581	0.37 (12), 1.44 (88)	1.309			
	167	429	581	2.40 (91), 3.42 (9)	2.496			
	117	429	582	3.202	-			
	77	315	570	3.932	-			
**6**^{Br}	77	345	507	26.527 (27), 50.831 (73)	44.22			
**6**	297	451	555	0.051 (56), 0.229 (46)	0.089			
	77	460	570	1.762 (53), 4.176 (47)	2.89			
**1**^{Br}	297	330	535	0.19 (4), 0.81 (35), 2.57 (32), 5.56 (29)	1.23			
	77	335	540	8.44 (3), 27.04 (94), 53.76 (2)	25.36			

^{a} Average amplitude-weighted emission lifetime for multiexponential decays ($\tau_{av} = \Sigma A^i\tau^i$, where A^i – the weight of the exponent). ^{b} Calculated from amplitude average lifetimes k_r = Q.Y/τ_{av}; k_{nr} = (1-Q.Y)/τ_{av}.

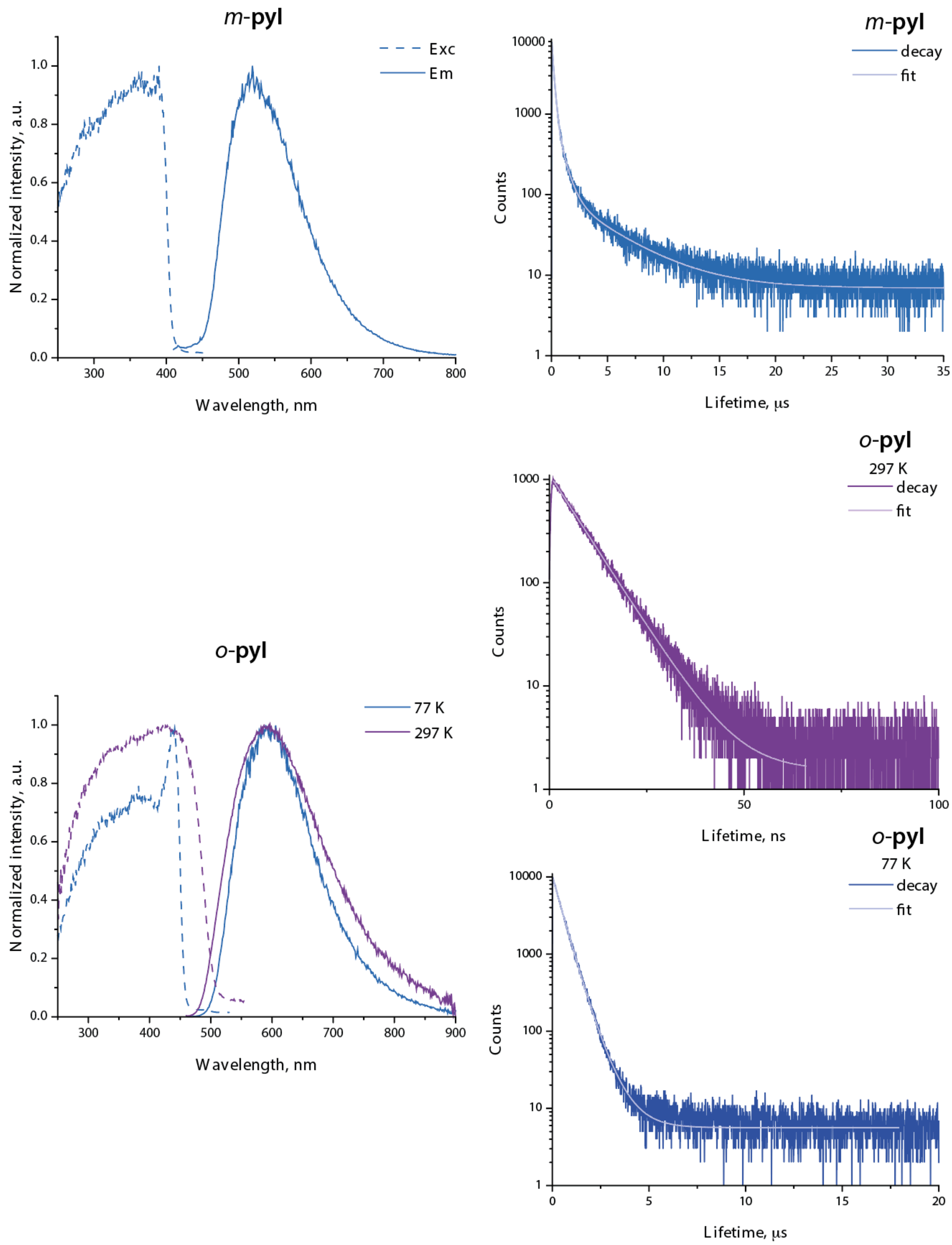

Figure S25. Steady-state excitation, emission, and time-resolved emission spectra of crystalline samples *o*-**pyl and** *m*-**pyl** measured at 297 and/or 77 K.

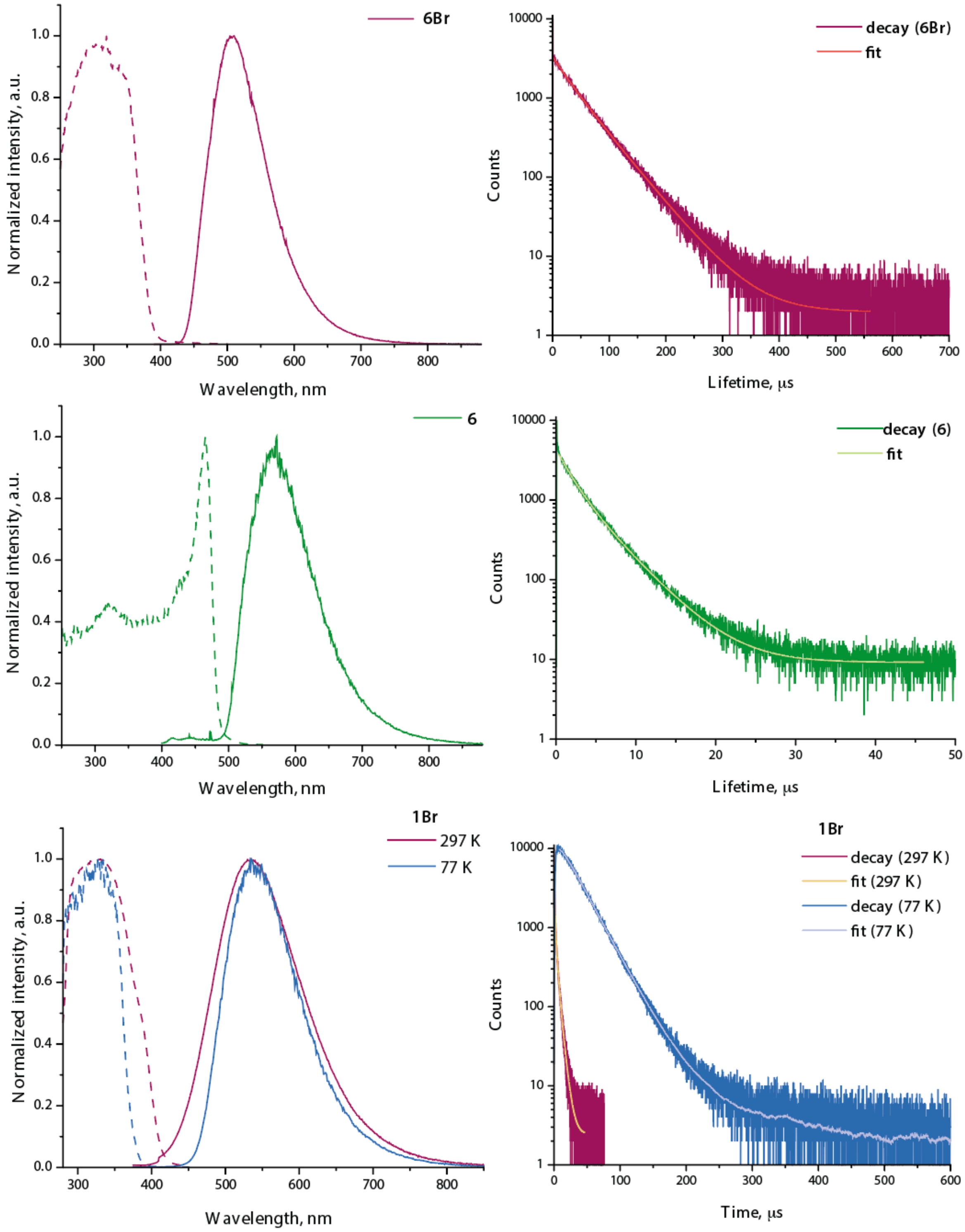

Figure S26. Steady state excitation, emission, and time-resolved emission spectra of crystalline samples **6**^{Br} measured at 77 K, and **6** and **1**^{Br} measured at 297 and 77 K.

Table S12. HOMO/LUMO levels; excitation ($S_0 \rightarrow S_1$), fluorescence ($S_1 \rightarrow S_0$) and triplet emission (T_2, $T_1 \rightarrow S_0$) electron density difference plots for **1** (isovalue of 0.04 a.u., loss electron density: blue, gain: red).

HOMO	LUMO
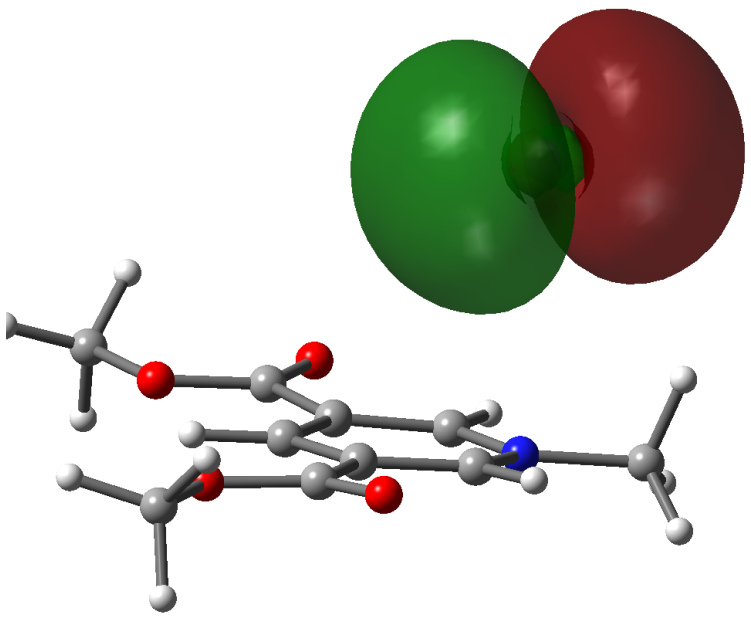	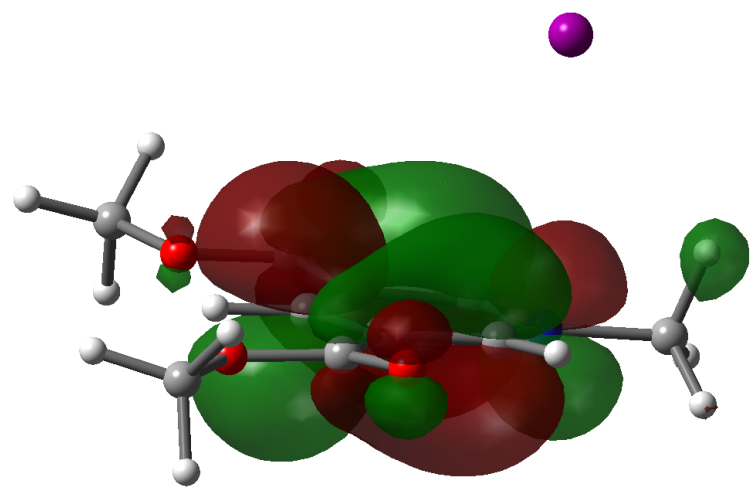
Excitation ($S_0 \rightarrow S_1$)	Fluorescence ($S_1 \rightarrow S_0$)
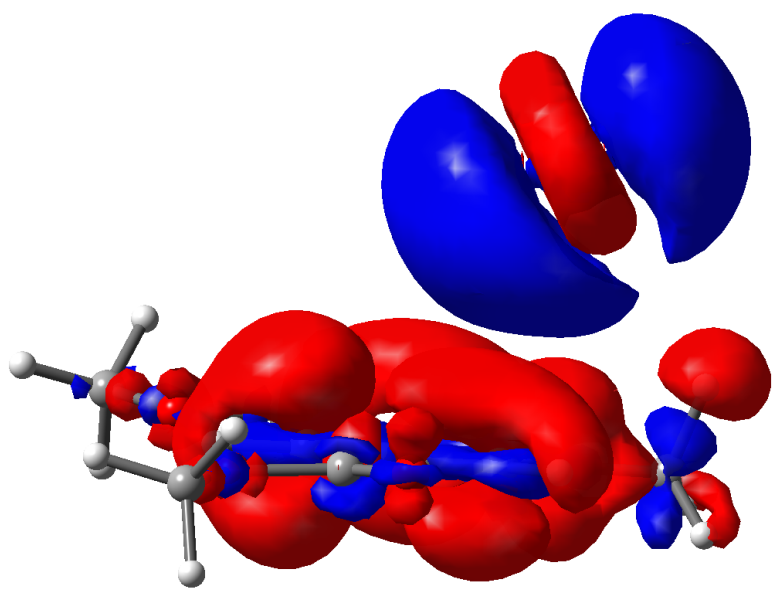	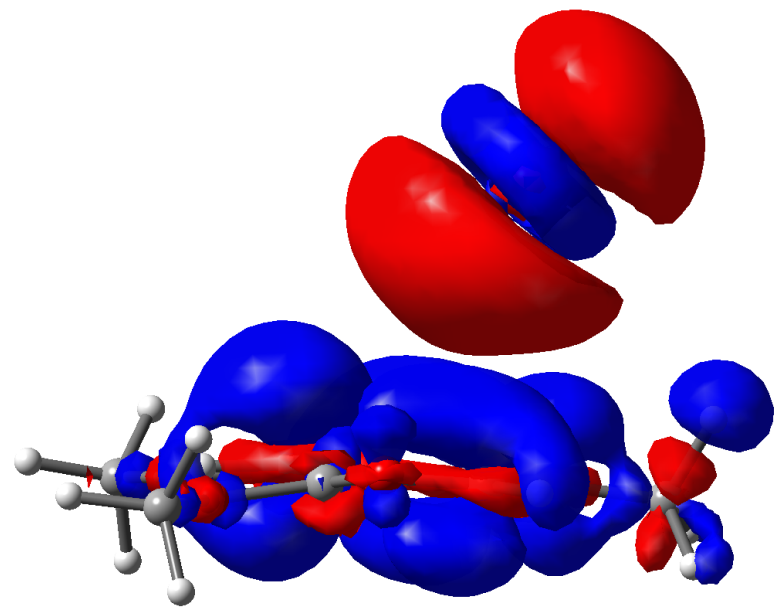
Phosphorescence ($T_2 \rightarrow S_0$)	Phosphorescence ($T_1 \rightarrow S_0$)
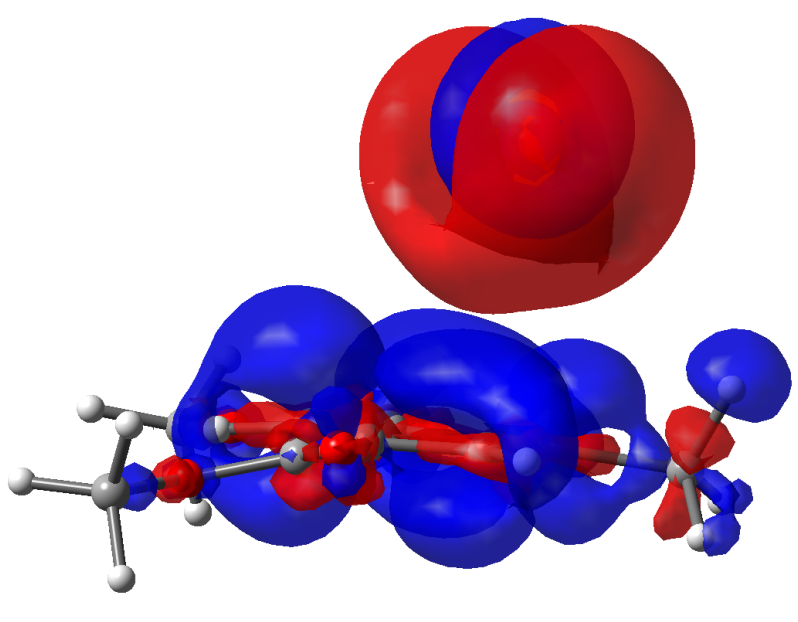	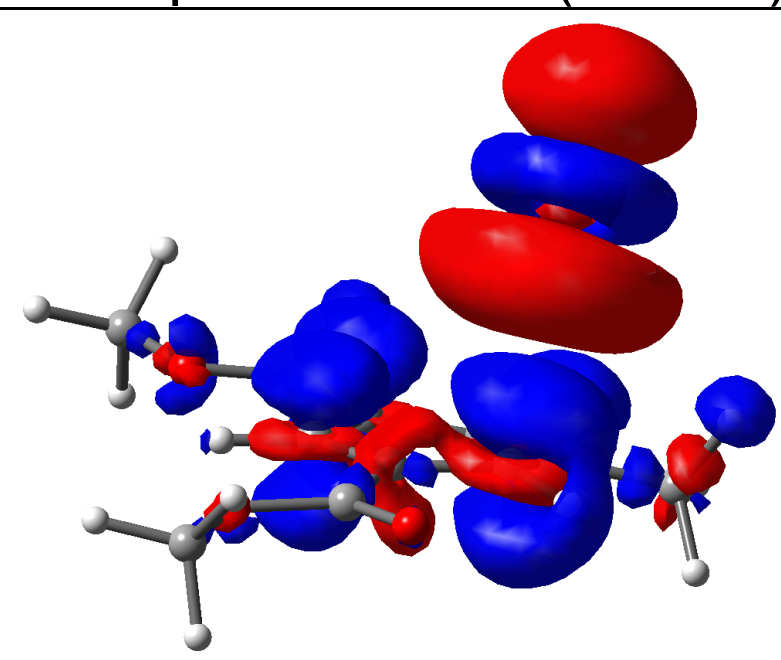

Table S13. HOMO/LUMO levels; excitation ($S_0 \rightarrow S_1$), fluorescence ($S_1 \rightarrow S_0$) and triplet emission (T_2, $T_1 \rightarrow S_0$) electron density difference plots for $I^{-} \cdots \mathbf{1} \cdots I^{-}$ (isovalue of 0.04 a.u., loss electron density: blue, gain: red).

HOMO	LUMO
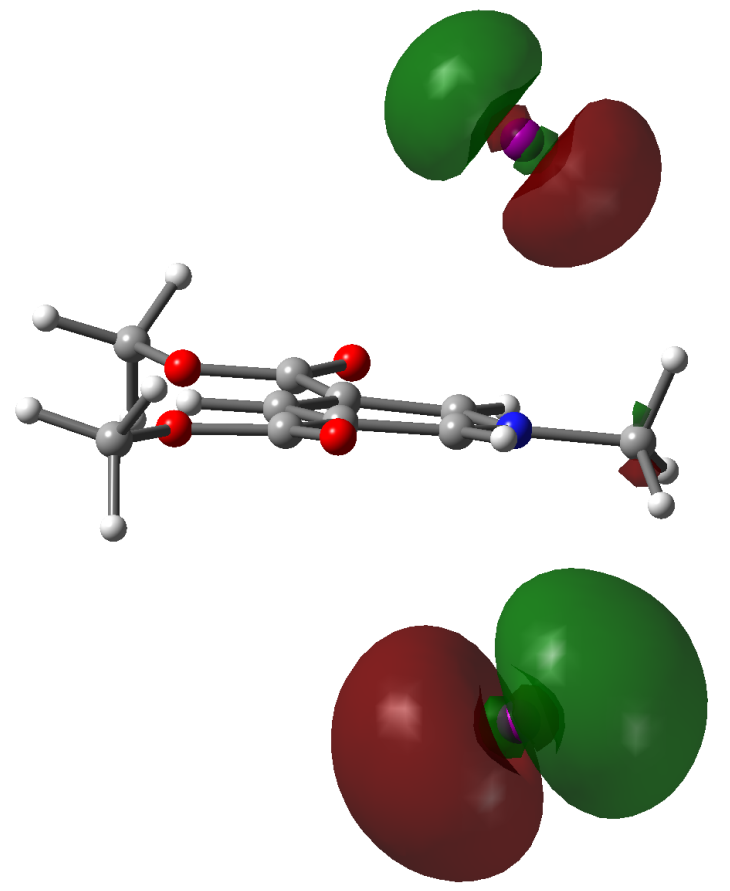	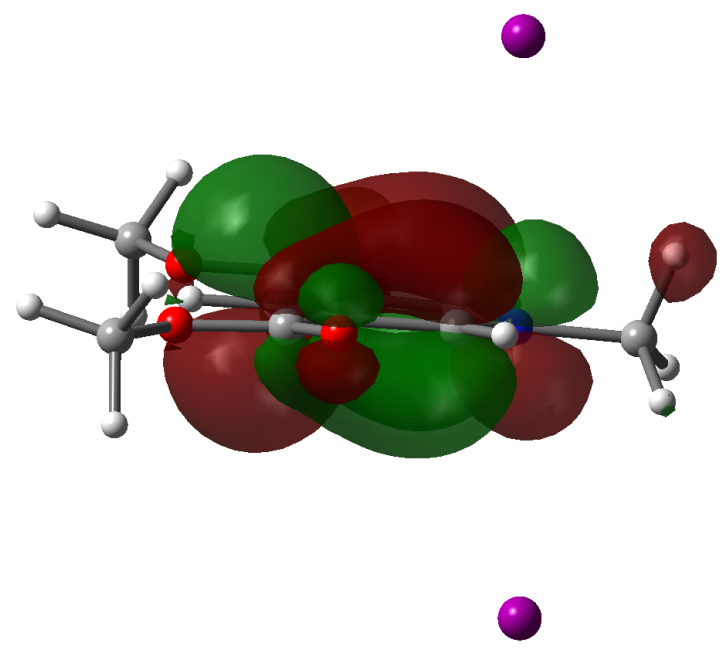
Excitation ($S_0 \rightarrow S_1$)	Fluorescence ($S_1 \rightarrow S_0$)
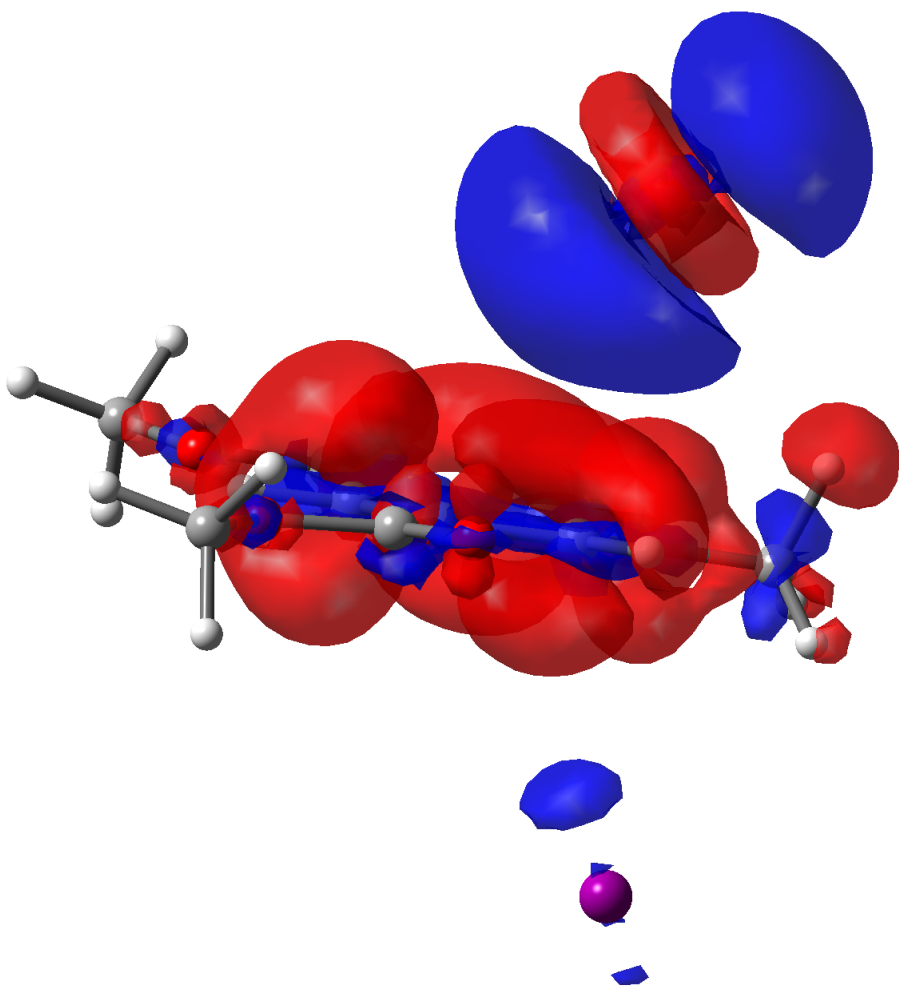	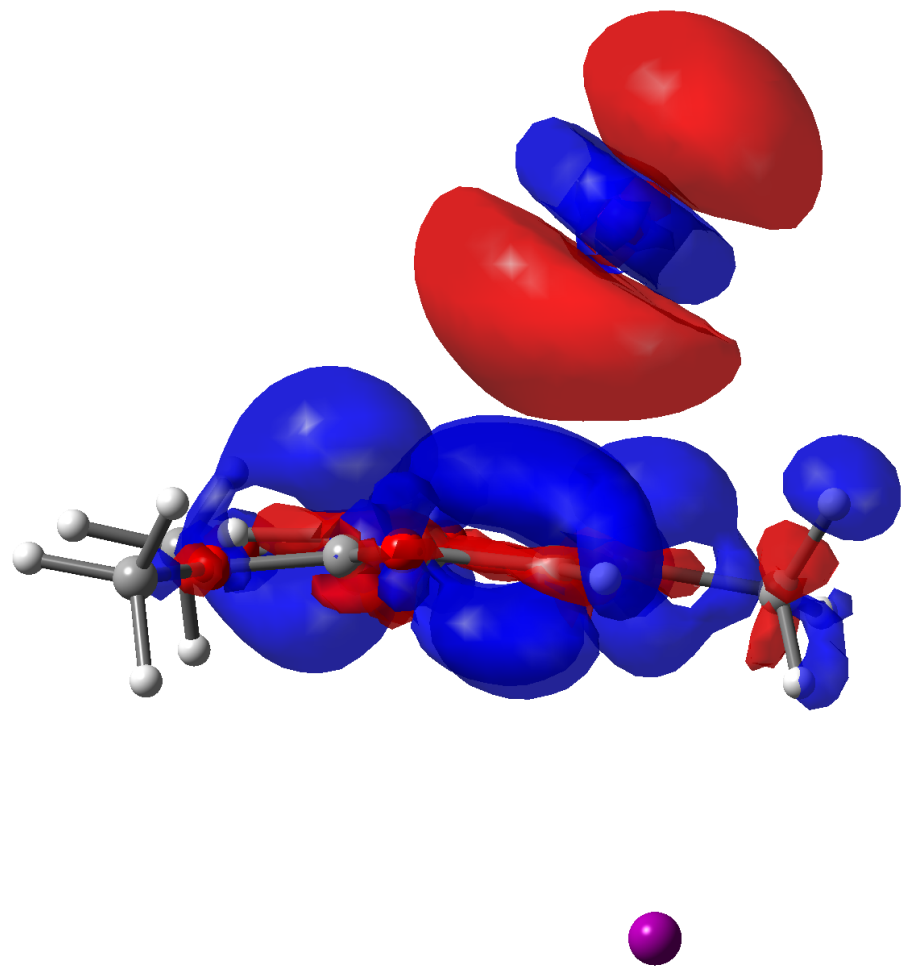
Phosphorescence ($T_2 \rightarrow S_0$)	Phosphorescence ($T_1 \rightarrow S_0$)
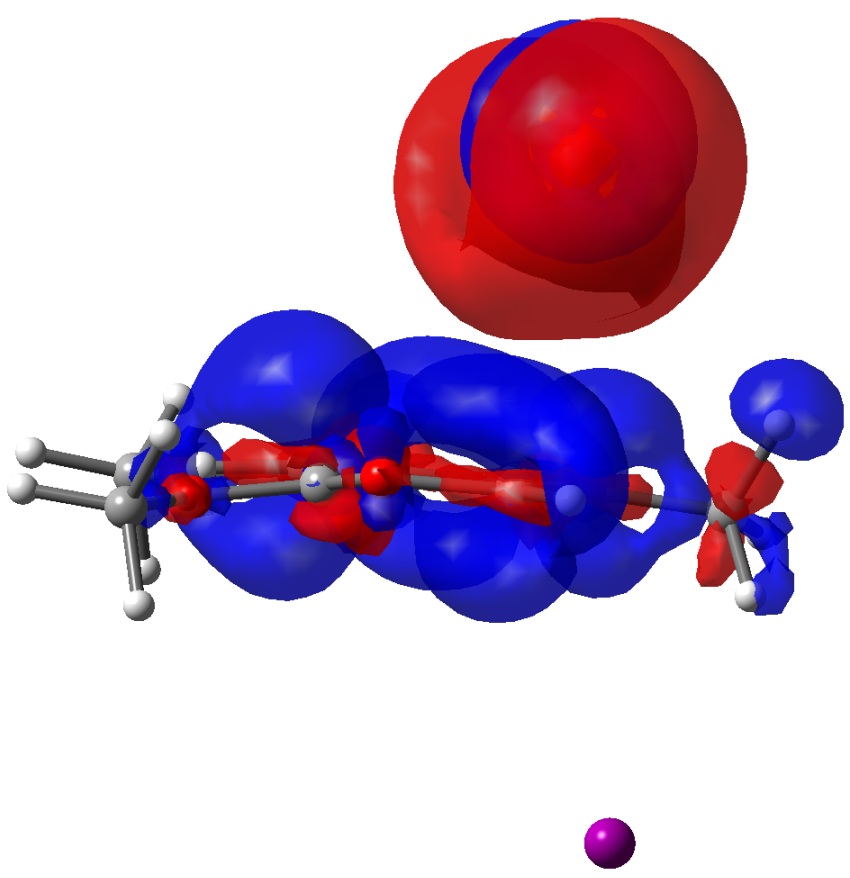	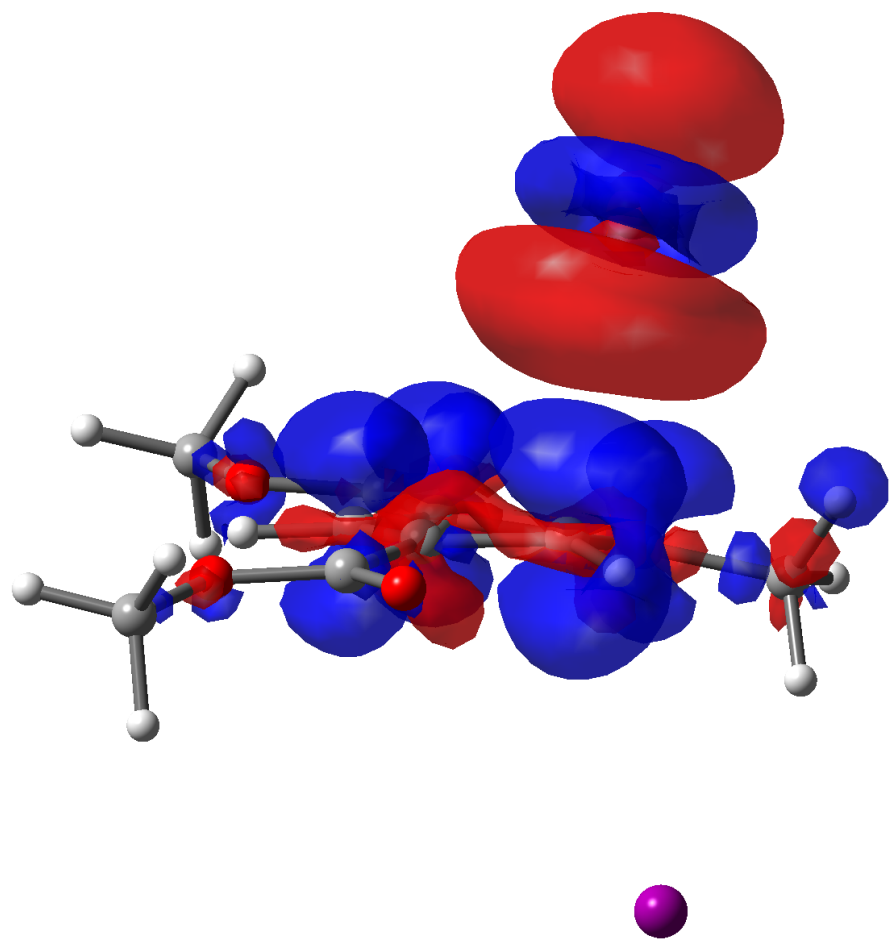

Table S14. HOMO/LUMO levels; excitation ($S_0 \rightarrow S_1$), fluorescence ($S_1 \rightarrow S_0$) and triplet emission (T_2, $T_1 \rightarrow S_0$) electron density difference plots for **2** (isovalue of 0.04 a.u., loss electron density: blue, gain: red).

HOMO	LUMO
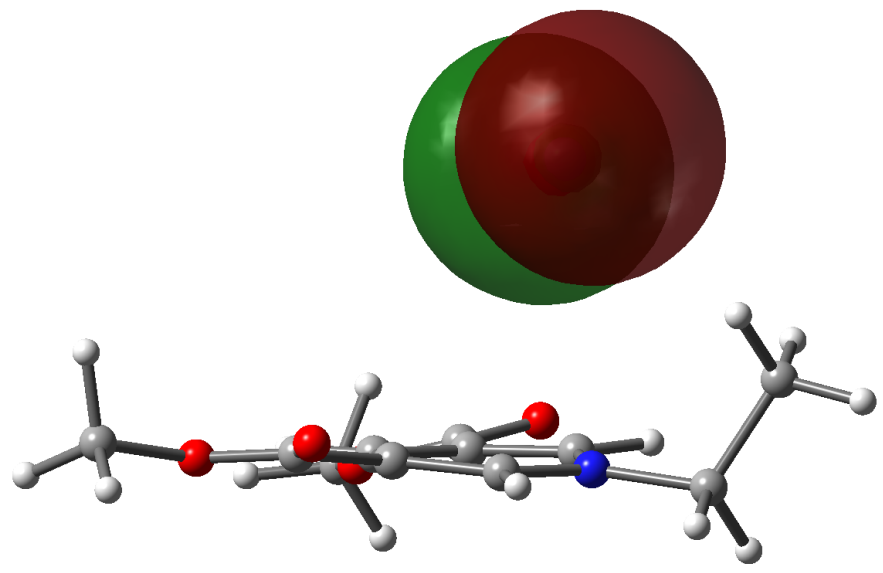	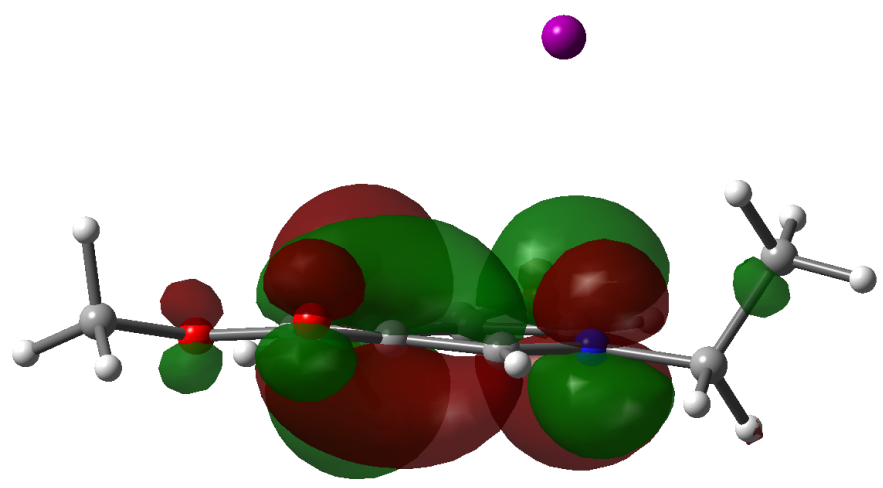
Excitation ($S_0 \rightarrow S_1$)	Fluorescence ($S_1 \rightarrow S_0$)
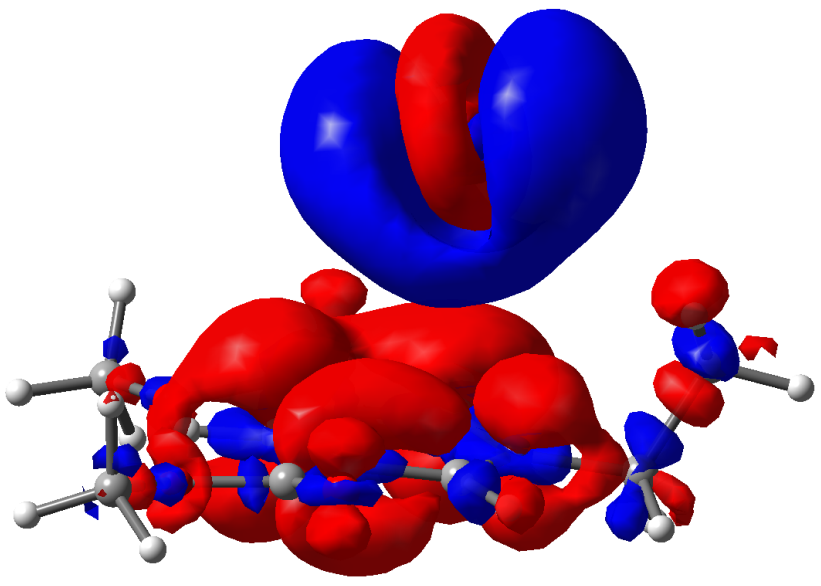	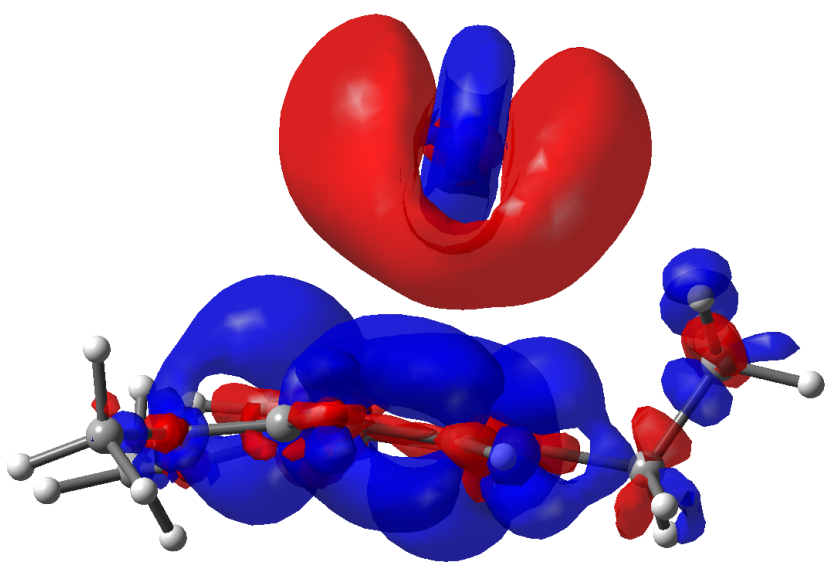
Phosphorescence ($T_2 \rightarrow S_0$)	Phosphorescence ($T_1 \rightarrow S_0$)
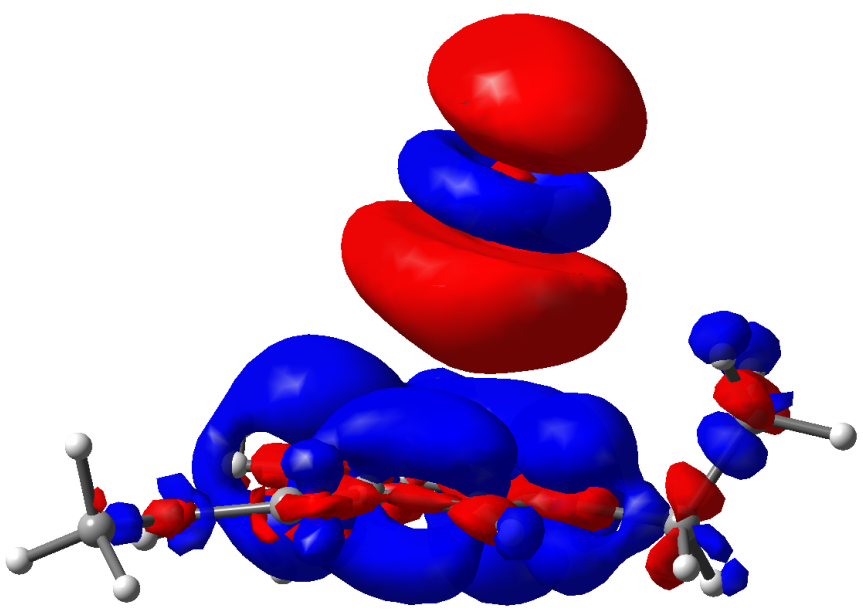	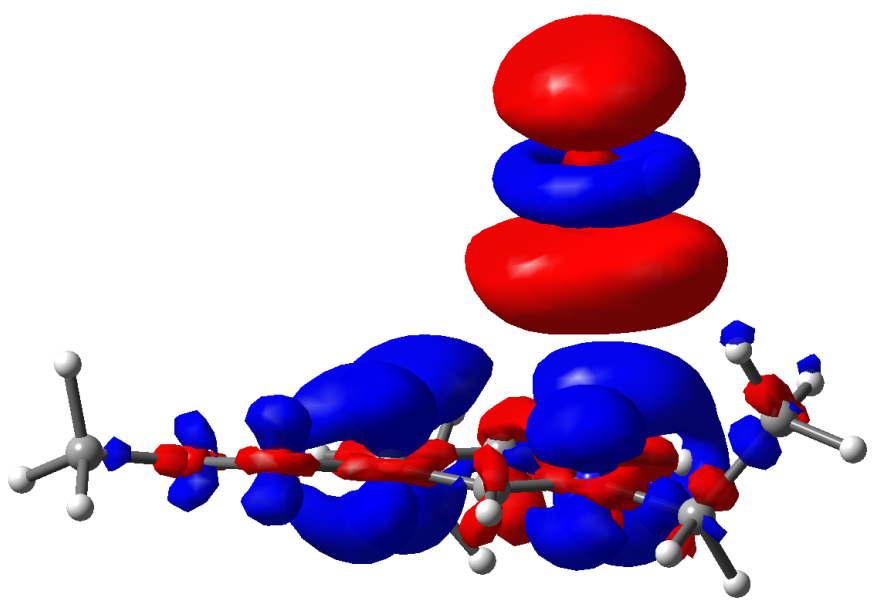

Table S15. HOMO/LUMO levels; excitation ($S_0 \rightarrow S_1$), fluorescence ($S_1 \rightarrow S_0$) and triplet emission (T_2, $T_1 \rightarrow S_0$) electron density difference plots for $I^{-}\cdots\mathbf{2}\cdots I^{-}$ (isovalue of 0.04 a.u., loss electron density: blue, gain: red).

HOMO	LUMO
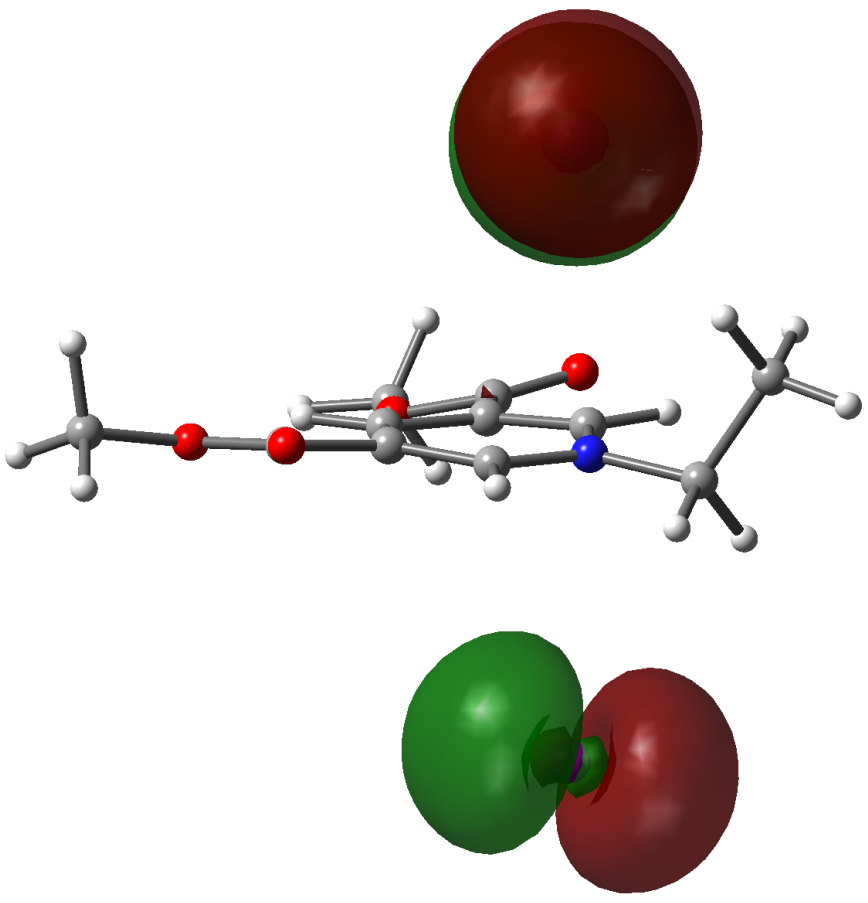	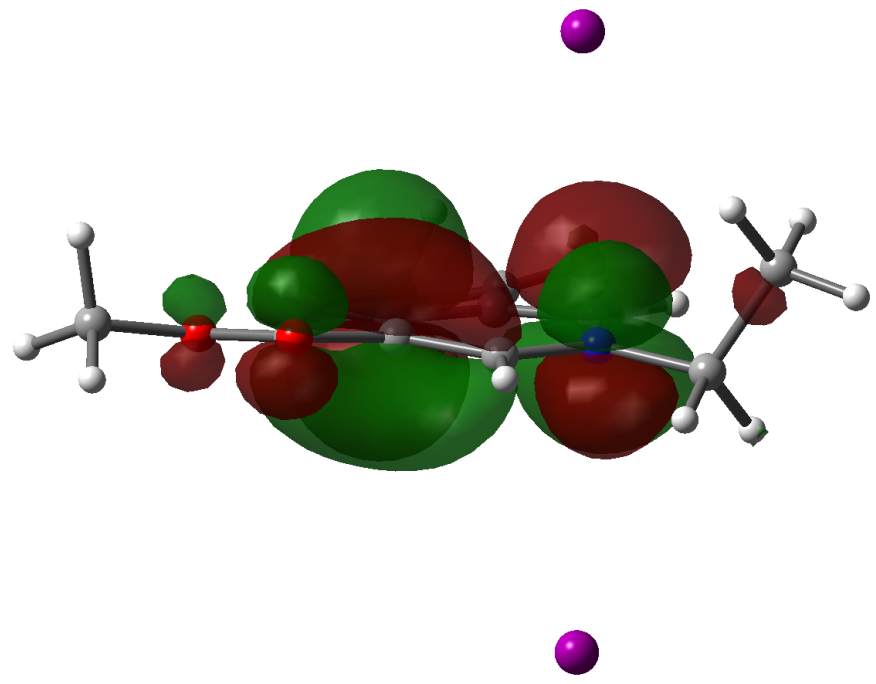
Excitation ($S_0 \rightarrow S_1$)	Fluorescence ($S_1 \rightarrow S_0$)
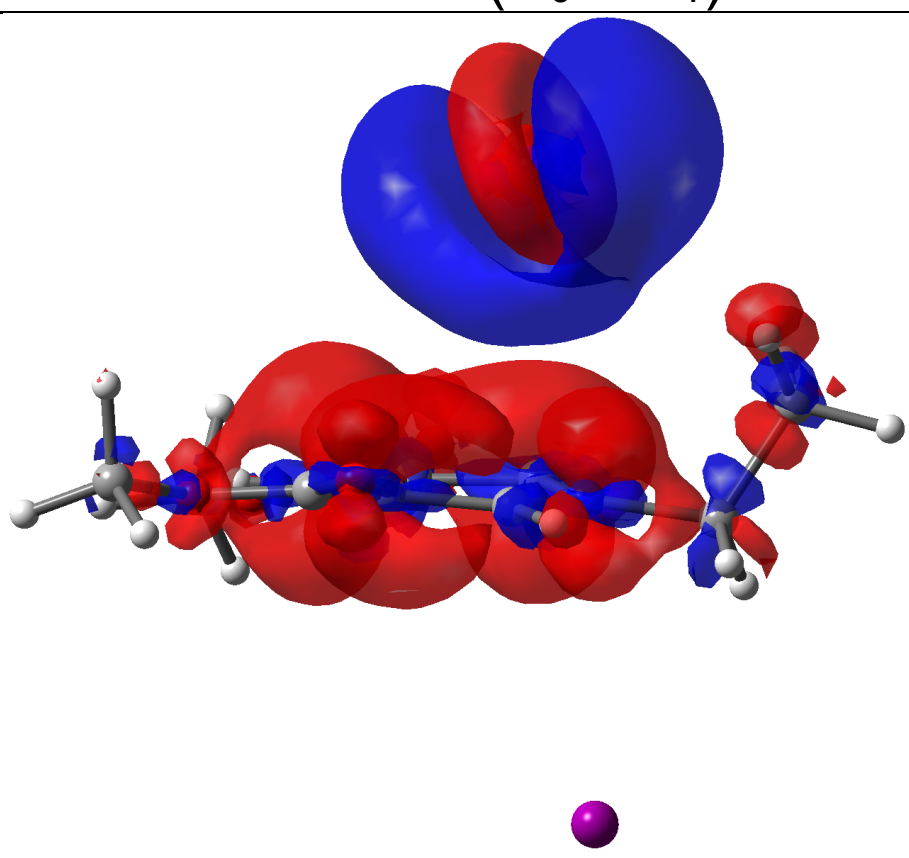	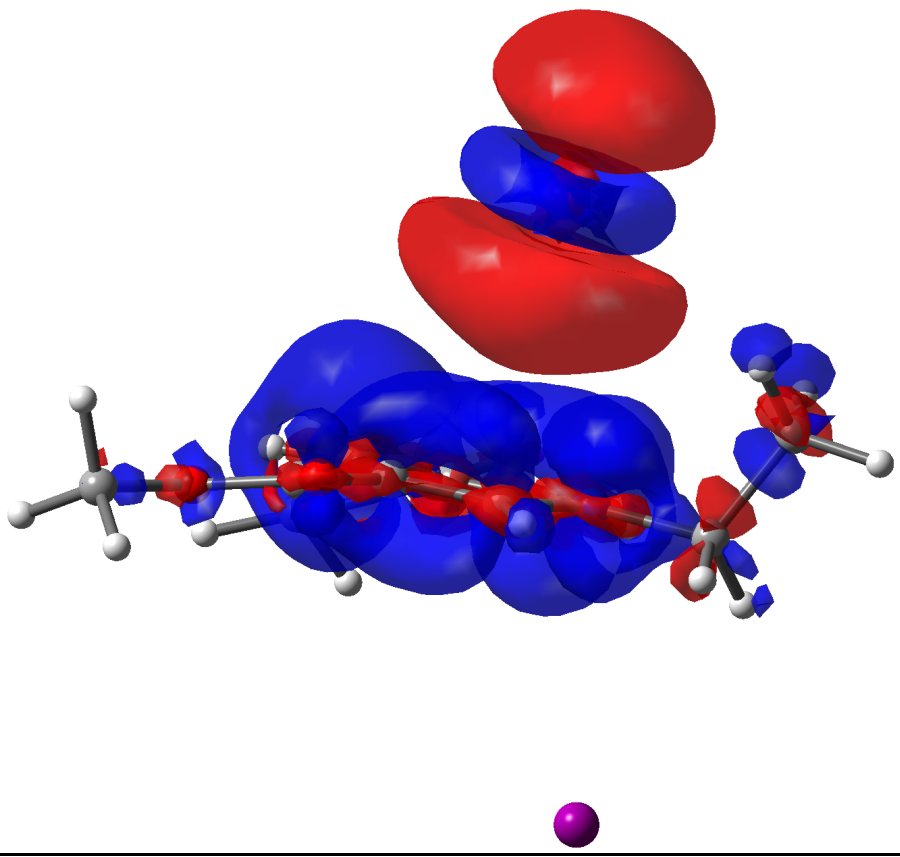
Phosphorescence ($T_2 \rightarrow S_0$)	Phosphorescence ($T_1 \rightarrow S_0$)
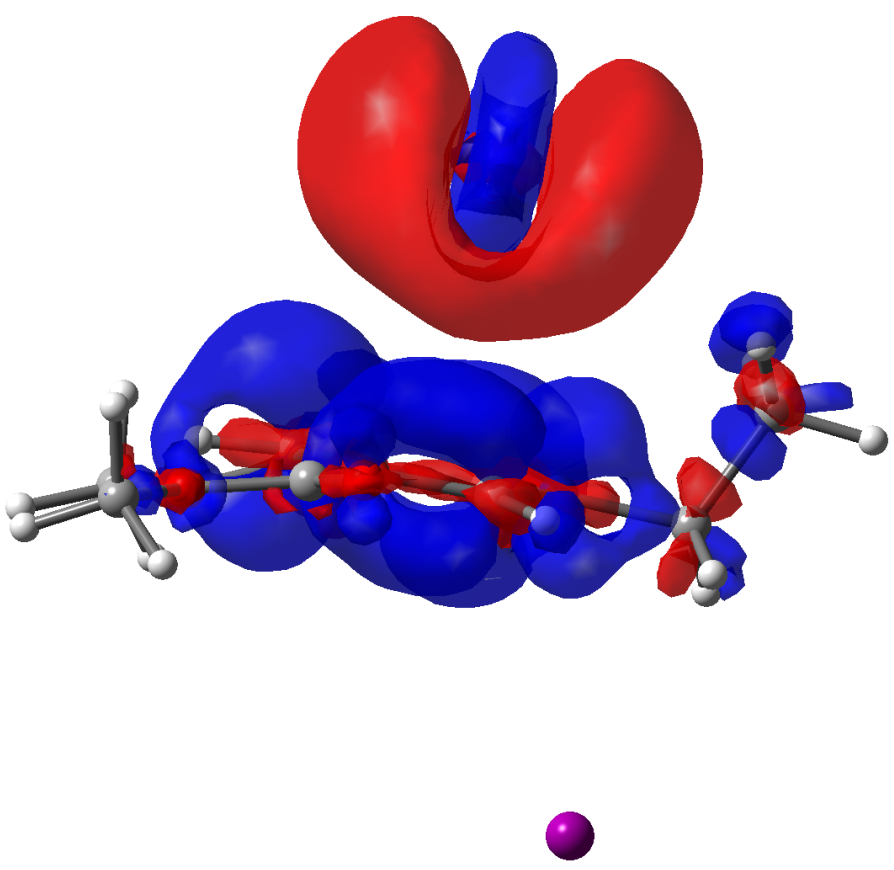	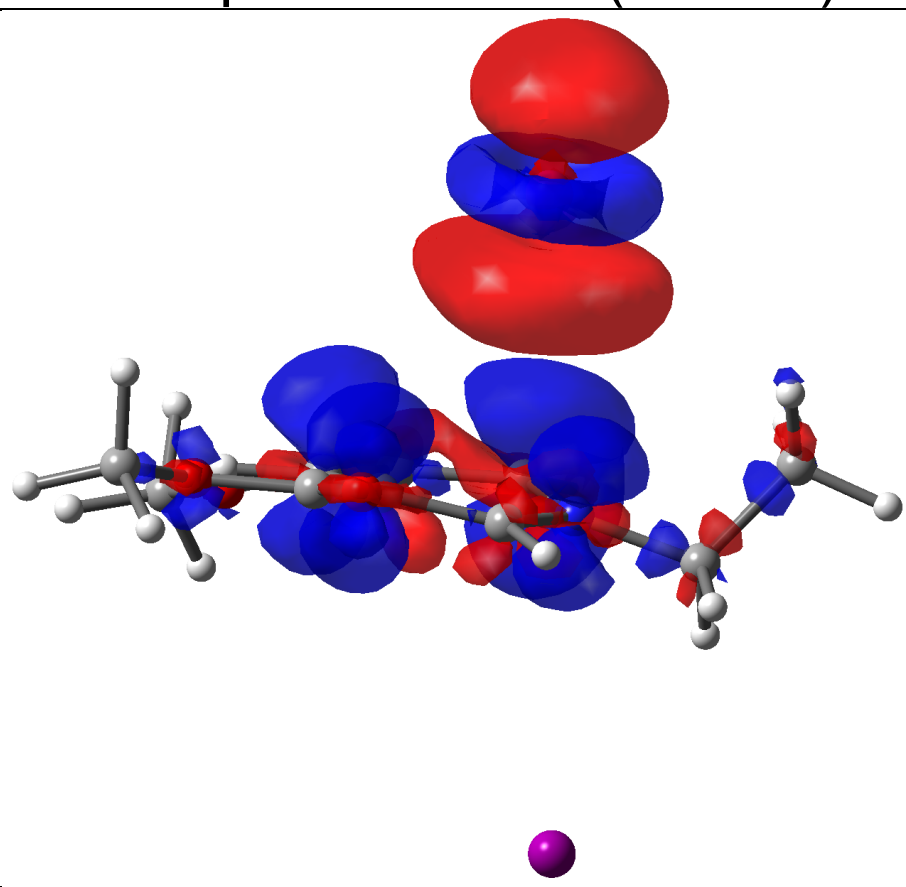

Table S16. HOMO/LUMO levels; excitation ($S_0 \rightarrow S_1$), fluorescence ($S_1 \rightarrow S_0$) and triplet emission (T_2, $T_1 \rightarrow S_0$) electron density difference plots for **3** (isovalue of 0.04 a.u., loss electron density: blue, gain: red).

HOMO	LUMO
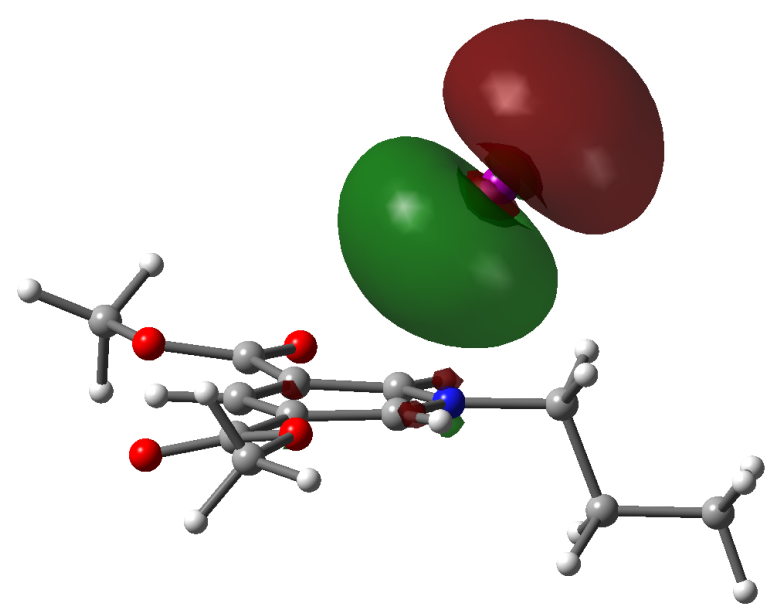	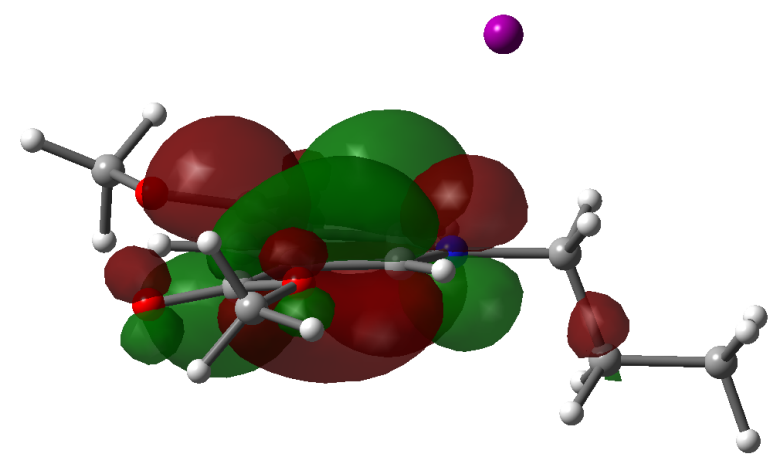
Excitation ($S_0 \rightarrow S_1$)	Fluorescence ($S_1 \rightarrow S_0$)
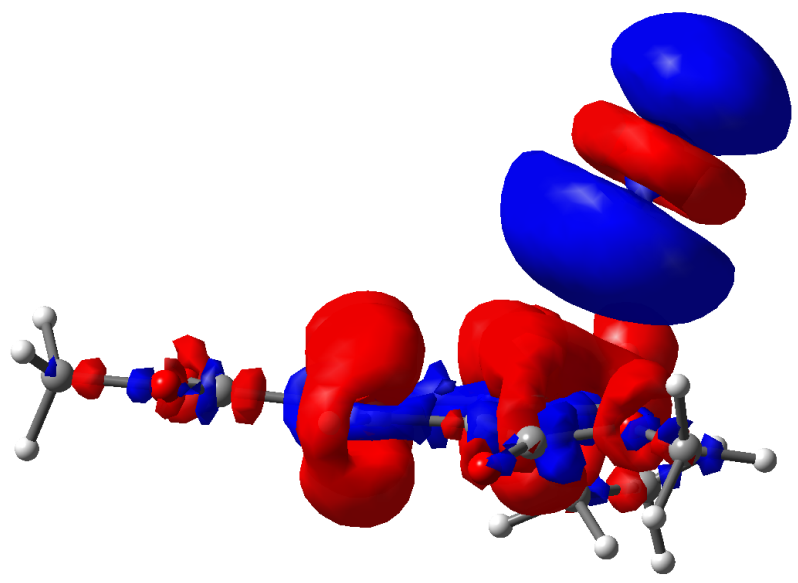	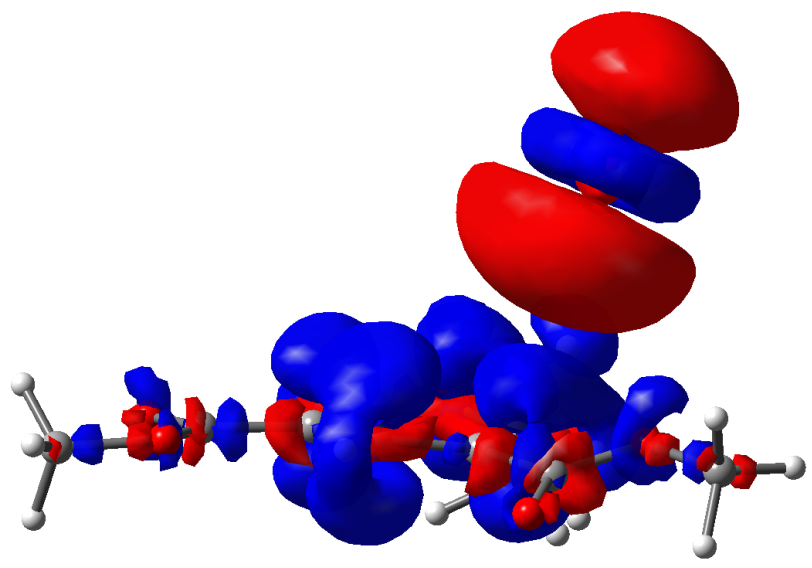
Phosphorescence ($T_2 \rightarrow S_0$)	Phosphorescence ($T_1 \rightarrow S_0$)
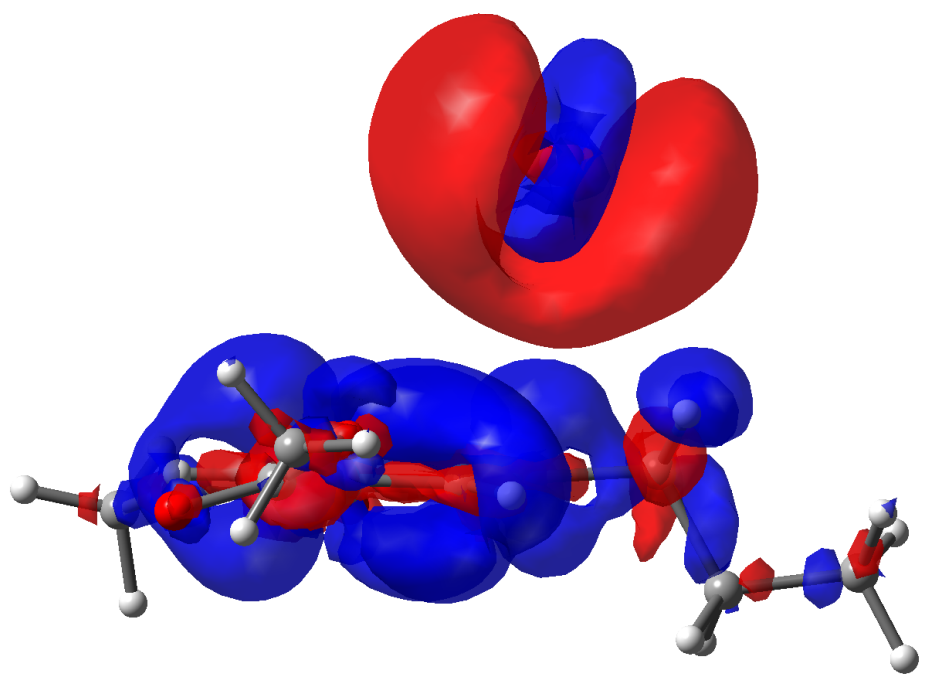	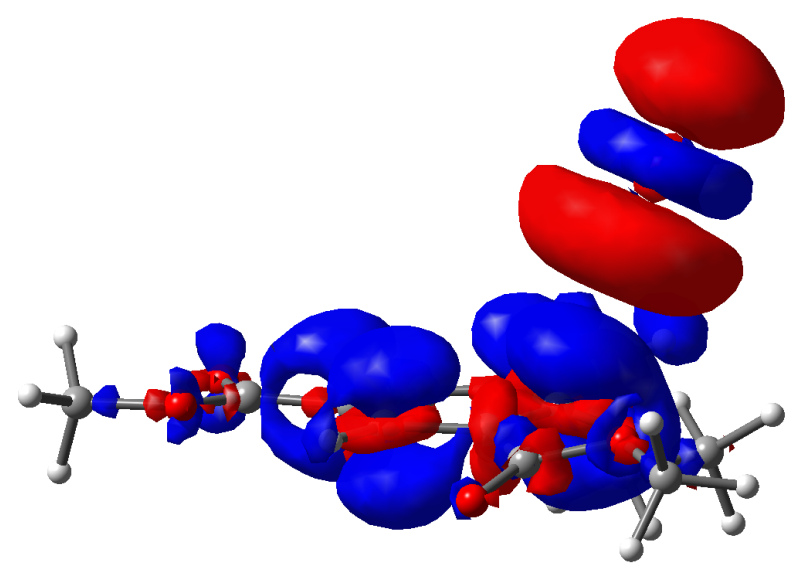

Table S17. HOMO/LUMO levels; excitation ($S_0 \rightarrow S_1$), fluorescence ($S_1 \rightarrow S_0$) and triplet emission (T_2, $T_1 \rightarrow S_0$) electron density difference plots for **4** (isovalue of 0.04 a.u., loss electron density: blue, gain: red).

HOMO	LUMO
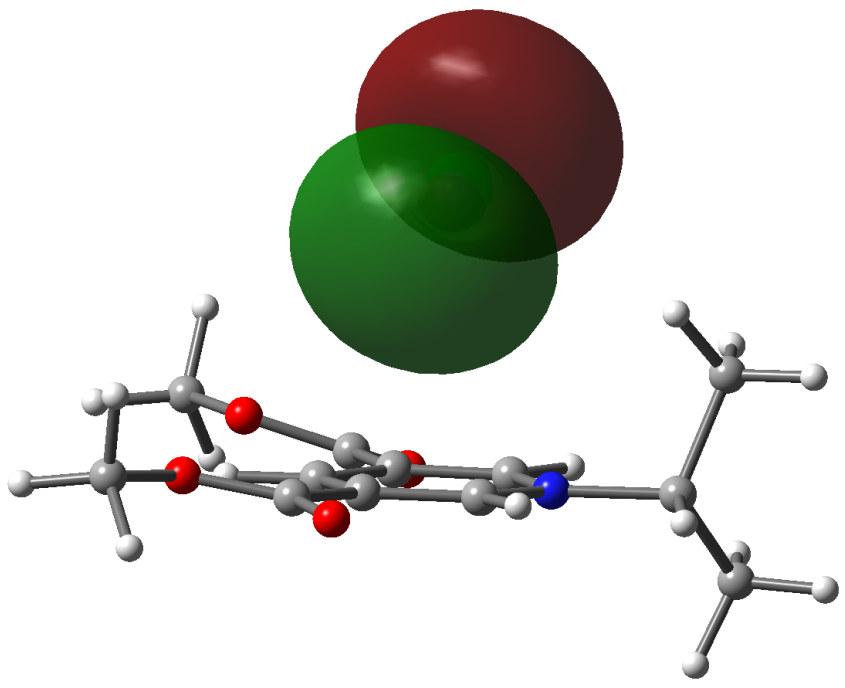	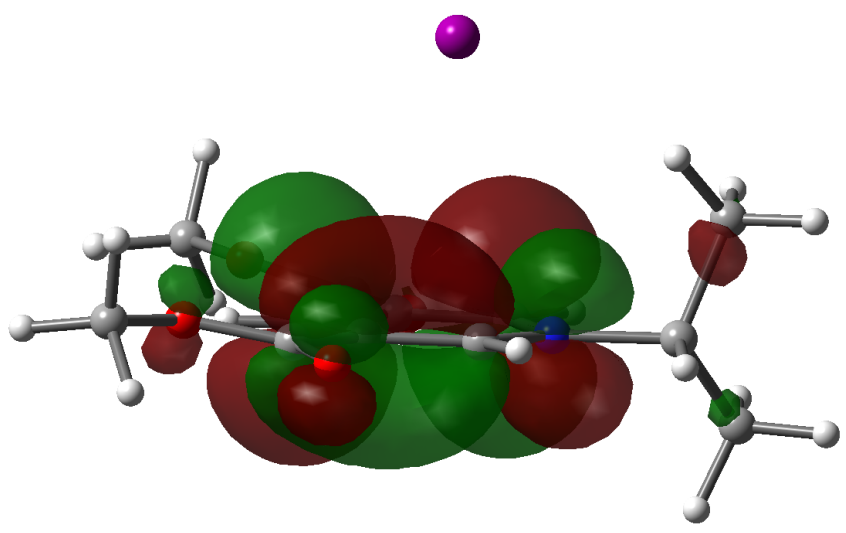
Excitation ($S_0 \rightarrow S_1$)	Fluorescence ($S_1 \rightarrow S_0$)
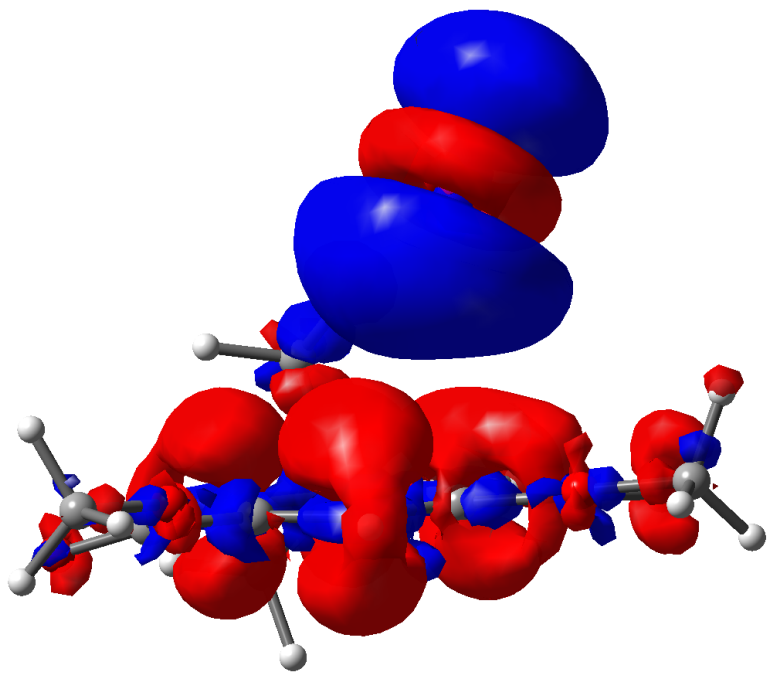	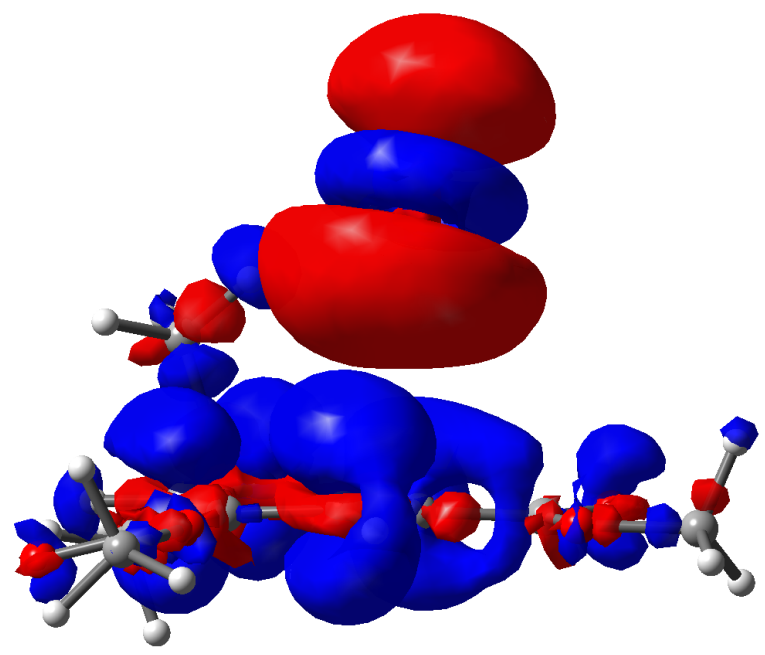
Phosphorescence ($T_2 \rightarrow S_0$)	Phosphorescence ($T_1 \rightarrow S_0$)
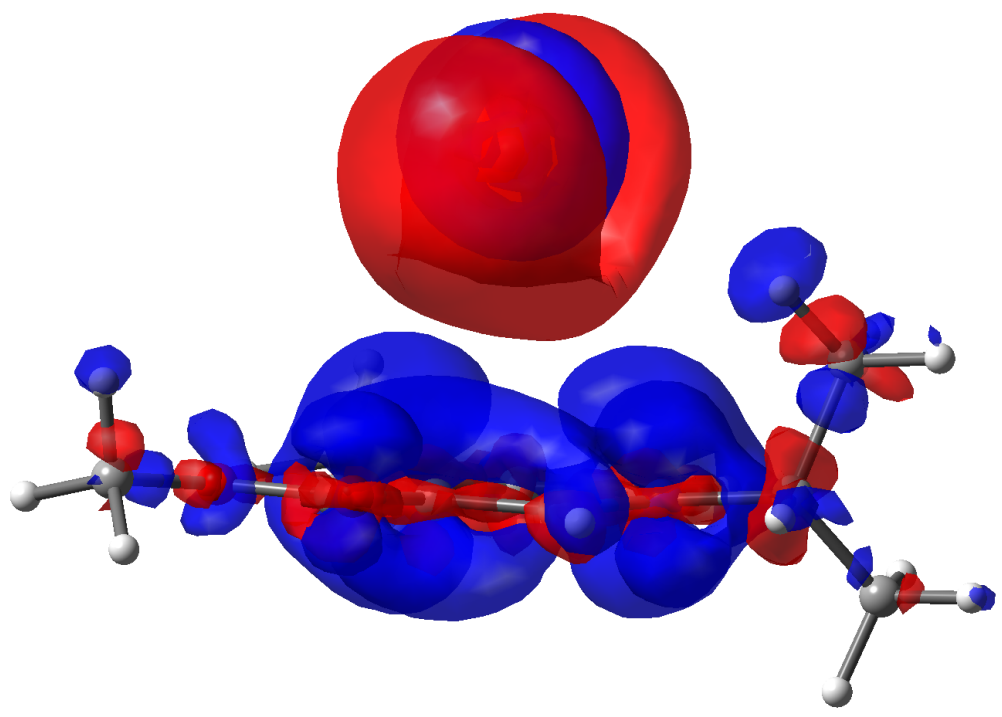	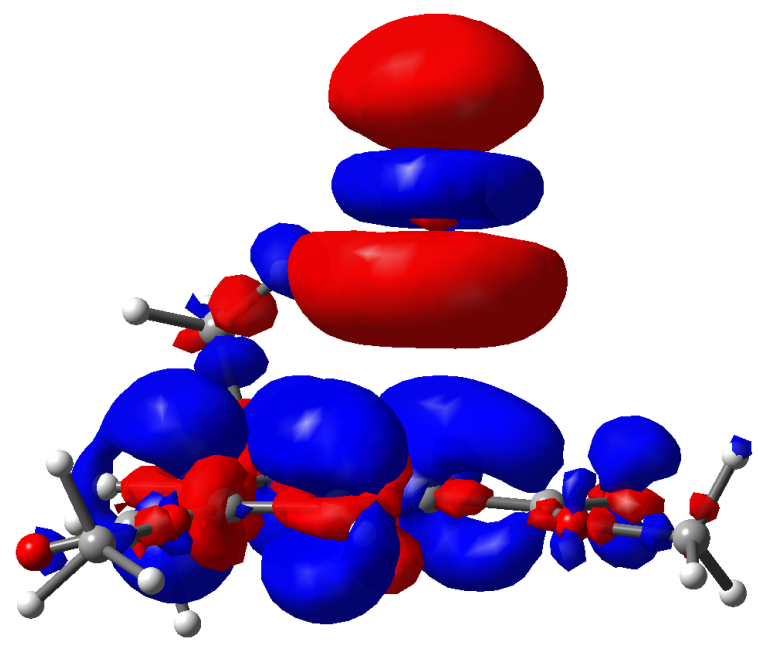

Table S18. HOMO/LUMO levels; excitation ($S_0 \rightarrow S_1$), fluorescence ($S_1 \rightarrow S_0$) and triplet emission (T_2, $T_1 \rightarrow S_0$) electron density difference plots for $I^{-}\cdots$**4**$\cdots I^{-}$ (isovalue of 0.04 a.u., loss electron density: blue, gain: red).

HOMO	LUMO
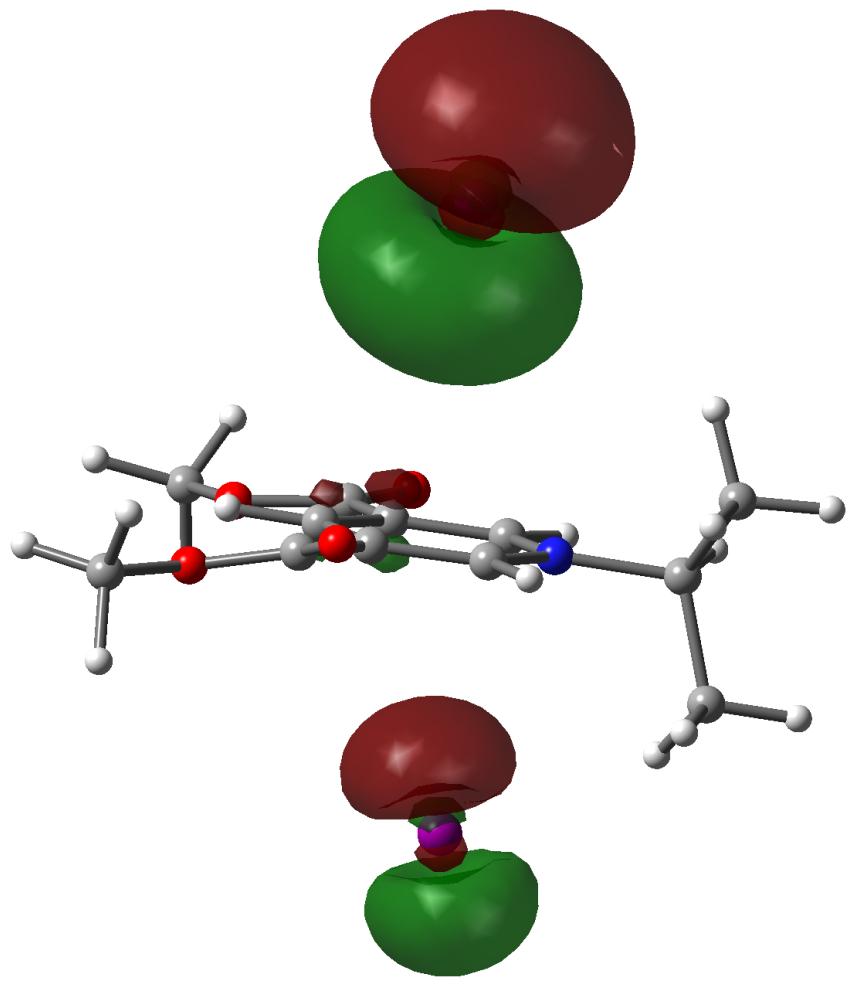	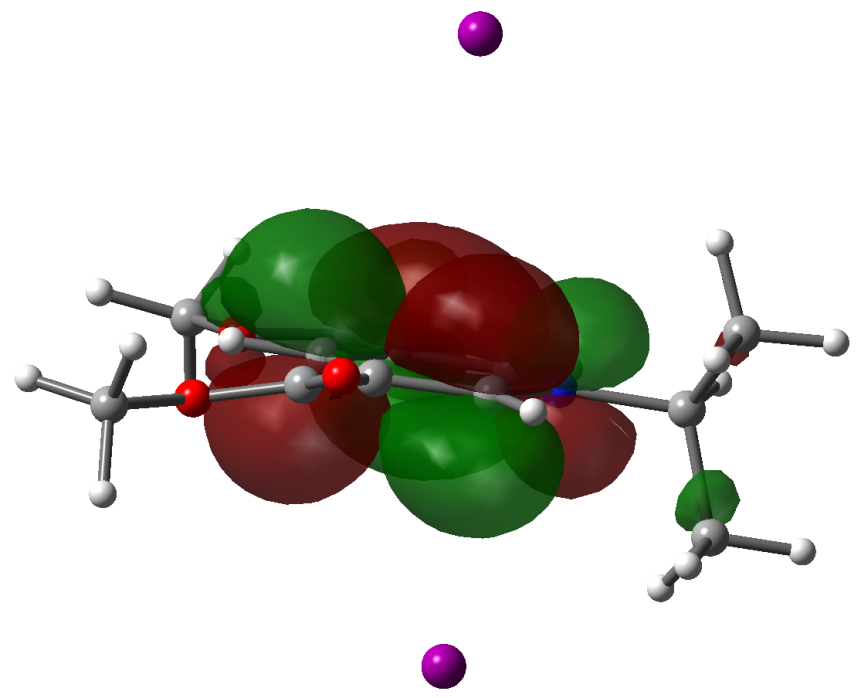
Excitation ($S_0 \rightarrow S_1$)	Fluorescence ($S_1 \rightarrow S_0$)
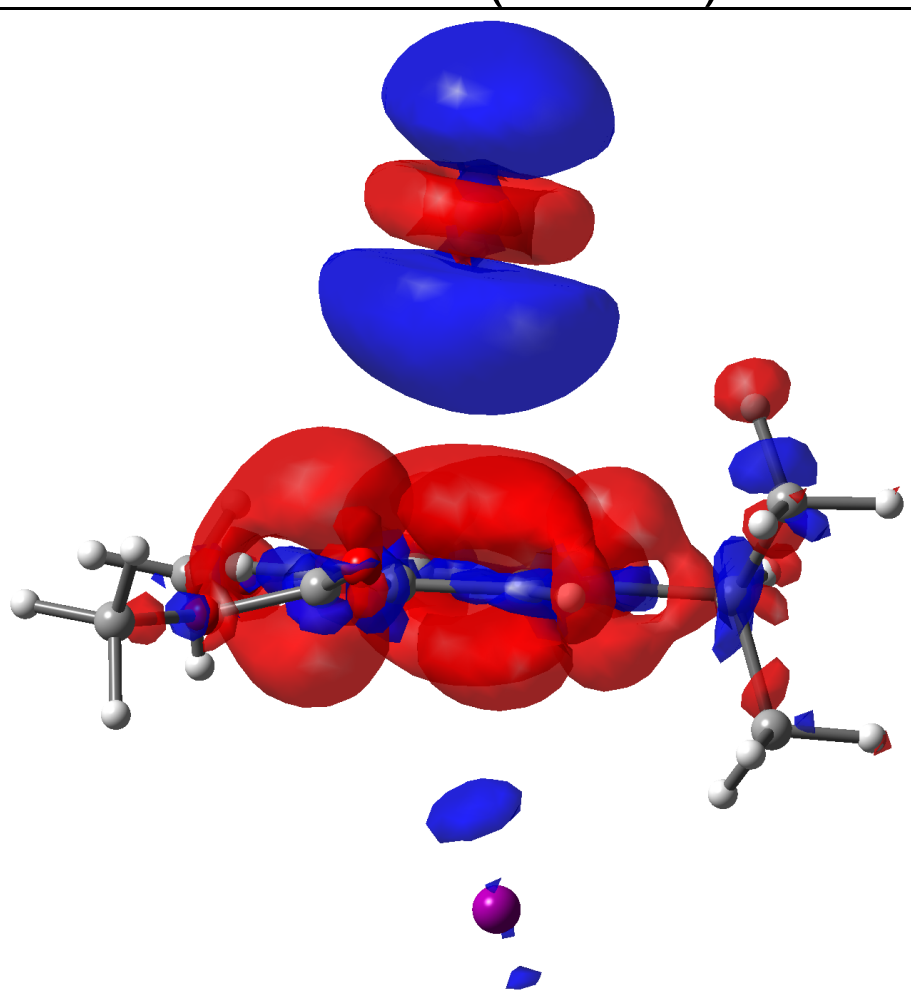	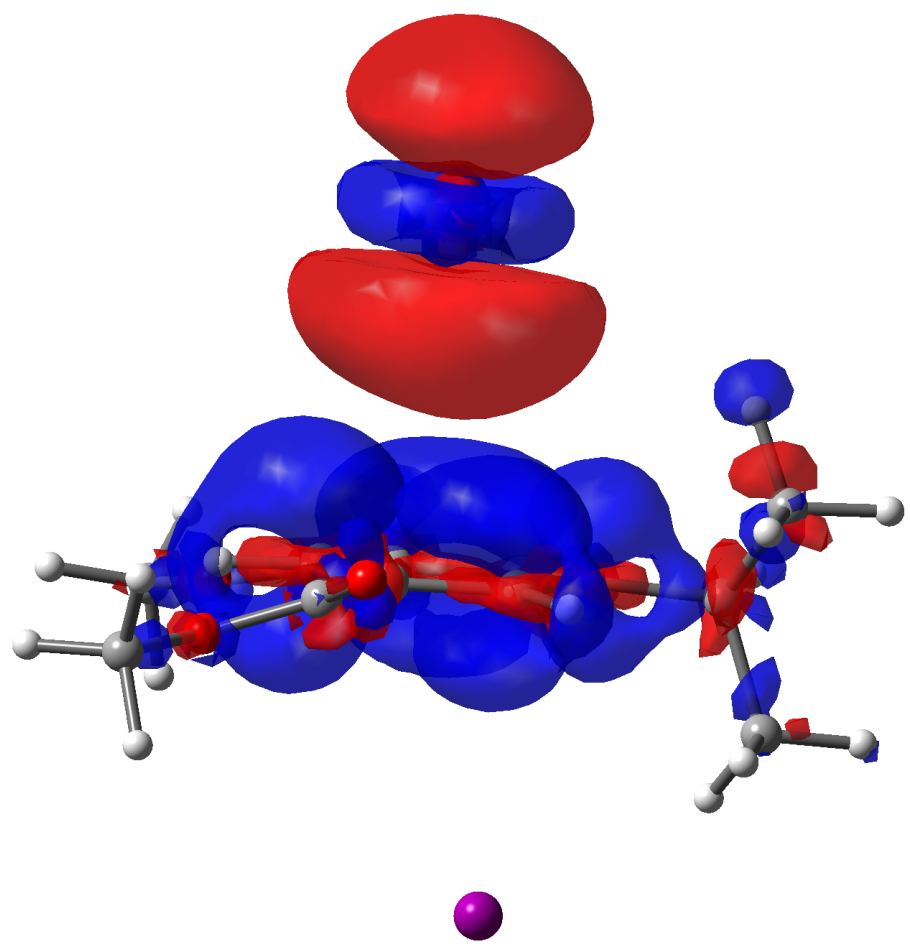
Phosphorescence ($T_2 \rightarrow S_0$)	Phosphorescence ($T_1 \rightarrow S_0$)
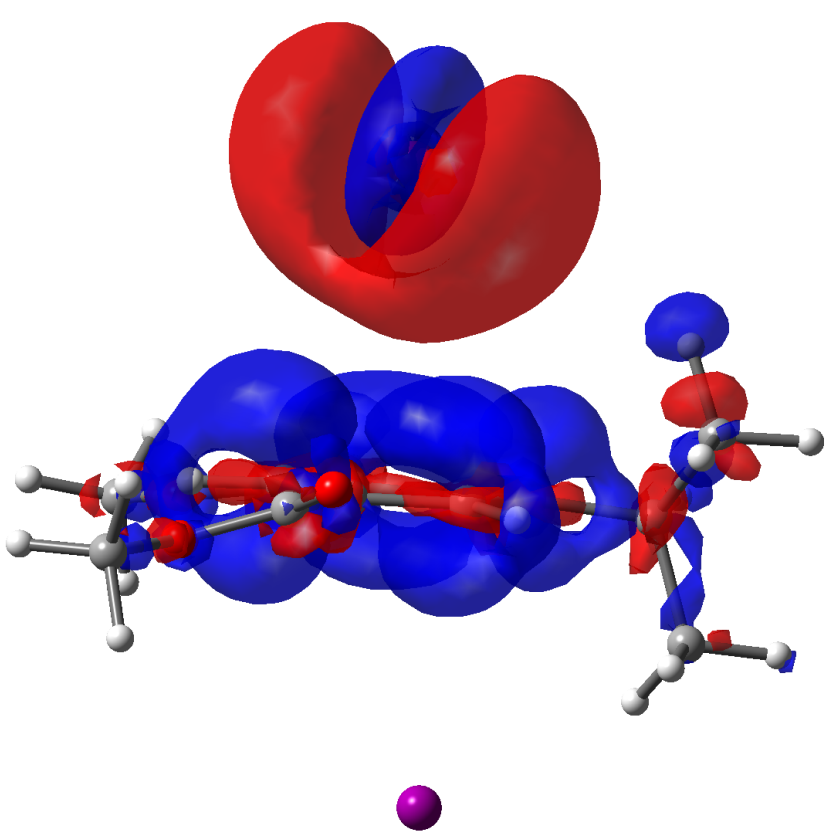	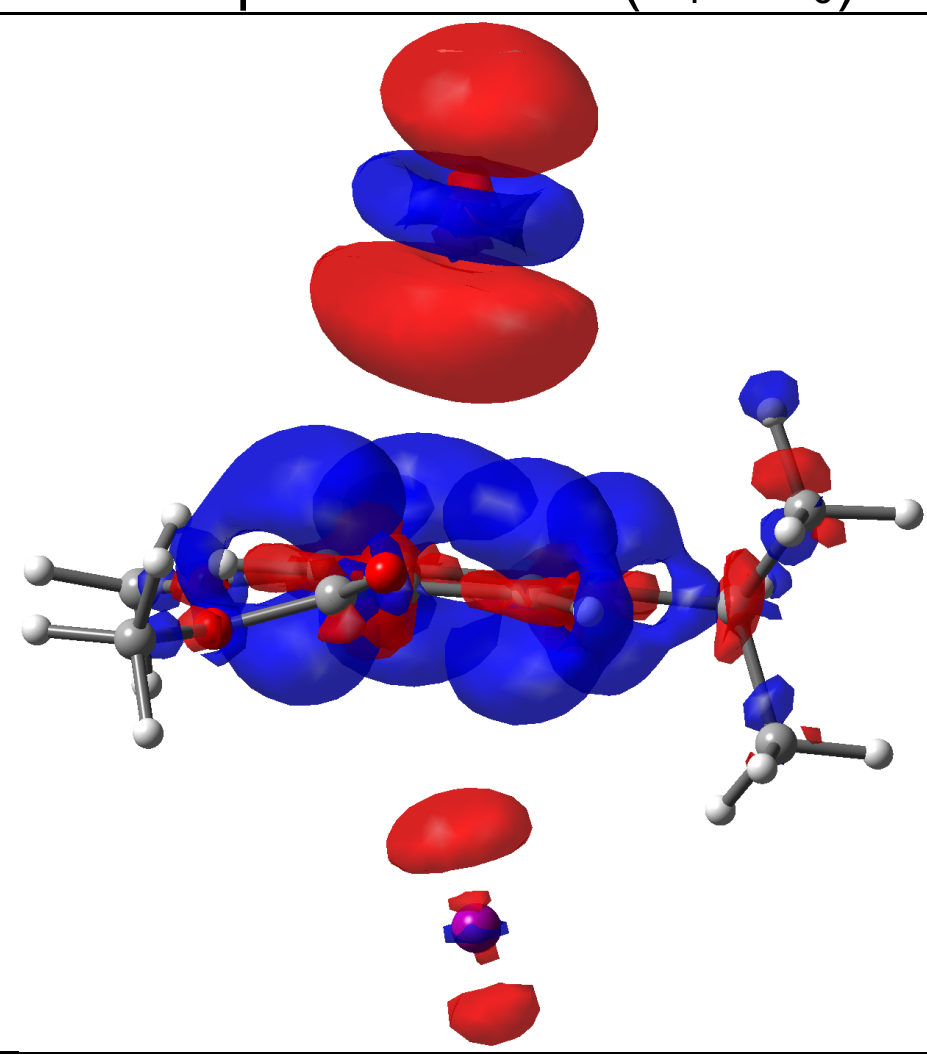

Table S19. Calculated oscillator strengths (*f*), transition energies (E), radiative rate constants (k_r) of phosphorescence, energy differences between the first excited states ($\Delta E(S_1–T_1)/\Delta E(S_1–T_2)$), spin-orbit coupling matrix elements ($<S_1|H_{SO}|T_1>/<S_1|H_{SO}|T_2>$) between singlet and triplet states and intersystem crossing rates (k_{ISC}) for **1–4** in Pyr^+ --- I^- and I^- --- Pyr^+ --- I^- configurations.

Conf.	Pyr^+ --- I^-				I^- --- Pyr^+ --- I^-		
Sample	**1**	**2**	**3**	**4**	**1**	**2**	**4**
**Excitation $S_0 \rightarrow S_1$**							
f	0.003	0.0014	0.0193	0.0092	0.0031	0.0014	0.0013
E (eV)	2.76	2.71	3.05	2.80	2.78	2.78	2.72
λ^{abs} (nm)	449	457	406	443	446	446	456
λ^{exp} (nm)	475	480	470	485	–	–	–
Character	CT						
**Fluorescence $S_1 \rightarrow S_0$**[a]							
f	0.0003	0.0005	0.0061	0.0016	0.0003	0.0005	0.001
E (eV)	2.18	2.02	2.28	2.10	2.13	2.08	2.18
λ (nm)	568	615	545	590	582	596	569
k_{ISC} (s^{-1})	2.1×10^{11}	–	–	2.1×10^{9}	–	–	–
Character	CT						
**Phosphorescence $T_1 \rightarrow S_0$**[b]							
$\Delta E(S_1-T_1)$ (eV)	0.07	0.13	0.11	0.17	0.11	0.14	0.14
E (eV)	2.12	1.89	2.17	1.93	2.02	1.94	2.04
λ (nm)	588	656	571	642	615	639	607
$\lambda_{Em,\ exp,\ 297\ K}$ (nm)	540	555	575	605	–	–	–
$\lambda_{Em,\ exp,\ 77\ K}$ (nm)	560	565	540	612	–	–	–
$<S_1\|H_{SO}\|T_1>$ (cm^{-1})[a]	482	456	176	518	507	452	133
k_r (s^{-1})	2.4×10^{2}	6.8×10^{2}	3.0×10^{2}	1.9×10^{3}	9.6×10^{2}	1.0×10^{3}	1.5×10^{5}
Character	CT						
**Phosphorescence $T_2 \rightarrow S_0$**[c]							
E (eV)	2.19	2.00	2.37	2.15	2.17	2.06	2.29
λ (nm)	566	619	523	576	571	601	541
$\lambda_{Em,\ exp,\ 297\ K}$ (nm)	540	555	575	605	–	–	–
$\lambda_{Em,\ exp,\ 77\ K}$ (nm)	560	565	540	612	–	–	–
k_r (s^{-1})	2.0×10^{4}	1.5×10^{4}	1.1×10^{4}	1.2×10^{4}	3.8×10^{4}	2.5×10^{4}	8.3×10^{3}
$\Delta E(S_1-T_2)$ (eV)[d]	-0.01	0.02	-0.09	-0.05	-0.04	0.02	-0.11
$\Delta E(T_1-T_2)$ (eV)[d]	0.08	0.11	0.20	0.22	0.15	0.12	0.25
$<S_1\|H_{SO}\|T_2>$ (cm^{-1})[a]	2085	2075	2125	2053	2057	2072	2065
Character	CT						

[a] – at optimized S_1 state geometry; [b] – at optimized T_1 state geometry; [c] – at optimized T_2 state geometry. [d] – adiabatic energy difference.

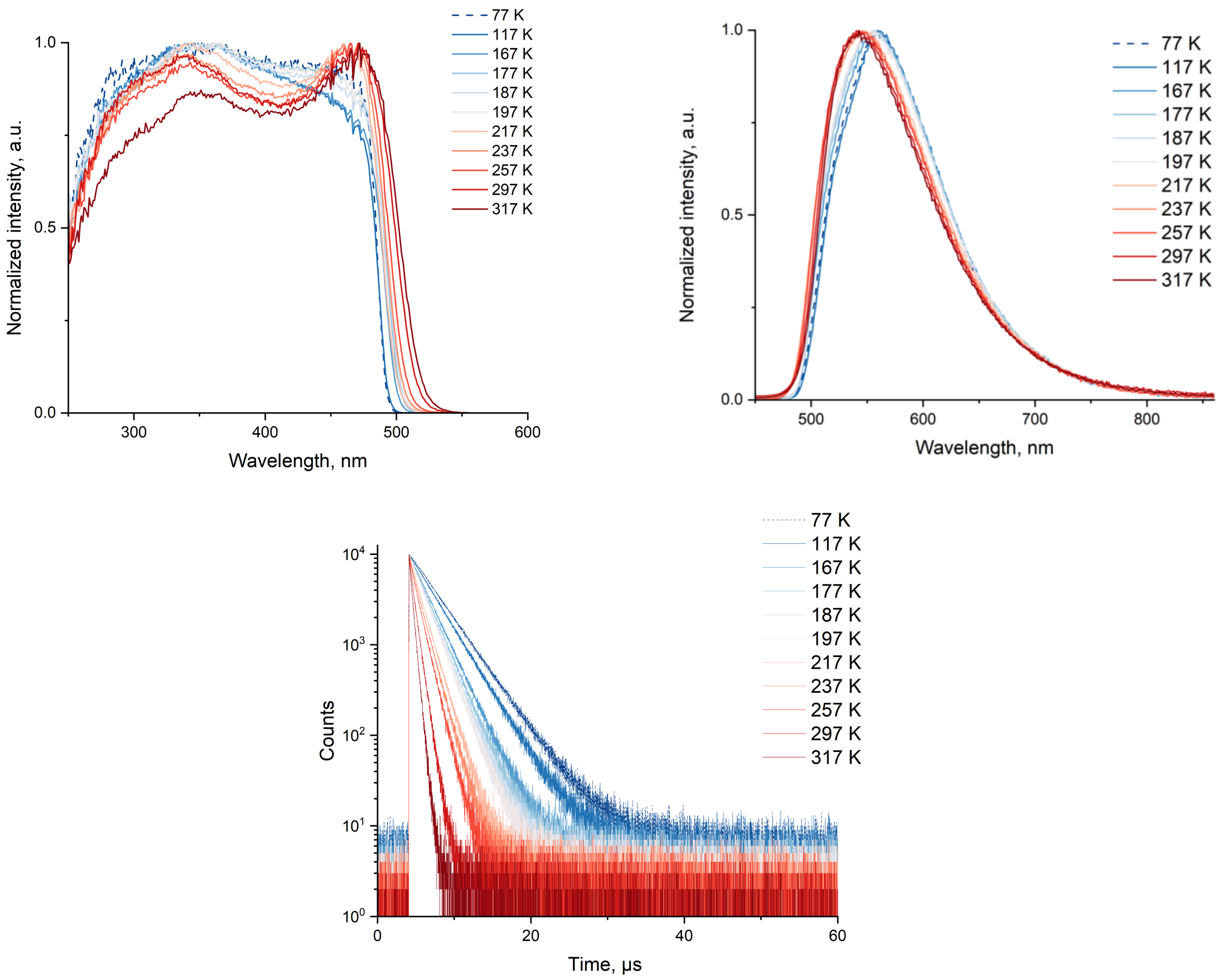

Figure S27. VT steady-state (excitation and emission) and time-resolved emission studies of crystalline **1**.

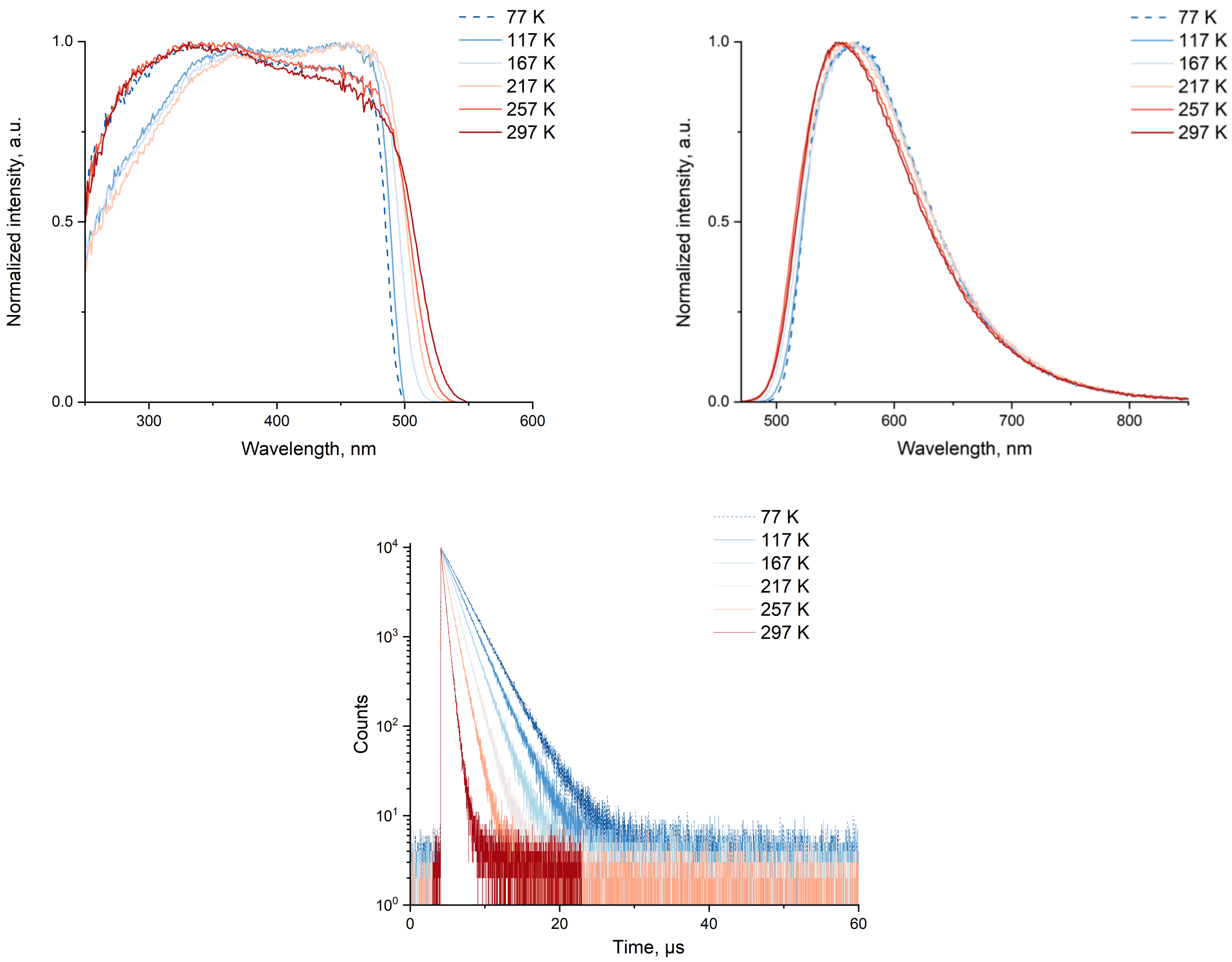

Figure S28. VT steady-state (excitation and emission) and time-resolved emission studies of crystalline **2**.

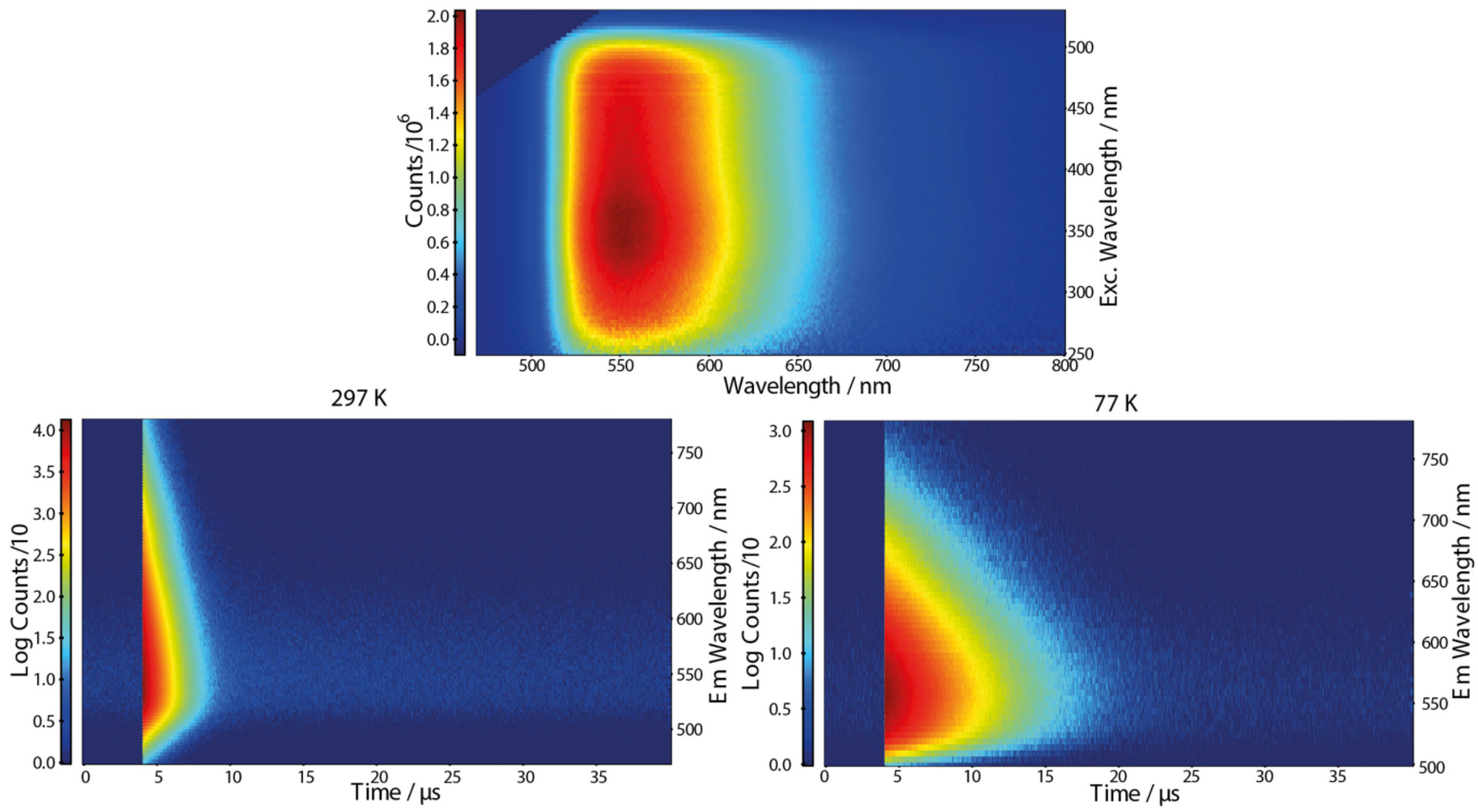

Figure S29. Excitation and emission mapping plot and time-resolved emission spectra of crystalline **2** at 297/77 K.

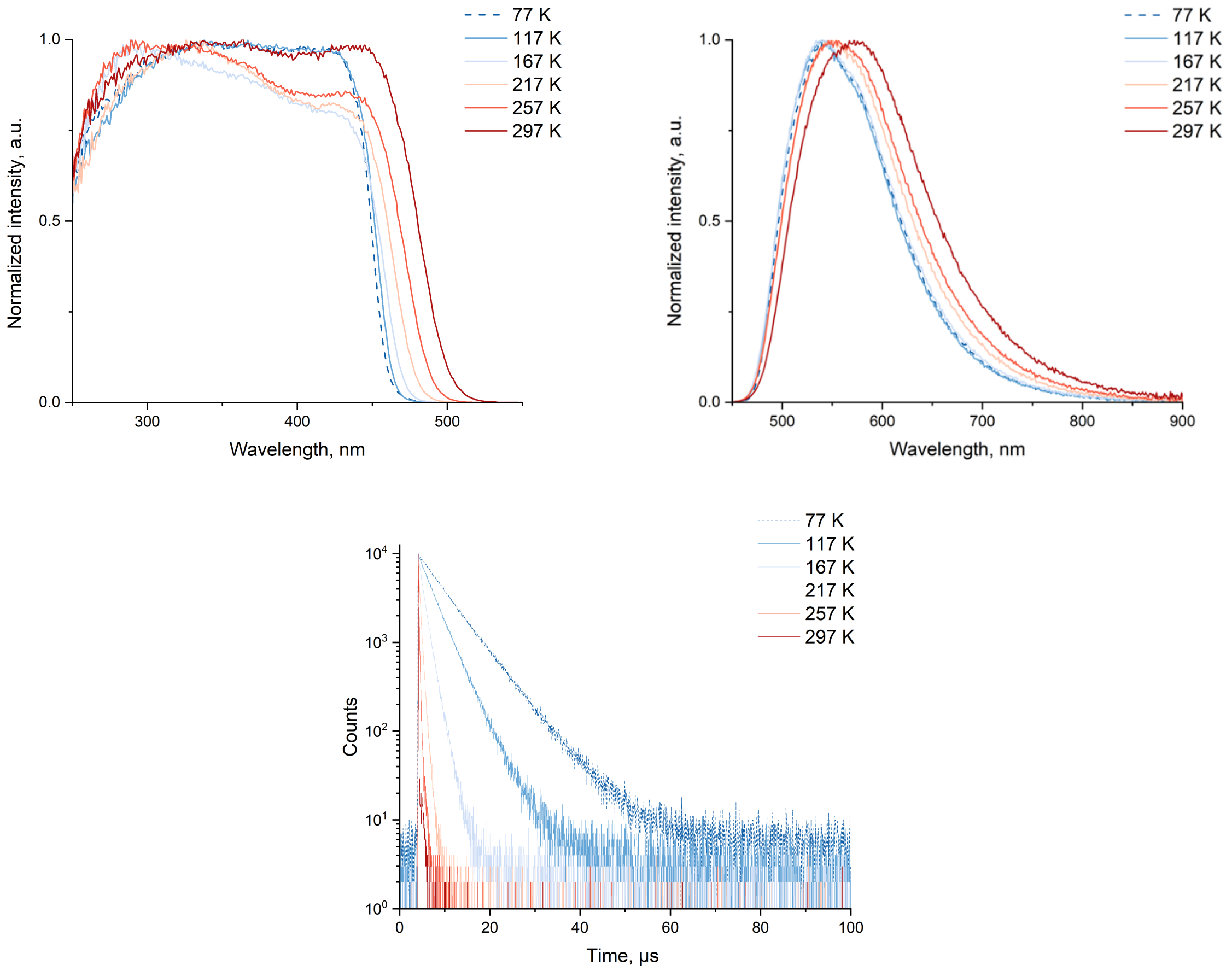

Figure S30. VT steady-state (excitation and emission) and time-resolved emission studies of crystalline **3**.

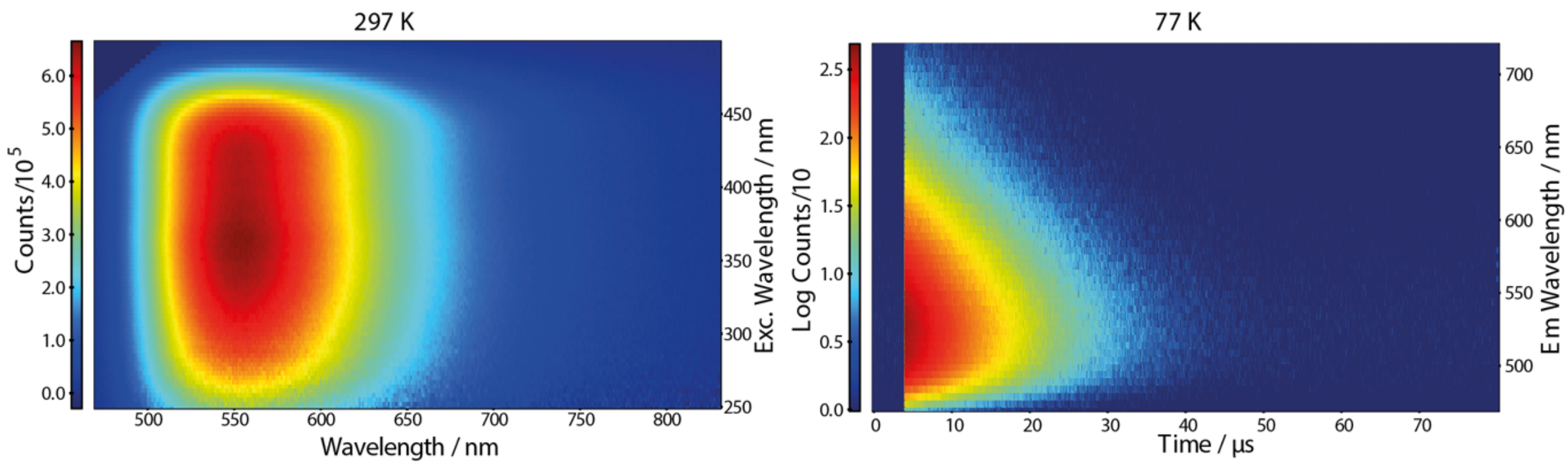

Figure S31. Excitation and emission mapping plot (297 K) and time-resolved emission spectra (77 K) of crystalline **3**.

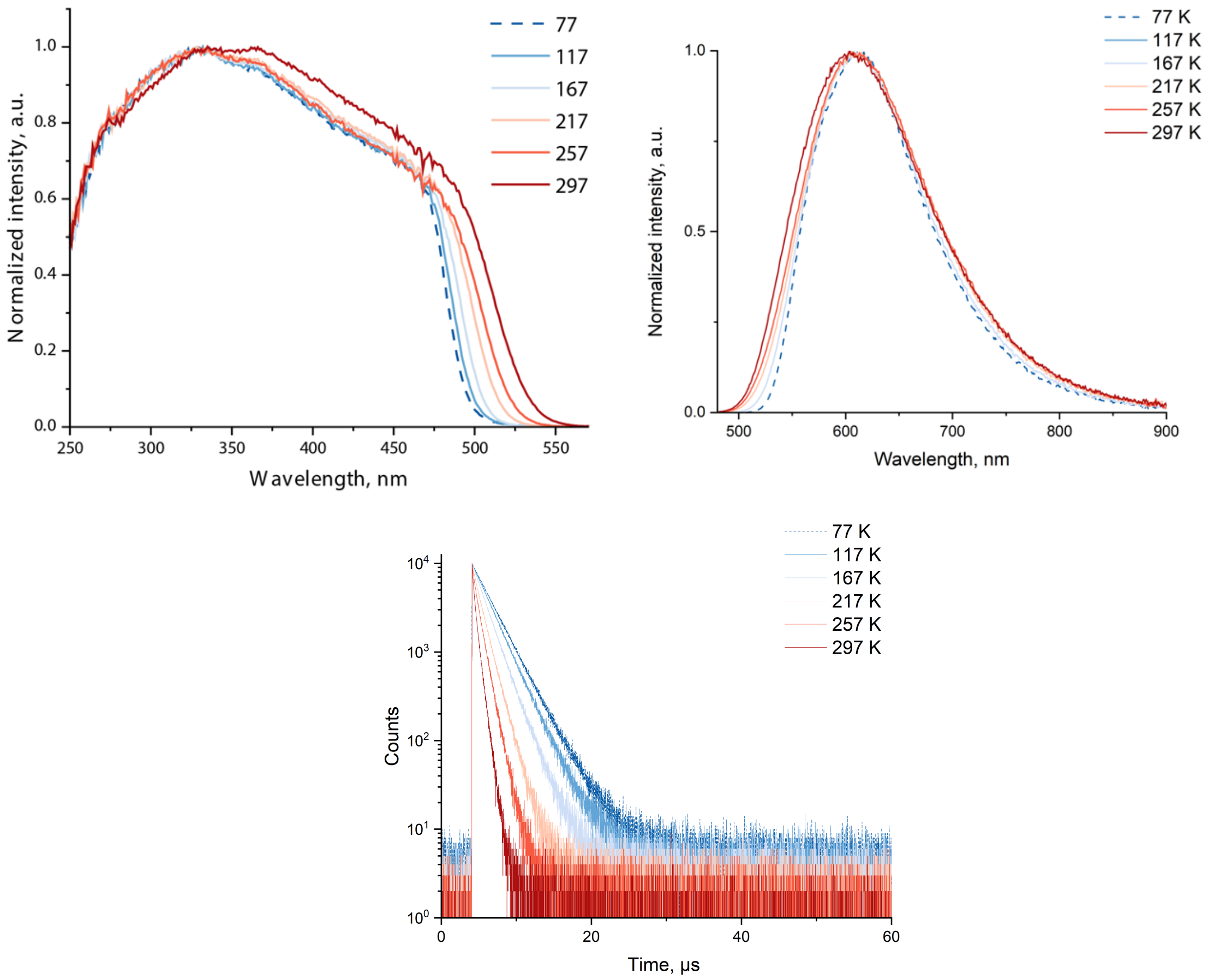

Figure S32. VT steady-state (excitation and emission) and time-resolved emission studies of crystalline **4**.

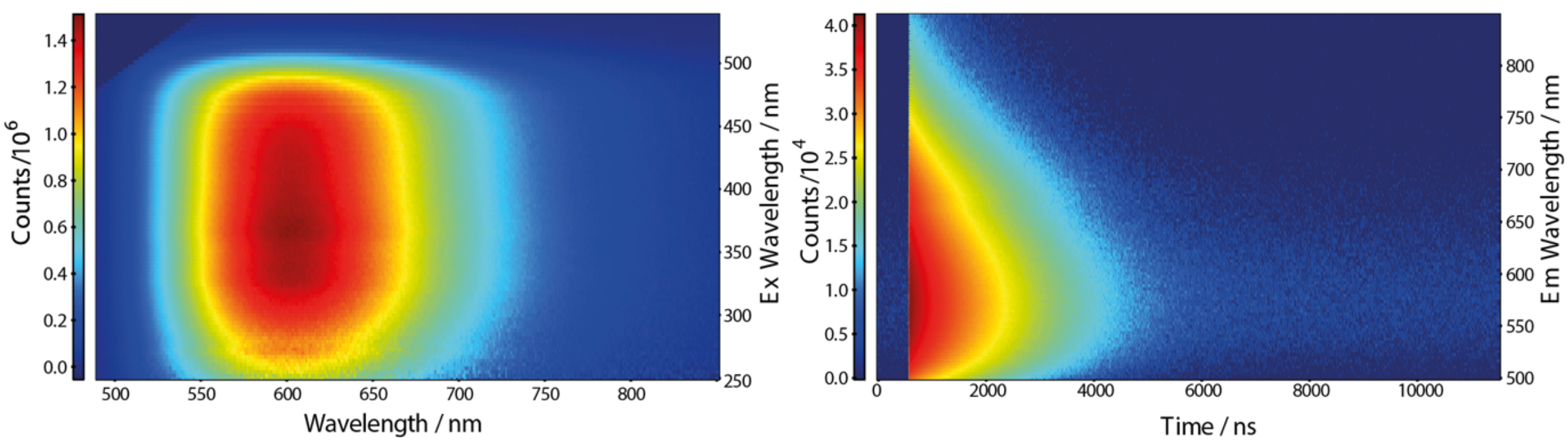

Figure S33. Excitation and emission mapping plot and time-resolved emission spectra of crystalline **4** at 297 K.

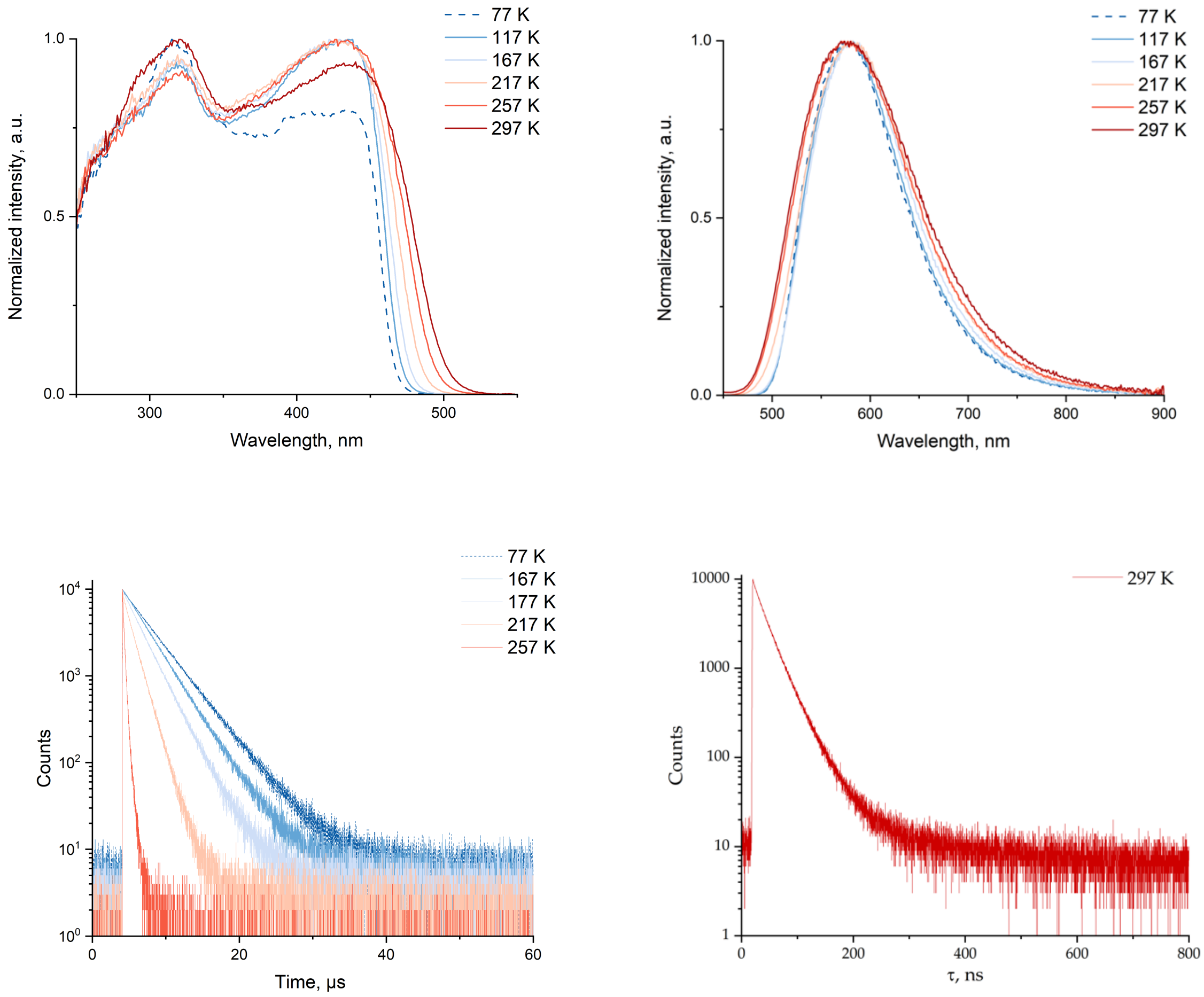

Figure S34. VT steady-state (excitation and emission) and time-resolved emission studies of crystalline ⁻**5**. VT decays at 77–257 K were measured in MSC mode, while at 297 K in TCSPC mode.

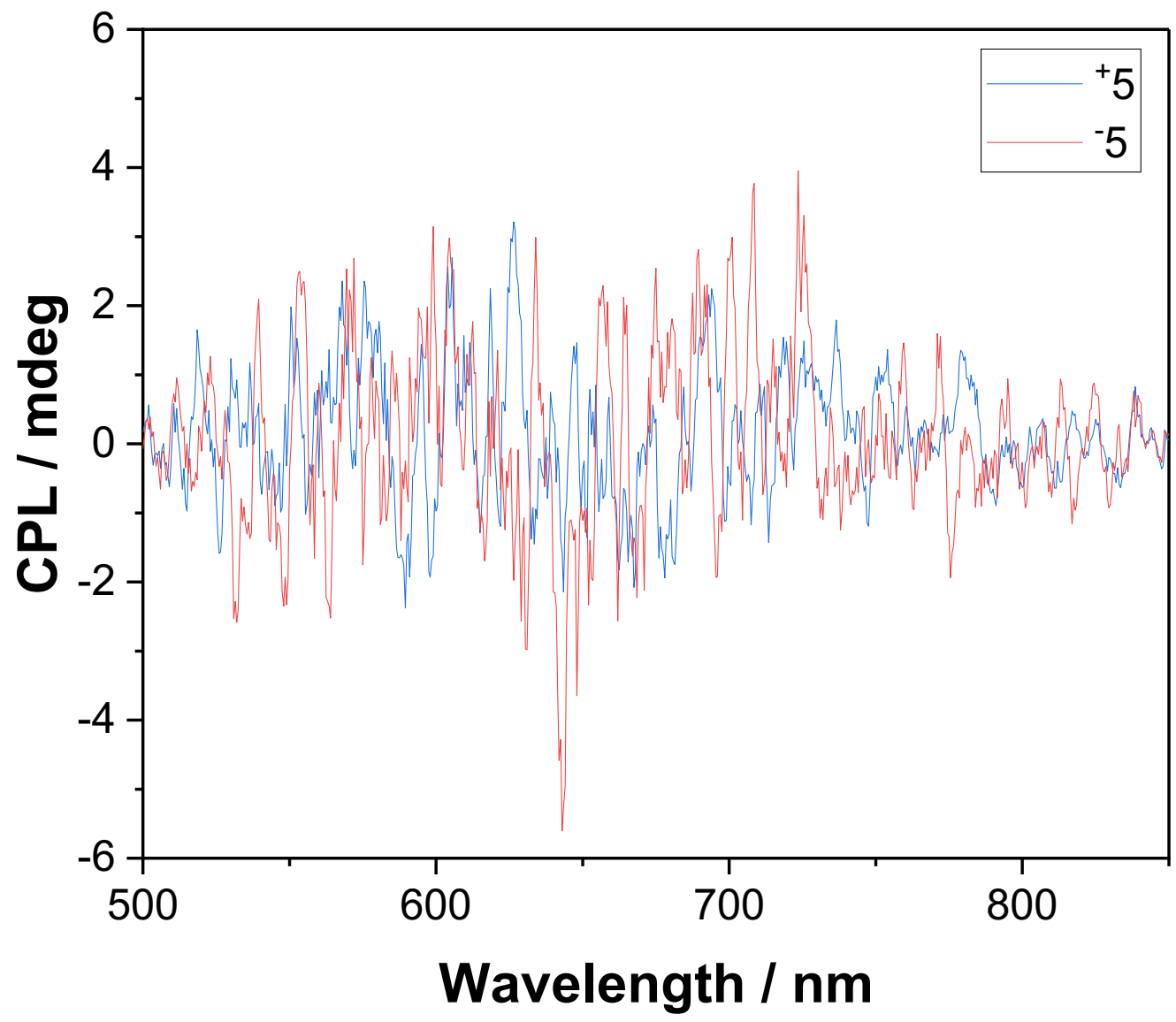

Figure S35. Solid-state CPL measurements of $^{-}$**5** and $^{+}$**5**. The results demonstrate that g_{lum} value is below 10^{-3}; correct determination cannot be performed due to the detection limit of our setup.

Table S20. Computed spin-orbit coupling matrix elements, energy differences between the lowest excited triplet state (T_1/T_2) and 10 lowest lying singlet excited states (S_n), and oscillator strengths (*f*) for the spin-allowed singlet-singlet transitions for **1**.

1

	T_1 -> S_0			T_2 -> S_0		
n	$\Delta E(S_n-T_1)$ [eV]	$\langle S_n \| Hso \| T_1 \rangle$ [cm^{-1}]	$f(S_n$->$S_0)$	$\Delta E(S_n-T_2)$ [eV]	$\langle S_n \| Hso \| T_2 \rangle$ [cm^{-1}]	$f(S_n$->$S_0)$
1	0.39	1881	0.0045	-0.01	1984	0.0017
2	0.44	1974	0.0009	0.04	865	0.0037
3	0.67	616	0.1574	0.10	2063	0.0026
4	1.12	32	0.0199	0.67	58	0.0020
5	1.25	45	0.0030	0.73	36	0.0167
6	1.30	73	0.0159	0.75	62	0.0001
7	2.60	13	0.0007	2.57	25	0.0058
8	2.89	3	0.0003	2.78	72	0.0328
9	2.98	100	0.0219	2.87	58	0.0090
10	3.28	17	0.0563	2.90	20	0.0114

Table S21. Computed spin-orbit coupling matrix elements, energy differences between the lowest excited triplet state (T_1/T_2) and 10 lowest lying singlet excited states (S_n), and oscillator strengths (*f*) for the spin-allowed singlet-singlet transitions for **2**.

2

	T_1 -> S_0			T_2 -> S_0		
n	$\Delta E(S_n-T_1)$ [eV]	$\langle S_n\|Hso\|T_1\rangle$ [cm^{-1}]	$f(S_n$->$S_0)$	$\Delta E(S_n-T_2)$ [eV]	$\langle S_n\|Hso\|T_2\rangle$ [cm^{-1}]	$f(S_n$->$S_0)$
1	0.46	1775	0.0090	0.00	2057	0.0012
2	0.48	1957	0.0001	0.05	1749	0.0001
3	0.74	811	0.1735	0.11	1251	0.0176
4	1.16	51	0.0143	0.81	14	0.0032
5	1.34	26	0.0020	0.84	49	0.0012
6	1.36	92	0.0260	0.87	37	0.0054
7	2.69	2	0.0034	2.68	99	0.0320
8	2.94	9	0.0024	2.83	80	0.0076
9	3.05	66	0.0086	2.87	32	0.0049
10	3.26	7	0.0554	2.93	45	0.0038

Table S22. Computed spin-orbit coupling matrix elements, energy differences between the lowest excited triplet state (T_1/T_2) and 10 lowest lying singlet excited states (S_n), and oscillator strengths (*f*) for the spin-allowed singlet-singlet transitions for **3**.

**3**	T_1 -> S_0			T_2 -> S_0		
n	$\Delta E(S_n-T_1)$ [eV]	$\langle S_n\|Hso\|T_1\rangle$ [cm^{-1}]	$f(S_n\text{->}S_0)$	$\Delta E(S_n-T_2)$ [eV]	$\langle S_n\|Hso\|T_2\rangle$ [cm^{-1}]	$f(S_n\text{->}S_0)$
1	0.38	2020	0.0002	-0.06	2118	0.0271
2	0.47	1179	0.0952	0.01	113	0.0002
3	0.53	1633	0.0715	0.14	2133	0.0044
4	1.10	21	0.0571	0.73	114	0.0322
5	1.24	68	0.0092	0.82	26	0.0030
6	1.35	58	0.0000	0.93	41	0.0027
7	2.34	6	0.0002	2.20	28	0.0041
8	2.63	3	0.0023	2.26	23	0.0045
9	2.71	14	0.0377	2.44	143	0.0466
10	2.90	19	0.0086	2.67	12	0.0001

Table S23. Computed spin-orbit coupling matrix elements, energy differences between the lowest excited triplet state (T_1/T_2) and 10 lowest lying singlet excited states (S_n), and oscillator strengths (*f*) for the spin-allowed singlet-singlet transitions for **4**.

**4**	T_1 -> S_0			T_2 -> S_0		
n	$\Delta E(S_n-T_1)$ [eV]	$\langle S_n\|Hso\|T_1\rangle$ [cm^{-1}]	$f(S_n\text{->}S_0)$	$\Delta E(S_n-T_2)$ [eV]	$\langle S_n\|Hso\|T_2\rangle$ [cm^{-1}]	$f(S_n\text{->}S_0)$
1	0.24	1038	0.0158	-0.04	1986	0.0072
2	0.32	1831	0.0090	0.02	876	0.0039
3	0.35	1883	0.0095	0.07	2077	0.0045
4	0.82	83	0.0031	0.58	31	0.0016
5	0.96	36	0.0008	0.67	77	0.0007
6	1.08	92	0.0425	0.76	113	0.0211
7	2.53	11	0.0003	2.32	10	0.0011
8	2.93	51	0.0238	2.70	136	0.0391
9	2.97	31	0.0097	2.74	13	0.0008
10	3.07	42	0.0077	2.80	22	0.0027

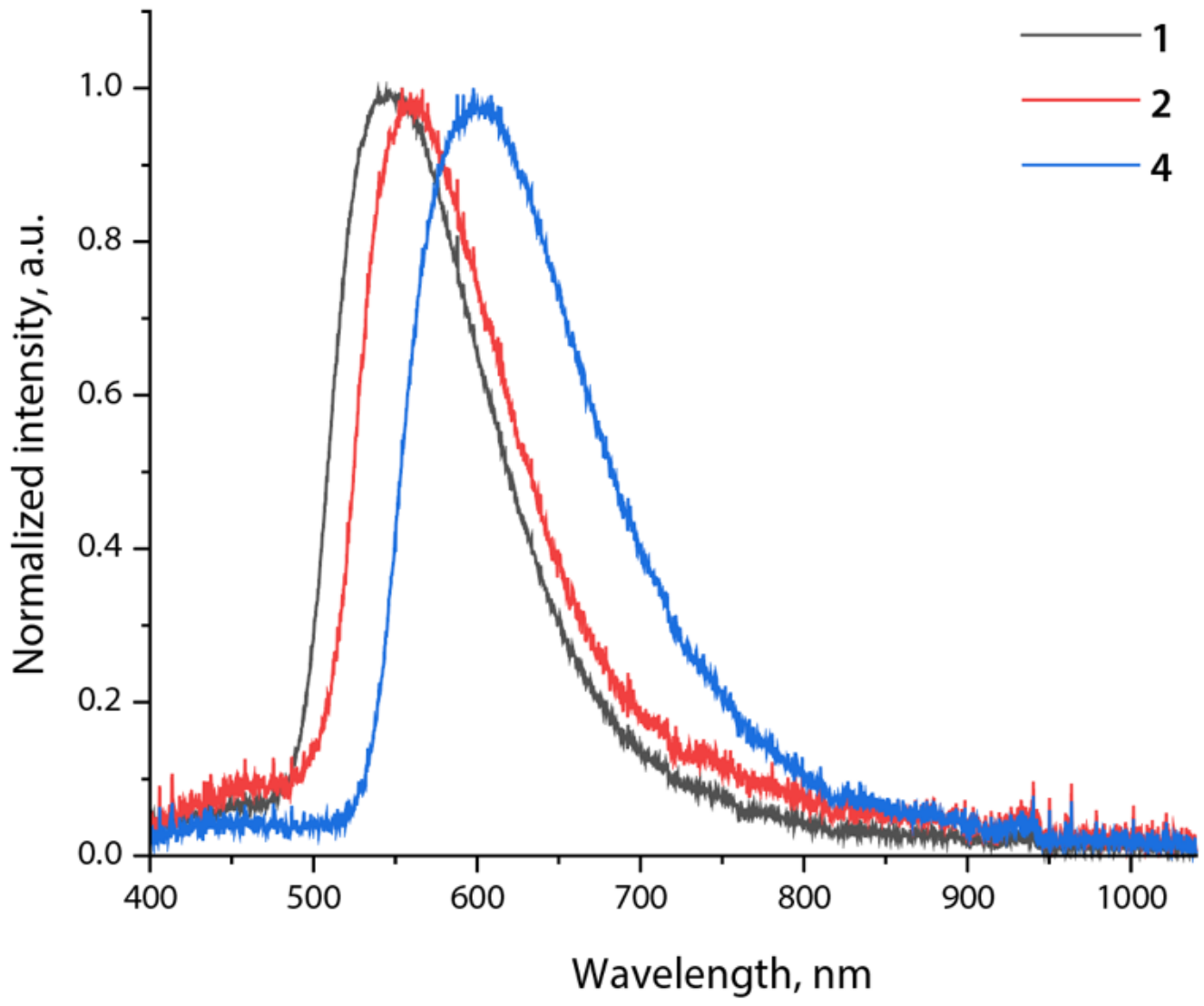

Figure S36. Radioluminescence spectra of crystalline samples **1**, **2**, and **4**.

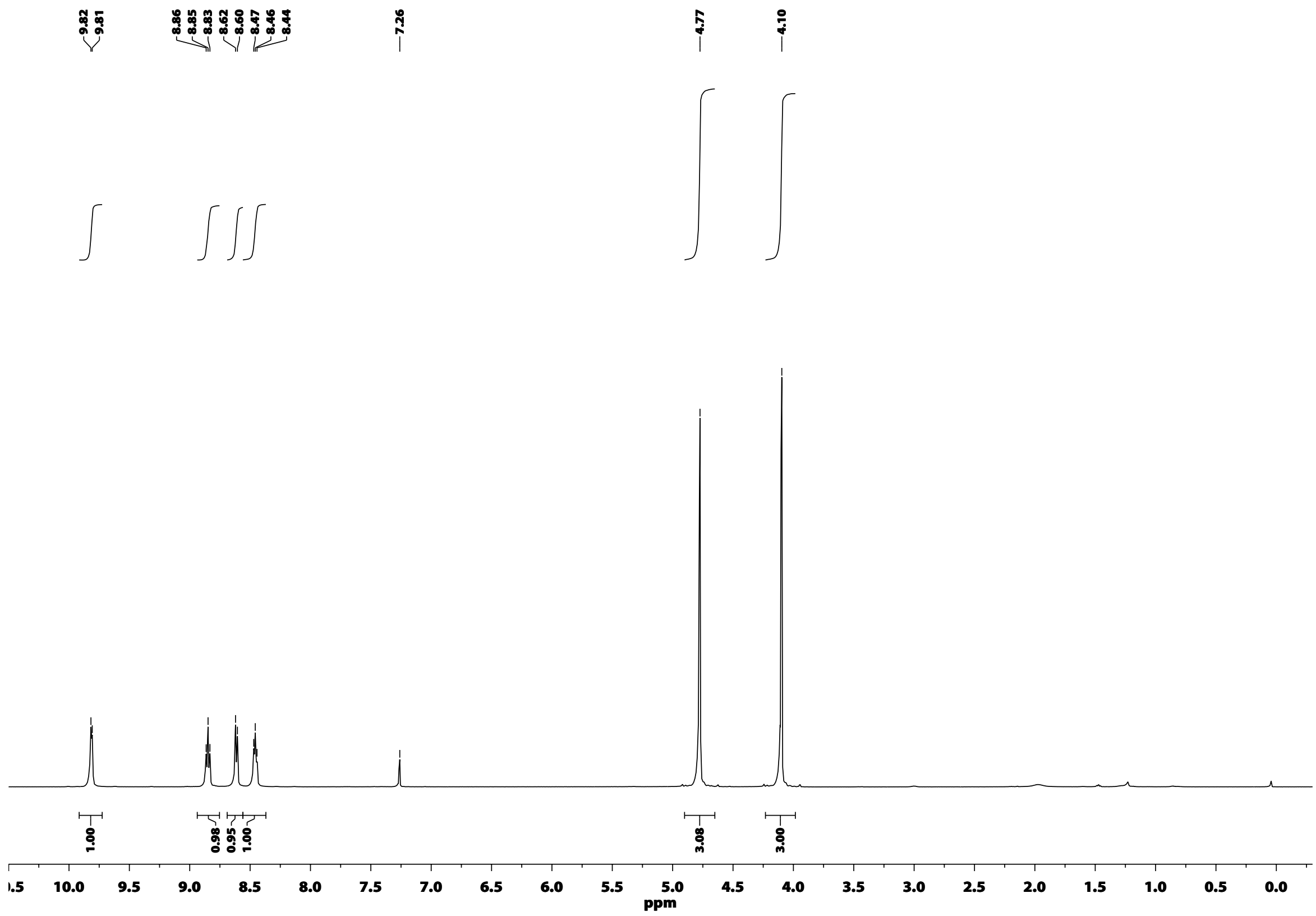

Figure S37. ^{1}H NMR spectrum of *o*-**pyMeI** ($CDCl_3$, 297 K, 500 MHz).

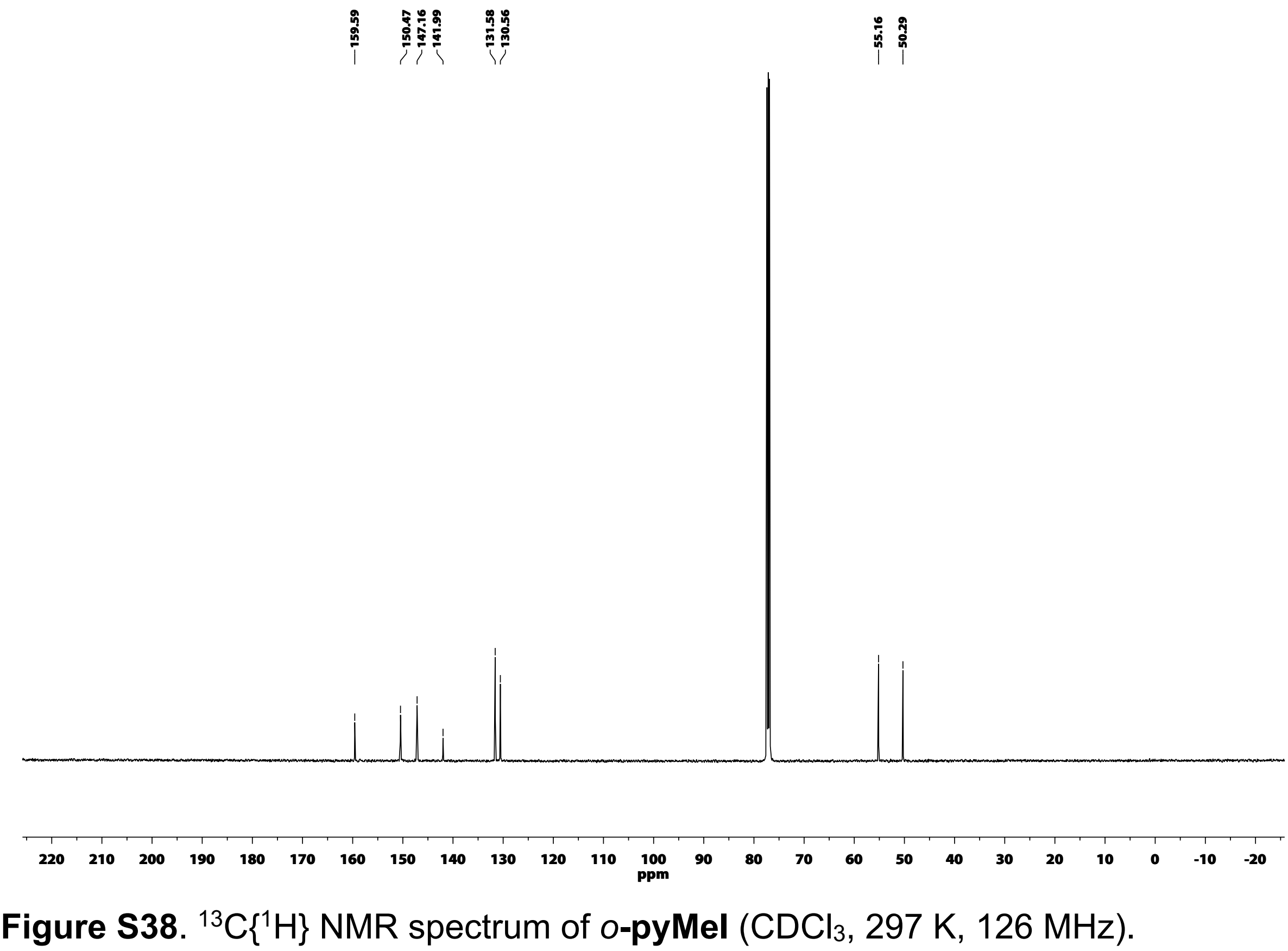

Figure S38. $^{13}C\{^{1}H\}$ NMR spectrum of *o*-**pyMeI** ($CDCl_3$, 297 K, 126 MHz).

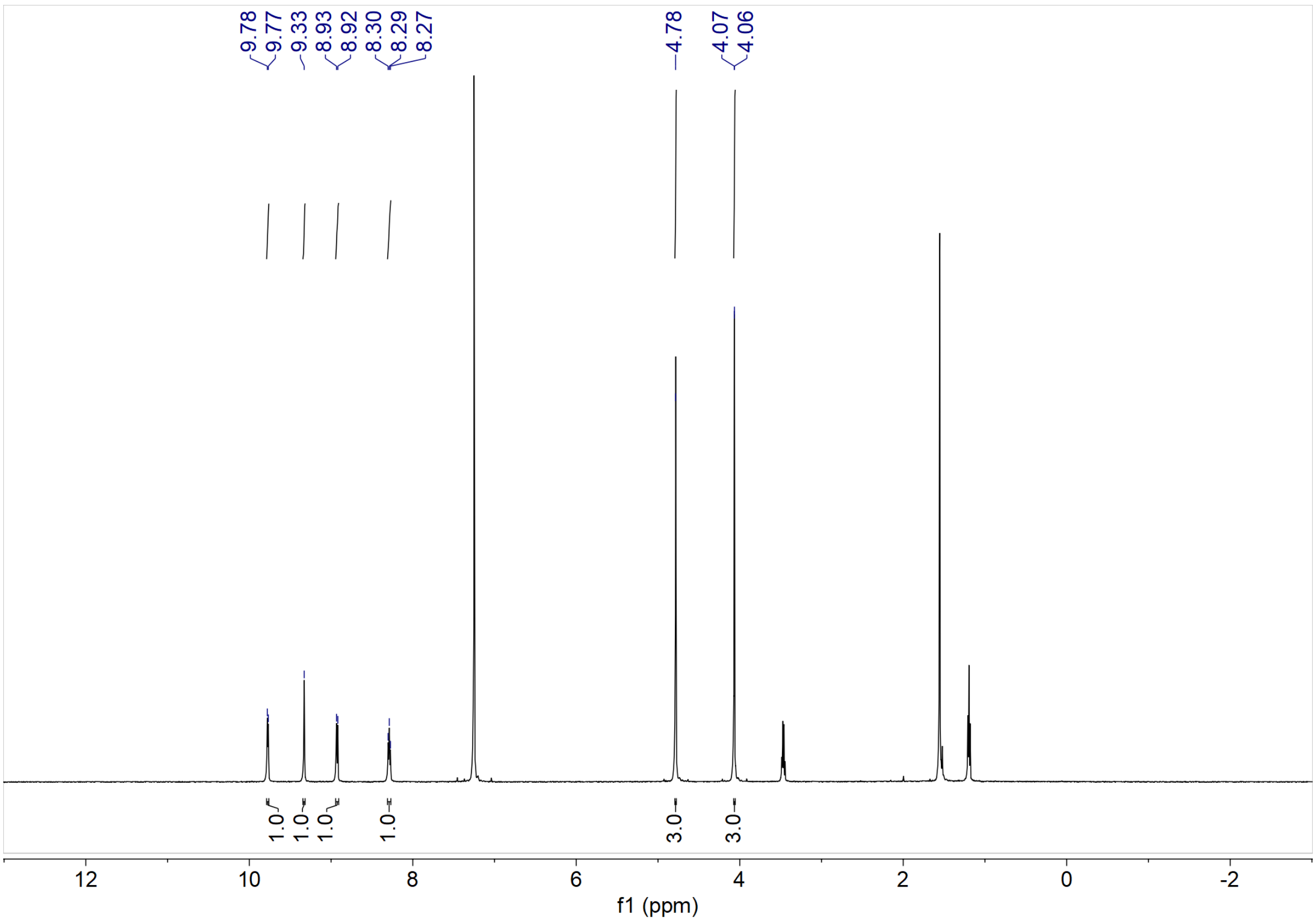

Figure S39. ^{1}H NMR spectrum of *m*-**pyMeI** ($CDCl_3$, 297 K, 500 MHz).

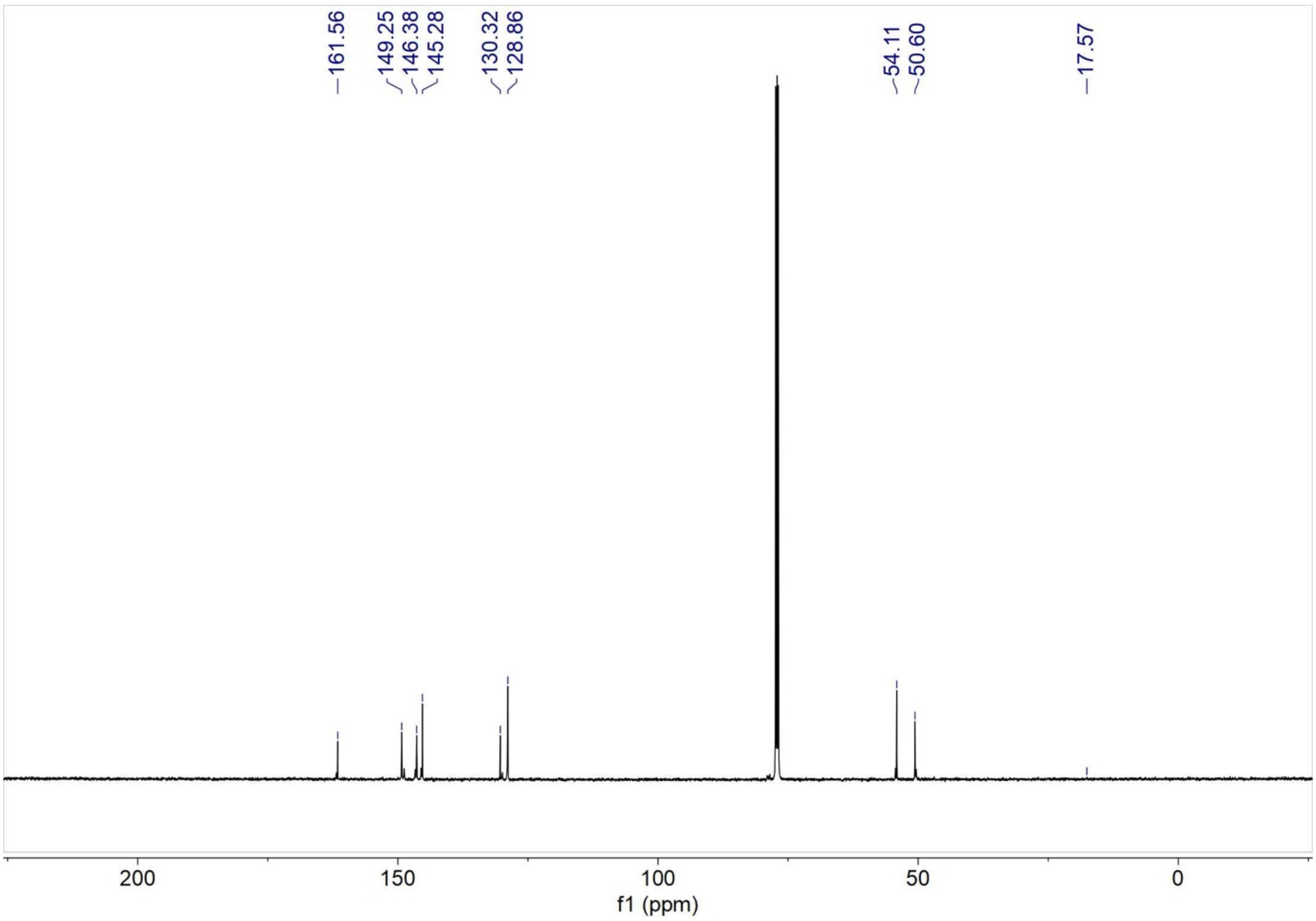

Figure S40. $^{13}C\{^{1}H\}$ NMR spectrum of *m*-**pyMeI** ($CDCl_3$, 297 K, 126 MHz).

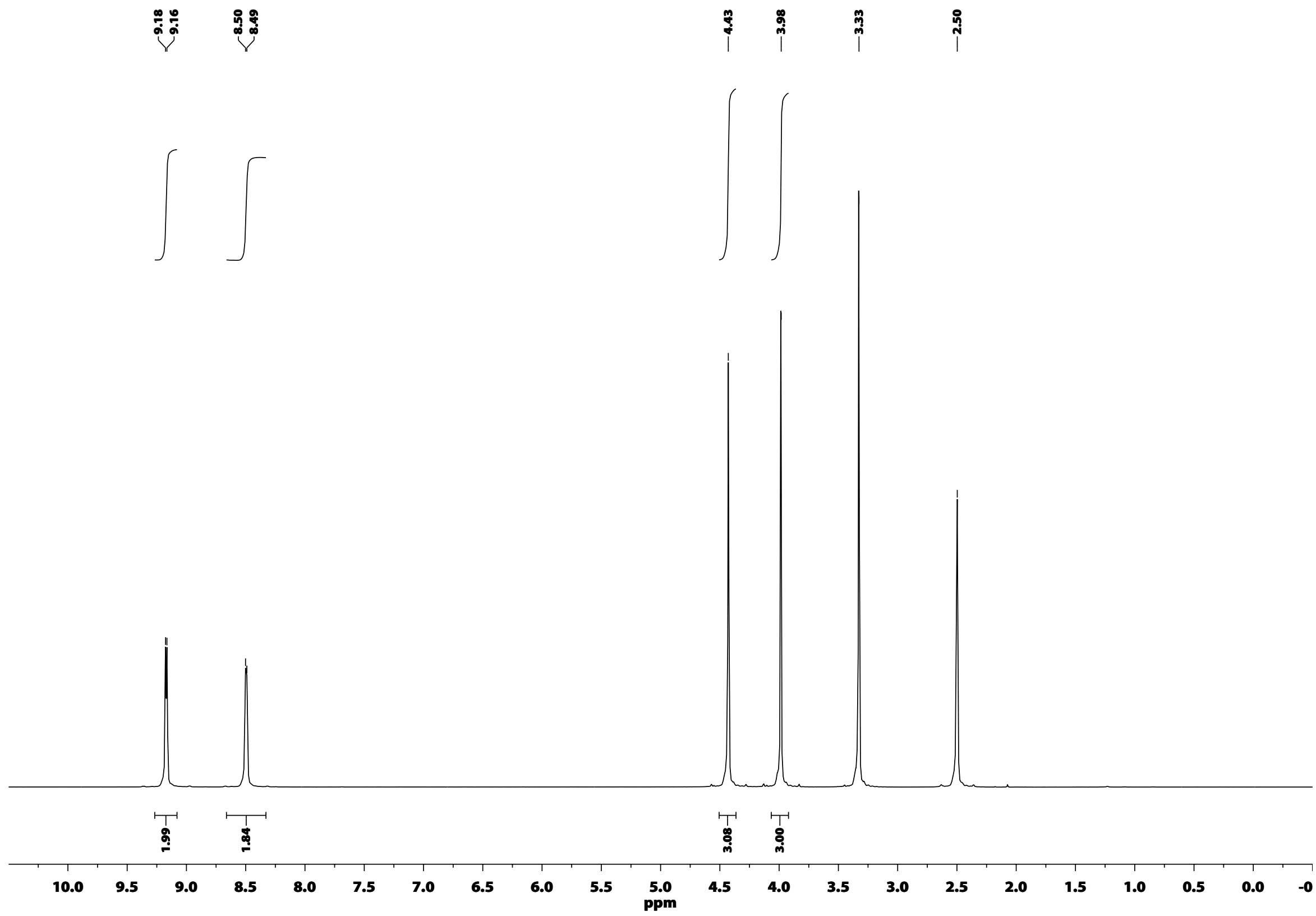

Figure S41. ^{1}H NMR spectrum of *p*-**pyMeI** (DMSO-d_6, 297 K, 500 MHz).

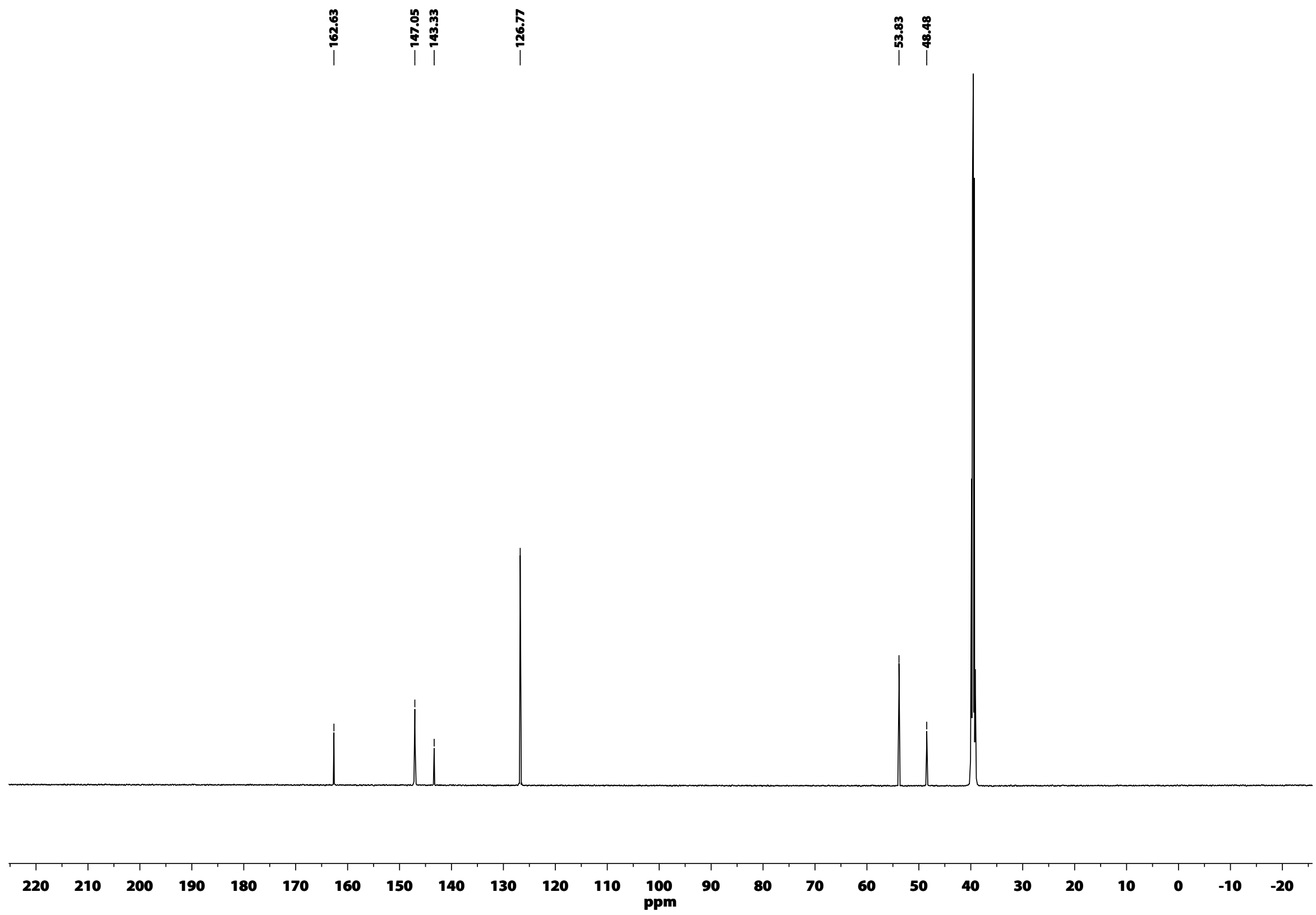

Figure S42. ^{13}C{^{1}H} NMR spectrum of *p*-**pyMeI** (DMSO-d_6, 297 K, 126 MHz).

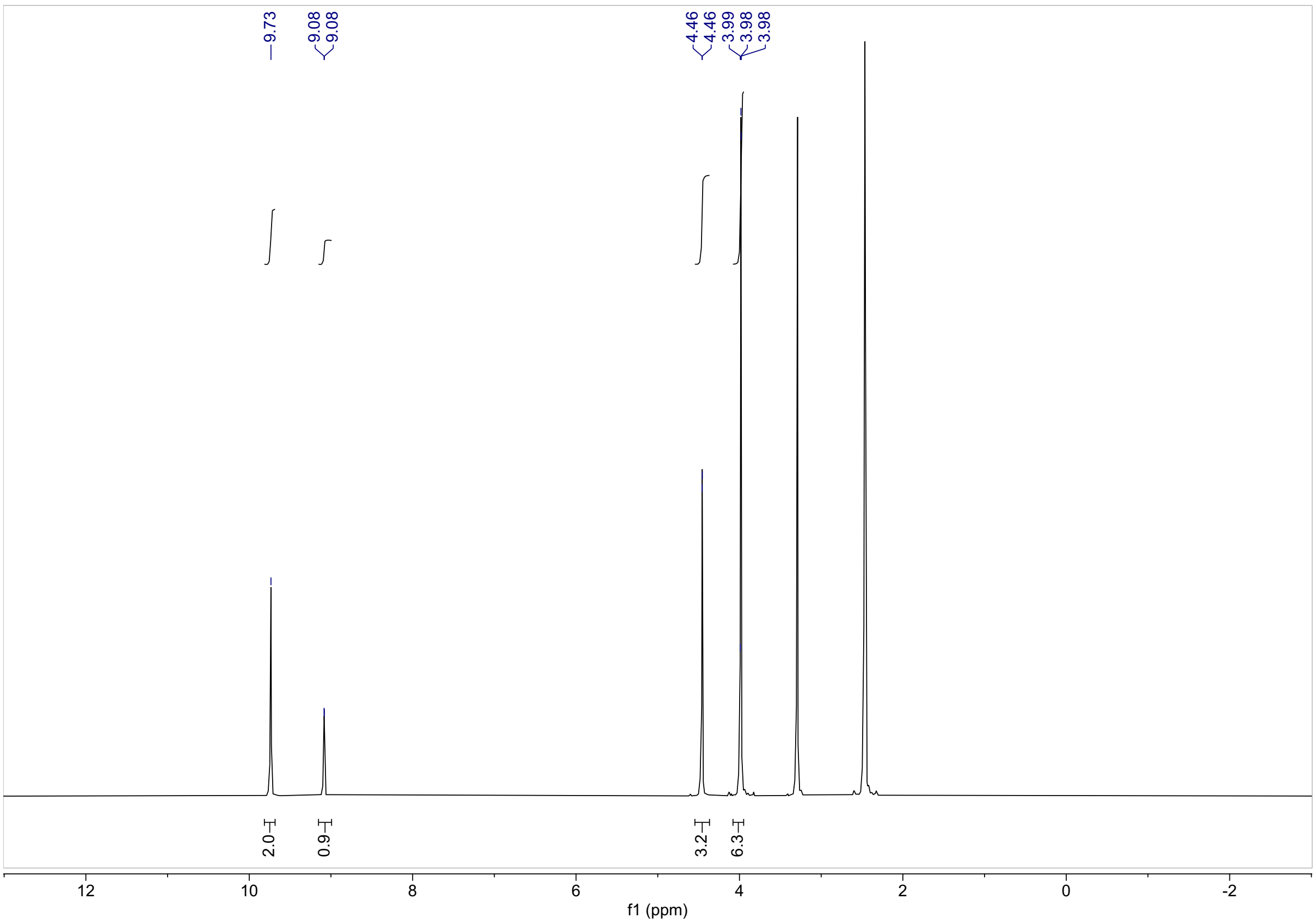

Figure S43. ^{1}H NMR spectrum of **1**^{OTf} (DMSO-d_6, 297 K, 500 MHz).

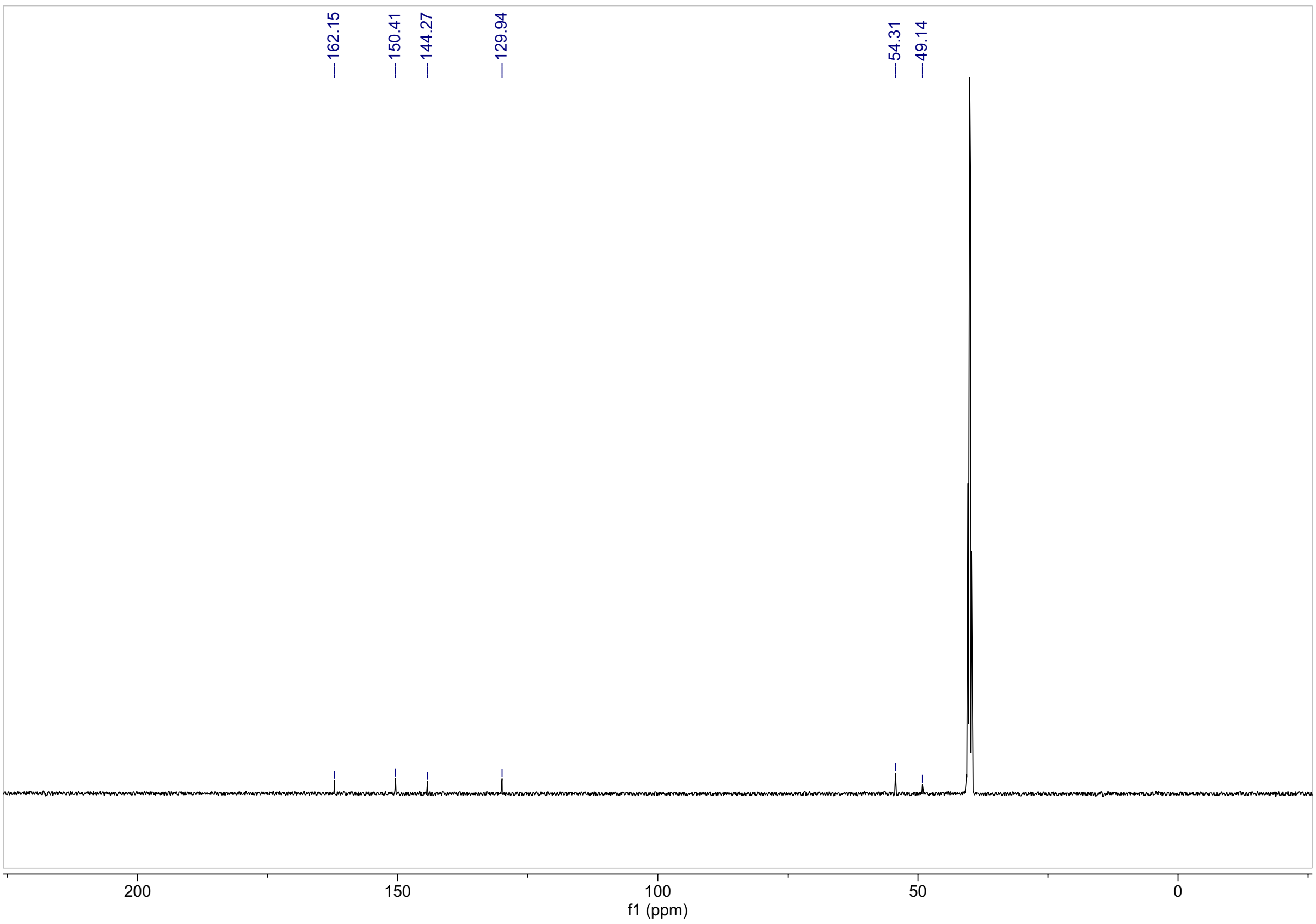

Figure S44. $^{13}C\{^{1}H\}$ NMR spectrum of **1**^{OTf} (DMSO-d_6, 297 K, 126 MHz).

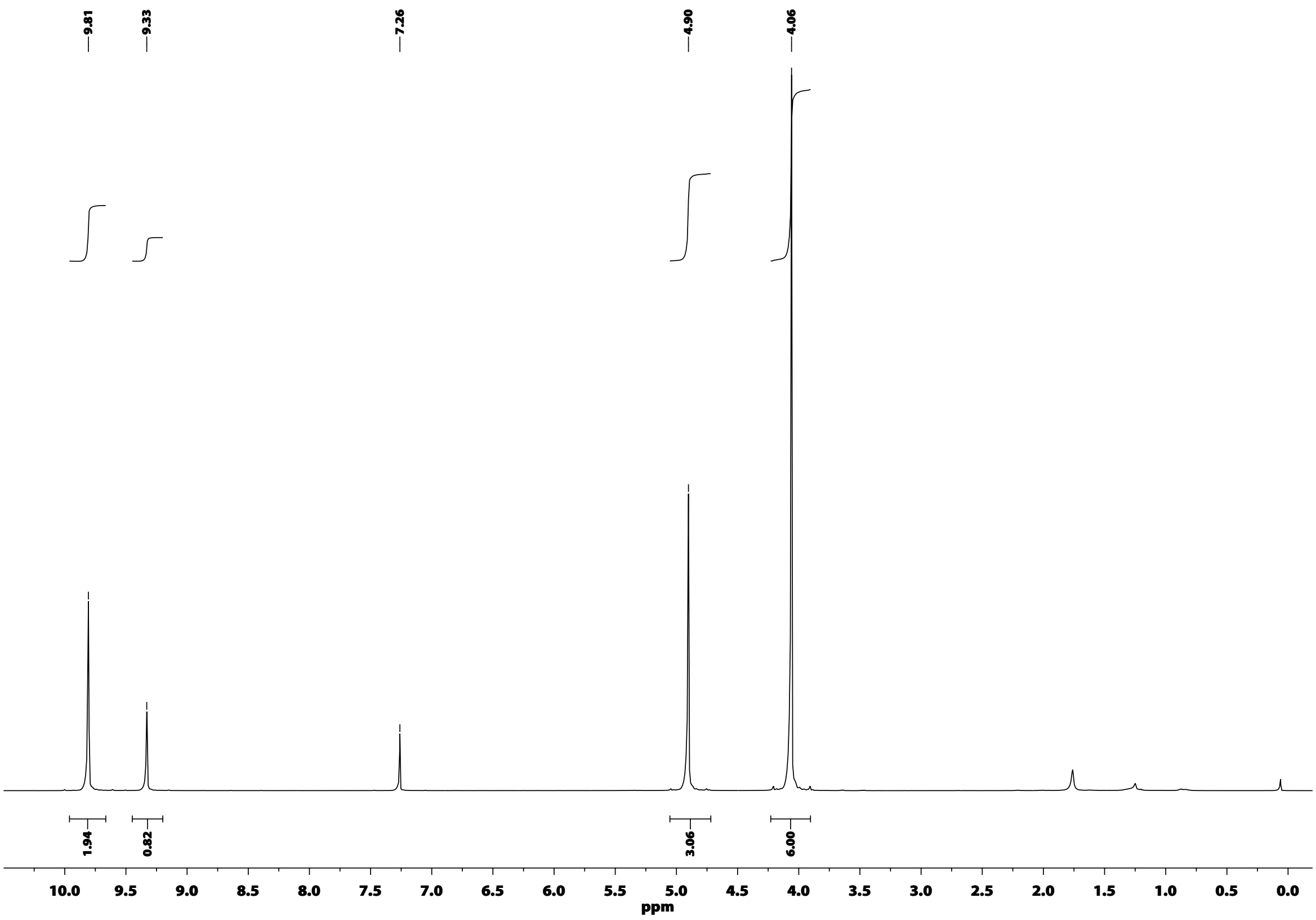

Figure S45. ^{1}H NMR spectrum of **1**^{Br} ($CDCl_3$, 297 K, 500 MHz).

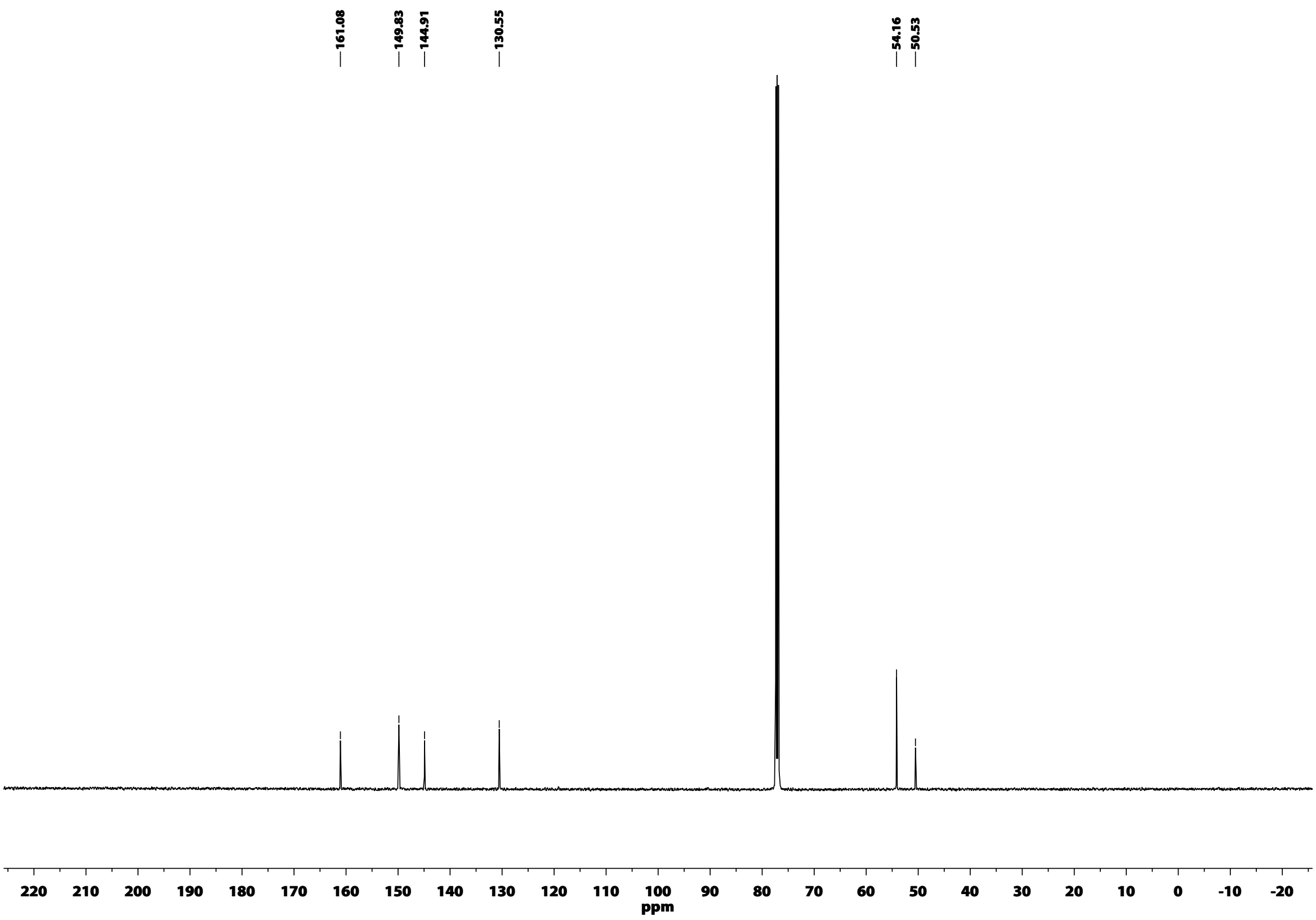

Figure S46. ^{13}C{^{1}H} NMR spectrum of **1**^{Br} ($CDCl_3$, 297 K, 126 MHz).

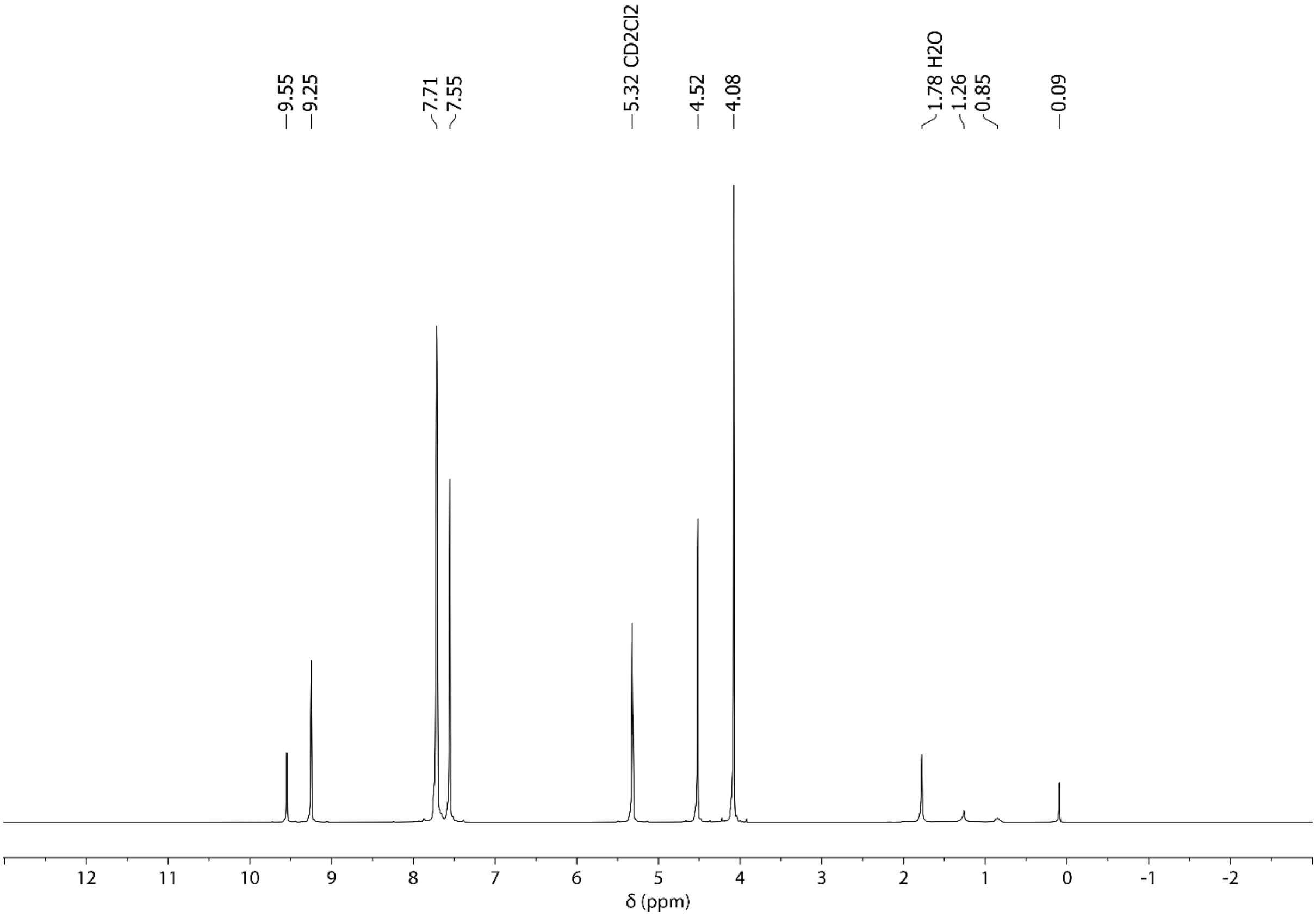

Figure S47. ^{1}H NMR spectrum of **1**^{BARF} (CD_2Cl_2, 297 K, 500 MHz).

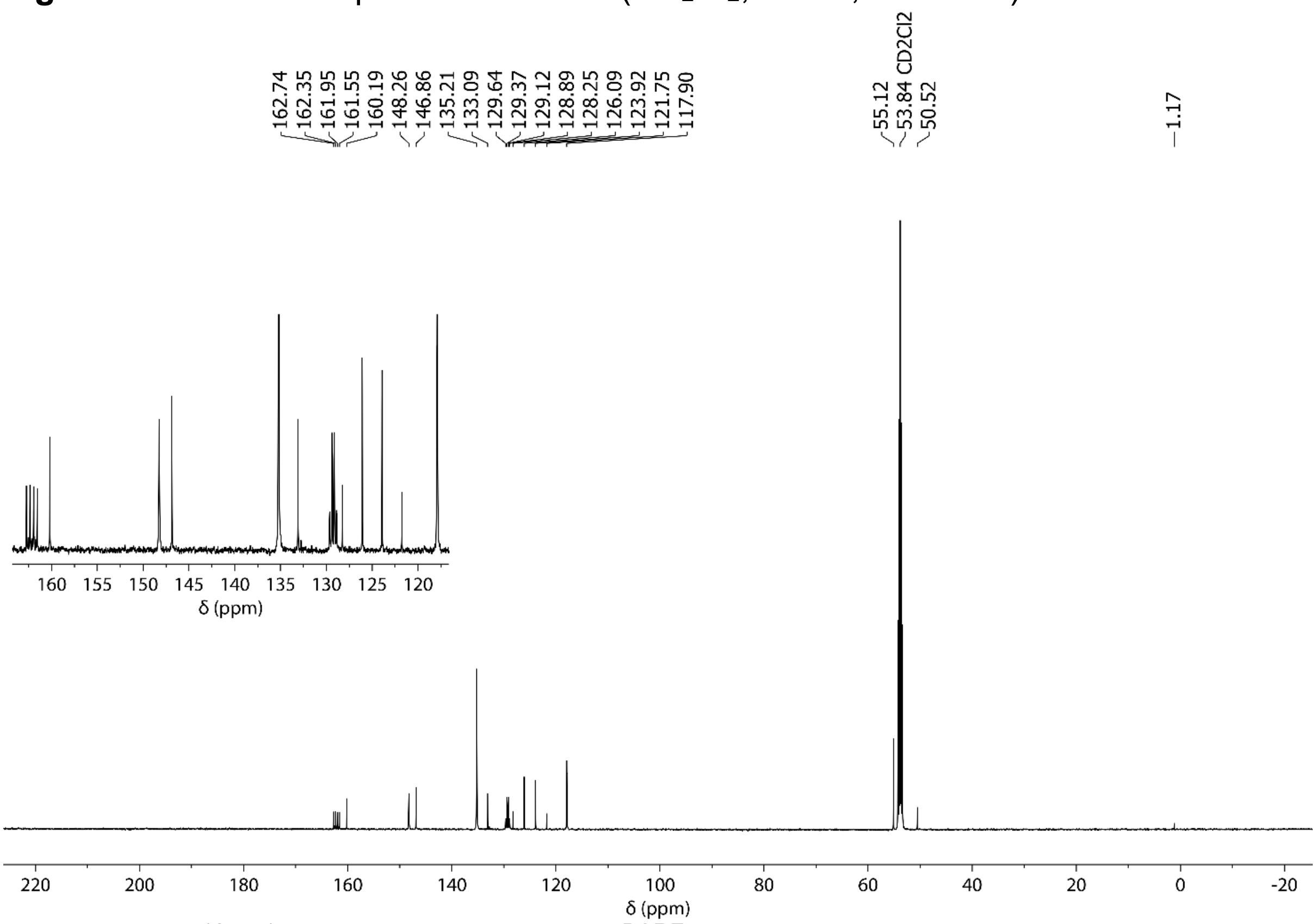

Figure S48. $^{13}C\{^{1}H\}$ NMR spectrum of **1**^{BARF} (CD_2Cl_2, 297 K, 126 MHz). Inset shows region from 162.5 ppm to 117.5 ppm.

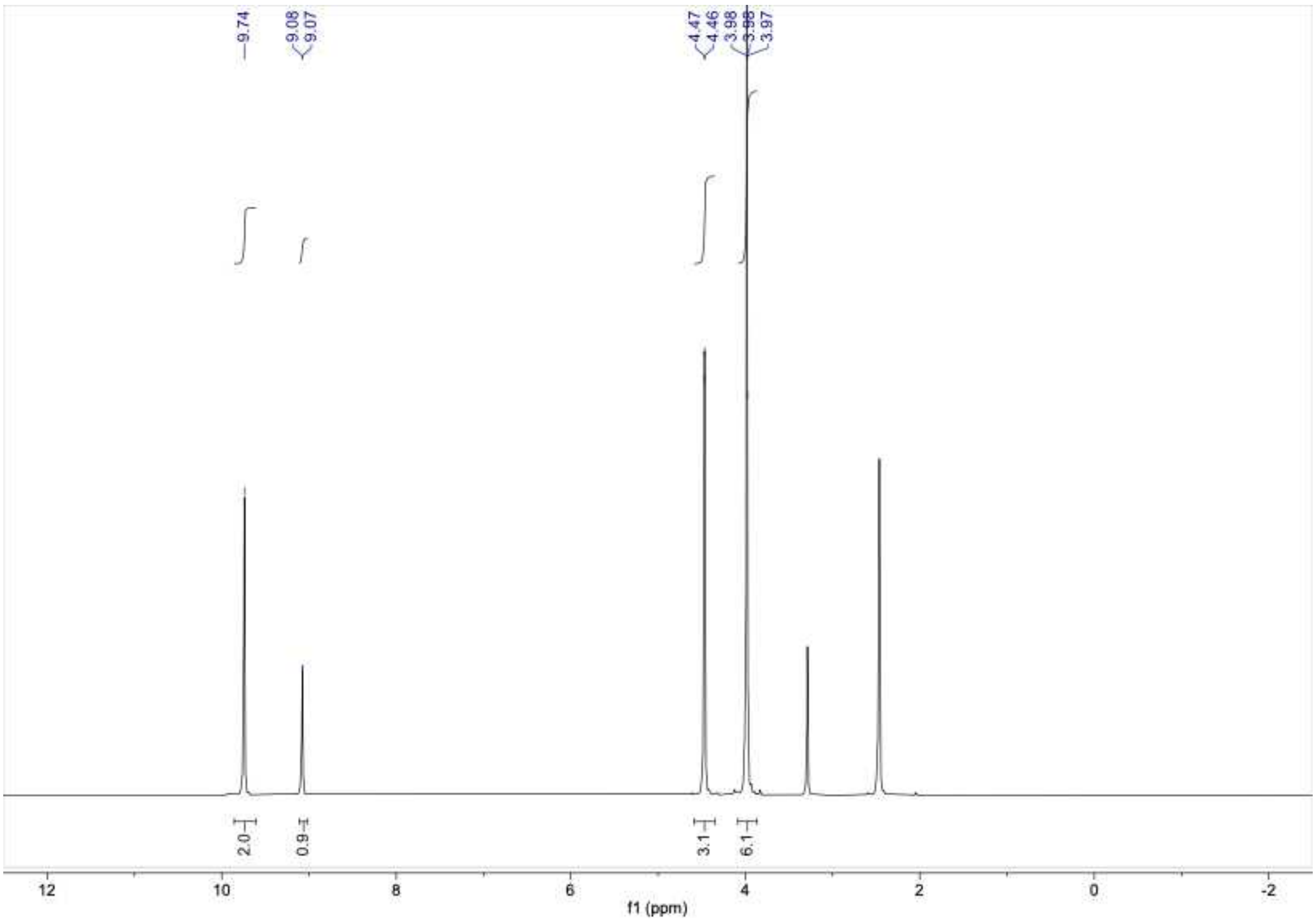

Figure S49. ^{1}H NMR spectrum of **1** (DMSO-d_6, 297 K, 500 MHz).

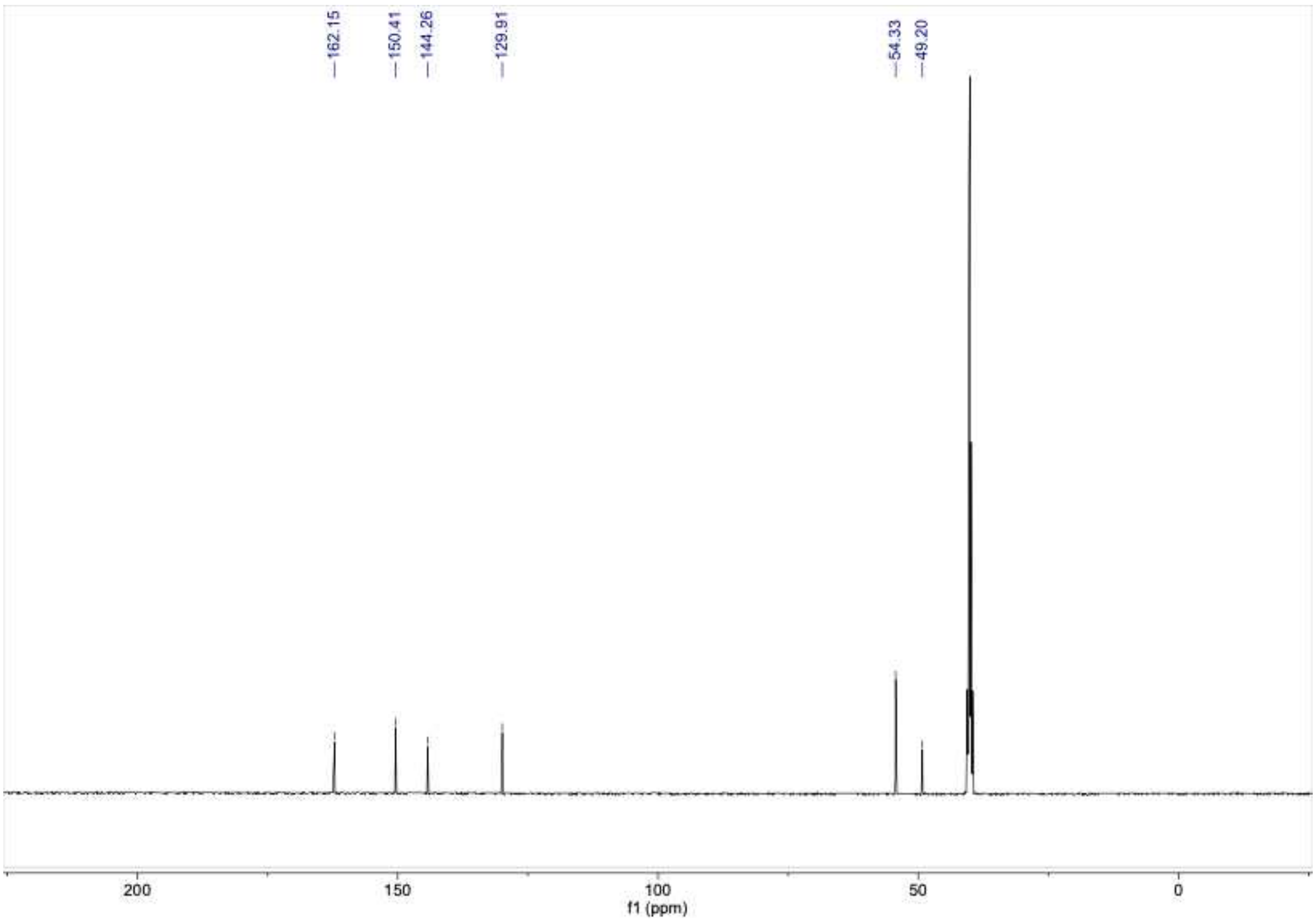

Figure S50. ^{13}C{^{1}H} NMR spectrum of **1** (DMSO-d_6, 297 K, 126 MHz).

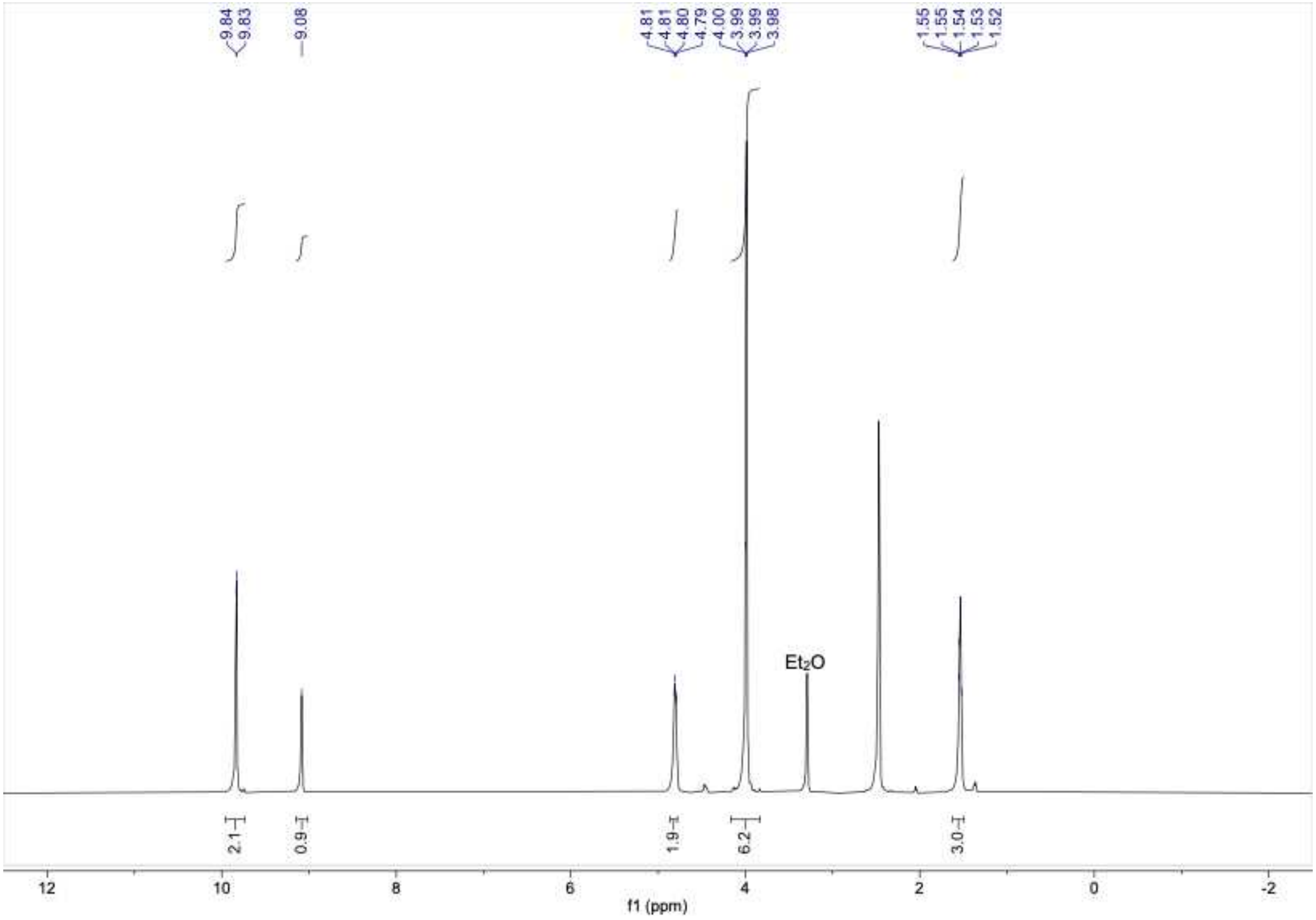

Figure S51. ^{1}H NMR spectrum of **2** (DMSO-d_6, 297 K, 500 MHz).

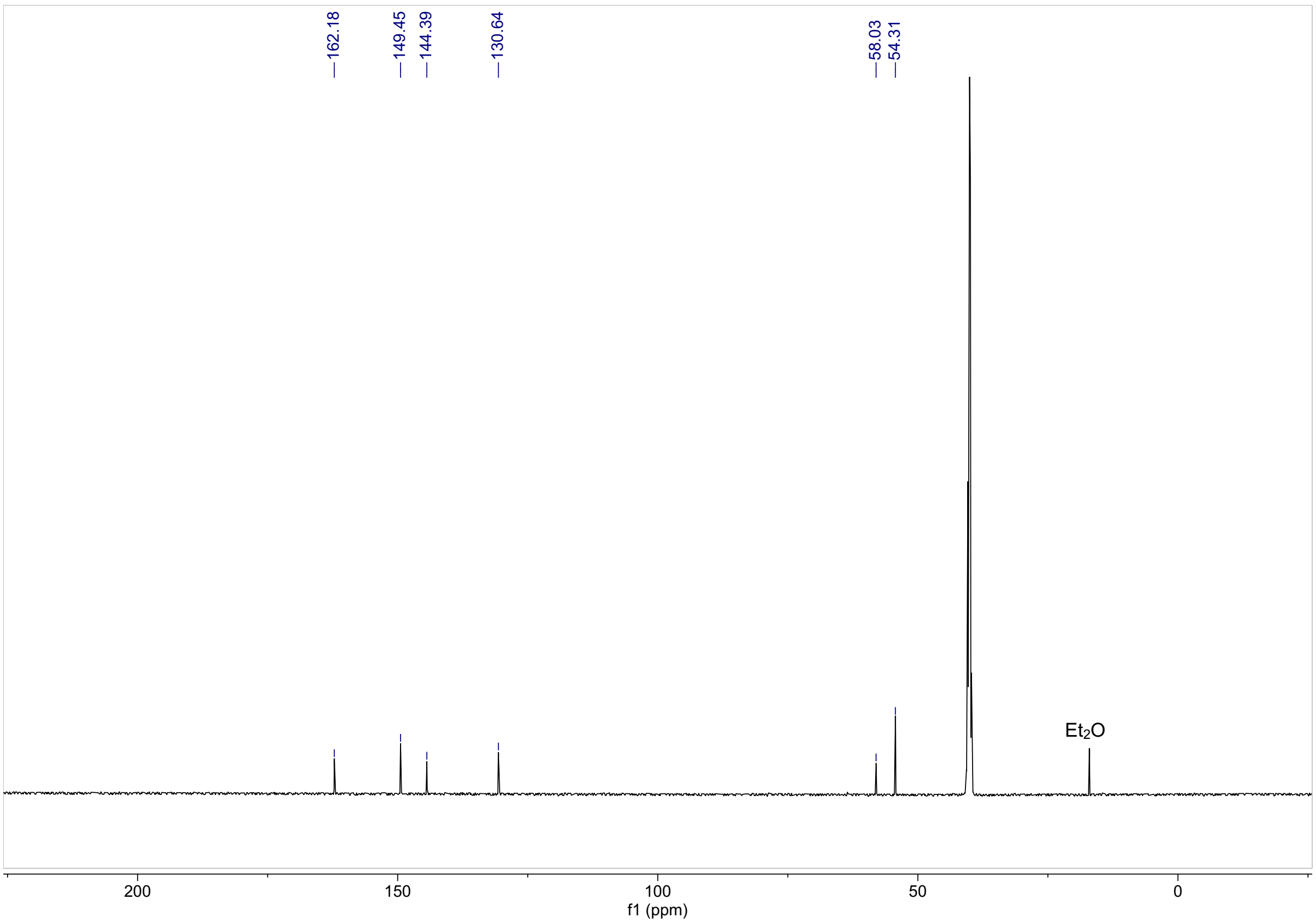

Figure S52. ^{13}C{^{1}H} NMR spectrum of **2** (DMSO-d_6, 297 K, 126 MHz).

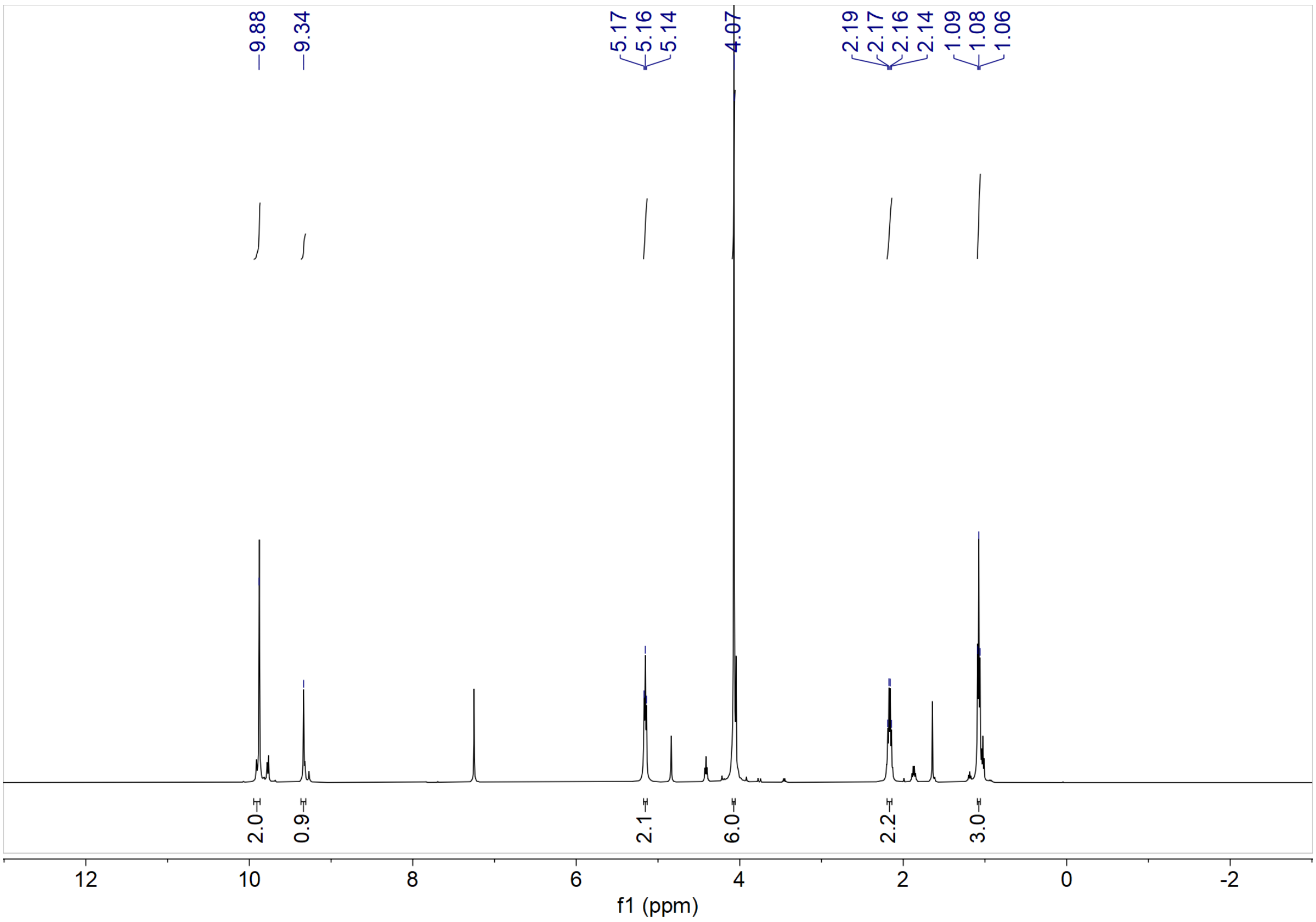

Figure S53. ^{1}H NMR spectrum of **3** ($CDCl_3$, 297 K, 500 MHz).

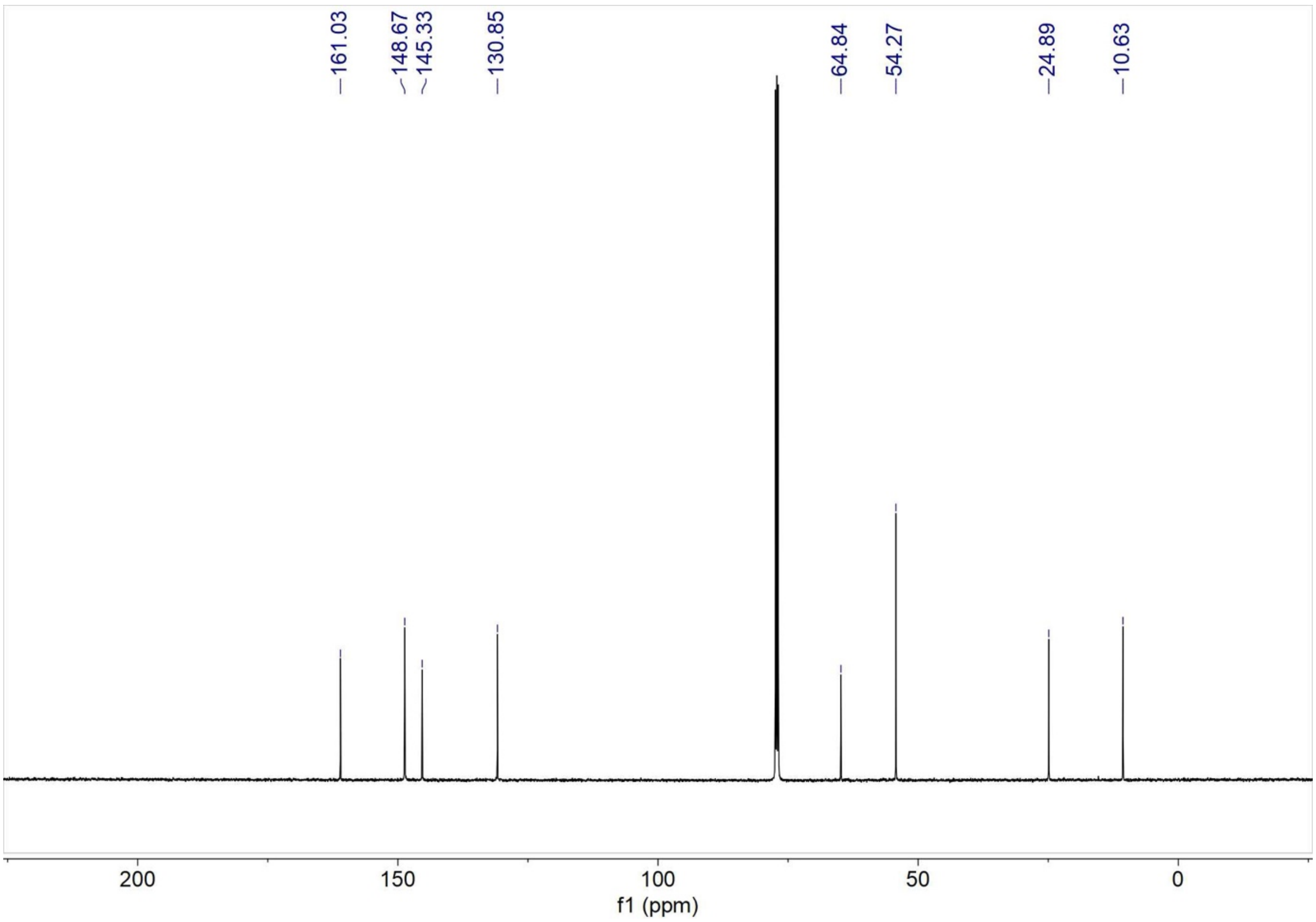

Figure S54. $^{13}C\{^1H\}$ NMR spectrum of **3** ($CDCl_3$, 297 K, 126 MHz).

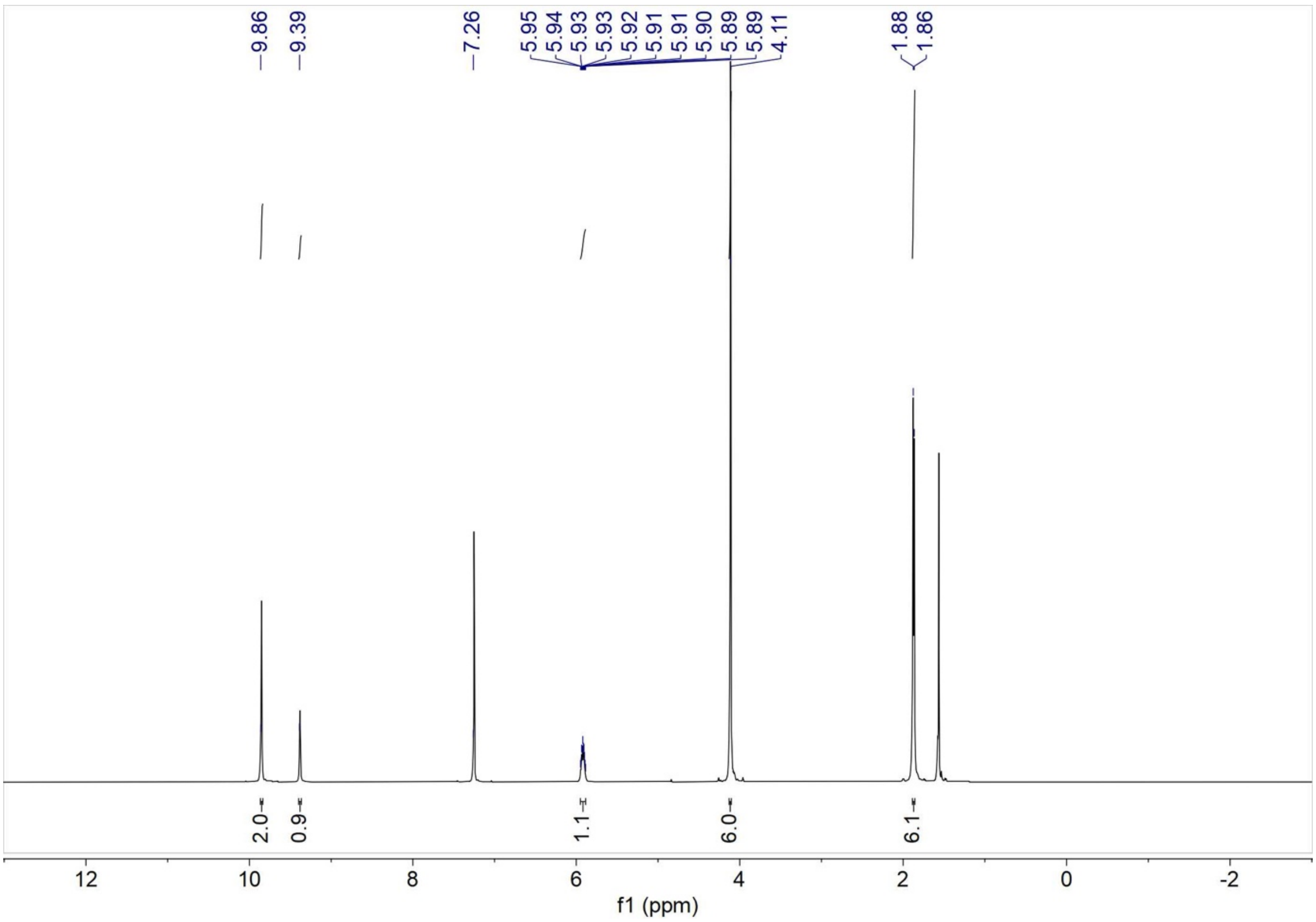

Figure S55. ^{1}H NMR spectrum of **4** ($CDCl_3$, 297 K, 500 MHz).

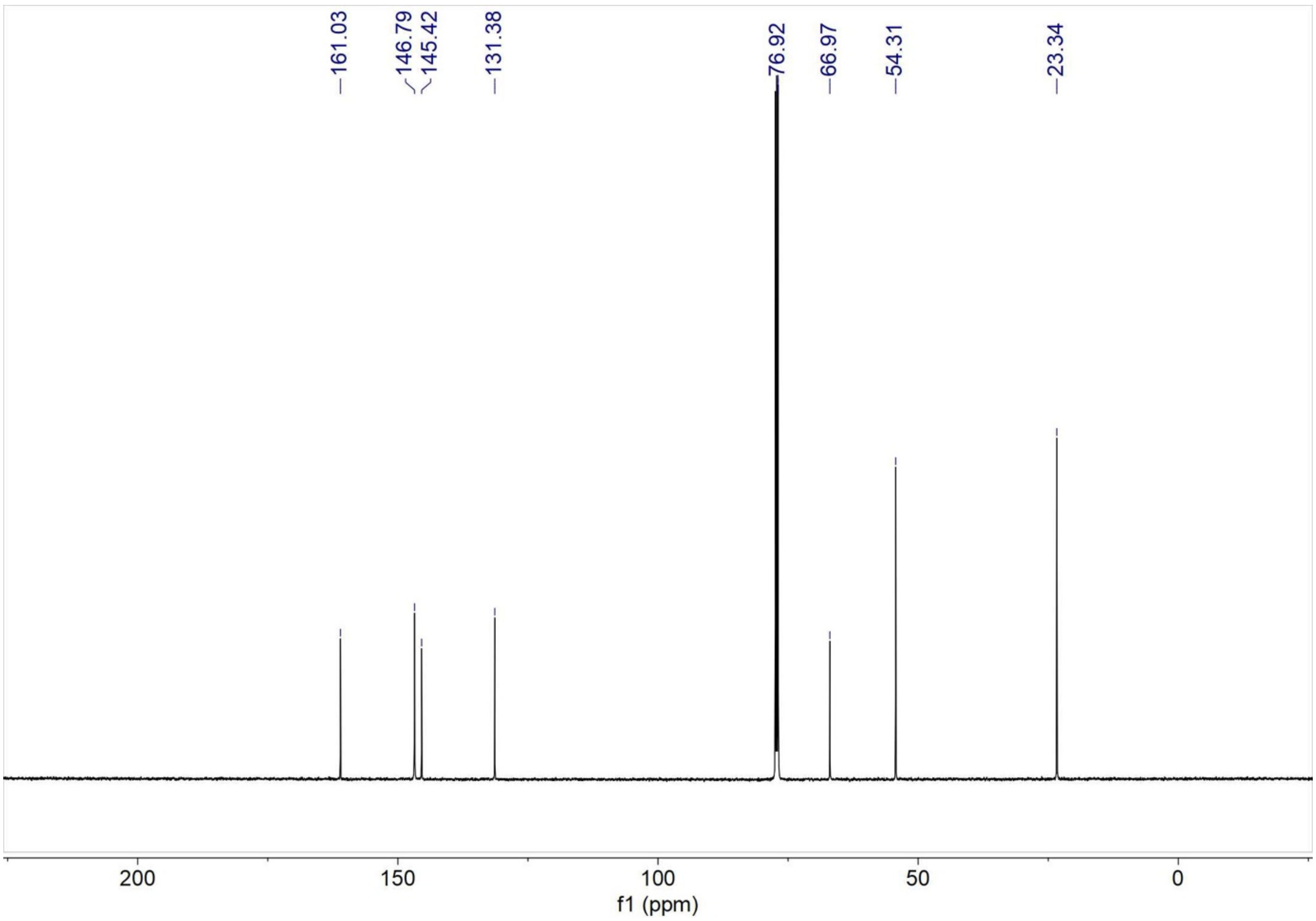

Figure S56. $^{13}C\{^{1}H\}$ NMR spectrum of **4** ($CDCl_3$, 297 K, 126 MHz).

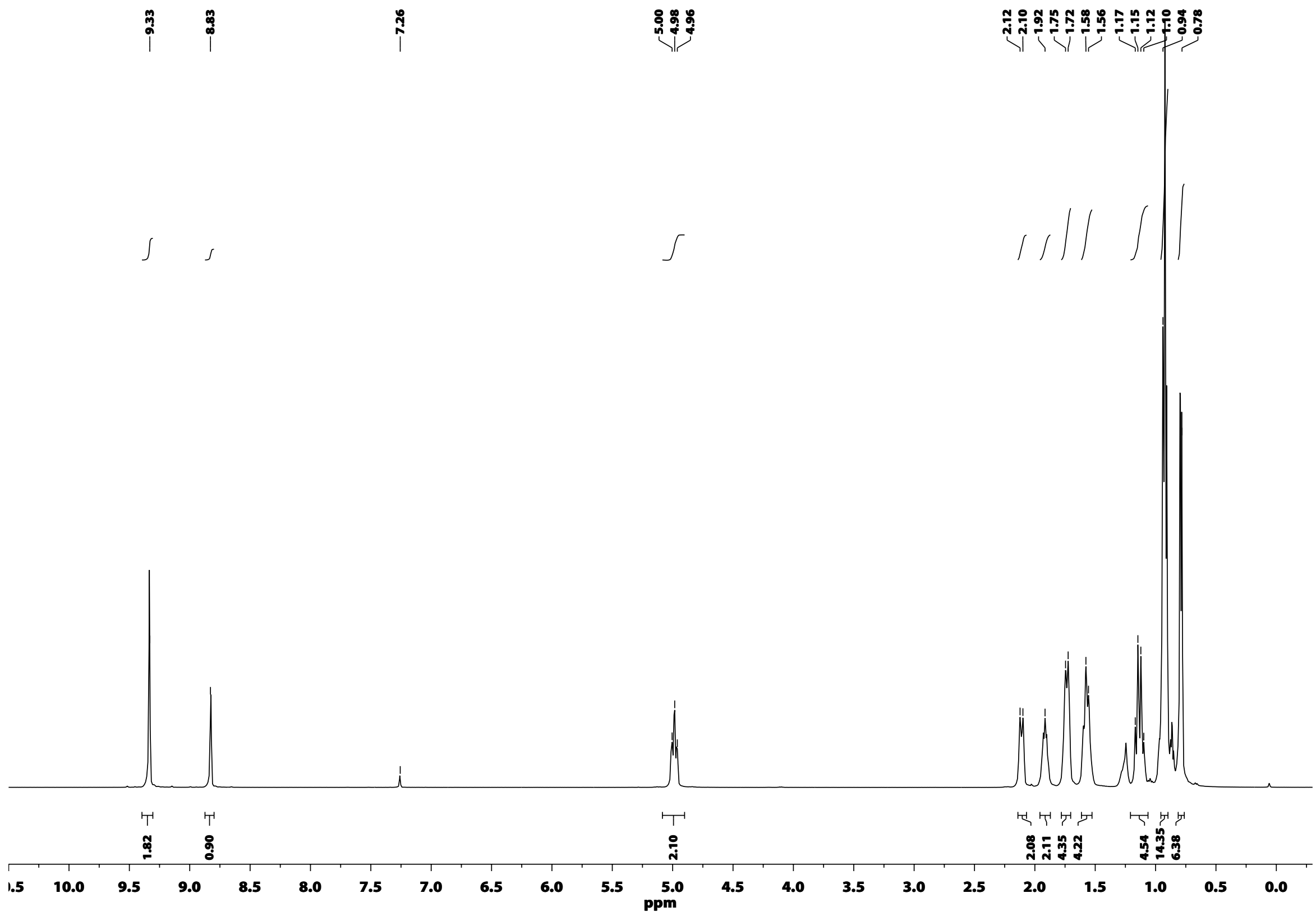

Figure S57. ^{1}H NMR spectrum of **(–)a5** ($CDCl_3$, 297 K, 500 MHz).

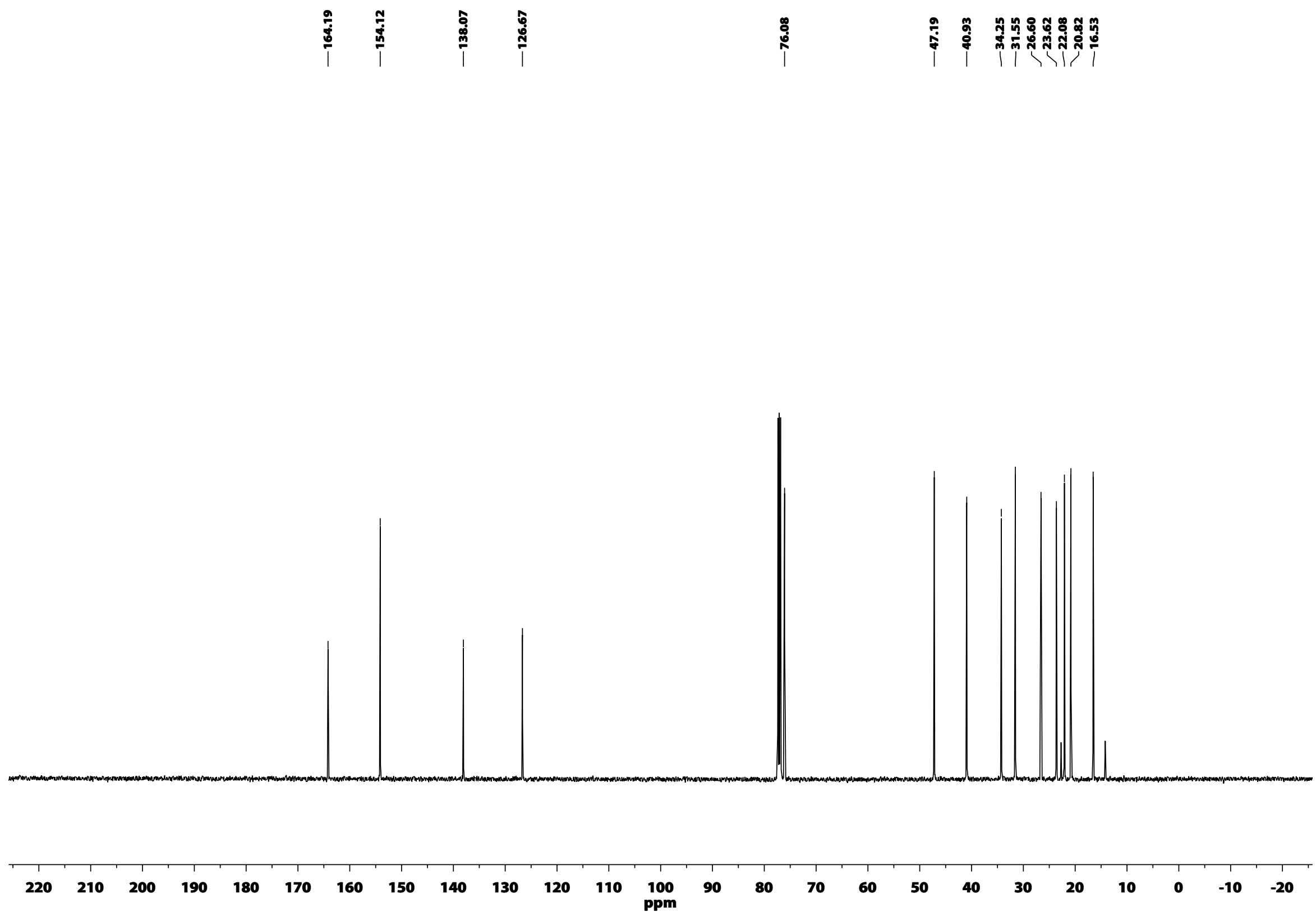

Figure S58. $^{13}C\{^{1}H\}$ NMR spectrum of **(–)a5** ($CDCl_3$, 297 K, 126 MHz).

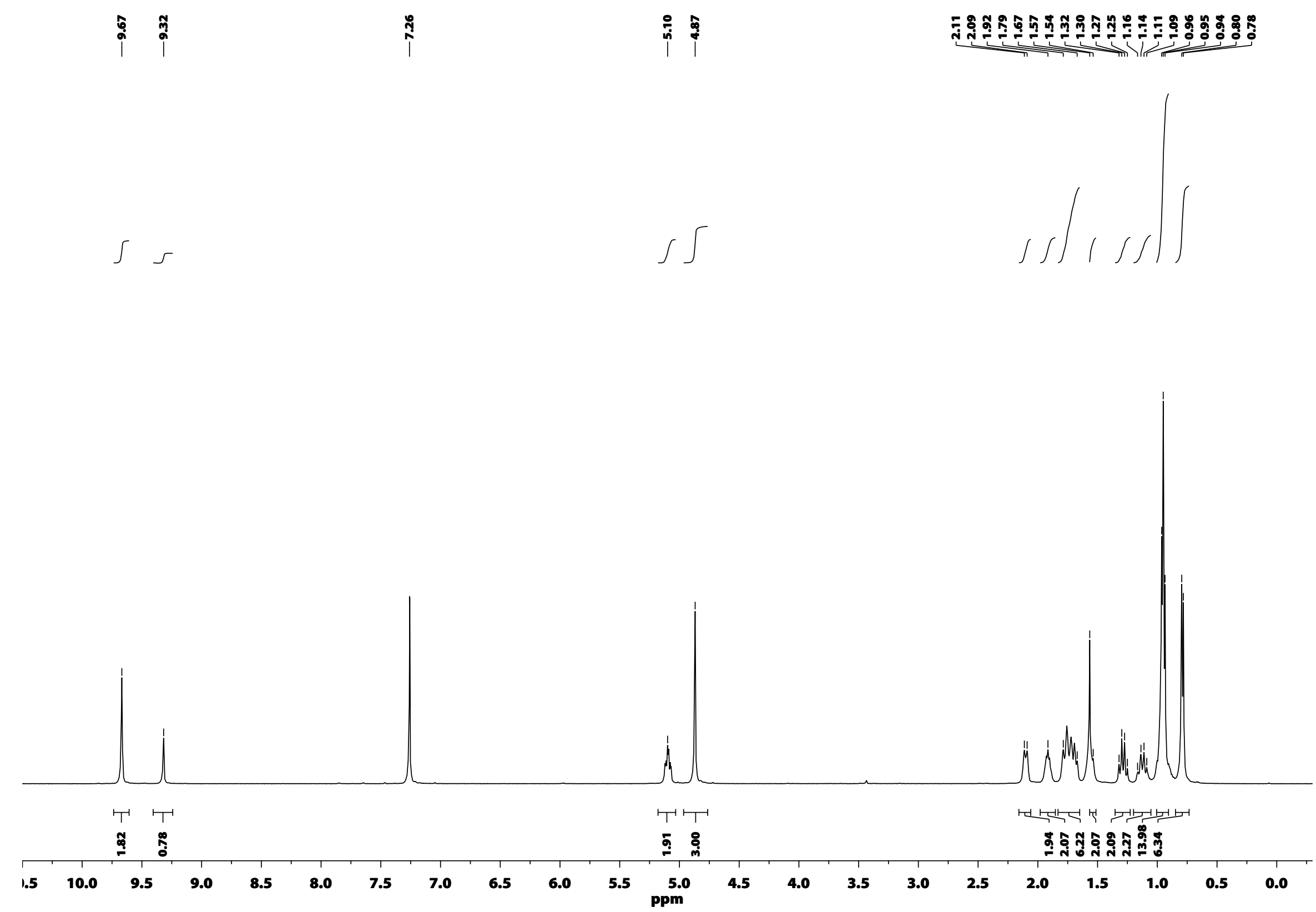

Figure S59. ^{1}H NMR spectrum of $^{-}$**5** ($CDCl_3$, 297 K, 500 MHz).

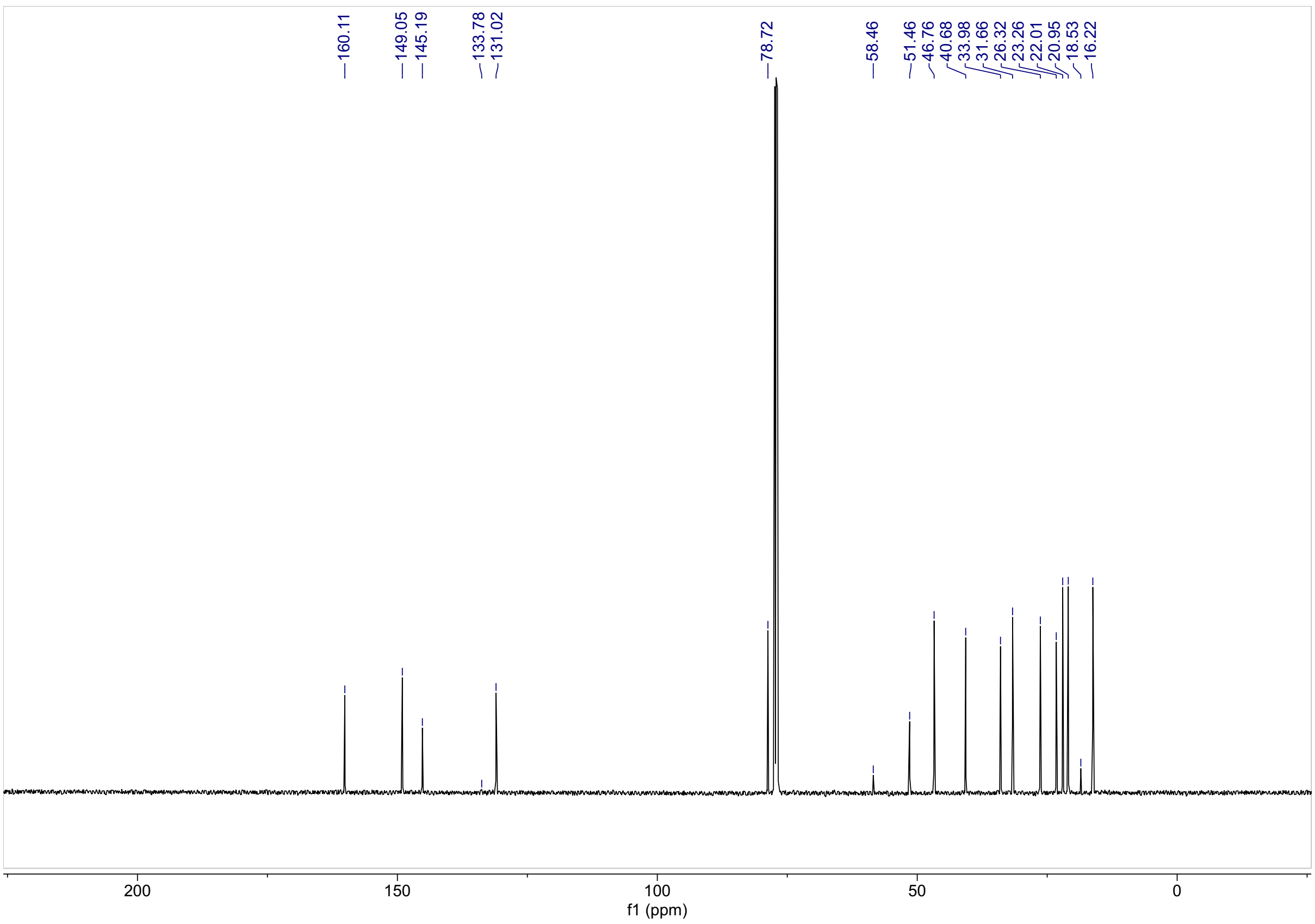

Figure S60. ^{13}C{^{1}H} NMR spectrum of $^{-}$**5** ($CDCl_3$, 297 K, 126 MHz).

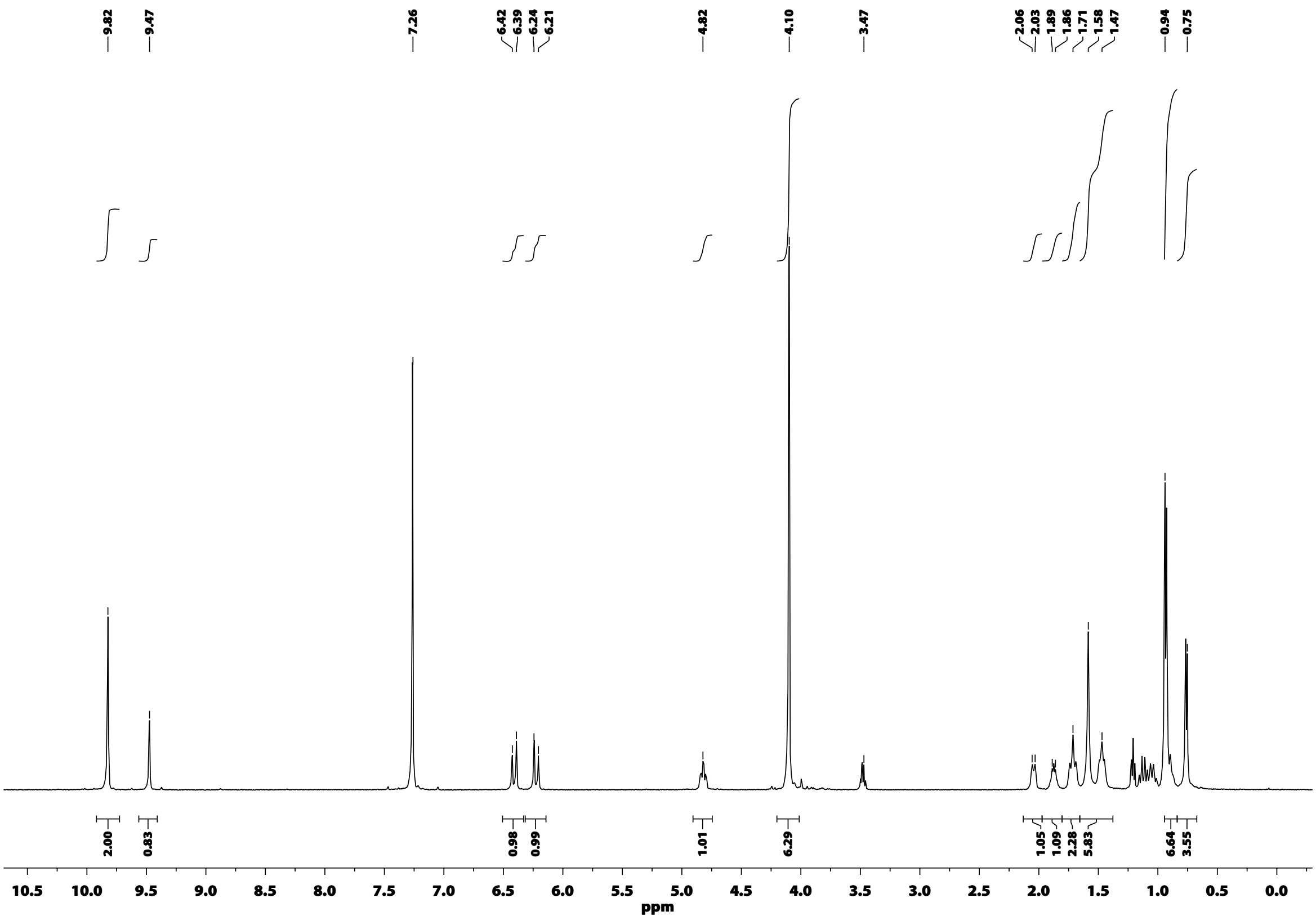

Figure S61. ^{1}H NMR spectrum of **6Br** ($CDCl_3$, 297 K, 500 MHz).

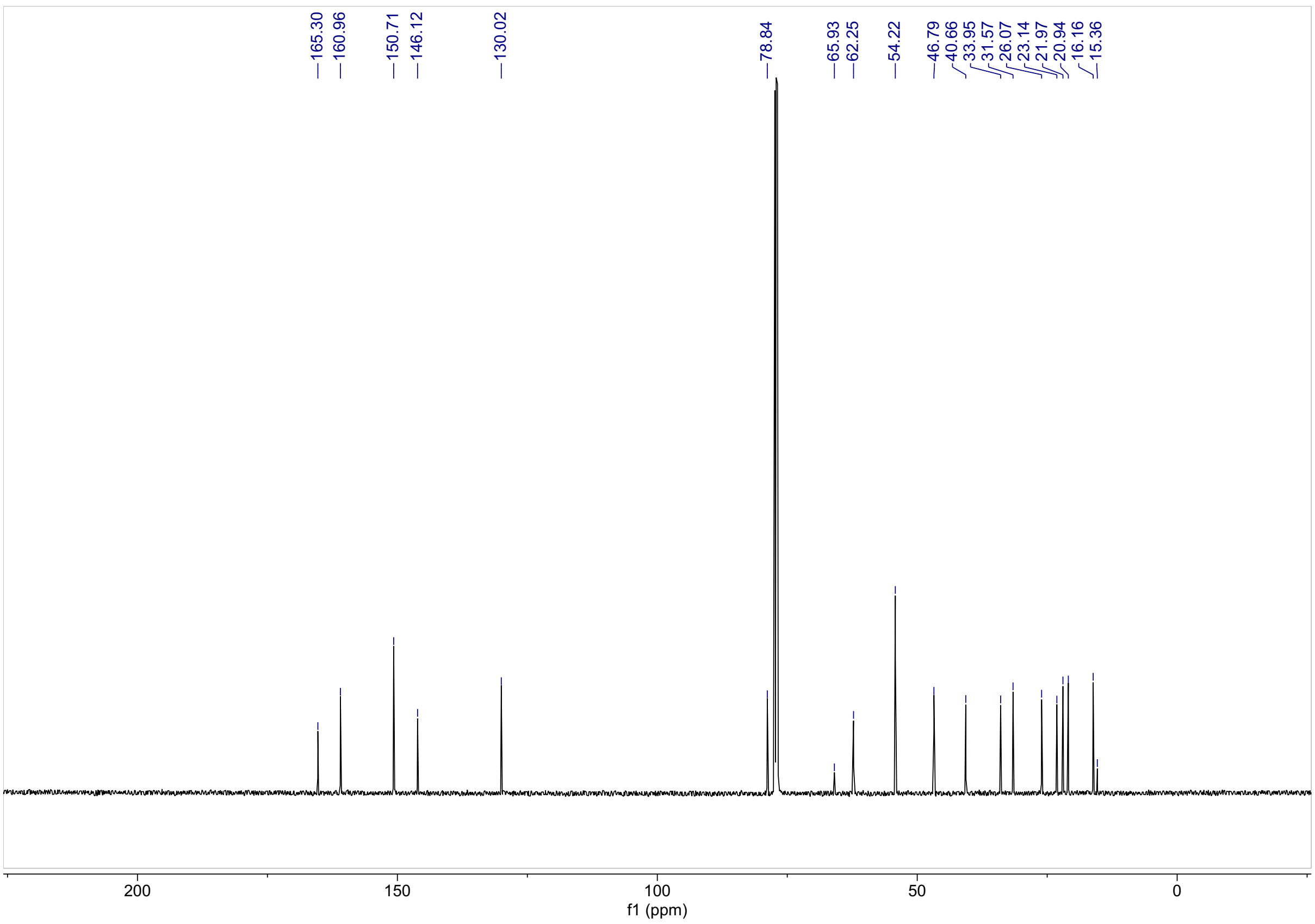

Figure S62. $^{13}C\{^{1}H\}$ NMR spectrum of **6Br** ($CDCl_3$, 297 K, 126 MHz).

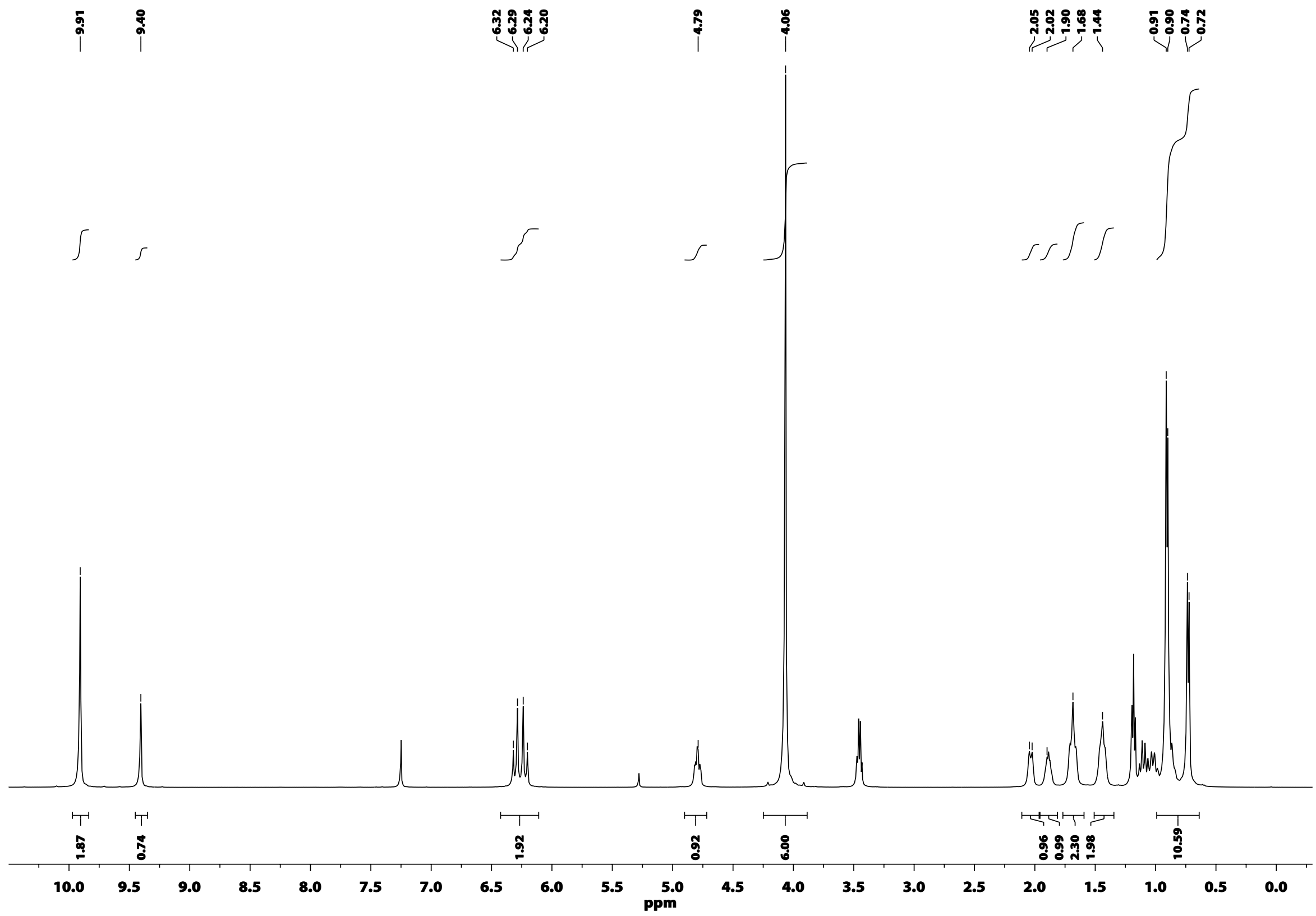

Figure S63. ^{1}H NMR spectrum of **6** ($CDCl_3$, 297 K, 500 MHz).

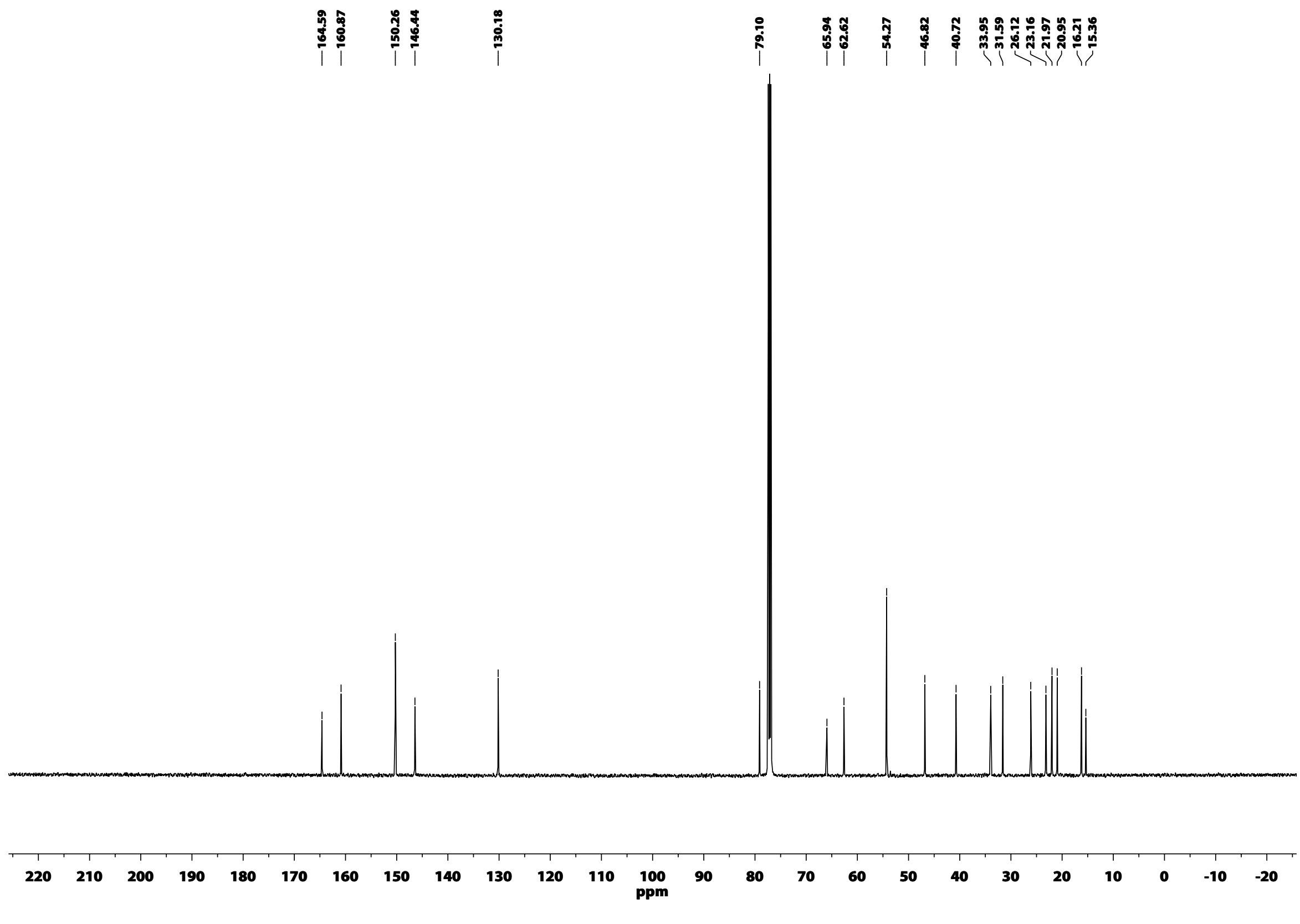

Figure S64. $^{13}C\{^{1}H\}$ NMR spectrum of **6** ($CDCl_3$, 297 K, 126 MHz).

References

[1] D. A. Klumpp, R. M. Sobel, S. G. Kokkinidou, B. Osei-Badu, Z. Liveris, R. A. Klumpp, M. R. Stentzel, "Synthesis of Menthol Glycinates and Their Potential as Cooling Agents" *ACS Omega* **2020**, *5*, 4043–4049.

[2] K. K. Geyl, A. Belyaev, D. Pál, D. Delcourt, J. S. Ward, P. Permi, V. Gervais, R. M. Gomila, A. Frontera, F. B. L. Cougnon, "Multiply entangled receptors for high-affinity anion recognition in water" *Chem* **2026**, *12*, 102936.

[3] E. Bitega, R. Patil, M. Zeller, S. V. Rosokha, "Charge-Assisted Anion−π Interaction and Hydrogen Bonding Involving Alkylpyridinium Cations" *ACS Omega* **2024**, *9*, 43058–43067.

[4] E. M. Kosower, "The Effect of Solvent on Spectra. I. A New Empirical Measure of Solvent Polarity: Z-Values" *J. Am. Chem. Soc.* **2002**, *80*, 3253–3260.

[5] K. N. Truong, S. Ito, J. M. Wojciechowski, C. R. Göb, C. J. Schürmann, A. Yamano, M. Del Campo, E. Okunishi, Y. Aoyama, T. Mihira, N. Hosogi, J. Benet-Buchholz, E. C. Escudero-Adán, F. J. White, J. D. Ferrara, R. Bücker, "Making the Most of 3D Electron Diffraction: Best Practices to Handle a New Tool" *Symmetry (Basel).* **2023**, *15*, 1555.

[6] S. Ito, F. J. White, E. Okunishi, Y. Aoyama, A. Yamano, H. Sato, J. D. Ferrara, M. Jasnowski, M. Meyer, "Structure determination of small molecule compounds by an electron diffractometer for 3D ED/MicroED" *CrystEngComm* **2021**, *23*, 8622–8630.

[7] O. V. Dolomanov, L. J. Bourhis, R. J. Gildea, J. A. K. Howard, H. Puschmann, "OLEX2: a complete structure solution, refinement and analysis program" *J. Appl. Crystallogr.* **2009**, *42*, 339–341.

[8] F. Neese, "Software update: The ORCA program system—Version 5.0" *Wiley Interdiscip. Rev. Comput. Mol. Sci.* **2022**, *12*, e1606.

[9] C. Adamo, V. Barone, "Toward reliable density functional methods without adjustable parameters: The PBE0 model" *J. Chem. Phys.* **1999**, *110*, 6158–6170.

[10] F. Weigend, R. Ahlrichs, "Balanced basis sets of split valence, triple zeta valence and quadruple zeta valence quality for H to Rn: Design and assessment of accuracy" *Physical Chemistry Chemical Physics* **2005**, *7*, 3297–3305.

[11] B. De Souza, G. Farias, F. Neese, R. Izsák, "Predicting Phosphorescence Rates of Light Organic Molecules Using Time-Dependent Density Functional Theory and the Path Integral Approach to Dynamics" *J. Chem. Theory Comput.* **2019**, *15*, 1896–1904.

[12] D. Peng, N. Middendorf, F. Weigend, M. Reiher, "An efficient implementation of two-component relativistic exact-decoupling methods for large molecules" *Journal of Chemical Physics* **2013**, *138*, 184105.

[13] P. Pollak, F. Weigend, "Segmented Contracted Error-Consistent Basis Sets of Double- and Triple-ζ Valence Quality for One- and Two-Component Relativistic All-Electron Calculations" *J. Chem. Theory Comput.* **2017**, *13*, 3696–3705.

[14] B. De Souza, F. Neese, R. Izsák, "On the theoretical prediction of fluorescence rates from first principles using the path integral approach" *Journal of Chemical Physics* **2018**, *148*, 034104.

[15] F. Neese, "An improvement of the resolution of the identity approximation for the formation of the Coulomb matrix" *J. Comput. Chem.* **2003**, *24*, 1740–1747.

[16] F. Neese, F. Wennmohs, A. Hansen, U. Becker, “Efficient, approximate and parallel Hartree–Fock and hybrid DFT calculations. A ‘chain-of-spheres’ algorithm for the Hartree–Fock exchange” *Chem. Phys.* **2009**, *356*, 98–109.
[17] B. Helmich-Paris, B. de Souza, F. Neese, R. Izsák, “An improved chain of spheres for exchange algorithm” *Journal of Chemical Physics* **2021**, *155*, 104109.
[18] F. Neese, “The SHARK integral generation and digestion system” *J. Comput. Chem.* **2023**, *44*, 381–396.
